# Metal-Free Reductive Cleavage of C–N and S–N Bonds by Photoactivated Electron Transfer from a Neutral Organic Donor**

**DOI:** 10.1002/anie.201306543

**Published:** 2013-12-06

**Authors:** Steven O'Sullivan, Eswararao Doni, Tell Tuttle, John A Murphy

**Affiliations:** WestCHEM, Department of Pure and Applied Chemistry, University of Strathclyde295 Cathedral Street, Glasgow G1 1XL (UK)

**Keywords:** electron transfer, cleavage reactions, photochemistry, radical ions, reduction

## Abstract

A photoactivated neutral organic super electron donor cleaves challenging arenesulfonamides derived from dialkylamines at room temperature. It also cleaves a) ArC–NR and b) ArN–C bonds. This study also highlights the assistance given to these cleavage reactions by the groups attached to N in (a) and to C in (b), by lowering LUMO energies and by stabilizing the products of fragmentation.

Recently, we have developed a range of highly reactive organic electron donors, including **1**–**3** (Scheme 1). These compounds undergo oxidation to radical cations and dications after loss of one and two electrons, respectively, and the aromaticity of these products contributes to the driving force for the oxidations.^1^ The radical cation **4** and dication **5** derived from **3** are shown in Scheme 1. The donor **1** was the first neutral organic compound to reductively cleave aryl iodides to aryl radicals,1a while the stronger donors **2**1b and **3**1d converted aryl iodides into aryl anions. The compounds **2** and **3** also reduced Weinreb amides,1f acyloin derivatives,1h and some sulfones.1c

**Scheme 1:**
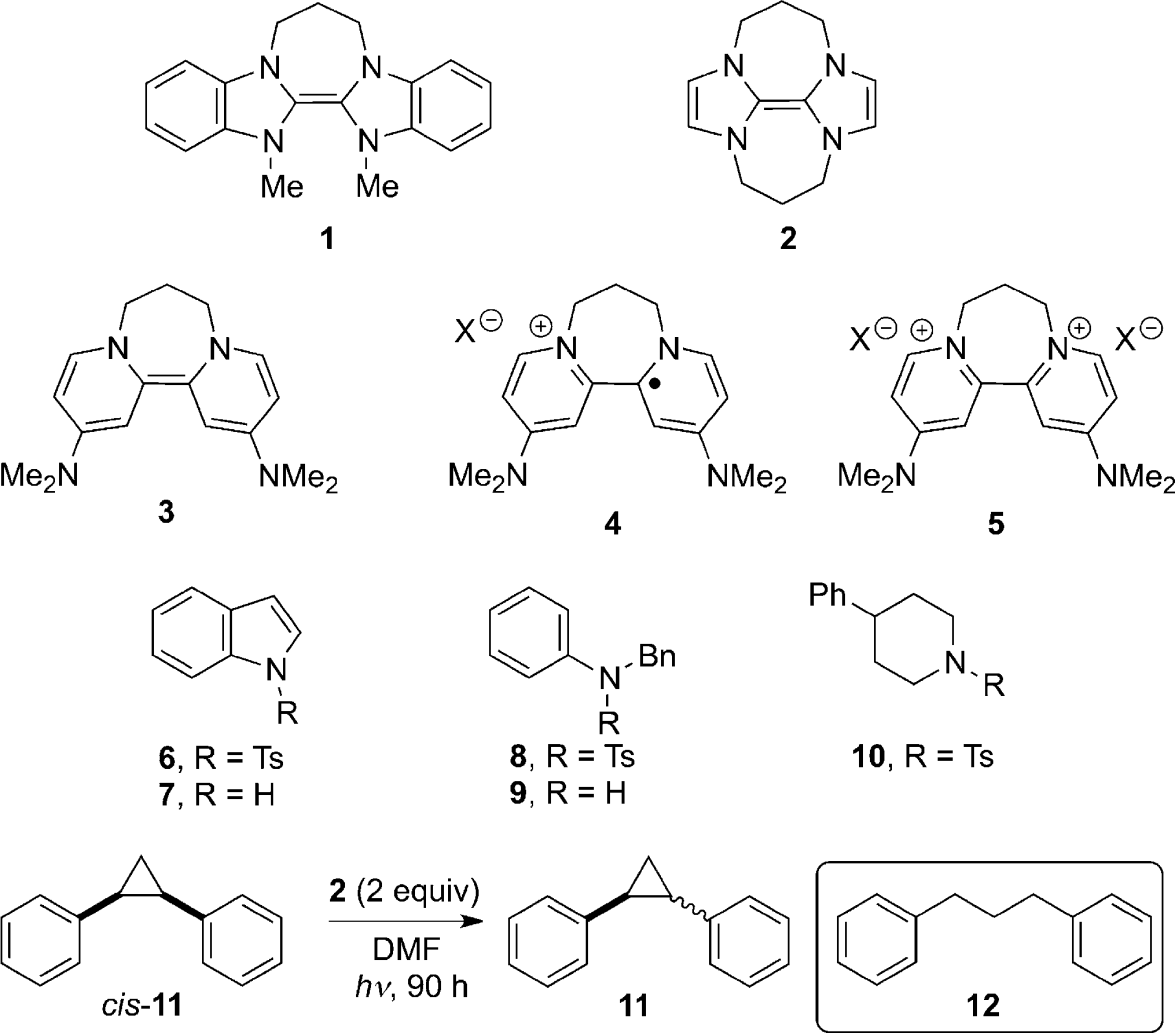
Protected amines and neutral organic electron donors. DMF=*N*,*N*-dimethylformamide, Ts=4-toluenesulfonyl.

The donor **2** also cleaved arenesulfonamides, but not N,N-dialkyl arenesulfonamides.1c For the sulfonamides **6**, **8**, and **10**, the electron is transferred from the donor to the arenesulfonyl unit, where the LUMO is located. The substrates **6** and **8** underwent efficient reductive cleavage of the N–S bond to afford the corresponding amines **7** and **9** [using donor **2** (6 equiv), DMF, 110 °C, 18 h]. In these cases, the nitrogen leaving groups are stabilized by resonance. However, the sulfonamide **10**, which, after fragmentation, would produce a nitrogen-centred leaving group which is unstabilized by resonance, remained completely unchanged.

Most recently, our donors **2** and **3**, vivid yellow and purple solids, respectively, were tested under photoactivation conditions and proved even more powerful than in the ground state, in that they were now able to reductively cleave the Ar–Cl bond in chlorobenzenes, a reaction which had never been seen with our ground-state electron donors.1q In addition, they were able to donate an electron to the *cis-*diphenylcyclopropane **11**, ultimately affording the 1,3-diphenylpropane **12** as a product.1q

These advances encouraged us to test the photoactivated **3** in other very challenging transformations, that is, the reductive cleavage of 1) difficult arenesulfonamides like **10**,^2^ and 2) N-benzyl groups,^3^ and we report herein our results. The donor **3** was selected since it is as strong as **2**, but is much more conveniently prepared.

The sulfonamides **10**, **14**, and **16** were chosen as substrates for reduction (Scheme 2). Fragmentation of their radical anions would give rise to a nitrogen-centred leaving group which would not be stabilized by resonance. Under photoactivated conditions (*λ*=365 nm, 2×100 W) at room temperature, each of the substrates underwent cleavage to afford the parent amine in good yield after work-up. The *λ*=365 nm irradiation does not overlap with the chromophore of the sulfonamides, and hence it is the highly colored **3** which undergoes photoactivation. This result marks the first time that dialkyl arenesulfonamides have been reductively cleaved by a neutral organic electron donor.

**Scheme 2:**
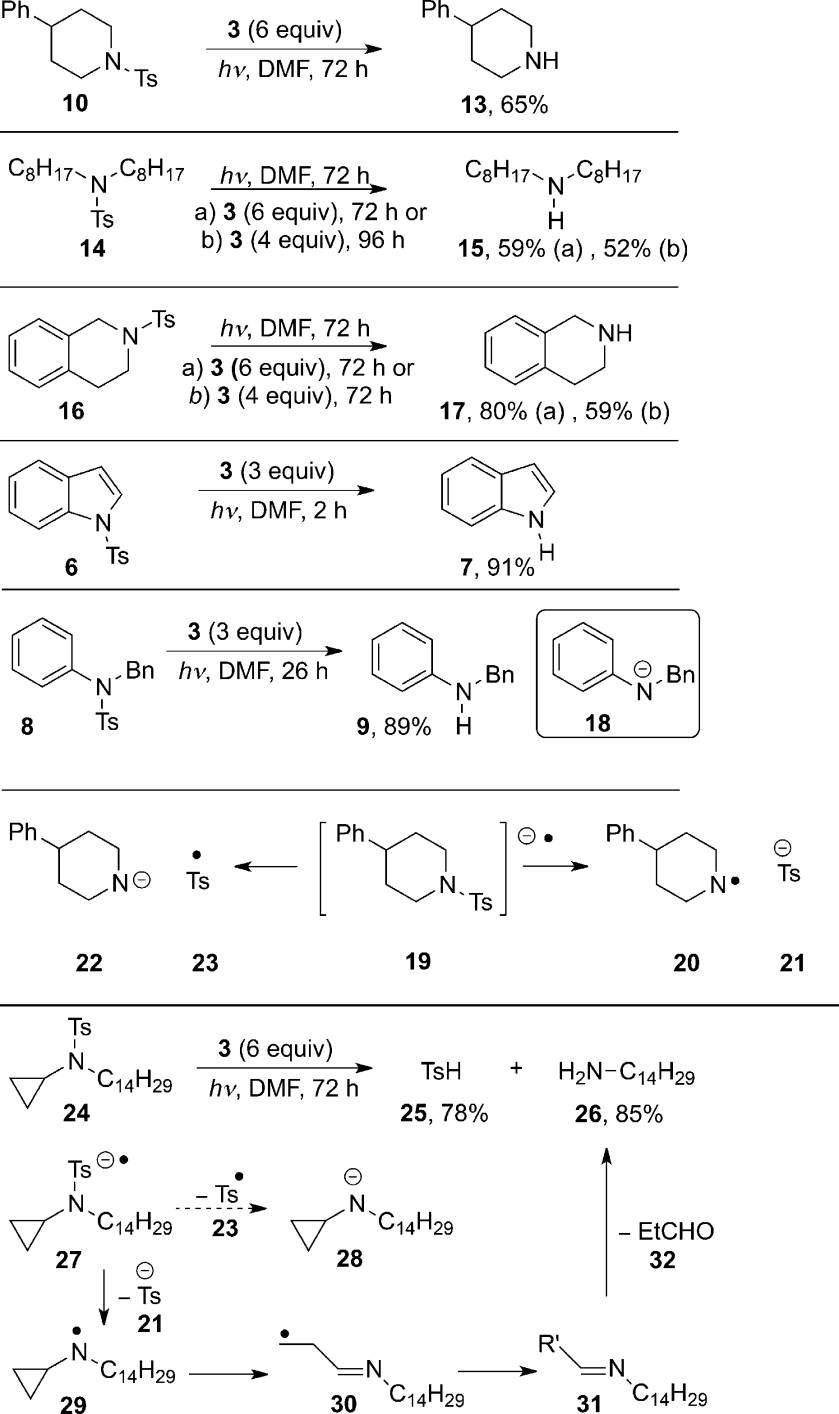
Reactions of sulfonamides with the photoactivated donor 3.

To verify the nature of the activation, two types of blank experiments were also conducted for the substrates **10**, **14**, and **16**. These blank reactions were conducted a) without **3**, but in the presence of photoactivation, and b) with **3**, but in the absence of photoactivation. In all cases, no product was formed and the starting substrate was recovered in excellent yield (see the Supporting Information). This reinforced the message that photoactivation of the donor (or the donor–substrate complex; see the Supporting Information), significantly enhances the driving force for electron transfer.

For completeness, the more reactive substrates, **6** and **8**, were also treated with **3** under photoactivated conditions (Scheme 2). They underwent efficient cleavage, as expected, with the products **7** and **9**, respectively, being isolated in excellent yield after shorter reaction times in the presence of three equivalents of **3**.

For the radical anion **19**, formed at the sulfonamide group in **10** after electron transfer (Scheme 2), we calculated whether fragmentation to either the dialkylamide anion **22** and a sulfonyl radical **23**, or the dialkylaminyl radical **20** and a sulfinate anion **21** would be preferred.^4^ Density functional theory^5^, ^6^ (DFT) calculations were employed to optimize all structures with the gradient corrected B97-D functional with a long-range dispersion correction.^7^ All atoms were described with the 6-311++G(d,p) basis set.^8^, ^9^ Subsequent single-point energy calculation of the optimized geometry was performed at the same level of theory within a polarizable continuum model (CPCM)^10^ with the dielectric constant of *N*,*N*-dimethylformamide (DMF, *ε*=37.219; see the Supporting Information for details). These studies indicated that fragmentation of the radical anion **19** into **20** and **21** is thermodynamically preferred, both when **19** alone is considered as an entity, and also when a complex between the radical cation of the donor and **19** is considered. Experimental evidence in favor of the formation of an aminyl radical following fragmentation was also seen when substrate **24** was subjected to cleavage. Here fragmentation into the toluenesulfinyl radical **23** and dialkylamide anion **28** should lead to the isolation of the corresponding amine, *N*-cyclopropyl-*N*-tetradecylamine, upon work-up. In contrast, cleavage to **21** and the aminyl radical **29** should lead to rapid opening of the cyclopropane ring to afford the imine **30**, which could undergo quenching of its radical in a number of ways1k to form the imine product **31**, and thus undergo hydrolysis under mild work-up conditions to afford the tetradecylamine **26**. In fact, the experiment afforded **26** (85 %) and **25** (78 %), thus supporting **29** as an intermediate.

The next task was to investigate whether N-benzyl groups could be cleaved. The outcome from the substrate **8** deserves comment because it shows no cleavage of the benzyl group. This is in accord with expectations in this case, as the LUMO is localized on the electron-poor *p*-toluenesulfonyl (tosyl) group, rather than on the benzyl group. When the S–N bond is cleaved, the aniline anion **18** results, and the arene ring is too electron-rich to receive another electron. Either during the reaction or upon work-up, **18** undergoes protonation to **9**.

To modify the structure of **8** so that cleavage of an N-benzyl bond might occur, we designed substrates such that the benzyl group was the site of the LUMO within the substrate. To this end, benzyl alkyl methanesulfonamide derivatives (**33 a**–**i**) were chosen (Table 1). DFT studies (B3LYP / 6-31G*), taking **33 c** and **33 g** as examples, in a DMF solvent continuum indicated that the LUMO of these compounds was located on the benzyl group. If electron transfer occurred to the benzyl group, then the leaving group would likely be the sulfonamide anion, and this would be protonated to form **34** upon work-up. Upon trying the reactions, very good yields of N-benzyl bond cleavage were seen in each case (Table 1). The substrate **33 e** features a benzyl group and a dimethoxybenzyl group. The outcome shows competitive cleavage of these two benzyl groups, with marginal selectivity for the formation of 34 e*, which is consistent with a very slightly preferential electron transfer to the less electron-rich aryl ring, that is, the C_6_H_5_ ring. To show that photoactivation was required for these reactions, **33 g** was subjected to a parallel reaction in which photoactivation was omitted. This reaction afforded an excellent recovery of the unchanged **33 g** (94 %).

**Table 1:** Reductive deprotection of benzyl methanesulfonamides (33) with 3
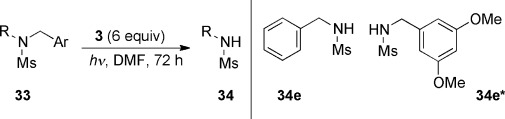

Substrate	R	Ar	33 Yield [%]^[a]^	34 Yield [%]^[b]^
**33 a**	CyCH_2_	3,5-(MeO)_2_C_6_H_3_	**33 a**: 9	**34** **a**: 80
**33 b**	*i-*pentyl	3,5-(MeO)_2_C_6_H_3_	**33 b**: 12	**34** **b**: 80
**33 c**	*i-*butyl	3,5-(MeO)_2_C_6_H_3_	**33 c**: 0	**34** **c**: 79
**33 d**	C_12_H_25_	3,5-(MeO)_2_C_6_H_3_	**33 d**: 21	**34** **d**: 64
**33 e**	C_6_H_5_CH_2_	3,5-(MeO)_2_C_6_H_3_	**33 e**: 7	**34** e*: 35 +**34** **e**: 28
**33 f**	C_12_H_25_	C_6_H_5_	**33 f**: 15	**34** **d**: 80
**33 g**	CyCH_2_	C_6_H_5_	**33 g**: 14	**34** **a**: 71
**33 h**	*n*-butyl	4-(CF_3_)C_6_H_4_	**33 h**: 0	**34** **h**: 84
**33 i**	Cy	4-(CF_3_)C_6_H_4_	**33 i**: 0	**34** **i**: 75

[a] Recovered starting material. [b] Yield of isolated product. Ms=methanesulfonyl.

Since benzyl methanesulfonamides had worked so well we next investigated the more challenging allyl methanesulfonamides. Because these compounds have less extensive π systems, their LUMO energies are expected to be higher than their benzyl counterparts. In support of this, the mixed allyl benzyl substrate **35 a** showed selectivity for the benzyl cleavage to **37** (62 %; Table 2). This outcome was in line with expectations since the LUMO of this substrate (and the SOMO of its radical anion) were associated with the arene ring, rather than with the allyl group or with the sulfonyl group. However, the presence of some product resulting from allyl cleavage, that is, **36 a** (10 %), encouraged us to think that substrates which did not feature an N-benzyl group might undergo cleavage of the allyl group. This selectivity in **35 a** for benzyl cleavage over allyl cleavage contrasts with that seen in palladium-induced reduction of benzyl allylamines where the affinity of Pd^0^ for olefins dominates the reactivity.^11^ It also surprisingly contrasts with the selectivity in favor of deallylation seen in the reductive deprotection of sugars with SmI_2_ reported by Hilmersson et al.^12^

**Table 2:** Reductive deprotection of allyl methanesulfonamides 35
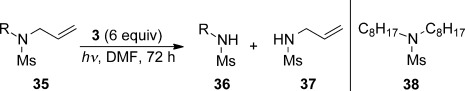

Substrate	R	35 Yield [%]^[a]^	36 Yield [%]^[b]^	37 Yield [%]^[b]^
**35 a**	C_6_H_5_CH_2_	**35 a**: 15	**36 a**: 10	62
**35 b**	C_6_H_5_(CH_2_)_2_	**35 b**: 57	**36 b**: 41	0
**35 c**	C_6_H_5_(CH_2_)_3_	**35 c**: 47	**36 c**: 42	0
**35 d**	C_12_H_25_	**35 d**: 32	**36 d**: 63^[c]^	0
**35 e**	*i*-pentyl	**35 e**: 38	**36 e**: 50	0

[a] Recovered starting material. [b] Yield of isolated product. We recognize that **36 a**=**34 e**, **36 d**=**34 d**, and **36 e**=**34 b**. [c] When additional donor **3** (6 equiv) was added after 72 h, and the reaction continued for a further 72 h, **36 d** (81 %) was isolated.

When the allyl alkyl methanesulfonamides **35 b**–**e** were treated under photoactivation conditions with **3**, cleavage of the allyl group was exclusively seen, with moderate to good yields of the products **36** being isolated. Taking the substrates **35 d** and **35 e** as examples, the LUMO of the substrates (and the SOMO of their radical anions) are sited on the allyl groups, thus allowing the selectivity of the observed reactions to be easily understood. In **35 b** and **35 c**, the LUMO lies on the aryl ring, however, the radical anion shows spontaneous cleavage of the allyl group. In this case, electron transfer to the arene should occur preferentially. There is no driving force for fragmentation of the arene radical anion in these two cases, since that would give an alkyl leaving group unstabilized by resonance, so intramolecular electron transfer to the allyl group can occur, thus leading to the observed fragmentation. To explore whether photoactivation was needed to trigger these reactions, the substrate **35 b** was subjected to the same reaction conditions, except that no photoactivation was provided. In this case, no deprotection occurred and **35 b** was recovered in quantitative yield.

This ability to transfer an electron to an *N*-allylsulfonamide takes the photoactivated electron donors into new territory, as no previous deallylation reaction has been reported. To check if the allyl group was really needed, or if N,N-dialkyl methanesulfonamides would undergo reaction by electron transfer to the sulfonyl group, the *N*,*N*-dioctyl methanesulfonamide **38** was subjected to reaction with the photoactivated **3**. In this case, no new product was detected and the starting material **38** (92 %) was recovered unchanged.

The ability to transfer an electron to an ArC–N ring group is evident in the above results with the substrates **33 a**–**i**, and this led us to investigate what happens in the transposed case, that is, ArN–C. An amine nitrogen atom directly attached to the arene should make electron transfer to the arene more difficult, but the accessibility of the LUMO for electron transfer should depend upon the third group attached to the nitrogen atom. With the simple *N-*methyl-*N*-allylaniline **39 a**, very little cleavage occurred, but the product that was isolated, **40 a** (6 %), showed cleavage of the N-allyl bond (Table 3). To better facilitate the cleavage reaction, the N*-*Me group was replaced by an N-acyl group. The electron-withdrawing acyl group can lower the LUMO energy and hence make electron transfer to the LUMO easier. In the event, protection of the nitrogen atom as an acetamide (**39 c**), a pivalamide (**39 d**), and a urethane (**39 e**), all enhanced the cleavage of the allyl group.^13^ A blank experiment was also conducted on **39 c** (in the absence of photoactivation) and this showed no conversion into the product, but rather quantitative recovery of **39 c**, thus illustrating the essential role of the photoactivation of the donor. The pivalamide was most successful, thus affording the product **40 d** in 83 % yield. The significant difference in efficiency between **39 c** and **39 d** led us to investigate whether deprotonation of the acetyl group by the basic donors might be occurring. When a repeat of the experiment with **39 c** was subjected to addition to D_2_O, as opposed to H_2_O, prior to acidification and extraction, both the product **40 c** and the recovered starting material **39 c** showed incorporation of a single deuterium atom by mass spectrometry. For further thoughts on the role of deprotonation, see discussion of reactivity of substrate **44**.

**Table 3:** Reductive deprotection of allylanilines with electron donor 3
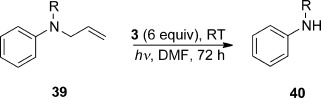

Substrate	R	39 Yield [%]^[a]^	40 Yield [%]^[b]^
**39 a**	Me	**39 a**: 62	**40 a**: 6
**39 b**	allyl	**39 b**: 81	**40 b**: 7
**39 c**	COMe	**39 c**: 59	**40 c**: 33
**39 d**	CO*t*Bu	**39 d**: 8	**40 d**: 83
**39 e**	CO_2_Et	**39 e**: 37	**40 e**: 58

[a] Recovered starting material. [b] Yield of isolated product.

Since the ease of bond cleavage in the radical anion seems to correlate with the stabilization given to the radical and anion products, then replacing the alkene of the allyl group in **39** by a carbonyl group, as in **41**, (Table 4) might additionally facilitate the cleavage reactions, since the anionic leaving group would be an enolate, in place of an allyl anion. Accordingly, the substrates **41 a**–**c** were prepared. Encouragingly, the N-methyl substrate **41 a** showed a higher yield of cleavage product [here **40 a** (34 %)] than had been seen for the corresponding allyl case, **39 a** (6 %). The N-acetyl and the N-carbethoxy cases, **41 b** and **41 c**, respectively, underwent very efficient reaction (74 % and 92 % yield of products respectively) with loss of the CH_2_CO_2_Et side chain. This outcome shows that ArN–C bonds are also subject to reductive cleavage, and that the efficiency of cleavage correlates with stabilization of the radical and anion produced. A repeat reaction was carried out for **41 b**, but in the absence of photoactivation. This reaction gave no **40 c**, but gave recovered starting material (**41 b**, 91 %). A final example of this series, **41 d**, was reacted to give a comparison with other sulfonamide substrates reported herein, and this afforded **42** (89 %), the expected product of fragmentation of the arenesulfonamide radical anion.

**Table 4:** Reductive deprotection of *N-*(acylmethyl)anilines with electron donor 3
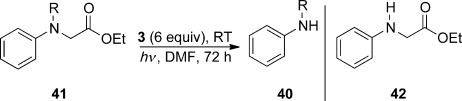

Substrate	R	41 Yield [%]^[a]^	Product Yield [%]^[b]^
**41 a**	Me	**41 a**: 58	**40 a**: 34
**41 b**	COMe	**41 b**: 25	**40 c**: 74
**41 c**	CO_2_Et	**41 c**: 0	**40 e**: 92
**41 d**	Ts	**41 d**: 0	**42**: 89

[a] Recovered starting material. [b] Yield of isolated product.

To verify the importance of the aryl group, the substrate **43** was next prepared (Scheme 3). If electron transfer to the ester group occurred, then cleavage of the N–CH_2_CO_2_Et bond might have been expected, but none was seen. Accordingly, the N-aryl group is crucial for the N–C cleavage to occur.

**Scheme 3:**
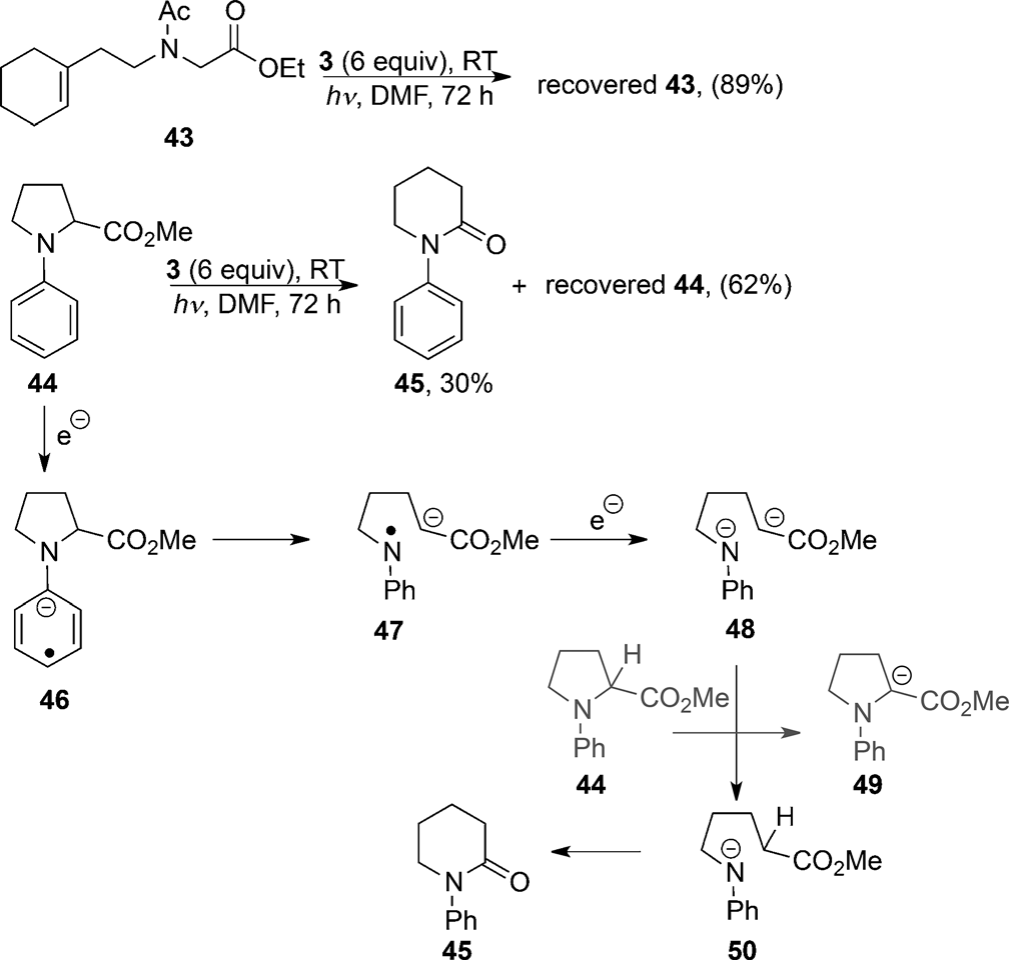
Substrates for ArN–C cleavage.

Finally, we prepared the modified ArN–C substrate **44** where cleavage of the ArN–C bond at the radical anion stage (**46**) would leave the radical and anion tethered together in **47** (Scheme 3). In this case, an intriguing rearrangement of the pyrrolidine into a piperidone product **45** (30 %) occurred. Efforts to improve the conversion by adding more equivalents of **3** were not successful, and this is consistent with the representation in Scheme 3. The initial radical anion **46** undergoes fragmentation to **47**. In the presence of excess **3**, further reduction to the amidyl anion **48** should occur rapidly. The dianion **48** is unlikely to cyclize, but cyclization could occur after proton transfer from another molecule of **44**, thereby forming the enolate **49** which will not undergo any reduction. Finally, cyclization of the anion **50** would afford the piperidone **45**. If this proposal is correct, it would also be relevant for the closest analogue of **44**, that is, **41 a**. The lower yield in these two substrates could therefore be explained both by this proton transfer from substrate and by the inherent difficulty of electron transfer to an N,N-dialkylaniline.

To conclude, electron transfer from the photoactivated neutral electron donor **3** delivers high yields of S–N and C–N cleavage products for a range of nitrogen-containing species including anilines, sulfonamides, and amides. These reactions proceed at room temperature and under mild reaction conditions in the absence of any metal reagents, thus illustrating challenging reactions which can be achieved by photoactivated neutral organic electron donors.

## Supporting Information

As a service to our authors and readers, this journal provides supporting information supplied by the authors. Such materials are peer reviewed and may be re-organized for online delivery, but are not copy-edited or typeset. Technical support issues arising from supporting information (other than missing files) should be addressed to the authors.

miscellaneous_information
