# Supplementary material for: Metal-Free Reductive Cleavage of C–N and S–N Bonds by Photoactivated Electron Transfer from a Neutral Organic Donor
Source: Angew Chem Int Ed Engl. 2013 Dec 6;53(2):474–8. doi: 10.1002/anie.201306543 (PMC4227565; doi:10.1002/anie.201306543)
Supplement: Supplementary file 1 — miscellaneous_information [file anie0053-0474-SD1.pdf]

Angewandte Chemie
Eine Zeitschrift der Gesellschaft Deutscher Chemiker

Supporting Information
© Wiley-VCH 2013
69451 Weinheim, Germany

Metal-Free Reductive Cleavage of C–N and S–N Bonds by Photoactivated Electron Transfer from a Neutral Organic Donor**

Steven O'Sullivan, Eswararao Doni, Tell Tuttle, and John A. Murphy**

anie_201306543_sm_miscellaneous_information.pdf

Supplementary Information:

Experimental Results________________S2

Details of Computational Study________S39

Spectra ___________________________S58

References_________________________S119

General

All chemicals and reagents were obtained from commercial suppliers and reactions were carried out under anhydrous conditions unless otherwise stated.

Acetonitrile was distilled over phosphorus pentoxide. Hexane, CH_2Cl_2, Et_2O and THF were all kept anhydrous by purification by an Innovative Technology inc. PURESOLV SPS-400-5 solvent purification apparatus. Solvents were deoxygenated by bubbling dry argon through with stirring for 1 hour.

Ultra-violet experiments were carried out using two Blak Ray® B-100 Series high intensity inspection lamps, with 100 W, 365 nm spot bulbs.

Chromatography was carried out on silica gel 60 (200-400 mesh). TLC analysis was carried out on aluminium sheets of silica gel 60 F_{245} and developed in acidic ethanolic vanillin or a phosphomolybdic acid solution.

^{1}H NMR and ^{13}C NMR analyses were carried out on a Bruker Avance AV3 400 spectrometer, operating at 400 and 100 MHz respectively or on a Bruker DRX500 spectrometer operating at 500 and 125 MHz respectively. $CDCl_3$, DMSO - d_6 or benzene - d_6, was taken as the solvent. Chemical shifts are reported in parts per million shift (δ value) calibrated against the residual solvent peak. Signal patterns are indicated as s, singlet; br. s, broad singlet; d, doublet; dd, double doublet; t, triplet; dt, double triplet; q, quartet; m, multiplet. Coupling constants (J) are given in hertz (Hz).

Infra-red analyses were carried out on a SHIMADZU IRAffinity-1 FTIR Spectrophotometer (ATR).

High Resolution Mass spectral analyses were carried out at EPSRC National Mass Spectrometry Service Centre in Swansea on a LTQ Orbitrap XL using Atmospheric Pressure Chemical Ionisation (APCI) or High Resolution Nano-Electrospray (HNESP), and masses observed are accurate to within 5 ppm.

Low resolution mass spectral analyses were carried out on a Thermofinnigan LCQ DUO LDU 00377 Mass Spectrometer operating electrospray ionisation (ESI). GC/CI was carried out using Agilent Technologies 7890A GC System, 5975C Inert XL EI/CI MSD with Triple Axis Detector with an Agilent DB5-MS 30 m x 0.25 mm column with 0.25 μ packing. The carrier gas was helium at 1 mL/min and the reagent gas was methane. GC/EI was carried out using ThermoFinnigan PolarisQ Ion Trap Spectrometer with an Agilent DB5-MS 30 m x 0.25 mm column with 0.25 μ packing. The carrier gas was helium at 1 mL/min. MALDI MS was carried out on a Shimadzu Axima-CFR system with no matrix.

Melting points were measured on a Gallenkamp Griffin SG94/05/530 Melting Point Apparatus and are unamended.

Synthesis of 1,3-bis(*N'*,*N'*-dimethyl-4-aminopyridinium)propane dibromide

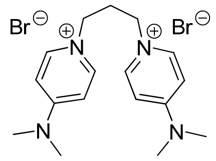

A solution of 4-(dimethylamino)pyridine (9.16 g, 75 mmol, 2.5 equiv.) and 1,3-dibromopropane (6.06 g, 30 mmol, 1.0 equiv.) in a flask containing acetonitrile (60 mL) was stirred at reflux for 16 h, under argon. Diethyl ether (10 mL) was added to the reaction flask and the product precipitated instantaneously, and was then filtered. To precipitate more of the solid, an additional amount of diethyl ether (20 mL) was added to the filtrate. After filtration, the solid was washed with diethyl ether (3 x 50 mL) and dried under vacuum to give 1,3-bis(*N'*,*N'*-dimethyl-4-aminopyridinium)propane dibromide[1] (13 g, 97%) as a white solid m.p. 199-203 °C (lit.[1] m.p. 199-203 °C); [Found: (ESI$^+$) (M-Br)$^+$ 365.1338. $C_{17}H_{26}BrN_4$ (M-Br) requires 365.1335]; ν_{max}(ATR)/cm^{-1} 3027, 2725, 2468, 1649, 1571, 1403; δ_H (400 MHz, DMSO-d$_6$) 2.36 (2H, quintet, J = 7.2 Hz, $CH_2CH_2CH_2$), 3.18 (12H, s, NCH_3), 4.28 (4H, t, J = 7.2 Hz, NCH_2), 7.04 (4H, d, J = 7.6 Hz, Ar*H*), 8.34 (4H, d, J = 7.6 Hz, Ar*H*); δ_C (100 MHz, DMSO-d$_6$) 31.2, 39.8, 53.7, 107.8, 141.8, 155.8.

Synthesis of *N*,*N*,*N'*,*N'*-tetramethyl-7,8-dihydro-6H-dipyrido[1,2-a;2',1'-c][1,4] diazepine-2,12-diamine 3

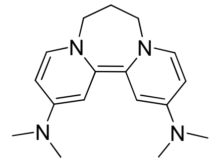

A mixture of 1,3-bis(*N'*,*N'*-dimethyl-4-aminopyridinium)propane dibromide (13.38 g, 30 mmol, 1.0 equiv.) and pre-washed NaH (~95%, stored in glovebox, 4.55 g, 180 mmol, 6 equiv.) were taken in a Schlenk flask mounted with a dry ice condenser, and the atmosphere was made oxygen-free by flushing with argon. At this point, ammonia (75 mL) was condensed, left at reflux for 4 h and was subsequently allowed to evaporate overnight (14 h) under a steady argon flow. The flask was then transferred to an oxygen-free, moisture-free glove box. The solid was extracted with dry diethyl ether (300 mL) and the solvent was then removed by distillation and under vacuum (10-20 mbar) to give the pure *N*,*N*,*N'*,*N'*-tetramethyl-7,8-dihydro-6*H*-dipyrido-[1,2-a;2',1'-c][1,4]-diazepine-2,12-diamine **3**[1] (7.61 g, 89%) as a purple-black, moisture-sensitive and highly oxygen-sensitive solid. δ_H (400 MHz, benzene-d$_6$) 1.00 (2H, quintet, J = 6.3 Hz, $CH_2CH_2CH_2$), 2.46 (12H, s, $N(CH_3)_2$), 3.03 (4H, t, J = 6.3 Hz, NCH_2), 4.91 (2H, dd, J = 7.5, 2.2 Hz, Ar*H*), 5.14 (2H, d, J = 2.2 Hz, Ar*H*), 5.64 (2H, d, J = 7.5

Hz, Ar*H*); δ_C (100 MHz, benzene-d$_6$) 24.5, 40.8, 52.6, 95.8, 96.2, 116.0, 138.7, 143.7. The ^{1}H-NMR and ^{13}C-NMR spectral data are consistent with the previously published data of **3**.[1]

General acid-base work-up procedure for the reduction of arenesulfonamides

The reaction mixture was quenched with 1N HCl (10 mL) and then extracted with diethyl ether (3 x 10 mL). The combined ether layers were washed with water (10 mL), brine solution (10 mL) and dried over anhydrous sodium sulfate. The filtered solution was concentrated to afford recovery of pure starting materials in yields as stated. The collected aqueous phase was then basified with 2N NaOH and extracted with diethyl ether (3 x 10 mL). The combined ether layers were washed with water (10 mL), brine solution (10 mL) and dried over anhydrous sodium sulfate. The filtered solution was concentrated to afford the corresponding pure reduced products in yields as stated.

Synthesis of 4-phenyl-1-tosylpiperidine 10[2]

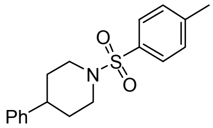

To a mixture of 4-phenylpiperidine **13** (0.483 g, 3 mmol) and pyridine (1 mL), *p*-toluenesulfonyl chloride (0.686 g, 3.6 mmol) in dry dichloromethane (10 mL) was added slowly and stirred at room temperature for 1 h. The reaction mixture was then washed with aqueous 1N HCl (10 mL) and extracted with diethyl ether (2 x 10 mL). The combined organic phases were washed with water (10 mL), brine solution (10 mL) and dried over anhydrous sodium sulfate. The filtered solution was concentrated and was purified by column chromatography [5% ethyl acetate in petroleum ether] to yield 4-phenyl-1-tosylpiperidine **10**[2] (0.714 g, 75%) as a white powder m.p. 150-152 °C (lit.[2] 151-152 °C); [Found: (CI corona$^+$) (M+H)$^+$ 316.1368. $C_{18}H_{22}NO_2S$ (M+H) requires 316.1366]; ν_{max}(ATR)/cm^{-1} 3025, 2943, 2922, 2840, 1595, 1493, 1450, 1341, 1164; δ_H (400 MHz, CDCl$_3$) 1.84-1.92 (4H, m, ArCH(C*H*$_2$)$_2$), 2.34-2.45 (3H, m, ArC*H*, NC*H*$_2$), 2.48 (3H, s, ArC*H*$_3$), 3.94-3.98 (2H, m, NC*H*$_2$), 7.16 (2H, d, J = 8.8 Hz, ArH), 7.21-7.25 (1H, m, ArH), 7.28-7.34 (2H, m, ArH), 7.37 (2H, d, J = 8.4 Hz, ArH), 7.71 (2H, d, J = 8.4 Hz, ArH); δ_C (100 MHz, CDCl$_3$) 21.0, 32.1, 41.3, 46.4, 126.1, 126.2, 127.3, 128.1, 129.1, 132.7, 143.0, 144.4; *m/z* (ESI$^+$) 316 ([M+H]$^+$, 100%), 247 (6).

UV-activated reduction of 4-phenyl-1-tosylpiperidine 10

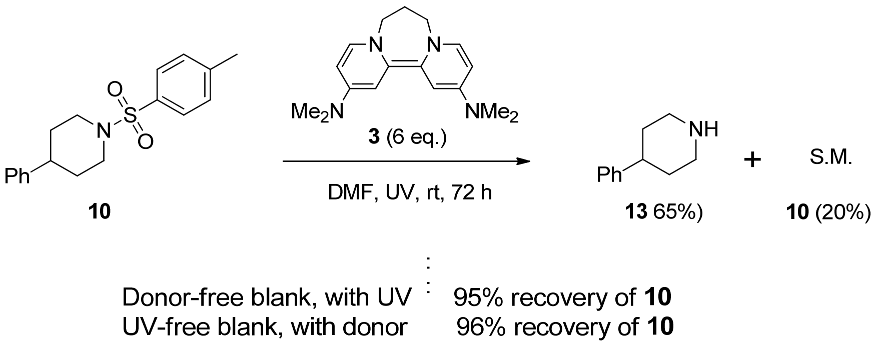

The general procedure for electron transfer reactions under UV conditions was applied to 4-phenyl-1-tosylpiperidine **10** (94.0 mg, 0.3 mmol, 1 equiv.) using the donor **3** (341 mg, 1.8 mmol, 6 equiv.) for 72 h at room temperature. After following the general acid-base work-up procedure for the reduction of arenesulfonamides, pure starting material **10** (19 mg, 20%) and 4-phenylpiperidine **13**[3] (31 mg, 65%) were obtained as white solids. For **13**: m.p. 62-64 °C (lit.[3] 62-64 °C); [Found: (CI corona$^+$) (M+H)$^+$ 162.1277. $C_{11}H_{16}N$ (M+H) requires 162.1277]; ν_{max}(ATR)/cm^{-1} 3293, 2931, 2917, 2848, 1644, 1543, 1451, 1413, 1370, 1247; δ_H (400 MHz, CDCl$_3$) 1.69-1.73 (2H, m, ArCHC*H$_2$*), 1.80 (1H, bs, N*H*), 1.84-1.88 (2H, m, ArCHC*H$_2$*), 2.61-2.67 (1H, m, ArC*H*), 2.74-2.81 (2H, m, NHC*H$_2$*), 3.20-3.23 (2H, m, NHC*H$_2$*), 7.20-7.35 (5H, m, ArH); δ_C (100 MHz, CDCl$_3$) 34.1, 42.6, 46.7, 125.6, 126.3, 127.9, 146.3. The spectral data were consistent with the literature data of the same compound.[3]

Donor-free blank and UV-free blank reactions were performed as per the general procedure and provided the starting material **10** (0.089 g, 95%) and (0.090 g, 96%), respectively.

Synthesis of 4-methyl-*N,N*-dioctylbenzenesulfonamide 14[4]

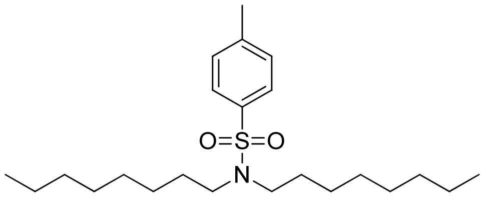

To a mixture of di-*N*-octylamine **15** (0.482 g, 2 mmol) and pyridine (0.5 mL), *p*-toluenesulfonyl chloride (0.46 g, 2.4 mmol) in dry dichloromethane (5mL) was added slowly and stirred at room temperature for 1 h. The reaction mixture was then washed with aqueous 1N HCl (10 mL) and extracted with diethyl ether (2 x 10 mL). The combined organic phases were washed with water (10 mL), brine solution (10 mL) and dried over anhydrous sodium sulfate. The filtered solution was concentrated and was purified by column chromatography [4% ethyl acetate in petroleum ether] to yield 4-methyl-*N,N*-dioctylbenzenesulfonamide **14** as a colourless oil (0.556 g, 70%). [Found: (ESI$^+$) (M+H)$^+$ 396.2931. $C_{23}H_{42}NO_2S$ (M+H) requires 396.2931]; ν_{max}(film)/cm^{-1} 3029, 2927, 2856, 1599, 1466, 1376, 1341, 1159; δ_H (400 MHz, CDCl$_3$) 0.88 (6H, t, J = 7.2 Hz, CH$_2$CH$_2$C*H$_3$*), 1.25-1.30 (20H,

m, $NCH_2CH_2(CH_2)_5CH_3$), 1.48-1.52 (4H, m, $NCH_2CH_2CH_2$), 2.42 (3H, s, $ArCH_3$), 3.09 (4H, t, J = 7.6 Hz, $NCH_2CH_2CH_2$), 7.28 (2H, d, J = 8.0 Hz, ArH), 7.68 (2H, d, J = 8.0 Hz, ArH); δ_C (100 MHz, $CDCl_3$) 14.1, 21.4, 22.6, 26.7, 28.7, 29.2, 31.8, 48.2, 127.1, 129.5, 137.2, 142.8; *m/z* (ESI^+) 808 [$(2M+NH_4)^+$, 100%], 396 [$(M+H)^+$, 85%].

UV-activated reduction of 4-methyl-*N*,*N*-dioctylbenzenesulfonamide 14

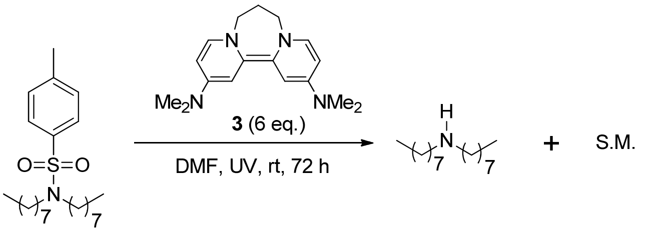

14 15 (59%) 14 (28%)

Donor-free blank, with UV 95% recovery of **14**
UV-free blank, with donor 97% recovery of **14**
Donor **3** (4 equiv.), 96h **15** (52%), **14** (33%)

The general procedure for electron transfer reactions under UV conditions was applied to 4-methyl-*N*,*N*-dioctylbenzenesulfonamide **14** (118.6 mg, 0.3 mmol, 1 equiv.) using the donor **3** (511 mg, 1.8 mmol, 6 equiv.) for 72 h at room temperature. After following the general acid-base work-up procedure for the reduction of arenesulfonamides, pure starting material **14** (32.9 mg, 28%) and di-*N*-octylamine **15**[5] (42.8 mg, 59%) were obtained as colourless oils. For **15**: [Found: (ESI^+) $(M+H)^+$ 242.2841. $C_{16}H_{36}N$ (M+H) requires 242.2842]; ν_{max}(film)/cm^{-1} 3274, 3116, 2955, 2924, 2853, 1468, 720; δ_H (400 MHz, $CDCl_3$) 0.87 (6H, t, J = 7.6 Hz, $N(CH_2)_7CH_3$), 1.05 (1H, bs, NH), 1.26-133 (20H, m, $N(CH_2)_2(CH_2)_5CH_3$), 1.44-1.49 (4H, m, $NCH_2CH_2(CH_2)_5CH_3$), 2.58 (4H, t, J = 7.2 Hz, $NCH_2(CH_2)_7CH_3$); δ_C (100 MHz, $CDCl_3$) 14.1, 22.7, 27.4, 29.3, 29.6, 30.2, 31.8, 50.2; *m/z* (ESI^+) 242 [$(M+H)^+$, 100%].

Donor-free and UV-free blank reactions were performed as per the general procedure and provided the starting material **14** (0.113 g, 95%) and (0.1152 g, 97%), respectively.

Synthesis of 2-tosyl-1,2,3,4-tetrahydroisoquinoline 16[6]

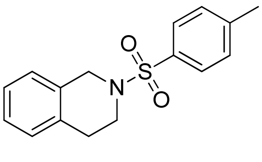

To a mixture of 1,2,3,4-tetrahydroisoquinoline **17** (0.2663 g, 2 mmol) and pyridine (0.5 mL), *p*-toluenesulfonyl chloride (0.46 g, 2.4 mmol) in dry dichloromethane (5 mL) was added slowly and stirred at room temperature for 1 h. The reaction mixture was then washed with aqueous 1N HCl (10 mL) and extracted with diethyl ether (2 x 10 mL). The combined organic phases were washed with water (10 mL), brine solution (10 mL) and dried over anhydrous sodium sulfate. The filtered solution was concentrated and was purified by column chromatography [5% ethyl acetate in petroleum ether] to yield 2-tosyl-1,2,3,4-tetrahydroisoquinoline **16** (0.505 g, 88%) as a white solid m.p. 145-146 °C (lit.:[7] 147 °C); [Found: (ESI^+) $(M+H)^+$ 288.1052. $C_{16}H_{18}NO_2S$ (M+H) requires 288.1053]; ν_{max}(film)/cm^{-1} 3023, 2935, 2861, 1594, 1455, 1352, 1339, 1165, 1136; δ_H (400 MHz, $CDCl_3$) 2.43 (3H, s, ArC*H*$_3$), 2.94 (2H, t, *J* = 5.6 Hz, ArC*H*$_2$CH$_2$N), 3.37 (2H, t, *J* = 5.6 Hz, ArCH$_2$C*H*$_2$N), 4.26 (2H, s, ArC*H*$_2$N), 7.03-7.16 (4H, m, ArH), 7.33 (2H, d, *J* = 7.6 Hz, ArH), 7.74 (2H, d, *J* = 8.0 Hz, ArH); δ_C (100 MHz, $CDCl_3$) 21.5, 28.9, 43.7, 47.5, 126.3, 126.4, 126.7, 127.8, 128.8, 129.7, 131.7, 133.1, 133.3, 143.7; *m/z* (ESI^+) 305 [$(M+NH_4)^+$, 7%], 288 [$(M+H)^+$, 100%].

UV-activated reduction of 2-tosyl-1,2,3,4-tetrahydroisoquinoline 16

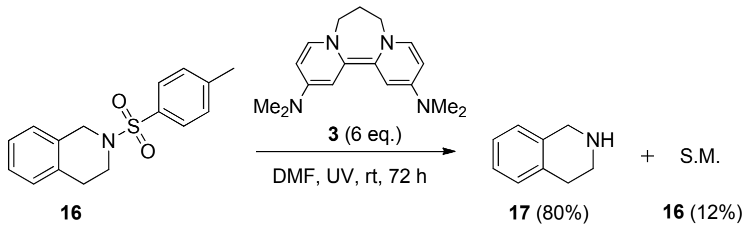

Donor-free blank, with UV	92% recovery of **16**
UV-free blank, with donor	93% recovery of **16**
Donor **3** (4 equiv.), 72 h	**17** (59%), **16** (18%)

The general procedure for electron transfer reactions under UV conditions was applied to 2-tosyl-1,2,3,4-tetrahydroisoquinoline **16** (86.2 mg, 0.3 mmol, 1 equiv.) using the donor **3** (511 mg, 1.8 mmol, 6 equiv.) for 72 h at room temperature. After following the general acid-base work-up procedure for the reduction of arenesulfonamides, pure starting material **16** (10.6 mg, 12%), as a white solid, and 1,2,3,4-tetrahydroisoquinoline **17**[8] (32.0 mg, 80%), as a yellow liquid, were obtained. For **17**: [Found: (EI^+) $(M-H)^+$ 132.0808. $C_9H_{10}N$ (M-H) requires 132.0808]; ν_{max}(film)/cm^{-1} 3290, 3019, 2923, 2834, 1668, 1581, 1495, 744; δ_H (400 MHz, $CDCl_3$) 1.93 (1H, bs, NH), 2.81 (2H, t, *J* = 6.0 Hz, ArC*H*$_2$CH$_2$N), 3.15 (2H, t, *J* = 6.0 Hz, ArCH$_2$C*H*$_2$N), 4.03 (2H, s, ArC*H*$_2$N), 7.00-7.03 (1H, m, ArH), 7.08-7.15 (3H, m, ArH); δ_C (100 MHz, $CDCl_3$) 29.2, 43.9, 48.3, 125.7, 126.0, 126.2, 129.3, 134.8, 136.0. The spectral data were consistent with the literature data.[8]

Donor-free and UV-free blank reactions were performed as per the general procedure and provided the starting material **16** (0.0791 g, 92%) and (0.0803 g, 93%), respectively.

Synthesis of 1-tosyl-1H-indole 6[9]

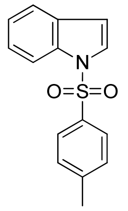

p-Toluenesulfonyl chloride (0.46 g, 2.4 mmol) in dry dichloromethane (5mL) was added slowly to a mixture of indole **7** (0.234 g, 2 mmol) and pyridine (0.5 mL), and stirred at room temperature for 1 h. The reaction mixture was then washed with aqueous 1N HCl (10 mL) and extracted with diethyl ether (2 x 10 mL). The combined organic phases were washed with water (10 mL), brine solution (10 mL) and dried over anhydrous sodium sulfate. The filtered solution was concentrated and purified by column chromatography (10% diethyl ether in petroleum ether) to yield 1-tosyl-1H-indole **6** (0.522 g, 91%) as a white solid m.p. 82-84 °C (lit.:[9] 83-84 °C); [Found: (ESI^+) $(M+H)^+$ 272.0739. $C_{15}H_{14}NO_2S$ (M+H) requires 272.0740]; ν_{max}(KBr)/cm^{-1} 3116, 2919, 1596, 1446, 1369, 1260, 1168, 1128; δ_H (400 MHz, $CDCl_3$) 2.34 (3H, s, C*H3*), 6.67 (1H, dd, *J* = 3.6, 0.8 Hz, ArH), 7.21-7.26 (3H, m, ArH), 7.30-7.35 (1H, m, ArH), 7.53-7.59 (2H, m, ArH), 7.76-7.79 (2H, m, ArH), 8.00-8.02 (1H, m, ArH); δ_C (100 MHz, $CDCl_3$) 21.6, 109.0, 113.6, 121.4, 123.3, 124.6, 126.3, 126.8, 129.9, 130.7, 134.8, 135.3, 144.9; *m/z* (ESI^+) 289 ($[M+NH_4]^+$, 29%), 272 ($[M+H]^+$, 100%).

UV-activated reduction of 1-tosyl-1H-indole 6

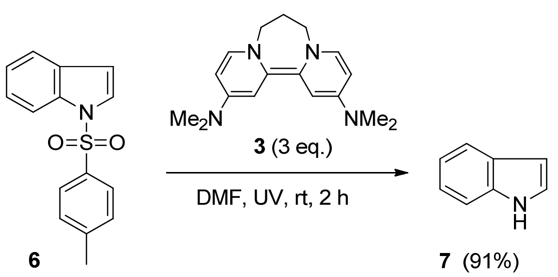

6 **7** (91%)

Donor-free blank, with UV : **7** (4%) from ^{1}H-NMR of crude product
UV-free blank, with donor : **7** (31%)

The general procedure for electron transfer reactions under UV conditions was applied to 1-tosyl-1H-indole **6** (81.3 mg, 0.3 mmol, 1 equiv.) using the donor **3** (255 mg, 0.9 mmol, 3 equiv.) for 2 h at room temperature. After following the general acid-base work-up procedure for the reduction of arenesulfonamides, only indole **7**[10] (31.8 mg, 91%) was obtained as a white solid m.p. 51-53 °C; (lit.:[10] 51-53 °C); ν_{max}(KBr) /cm^{-1} 3400, 3098, 3049, 1576, 1506, 1456, 1247, 745; δ_H (400 MHz, $CDCl_3$) 6.59-6.60 (1H, m, ArH), 7.14-7.18 (1H, m, ArH), 7.21-7.27 (2H, m, ArH), 7.42 (1H, dd, *J* = 8.0, 0.4 Hz ArH), 7.92 (1H, d, *J* = 8.0 Hz, ArH), 8.11 (1H, bs, NH); δ_C (100 MHz, $CDCl_3$) δ 102.6, 111.0, 119.8, 120.8, 122.0, 124.1, 127.9, 135.8. The spectral data were consistent with the literature data.[10]

(a) Donor-free and (b) UV-free blank reactions were performed as per the general procedure and provided the product **7** in 4% conversion (from ^{1}H-NMR of the crude product) and (0.011 g, 31%), respectively.

Synthesis of *N*-benzyl-4-methyl-*N*-phenylbenzenesulfonamide 8[11]

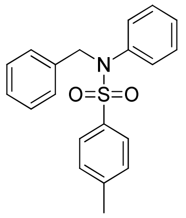

To a mixture of *N*-benzylaniline **9** (0.916 g, 5 mmol) and pyridine (2 mL), *p*-toluenesulfonyl chloride (1.1439 g, 6 mmol) in dry dichloromethane (10 mL) was added slowly and stirred at room temperature for 90 min. The reaction mixture was then washed with aqueous 1N HCl (10 mL) and extracted with diethyl ether (2 x 10 mL). The combined organic phases were washed with water (10 mL), brine solution (10 mL) and dried over anhydrous sodium sulfate. The filtered solution was concentrated and purified by column chromatography [30% ethyl acetate in petroleum ether] to yield *N*-benzyl-4-methyl-*N*-phenylbenzenesulfonamide **8** (1.616 g, 96%) as a white solid m.p. 138-140 °C (lit.:[11] 139-140 °C); [Found: (ESI$^+$) (M+H)$^+$ 338.1204. $C_{20}H_{20}NO_2S$ (M+H) requires 338.1209]; ν_{max}(film)/cm^{-1} 3027, 2919, 1597, 1492, 1347, 1153, 1095; δ_H (400 MHz, $CDCl_3$) 2.46 (3H, s, ArC*H$_3$*), 4.73 (2H, s, ArC*H$_2$*NAr), 6.97-7.00 (2H, m, ArH), 7.20-7.23 (8H, m, ArH), 7.27-7.30 (2H, m, ArH), 7.54-7.56 (2H, m, ArH); δ_C (100 MHz, $CDCl_3$) 21.6, 54.7, 127.7, 127.9, 127.9, 128.5, 128.6, 129.0, 129.1, 129.6, 135.7, 136.0, 139.0, 143.5; *m/z* (ESI$^+$) 355 [(M+NH$_4$)$^+$, 55%], 338 [(M+H)$^+$, 100%], 183 (3).

UV-activated reduction of *N*-benzyl-*N*-phenyl-4-methylbenzenesulfonamide 8

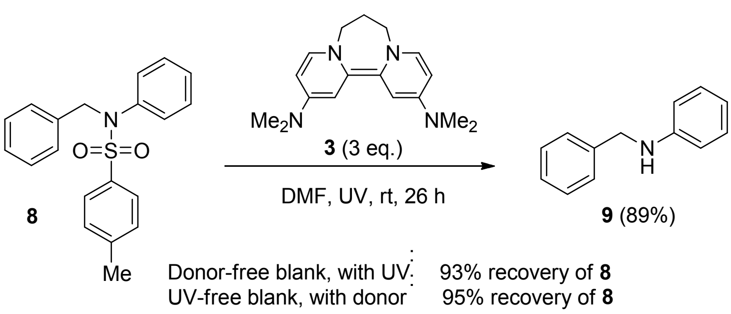

The general procedure for electron transfer reactions under UV conditions was applied to *N*-benzyl-*N*-phenyl-4-methylbenzenesulfonamide **8** (101 mg, 0.3 mmol, 1 equiv.) using the donor **3** (255 mg, 0.9 mmol, 3 equiv.) for 26 h at room temperature. After following the general acid-base work-up procedure for the reduction of arenesulfonamides, only *N*-benzylaniline **9**[12] (48 mg, 89%) was

obtained as a white solid m.p. 34-35 °C (lit.:[12] 33-34 °C); [Found: (ESI$^+$) (M+H)$^+$ 184.1122. $C_{13}H_{14}N$ (M+H) requires 184.1121]; ν_{max}(KBr) /cm^{-1} 3417, 3022, 2924, 2846, 1603, 1512, 1329, 736; δ_H (400 MHz, $CDCl_3$) 4.25 (1H, bs, NH), 4.35 (2H, s, ArCH_2N), 6.66-6.68 (2H, m, ArH), 6.72-6.76 (1H, m, ArH), 7.17-7.21 (2H, m, ArH), 7.27-7.31 (1H, m, ArH), 7.34-7.41 (4H, m, ArH); δ_C (100 MHz, $CDCl_3$) 48.4, 112.9, 117.6, 127.3, 127.6, 128.7, 129.3, 139.5, 148.2; *m/z* (ESI$^+$) 184 [(M+H)$^+$, 100%].

Donor-free and UV-free blank reactions were performed as per the general procedure and provided the starting material **8** (0.0942 g, 93%) and (0.096 g, 95%), respectively.

Synthesis of *N*-cyclopropyl-4-methylbenzenesulfonamide[13]

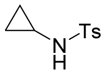

To a solution of cyclopropylamine (1.14 g, 20 mmol, 1 equiv) and pyridine (1.90 g, 24 mmol, 1.2 equiv.) in water (20 mL) was added toluenesulfonyl chloride (4.19 g, 22 mmol, 1.1 equiv.) portion-wise with stirring. The reaction mixture was then stirred at room temperature for 1 h before filtering and rinsing the precipitate with additional 10 mL water. The yellow solid was then dissolved in ether, dried over sodium sulfate and concentrated in vacuo to yield an off-white solid. Recrystallization from ethyl acetate/hexane gave rise to *N*-cyclopropyl-4-methylbenzenesulfonamide as a white crystalline solid (3.72 g, 88%); m.p. 72-74 °C [lit.[13] 74-76 °C]; ν_{max} (ATR)/cm^{-1} 3271, 2993, 1599, 1409, 1362, 1315, 1155, 1093, 881, 816; δ_H (400 MHz, $CDCl_3$) 0.59-0.63 (4H, m, cyclopropyl-*H*), 2.23-2.29 (1H, m, NC*H*), 2.46 (3H, s, ArCH_3), 4.95 (1H, s, N*H*), 7.34 (s, J = 8.4 Hz, Ar*H*), 7.81 (s, J = 8.4 Hz, Ar*H*); δ_C (100 MHz, $CDCl_3$) 5.6, 21.0, 23.8, 127.0, 129.1, 136.2, 143.0; *m/z* (GCMS, CI$^+$, CH_4) 252.0([M+C_3H_7]$^+$, 5%), 212.0 ([M+H]$^+$, 100), 154.9 (78).

Synthesis of *N*-cyclopropyl-*N*-tetradecyl-4-methylbenzenesulfonamide 24

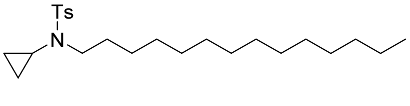

To sodium hydride (as a 60% suspension in mineral oil, 440 mg, 11 mmol, 1.1 equiv.) dispersed in dry THF (20 mL) was added a solution of *N*-cyclopropyl-4-methylbenzenesulfonamide (2.32 g, 11 mmol, 1.1 equiv.) in dry THF (15 mL) under argon. A dense slurry formed which did not disperse upon addition of more dry THF (20 mL) so dry DMF (10 mL) was added and stirred for 1 h before addition of 1-bromotetradecane (2.44 ml, 10 mmol, 1 equiv.) then stirring overnight at reflux. The reaction mixture was then allowed to return to room temperature and quenched with HCl (aqueous, 2N, 50 mL) and diethyl ether (150 mL) then extracted with further ether (2 x 50 mL). This solution was then sequentially washed with HCl (aqueous, 2N, 50 mL), water (50 mL), brine (25 mL), dried over sodium sulfate, filtered and evaporated under reduced pressure. The resulting oil was purified by

column chromatography in 10 % diethyl ether/petroleum ether to yield *N-cyclopropyl-N-tetradecyl-4-methylbenzenesulfonamide* **24** as a white solid (3.62 g, 89%); m.p. 35-37 °C; ν_{max} (ATR)/cm^{-1} 2922, 2852, 1456, 1344, 1161, 1091, 1026, 814, 713; δ_H (400 MHz, $CDCl_3$) 0.66-0.73 (2H, m, cyclopropyl-*H*), 0.84-0.88 (2H, m, cyclopropyl-*H*), 0.90 (3H, t, *J* = 6.8 Hz, CH_2CH_3) 1.24-1.38 (22H, m, $(CH_2)_{11}$), 1.57-1.64 (2H, m, NCH_2CH_2), 2.00-2.06 (1H, m, NC*H*), 2.45 (3H, s, ArCH_3), 3.15-3.19 (2H, m, NCH_2), 7.32 (s, *J* = 8.1 Hz, Ar*H*), 7.76 (s, *J* = 8.1 Hz, Ar*H*); δ_C (100 MHz, $CDCl_3$) 6.7, 13.5, 20.8, 22.1, 26.4, 27.9, 28.7, 28.8, 29.0, 29.0, 29.1, 29.2, 29.8, 31.4, 50.7, 127.0, 128.9, 135.3, 142.5; *m*/*z* (GCMS, CI^+, CH_4) 436.2 ($[M+C2H_5]^+$, 15%), 408.2 ($[M+H]^+$, 100), 252.2 (18).

Reaction of *N*-cyclopropyl-4-methyl-*N*-tetradecylbenzenesulfonamide 24 with photoactivated donor 3

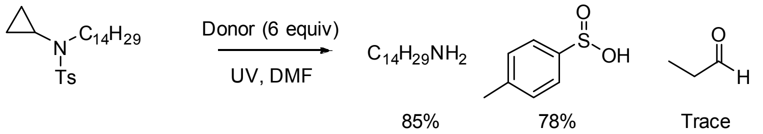

N-cyclopropyl-4-methyl-*N*-tetradecylbenzenesulfonamide **24** (105 mg, 0.3 mmol, 1 equiv.) was reacted under standard conditions with photoactivated donor **3** (512 mg, 1.8 mmol, 6 equiv.). After 72 h reaction time the reaction mixture was poured into HCl (2N, aq., 25 mL) and diethyl ether (25 mL), The aqueous fraction was washed with an additional 2 x 25 mL ether and the combined organic fractions rinsed with HCl (2N, aq., 25 mL), water (25 mL) and brine, then dried over sodium sulfate and concentrated under reduced pressure to yield 4-methylbenzenesulfinic acid, *N*-cyclopropyl-4-methyl-*N*-tetradecylbenzenesulfonamide **24** and propionaldehyde **32** (trace).

The original acidic aqueous fractions from above were basified to pH 10 with sodium hydroxide then extracted with ether (3 x 25 mL). The combined organic fractions were then rinsed with water (25 mL) and brine (2 x 25 mL), dried (sodium sulfate) and concentrated in vacuo to afford tetradecylamine **26** as an off-white solid (58 mg, 85%); m.p. 36-38 °C [lit.[14] 40 °C]; ν_{max} (ATR)/cm^{-1} 3334, 2955, 2918, 2851, 1570, 1487, 1468, 1262, 1096, 1022, 802, 719; δ_H (400 MHz, $CDCl_3$) 0.90 (3H, t , *J* = Hz, $(CH_2)_{13}CH_3$), 1.24-1.39 (24H, m, $NCH_2(CH_2)_{12}$), 1.43-1.51 (2H, m, NCH_2), 2.78-2.91 (2H, bs, NH_2); δ_C (100 MHz, $CDCl_3$) 13.6, 22.2, 28.9, 28.9, 29.1, 29.2, 29.2, 31.4; *m*/*z* (GCMS, CI^+, CH_4) 242.2 ($[M+C_2H_5]^+$, 24%), 214.1 ($[M+H]^+$, 97), 212.1 (100).

Basification followed by re-extraction of the mixture of 4-methylbenzenesulfinic acid **25** and *N*-cyclopropyl-4-methyl-*N*-tetradecylbenzenesulfonamide **24** yielded the two products (36 mg, 78%) and (6 mg, 9%) respectively with data as reported above.

Propionaldehyde was tentatively detected in low concentration by a signature aldehyde peak in ^1H NMR (9.77, t, *J* = 1.7 Hz). See below.

General Procedure A– Methanesulfonylation of amines:

The amine (1 equiv.) was dissolved in dry dichloromethane (20 mL) and triethylamine (4 equiv.) added with stirring. Methanesulfonyl chloride (2 equiv.) was added dropwise to the stirred solution under a flow of argon then left stirring at room temperature overnight. Aqueous 2N NaOH (50 mL) was then added, and extracted with dichloromethane (3 x 25 mL). The aqueous fraction was acidified with aqueous HCl (37%) then washed with further portions of DCM (4 x 25 mL). The organic fractions were rinsed with water (3 x 20 mL), dried over Na_2SO_4 and the solvent evaporated. The crude organic residue was then filtered through celite (DCM), to afford the pure corresponding methanesulfonamide.

***N*-Benzylmethanesulfonamide 34e**[15]

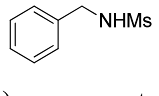

Benzylamine (5.36 g, 50 mmol, 1 equiv.) was reacted according to procedure A to afford *N*-benzylmethanesulfonamide **34e**[15] as a white solid (7.87 g, 87%); mp 61-63 °C [lit.[15] 63-64 °C]; ν_{max} (ATR)/cm^{-1} 3232, 3014, 1461, 1438, 1320, 1134, 1062, 972, 864, 769; δ_H (400 MHz, $CDCl_3$) 2.90 (3H, s, SO_2CH_3), 4.35 (2H, d, *J* = 6.0 Hz, ArCH_2N), 4.67 (1H, br. s, N*H*), 7.33-7.42 (5H, m, Ar*H*); δ_C (100 MHz, $CDCl_3$) 40.7, 46.7, 127.4, 127.7, 128.5, 136.1; *m/z* (ESI$^+$) 184.00 ($[M-H]^+$, 100%).

N*-(3,5-dimethoxybenzyl)methanesulfonamide 34e

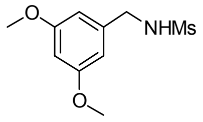

(3,5-Dimethoxyphenyl)methanamine (16.72 g, 100 mmol, 1 equiv.) was reacted according to procedure A to afford *N-(3,5-dimethoxybenzyl)methanesulfonamide* **34e*** as a white solid (8.50 g, 69%). M.pt. 77-79 °C; [Found: (HNESP$^+$) $(M+NH_4)^+$, 263.1063. $C_{10}H_{19}N_2O_4S$ $(M+NH_4)$, requires 263.1060; $(M+H)^+$, 246.0797. $C_{10}H_{16}NO_4S$ (M+H) requires 246.0795]; ν_{max} (ATR)/cm^{-1} 2935, 2846, 1595, 1459, 1321, 1205, 1146, 1064, 924, 791, 737; δ_H (500 MHz, $CDCl_3$) 2.92 (3H, s, SO_2CH_3), 3.80 (6H, s, ArOCH_3), 4.27 (2H, d, *J* = 6 Hz, ArCH_2N), 4.50-4.58 (1H, bs, N*H*), 6.41 (1H, t, *J* = 2.5 Hz, Ar*H*), 6.49 (2H, d, *J* = 2.5 Hz, Ar*H*); δ_C (125 MHz, $CDCl_3$) 41.2, 47.3, 55.4, 99.9, 105.7, 139.0, 161.3.

***N*-butylmethanesulfonamide 34h**[16]

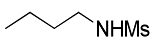

Butan-1-amine (7.31 g, 100 mmol, 1equiv.) was reacted according to procedure A to afford *N*-butylmethanesulfonamide **34h** [16] as a colourless oil (6.56 g, 42%); ν_{max} (ATR)/cm^{-1} 3504, 3202, 3094, 2958, 2874, 1565, 1461, 1308, 1164, 1052, 786; δ_H (500 MHz, $CDCl_3$) 0.95 (3H, t, *J* = 7.4 Hz, CH_2CH_3), 1.36-1.44 (2H, m, $CH_2CH_2CH_3$), 1.54-1.60 (2H, m, $NCH_2CH_2CH_2CH_3$), 2.92 (3H, s,

SO_2CH_3), 3.15 (2H, dt, *J* = 7.0, 6.8 Hz , NCH_2CH_2), 4.22 (1H, s, N*H*); δ_C (125 MHz, $CDCl_3$) 13.6, 19.7, 32.1, 40.1, 43.0; *m*/*z* (ESI^-) 149.9 ($[M-H]^-$, 100%).

N-cyclohexylmethanesulfonamide 34i[17]

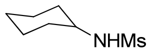

Cyclohexylamine (9.92 g, 100 mmol, 1 equiv.) was reacted according to procedure A to afford *N*-cyclohexylmethanesulfonamide **34i** as an off-white solid (16.2 g, 91%); m.p. 101-103 ^oC [lit.[17] 103 ^oC]; ν_{max} (ATR)/cm^{-1} 3260, 2924, 2855, 1446, 1305, 1149, 1082, 1002, 881, 756; δ_H (400 MHz, $CDCl_3$) 1.15-1.42 (6H, m, $NCHCH_2CH_2CH_2CH_2$), 1.60-1.65 (1H, m, NC*H*CH_2), 1.73-1.79 (2H, m, $NCHCH_2$), 2.00-2.04 (2H, m, $NCHCH_2$) 3.30 (3H, s, SO_2CH_3), 3.32-3.38 (1H, m, NC*H*), 4.09-4.18 (1H, bs, N*H*); δ_C (100 MHz, $CDCl_3$) 24.3, 24.6, 33.1, 41.5, 52.3; *m*/*z* ($APCI^+$) 195.1 ($[M+NH_4]^+$, 100%).

N-allylmethanesulfonamide 37[18]

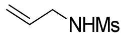

Allylamine (2.85 g, 50 mmol. 1 equiv.) was reacted according to procedure A to afford *N*-allylmethanesulfonamide **37**[18] as a yellow oil (4.71 g, 70%); ν_{max} (ATR)/cm^{-1} 3506, 3277, 3096, 1625, 1438, 1308, 1147, 1054, 970, 926, 836, 748; δ_H (500 MHz, $CDCl_3$) 2.98 (3H, s, SO_2CH_3), 3.78-3.81 (2H, m, NCH_2), 4.36 (1H, s, N*H*), 5.23-5.34 (2H, m, $CH{=}CH_2$), 5.85-5.93 (1H, m, C*H*=CH_2); δ_C (125 MHz, $CDCl_3$) 41.1, 45.7, 117.8, 133.5; *m*/*z* (ESI^-) 133.9 ($[M-H]^-$, 100%).

General Procedure B– Alkylation of methanesulfonamides:

Sulfonamide (1.0 equiv.) and alkyl halide (1.2 equiv.) were dissolved in DMF (25 mL) and potassium carbonate (2 equiv.) added. The suspension was heated at 90 °C for 72 h and then allowed to cool to room temperature. Water (30 mL) was then added, followed by ethyl acetate (3 x 30 mL). The solution was then washed with a further portions of water (30 mL) and one of brine (30 mL), dried over Na_2SO_4 and the solvent evaporated. The crude organic residue was then eluted over silica in ethyl acetate, to afford the pure corresponding trifluoromethanesulfonamide.

N-(Cyclohexylmethyl)-N-(3,5-dimethoxybenzyl)methanesulfonamide 33a

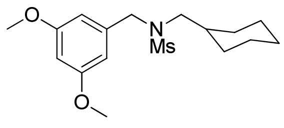

N-(3,5-Dimethoxybenzyl)methanesulfonamide **34e*** (1.96 g, 8 mmol, 1.0 equiv.), was reacted with (bromomethyl)cyclohexane (1.70 g, 9.6 mmol, 1.2 equiv.) according to procedure B to afford *N*-

(cyclohexylmethyl)-N-(3,5-dimethoxybenzyl)methanesulfonamide **33a** as a white solid (1.12 g, 54%). m.p. 59-61 °C; [Found: (HNESP$^+$) (2M+NH$_4$)$^+$, 700.3662. $C_{34}H_{58}N_3O_8S_2^+$ (2M+NH$_4$)$^+$, requires 700.3660; (M+NH$_4$)$^+$, 359.2001. $C_{17}H_{31}N_2O_4S^+$ (M+NH$_4$)$^+$, requires 359.1999; (M+H)$^+$, 342.1738. $C_{17}H_{28}NO_4S$ (M+H)$^+$ requires 342.1734]; ν_{max} (ATR)/cm^{-1} 2934, 2855, 1608, 1431, 1321, 1155, 1045, 1023, 976; δ_H (500 MHz, CDCl$_3$) 0.88-1.00 (3H, m, cyclohexyl), 1.16-1.29 (3H, m, cyclohexyl), 1.55-1.75 (5H, m, cyclohexyl), 2.83 (3H, s, SO$_2$C*H*$_3$), 3.01 (2H, d, *J* = 7.2 Hz, NC*H*$_2$CH), 3.82 (6H, s, ArOC*H*$_3$), 4.35 (2H, s, NC*H*$_2$Ar), 6.42 (1H, t, *J* = 2 Hz, Ar*H*), 6.52 (2H, d, *J* = 2 Hz, Ar*H*); δ_C (125 MHz, CDCl$_3$) 25.8, 26.4, 30.8, 36.0 39.0, 51.9, 53.8, 55.4, 99.7, 106.5, 138.5, 161.0.

_N_-(3,5-Dimethoxybenzyl)-_N_-isopentylmethanesulfonamide 33b

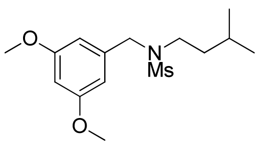

N-(3,5-Dimethoxybenzyl)methanesulfonamide **34e*** (491 mg, 2 mmol, 1 equiv.), was reacted with 1-bromo-3-methylbutane according to procedure B (362 mg, 2.4 mmol, 1.2 equiv.) to afford *N-(3,5-dimethoxybenzyl)-N-isopentylmethanesulfonamide* **33b** as a white solid (347 mg, 55%). m.p. 49-51 °C; [Found: (HNESP$^+$) (M+H)$^+$, 316.1579. $C_{15}H_{26}NO_4S$ (M+H), requires 316.1577]; ν_{max} (ATR)/cm^{-1} 3005, 2945, 2872, 2837, 1588, 1465, 1437, 1320, 1288, 1211, 1144, 1059, 971, 913, 857, 799, 785, 691; δ_H (400 MHz, CDCl$_3$) 0.86 (6H, d, *J* = 6.5 Hz, CH$_2$CHC*H*$_3$), 1.43-1.47 (2H, m, NCH$_2$C*H*$_2$CH), 1.49-1.57 (1H, m, NCH$_2$CH$_2$C*H*CH$_3$), 2.87 (3H, s, SO$_2$C*H*$_3$), 3.20-3.24 (2H, m, NC*H*$_2$CH$_2$), 3.82 (6H, s, ArOC*H*$_3$), 4.33 (2H, s, NC*H*$_2$Ar), 6.52 (2H, d, *J* = 2.2 Hz, Ar*H*), 6.42 (1H, t, *J* = 2.2 Hz, Ar*H*); δ_C (100 MHz, CDCl$_3$) 21.9, 25.3, 36.2, 38.7, 45.3, 50.5, 54.9, 99.3, 105.8, 138.1, 160.6.

_N_-(3,5-Dimethoxybenzyl)-_N_-isobutylmethanesulfonamide 33c

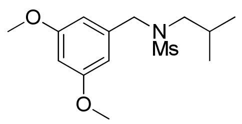

N-(3,5-Dimethoxybenzyl)methanesulfonamide **34e*** (490.6 mg, 2.0 mmol, 1 equiv.), was reacted with 1-iodo-2-methylpropane (441.6 mg, 2.4 mmol, 1.2 equiv.) according to procedure B to afford *N-(3,5-dimethoxybenzyl)-N-isobutylmethanesulfonamide* **33c** as a yellow oil (376 mg, 63%). [Found: (HNESP$^+$) (M+H)$^+$, 302.1423. $C_{14}H_{24}NO_4S$ (M+H), requires 302.1421]; ν_{max} (ATR)/cm^{-1} 2964, 2841, 1599, 1471, 1428, 1323, 1210, 1146, 1040, 950, 881, 833, 699; δ_H (400 MHz, CDCl$_3$) 0.91 (6H, d, *J* = 6.4 Hz, NCH$_2$CHC*H*$_3$), 1.83-1.93 (1H, m, NCH$_2$C*H*CH$_3$), 2.84 (3H, s, SO$_2$C*H*$_3$), 3.01 (2H, d, *J* = 7.6 Hz, NC*H*$_2$CH), 3.82 (6H, s, ArOC*H*$_3$), 4.35 (2H, s, NC*H*$_2$Ar), 6.42 (1H, t, *J* = 2.4 Hz, Ar*H*), 6.53 (2H, d, *J* = 2.4 Hz, Ar*H*); δ_C (100 MHz, CDCl$_3$) 19.5, 26.2, 38.3, 51.4, 54.7, 54.9, 99.2, 106.0, 137.9, 160.5.

***N*-(3,5-dimethoxybenzyl)-*N*-dodecylmethanesulfonamide 33d**

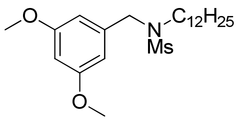

N-(3,5-Dimethoxybenzyl)methanesulfonamide **34e*** (1.23 g, 5 mmol, 1 equiv.), was reacted with dodecylbromide (2.49 g, 10 mmol, 2 equiv.) according to procedure B to afford *N-(3,5-dimethoxybenzyl)-N-dodecylmethanesulfonamide* **33d** as a colourless oil (1.54 g, 74%). [Found: (HNESP$^+$) $(M+H)^+$, 414.2660. $C_{22}H_{40}NO_4S$ (M+H), requires 414.2673]; ν_{max} (ATR)/cm^{-1} 2922, 2852, 1597, 1462, 1323, 1296, 1203, 1145, 1062, 10339, 958, 929, 833, 788, 721; δ_H (400 MHz, $CDCl_3$) 0.89 (3H, t, J = 6.8 Hz, $CH_2CH_2CH_3$), 1.21-1.35 (18H, m, $NCH_2CH_2(CH_2)_9CH_3$), 1.51-1.55 (2H, m, $NCH_2CH_2CH_2$), 2.85 (3H, s, SO_2CH_3), 3.17 (2H, t, J = 7.7 Hz, NCH_2CH_2), 3.80 (6H, s, $ArOCH_3$), 4.32 (2H, s, NCH_2Ar), 6.40 (1H, t, J = 2.2 Hz, Ar*H*), 6.51 (2H, d, J = 2.2 Hz, Ar*H*); δ_C (100 MHz, $CDCl_3$) 14.1, 22.7, 26.7, 28.0, 29.3, 29.5, 29.5, 31.9, 39.1, 47.5, 51.1, 55.4, 99.7, 106.3, 1338.7, 161.0.

***N*-benzyl-*N*-(3,5-dimethoxybenzyl)methanesulfonamide 33e**

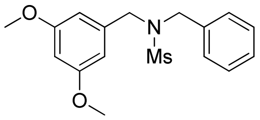

N-(3,5-Dimethoxybenzyl)methanesulfonamide **34e*** (1.23 g, 5 mmol, 1 equiv.), was reacted with benzyl bromide (1.28 g, 7.5 mmol, 1.5 equiv.) according to procedure B to afford *N-benzyl-N-(3,5-dimethoxybenzyl)methanesulfonamide* **33e** as a colourless oil (1.07 g, 64%). [Found: (HNESP$^+$) $(M+H)^+$, 336.1269. $C_{17}H_{22}NO_4S$ (M+H), requires 336.1264]; ν_{max} (ATR)/cm^{-1} 2931, 2839, 1595, 1456, 1319, 1203, 1141, 1049, 927, 788, 698, 686; δ_H (400 MHz, $CDCl_3$) 2.82 (3H, s, SO_2CH_3), 3.80 (6H, s, $ArOCH_3$), 4.30 (2H, s, NCH_2Ar), 4.39 (2H, s, NCH_2Ar), 6.42 (1H, t, J = 2.2 Hz, Ar*H*), 6.48 (2H, d, J = 2.2 Hz, Ar*H*), 7.28-7.39 (5H, m, Ar*H*); δ_C (100 MHz, $CDCl_3$) 39.7, 49.5, 54.9, 99.3, 106.1, 12.6, 128.2, 128.3, 135.0, 137.3, 160.6.

***N*-benzyl-*N*-dodecylmethanesulfonamide 33f**

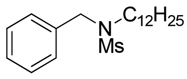

N-Benzylmethanesulfonamide **34e** (1.11 g, 6 mmol, 1.2 equiv.), was reacted with dodecyl bromide (1.25 g, 5 mmol, 1 equiv.) according to procedure B to afford *N-benzyl-N-dodecylmethane-sulfonamide* **33f** as a white solid (1.18 g, 66%); m.p. 35-37 ℃; [Found: (HNESP$^+$) $(M+NH_4)^+$, 371.2731. $C_{20}H_{39}N_2O_2S^+$ $(M+NH_4)$, requires 371.2727]; ν_{max} (ATR)/cm^{-1} 2916, 2848, 1589, 1467, 1327, 1145, 964, 808, 792, 723, 694; δ_H (400 MHz, $CDCl_3$) 0.91 (3H, t, J = 6.8 Hz, CH_2CH_3), 1.20-1.38 (18H, m, $NCH_2CH_2(CH_2)_9CH_3$), 1.51-1.62 (2H, m, $NCH_2CH_2CH_2$), 2.85 (3H, s, SO_2CH_3), 3.18

(2H, dd, J = 7.8, 7.5 Hz, NCH_2CH$_2$), 4.41 (2H, s, NCH_2Ar), 7.29-7.39 (5H, m, ArH); δ_C (100 MHz, CDCl$_3$) 13.6, 22.2, 26.1, 27.5, 28.7, 28.8, 29.5, 29.0, 29.1, 31.4, 38.7, 46.9, 50.5, 127.4, 127.9, 128.2, 135.7.

***N*-benzyl-*N*-(cyclohexylmethyl)methanesulfonamide 33g**

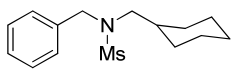

N-Benzylmethanesulfonamide **34e** (1.11 g, 6 mmol, 1.1 equiv.), was reacted with cyclohexylmethyl bromide (885 mg, 5 mmol, 1 equiv.) according to procedure B to afford *N-benzyl-N-(cyclohexylmethyl)methanesulfonamide* **33g** as a white solid (1.36 g, 97%); m.p. 97-99 ℃; [Found: (HNESP$^+$) (M+H)$^+$, 282.1526. $C_{15}H_{24}NO_2S^+$ (M+H), requires 282.1522]; ν_{max} (ATR)/cm^{-1} 2918, 2845, 1444, 1323, 1143, 1020, 964, 885, 796, 788, 754, 719, 698; δ_H (400 MHz, CDCl$_3$) 0.81-0.96 (2H, m, cyclohexyl), 1.11-1.22 (3H, m, cyclohexyl), 1.49-1.60 (2H, m, cyclohexyl), 1.62-1.79 (4H, m, cyclohexyl), 2.79 (3H, s, SO$_2$CH_3), 3.02 (2H, d, J = 7.4 Hz, NCH_2CH), 4.41 (2H, s, NCH_2Ar), 7.29-7.39 (5H, m, ArH); δ_C (100 MHz, CDCl$_3$) 25.2, 25.9, 30.2, 35.5, 38.5, 51.3, 53.2, 127.5, 128.2, 135.6.

***N*-Butyl-*N*-(4-(trifluoromethyl)benzyl)methanesulfonamide 33h**

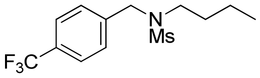

N-(3,5-Dimethoxybenzyl)bromide (1.20 g, 5 mmol, 1 equiv.), was reacted with *N*-butylmethanesulfonamide **34h** (832 mg, 5.5 mmol, 1.1 equiv.) according to procedure B to afford *N-butyl-N-(4-(trifluoromethyl)benzyl)methanesulfonamide* **33h** as a white solid (789 mg, 51%); m.p. 52-54 ℃; [Found: (HNESP$^+$) (M+H)$^+$, 310.1091. $C_{13}H_{19}F_3NO_2S^+$ (M+H), requires 310.1083]; ν_{max} (ATR)/cm^{-1} 2964, 2933, 2864, 1319, 1288, 1109, 1066, 1014, 966, 906, 785, 732, 694; δ_H (400 MHz, CDCl$_3$) 0.89 (3H, t, J = 7.6 Hz, CH$_2$CH_3), 1.32-1.33 (2H, m, CH$_3$CH_2CH$_3$), 1.47-1.56 (2H, m, NCH$_2$CH_2CH$_2$), 2.91 (3H, s, SO$_2$CH_3), 3.20 (2H, m, NCH_2CH$_2$), 4.46 (2H, s, NCH_2Ar), 7.51 (2H, d, J = 7.8, ArH), 7.64 (2H, d, J = 7.8, ArH); δ_C (100 MHz, CDCl$_3$) 13.5, 19.8, 30.1, 38.8, 47.8, 50.8, 124.1 (q, 272.5 Hz), 125.5 (q, J = 3.8 Hz), 129.0 (q, J = 31.3 Hz), 141.0.

***N*-Cyclohexyl-*N*-(4-(trifluoromethyl)benzyl)methanesulfonamide 33i**

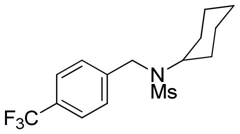

Sodium hydride (as a 60% suspension in mineral oil, 160 mg, 4 mmol, 2 equiv.) was rinsed with dry hexane (3 x 20 mL) and blown dry under argon. To this was added dry DMF (10 mL) followed by a solution of cyclohexylmethanesulfonamide **34i** (478 mg, 2 mmol, 1 equiv.) in dry DMF (10 mL). 1-

(Bromomethyl)-4-(trifluoromethyl)benzene (355 mg, 2 mmol, 1 equiv.) was dissolved in dry DMF (10 ml) and added to the mixture which was then stirred overnight at room temperature. The reaction mixture was poured into water (40 ml) and ethyl acetate (40 mL) and rinsed with further ethyl acetate (3 x 40 mL). This solution was then sequentially rinsed with water (3 x 30 mL), dried over sodium sulfate, filtered and evaporated under reduced pressure. The precipitate was eluted over silica (30 % ethyl acetate in petroleum ether) to afford *N-cyclohexyl-N-(4-(trifluoromethyl)benzyl)-methanesulfonamide* **33i** as a white solid (633 mg, 89%); m.p. 106-108 ℃; [Found: ($HNESP^+$) $(M+H)^+$, 336.1249. $C_{15}H_{21}F_3NO_2S^+$ (M+H), requires 336.1240]; ν_{max} (ATR)/cm^{-1} 2943, 2862, 1319, 1139, 1070, 974, 850, 810, 783, 742; δ_H (400 MHz, $CDCl_3$) 0.95-1.06 (1H, m, cyclohexyl), 1.27-1.41 (4H, m, cyclohexyl), 1.60-1.64 (1H, m, cyclohexyl), 1.78-1.80 (4H, m, cyclohexyl), 2.87 (3H, s, SO_2CH_3), 3.69-3.76 (1H, m, NC*H*CH_2), 4.44 (2H, s, NC*H*$_2$Ar), 7.53 (2H, d, *J* = 7.8, Ar*H*), 7.60 (2H, d, *J* = 7.8, Ar*H*); δ_C (100 MHz, $CDCl_3$) 24.6, 25.5, 31.6, 39.8, 46.2, 58.0, 123.8. (q, *J* = 271.6 Hz), 124.8 (q, *J* = 3.6Hz), 129.1(q, *J* = 32.8), 142.6.

Synthesis of *N,N*-dioctylmethanesulfonamide 38[19]

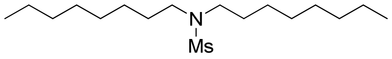

To a solution of *N,N*-dioctylamine **15** (15.11 mL, 50 mmol, 1 equiv.) and triethylamine (10.4 mL, 75 mmol, 1.5 equiv.) in dichloromethane (50 mL) at 0 °C, was added methanesulfonyl chloride (4.64 mL, 60 mmol, 1.2 equiv.). The reaction mixture was allowed to warm to room temperature and left stirring overnight, then quenched with 2N aqueous HCl (50 mL). The aqueous fraction was extracted with further dichloromethane (2 x 30 mL) and the combined organic fractions washed with additional aqueous HCl (2 x 30 mL), water (30 mL) and brine (30 mL), and then dried with anhydrous sodium sulfate, filtered and concentrated under reduced pressure to yield *N,N*-dioctylmethanesulfonamide **38**[19] as a pale yellow oil (15.50 g, 97 %); [Found: ($HNESP^+$) $(M+H)^+$, 320.2624. $C_{17}H_{38}NO_2S^+$ (M+H), requires 320.2618]; ν_{max} (ATR)/cm^{-1} 2924, 2854, 1465, 1332, 1143, 958, 783, 736; δ_H (400 MHz, $CDCl_3$) 0.85 (6H, t, *J* = 7.2 Hz, CH_2CH_3), 1.25-1.28 (20H, m, $NCH_2CH_2(CH_2)_5CH_3$), 1.54-1.60 (4H, m, $NCH_2CH_2CH_2$), 2.79 (3H, s, SO_2CH_3), 3.13 (4H, t, *J* = 7.7 Hz, NCH_2CH_2); δ_C (100 MHz, $CDCl_3$) 13.6, 22.1, 26.2, 28.2, 28.7, 31.3, 37.6, 47.3.

***N*-Allyl-*N*-benzylmethanesulfonamide 35a**[20]

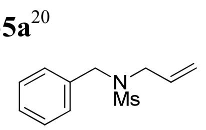

N-Benzylmethanesulfonamide **34e** (1.21 g, 10 mmol, 1 equiv.), was reacted with allyl bromide (1.81 g, 15 mmol, 1.5 equiv.) according to procedure B to afford *N*-allyl-*N*-benzylmethanesulfonamide **35a** as a colourless oil (1.60 g, 71%); ν_{max} (ATR)/cm^{-1} 3083, 3032, 2924, 2857, 1642, 1500, 1448, 1323, 1146, 1045, 929, 898, 754; δ_H (400 MHz, $CDCl_3$) 2.89 (3H, s, SO_2CH_3), 3.81(2H, d, *J* = 6.4 Hz,

NCH_2CH), 4.42 (2H, s, NCH_2Ar), 5.24-5.31 (2H, m, NCH_2CHCH), 5.79-5.89 (1H, m, NCH_2CHCH), 7.42-7.41 (5H, m, Ar*H*); δ_C (100 MHz, $CDCl_3$) 39.6, 48.5, 49.4, 119.4, 127.5, 128.0, 128.2, 131.6, 135.2; *m*/*z* (ESI^+) 225.9 ($[M+H]^+$).

_N_-Allyl-_N_-phenethylmethanesulfonamide 35b

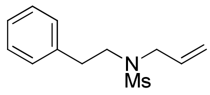

(2-Bromoethyl)benzene (925 mg, 5 mmol, 1 equiv.), was reacted with *N*-allylmethanesulfonamide **37** (811 mg, 6 mmol, 1.2 equiv.) according to procedure B to afford *N-allyl-N-phenethylmethanesulfonamide* **35b** as a white solid (550 mg, 46%); m.p. 55-57 ℃; [Found: ($HNESP^+$) $(M+H)^+$, 240.1056. $C_{12}H_{18}NO_2S$ (M+H), requires 240.1053]; ν_{max} (ATR)/cm^{-1} 3012, 2943, 2870, 1460, 1319, 1280, 1138, 1105, 923, 756, 732, 702; δ_H (400 MHz, $CDCl_3$) 2.73 (3H, s, SO_2CH_3), 2.92 (2H, t, J = 7.5 Hz, $ArCH_2CH_2N$), 3.47 (2H, t, J = 7.5 Hz, $ArCH_2CH_2N$), 3.86 (2H, d, J = 6.8 Hz, NCH_2CH), 5.28-5.33 (2H, m, NCH_2CHCH), 5.79-5.89 (1H, m, NCH_2CHCH), 7.22-7.35 (5H, m, Ar*H*); δ_C (100 MHz, $CDCl_3$) 34.8, 38.7, 48.0, 49.7, 118.8, 126.2, 128.1, 128.4, 132.4, 138.0.

_N_-Allyl-_N_-(3-phenylpropyl)methanesulfonamide 35c

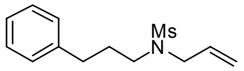

N-Allylmethanesulfonamide **37** (675 mg, 5 mmol, 1 equiv.) and (3-bromopropyl)benzene (1.49 g, 7.5 mmol, 1.5 equiv.) were reacted according to procedure B to afford *N-allyl-N-(3-phenylpropyl)methanesulfonamide* **35c** as a colourless oil (1.01 g, 80%); [Found: ($HNESP^+$) $(M+H)^+$, 254.1209. $C_{13}H_{20}NO_2S^+$ (M+H), requires 254.1212]; ν_{max} (ATR)/cm^{-1} 3026, 2931, 2862, 1454, 1321, 1141, 993, 960, 925, 785, 746, 698; δ_H (400 MHz, $CDCl_3$) 1.91-1.99 (2H, m, $ArCH_2CH_2CH_2N$), 2.64-2.68 (2H, m, $ArCH_2CH_2CH_2N$), 3.22-3.27 (2H, m, $ArCH_2CH_2CH_2N$) , 3.86 (2H, d, J = 6.4 Hz, NCH_2CHCH), 5.24-5.31 (2H, m, NCH_2CHCH), 5.79-5.89 (1H, m, NCH_2CHCH), 7.19-7.24 (3H, m, Ar*H*), 7.28-7.33 (2H, m, Ar*H*); δ_C (100 MHz, $CDCl_3$) 29.5, 32.3, 46.3, 49.6, 118.7, 125.6, 127.8, 128.0, 132.3, 140.7.

_N_-Allyl-_N_-dodecylmethanesulfonamide 35d

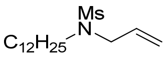

N-Allylmethanesulfonamide **37** (675 mg, 5 mmol, 1 equiv.) and dodecyl bromide (1.87 g, 7.5 mmol, 1.5 equiv.) were reacted according to procedure B to afford *N-allyl-N-dodecylmethanesulfonamide* **35d** as a white solid (1.22 g, 80%); m.p. 40-42 ℃; [Found: ($HNESP^+$) $(M+H)^+$, 304.2311. $C_{16}H_{34}NO_2S^+$ (M+H), requires 304.2305]; ν_{max} (ATR)/cm^{-1} 2914, 2845, 1323, 1141, 1112, 939, 916, 806, 786; δ_H (400 MHz, $CDCl_3$) 0.91 (3H, t, J = 6.4 Hz, CH_2CH_3), 1.24-1.33 (18H, m,

$NCH_2CH_2(CH_2)_9CH_3$), 1.55-1.62 (2H, m, $NCH_2CH_2CH_2$), 2.87 (3H, s, SO_2CH_3), 3.16-3.22 (2H, m, NCH_2CH_2), 3.87 (2H, d, J = 6.4 Hz, NCH_2CH), 5.25-5.33 (2H, m, NCH_2CHCH_2), 5.85 (1H, ddt, J = 17.0, 10.2, 6.4 Hz, NCH_2CHCH_2); δ_C (100 MHz, $CDCl_3$) 13.6, 22.2, 26.1, 26.1, 17.8, 28.7, 28.8, 29.0, 29.1, 31.4, 38.6, 46.6, 49.4, 117.5, 132.5.

Reaction of *N*-allyl-*N*-dodecylmethanesulfonamide 35d with donor 3

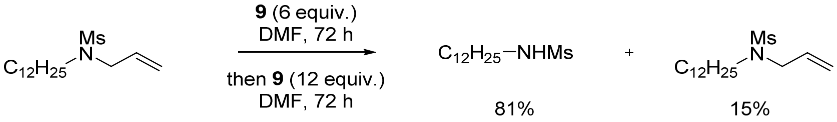

Donor **3** (512 mg, 1.8 mmol, 6 equiv.) was dissolved in degassed DMF (4 mL) in a glove-box. This solution was directly pipetted into the substrate, *N*-allyl-*N*-dodecylmethanesulfonamide **35d** (91 mg, 0.3 mmol). The solution was removed to a fumehood and stirred at room temperature for 72 h under UV radiation (365 nm). The sealed reaction flask was then returned to the glove-box and an additional portion of donor **3** (512 mg, 1.8 mmol, 6 equiv.) added. The flask was resealed and returned to the irradiation set-up for a further 72 h. The mixture was poured into aqueous 2N HCl (25 mL) and ethyl acetate (25 mL) before extracting with ethyl acetate (4 x 15 mL). The combined organic layers were then washed with water (3 x 20 mL) and brine (10 mL) and dried over Na_2SO_4. The crude organic residue, obtained after evaporation under reduced pressure, was eluted with 15 % ethyl acetate/ petroleum ether on silica gel, to afford the pure corresponding products as reported.

N-dodecylmethanesulfonamide **36d** was isolated as a white solid (64 mg, 81%). Data were as reported above.

N-allyl-*N*-dodecylmethanesulfonamide **35d** was recovered as a white solid (14 mg, 15%). Data were as reported above for this compound.

Synthesis of *N*-allyl-*N*-isopentylmethanesulfonamide 35e

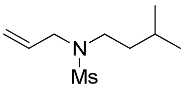

To a solution of *N*-allylmethanesulfonamide **37** (744 mg, 5.5 mmol, 1.1 equiv.) and allyl bromide (599 µL, 5 mmol, 1 equiv.) in DMF (10 mL) was added potassium carbonate (1.38 g, 10 mmol, 2 eq). The reaction mixture was left stirring at room temperature overnight, then quenched with 2N aqueous NaOH (25 mL). The aqueous fraction was rinsed with diethyl ether (3 x 20 mL) and the combined organic fractions rinsed with additional aqueous NaOH (2 x 15 mL), water (30 mL) and brine (30 mL) then dried with anhydrous sodium sulfate, filtered and concentrated under reduced pressure to yield *N-allyl-N-isopentylmethanesulfonamide* **35e** (982 mg, 87 %) as a colourless oil; [Found: ($HNESP^+$) $(M-H)^+$, 204.1051. $C_9H_{18}NO_2S^+$ (M-H), requires 204.1053]; ν_{max} (ATR)/cm^{-1} 2958, 2870, 1321, 1141, 960, 916, 786; δ_H (400 MHz, $CDCl_3$) 0.93 (6H, d, J = 6.3 Hz, $CHCH_3$), 1.49 (2H, dt, J = 7.4, 7.0 Hz,

NCH_2CH_2CH), 1.56-1.64 (1H, m, NCH_2CH_2CH), 2.87 (3H, s, SO_2CH_3), 3.22 (2H, t, J = 7.5 Hz, NCH_2CH_2), 3.86 (2H, d, J = 6.2 Hz, NCH_2CHCH_2), 5.26-5.34 (2H, m, NCH_2CHCH_2), 5.80-5.91 (1H, m, NCH_2CHCH_2); δ_C (100 MHz, $CDCl_3$) 22.3, 22.7, 37.0, 39.0, 45.4, 49.8, 118.9, 130.0.

General procedure C – Reaction of methanesulfomamides with donor 3

Donor **3** (512 mg, 1.8 mmol, 6 equiv.) was dissolved in degassed DMF (4 mL) in a glove-box. This solution was directly pipetted into the substrate (0.3 mmol, 1.0 equiv.). The solution was removed to a fumehood and stirred at room temperature for 72 h under UV radiation (365 nm). The mixture was poured into aqueous 2N HCl (25 mL) and ethyl acetate (25 mL) before extracting with ethyl acetate (4 x 15 mL). The combined organic layers were then washed with water (3 x 20 mL) and brine (10 mL) and dried over Na_2SO_4. The crude organic residue, obtained after evaporation under reduced pressure, was eluted with ethyl acetate on silica gel, to afford the pure corresponding products as reported.

Reaction of *N*-(cyclohexylmethyl)-*N*-(3,5-dimethoxybenzyl)methanesulfonamide 33a with donor 3

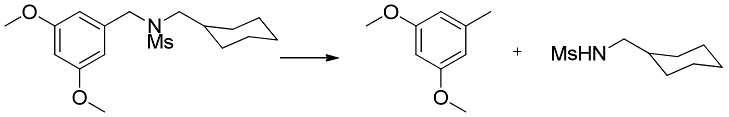

N-(Cyclohexylmethyl)-*N*-(3,5-dimethoxybenzyl)methanesulfonamide **33a** (102 mg, 0.3 mmol), was reacted according to procedure C to afford the products given below.

N-(cyclohexylmethyl)methanesulfonamide **34a**[21] was isolated as a white solid (46 mg, 80%); m.p. 70-72 °C (lit.[21] 71-73 °C); ν_{max} (ATR)/cm^{-1} 3280, 2928, 2854, 1459, 1435, 1301, 1144, 1059, 978, 954, 837, 755, 688; δ_H (400 MHz, $CDCl_3$) 0.89-1.01 (2H, m, cyclohexyl), 1.13-1.32 (3H, m, cyclohexyl), 1.44-1.56 (1H, m, cyclohexyl), 1.65-1.79 (5H, m, cyclohexyl), 2.96 (3H, s, SO_2CH_3) overlapping with 2.96-3.00 (2H, m, NCH_2CH), 4.40-4.49 (1H, bs, N*H*); δ_C (100 MHz, $CDCl_3$) 25.2, 25.8, 30.1, 37.6, 39.8, 49.0; *m*/*z* (EI$^+$) 191.0 ([M]$^+$, 6%), 110.0 (32), 108.0 (100), 96.0 (47), 83.0 (38), 54.9 (30).

In the NMR spectrum of the crude reaction product, peaks (at 2.33 (3H, s, $ArCH_3$), 3.80 (6H, s, $ArOCH_3$), 6.32 (1H, d, J = 2.1 Hz, Ar*H*), 6.36 (2H, d, J = 2.1Hz, Ar*H*)) tentatively identified 3,5-dimethoxytoluene,[22] when compared to the authentic material.

N-(cyclohexylmethyl)-*N*-(3,5-dimethoxybenzyl)methanesulfonamide **33a** was recovered as a white solid (9 mg, 9 %) with data matching those reported above for this compound.

Blank reaction of *N*-(cyclohexylmethyl)-*N*-(3,5-dimethoxybenzyl)methanesulfonamide 33a without photoactivation

In parallel, *N*-(cyclohexylmethyl)-*N*-(3,5-dimethoxybenzyl)methanesulfonamide **33a** (102 mg, 0.3 mmol), was reacted according to procedure C with the omission of the u.v. activation to afford only

recovery of *N*-(cyclohexylmethyl)-*N*-(3,5-dimethoxybenzyl)methanesulfonamide **33a** (96 mg, 94%) with data matching those reported above for this compound.

Reaction of *N*-(3,5-dimethoxybenzyl)-*N*-isopentylmethanesulfonamide 33b with donor 3

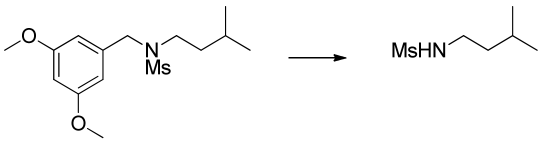

N-(3,5-Dimethoxybenzyl)-*N*-isopentylmethanesulfonamide **33b** (95 mg, 0.3 mmol), was reacted according to procedure C to afford the mixture of products given below.
N-isopentylmethanesulfonamide **34b**[23] was isolated as a colourless oil (41 mg, 82 %); ν_{max} (ATR)/cm^{-1} 3271, 2963, 2872, 1464, 1440, 1295, 1127, 1069, 1039, 991, 972, 941, 881, 816, 715; δ_H (400 MHz, $CDCl_3$) 0.94 (6H, d, *J* = 6.8 Hz, CHC*H*$_3$), 1.48 (2H, dt, *J* = 7.3, 6.8 Hz, NCH_2C*H*$_2$), 1.69 (1H, nonet, *J* = 6.8 Hz, CH_2C*H*CH_3), 2.97 (3H, s, SO_2C*H*$_3$), 3.16 (2H, dt, *J* = 7.6, 7.3 Hz, NC*H*$_2$$CH_2$), 4.29-4.28 (1H, bs, N*H*); δ_C (100 MHz, $CDCl_3$) 21.8, 25.0, 38.4, 39.7, 41.1; *m/z* (ESI$^-$) 164.00 ([M-H]$^-$, 100%).
N-(3,5-dimethoxybenzyl)-N-isopentylmethanesulfonamide **33b** was recovered as a white solid (11 mg, 12 %) with data matching those reported above for this compound.

Reaction of *N*-(3,5-dimethoxybenzyl)-*N*-isopentylmethanesulfonamide 33b in the presence of less donor 3

When *N*-isopentyl-*N*-(3,5-dimethoxybenzyl)methanesulfonamide **33b** (95 mg, 0.3 mmol), was reacted according to procedure C with only 3 equiv. donor **3** the following were isolated: *N*-(3,5-dimethoxybenzyl)-*N*-isopentylmethanesulfonamide **33b** (11 mg, 12 %), *N*-isopentylmethanesulfonamide **34b** was isolated as a colourless oil (41 mg, 82 %) and 3,5-dimethoxytoluene[22] as a colourless oil (4 mg, 5%); ν_{max} (ATR)/cm^{-1} 2497, 3213, 1593, 1476, 1153, 1060, 786; δ_H (400 MHz, $CDCl_3$) 2.33 (3H, s, ArC*H*$_3$), 3.80 (6H, s, ArOC*H*$_3$), 6.32 (1H, d, *J* = 2.1 Hz, Ar*H*), 6.36 (2H, d, *J* = 2.1Hz, Ar*H*); δ_C (100 MHz, $CDCl_3$) 21.3, 54.7, 97.0, 106.6, 139.7, 160.2; *m/z* (ESI$^+$) 153.00 ([M+H]$^+$, 100%).

Reaction of *N*-(3,5-dimethoxybenzyl)-*N*-isobutylmethanesulfonamide 33c with donor 3

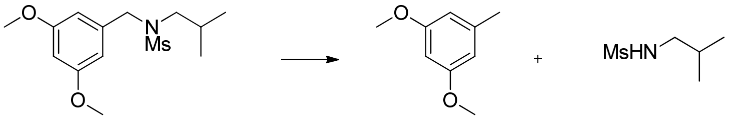

N-(3,5-dimethoxybenzyl)-*N*-isobutylmethanesulfonamide **33c** (72 mg, 0.3 mmol), was reacted according to procedure C to afford *N*-isobutylmethanesulfonamide **34c** as a colourless oil (36 mg, 79 %); ν_{max} (ATR)/cm^{-1} 3256, 3016, 2958, 2868, 1474, 1448, 1304, 1136, 1067, 976, 881, 817, 762; δ_H (400 MHz, $CDCl_3$) 0.96 (6H, d, *J* = 6.5 Hz, CHC*H*$_3$), 1.77-1.87 (1H, m, CH_2C*H*CH_3), 2.93-2.96 (2H,

m, NCH_2CH), 2.96 (1H, s, NH), 4.72 (3H, s, SO_2CH_3); δ_C (100 MHz, $CDCl_3$) 19.4, 28.3, 39.6, 50.1; *m/z* (ESI^-) 149.93 ($[M-H]^-$, 100%).

Peaks at δ 2.33 (3H, s, ArCH_3), 3.80 (6H, s, ArOCH_3), 6.32 (1H, d, J = 2.1 Hz, ArH), 6.36 (2H, d, J = 2.1Hz, ArH)) tentatively identified 3,5-dimethoxytoluene in trace amounts in a ^{1}H NMR spectrum of the crude reaction mixture when compared to the authentic material.

Reaction of *N*-(3,5-dimethoxybenzyl)-*N*-dodecylmethanesulfonamide 33d with donor 3

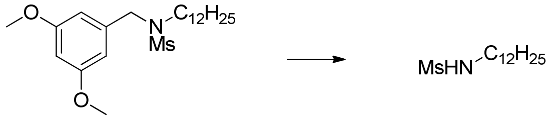

N-(3,5-Dimethoxybenzyl)-*N*-dodecylmethanesulfonamide **33d** (124 mg, 0.3 mmol), was reacted according to procedure C to afford the products given below.

N-Dodecylmethanesulfonamide **34d**[24] was isolated as a white solid (50 mg, 64%); m.p. 75-77 ℃; [Found: ($HNESP^+$) $(M+NH_4)^+$, 281.2262. $C_{13}H_{33}N_2O_2S^+$ $(M+NH_4)$, requires 281.2258]; ν_{max} (ATR)/cm^{-1} 3267, 2957, 2957, 2850, 1415, 1031, 1141, 1132, 10664, 983, 889, 761, 717; δ_H (400 MHz, $CDCl_3$) 0.88 (3H, t, J = 7.7 Hz, CH_2CH_3), 1.22-1.41 (18H, m, $NCH_2CH_2(CH_2)_9CH_3$), 1.50-1.55 (2H, m, $NCH_2CH_2CH_2$), 2.95 (3H, s, SO_2CH_3), 3.11 (q, J = 6.9 Hz, NCH_2CH_2), 4.40-4.50 (1H, bs, NH); δ_C (100 MHz, $CDCl_3$) 14.1, 22.7, 26.6, 29.1, 29.3, 29.4, 29.5, 29.6, 30.1, 31.9, 40.2, 43.4.

Residual *N*-(3,5-dimethoxybenzyl)-*N*-dodecylmethanesulfonamide **33d** was recovered as a white solid (26 mg, 21%) with spectroscopic data matching those reported above for this compound.

Reaction of *N*-benzyl-*N*-(3,5-dimethoxybenzyl)methanesulfonamide 33e with donor 3

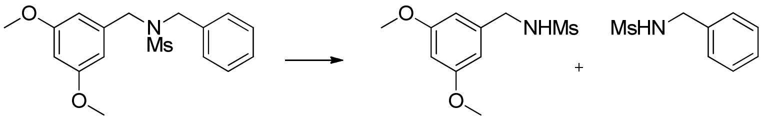

N-benzyl-*N*-(3,5-dimethoxybenzyl)methanesulfonamide **33e** (100.6 mg, 0.3 mmol), was reacted according to procedure C to afford the mixture of products given below.

N-(3,5-dimethoxybenzyl)methanesulfonamide **34e*** was isolated as a white solid (21 mg, 28%). Data were as reported above for this compound.

N-benzylmethanesulfonamide **34e**[15] was isolated as a white solid (20 mg, 35%), with spectroscopic data matching those in the literature.

N-benzyl-N-(3,5-dimethoxybenzyl)methanesulfonamide **33** was recovered as a white solid (7 mg, 7 %) with spectroscopic data matching those reported above for this compound.

Reaction of *N*-benzyl-*N*-dodecylmethanesulfonamide 33f with donor 3

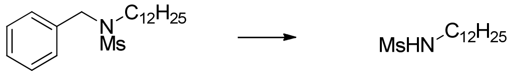

N-benzyl-*N*-dodecylmethanesulfonamide **33f** (106 mg, 0.3 mmol), was reacted according to procedure C to afford the products below.

N-dodecylmethanesulfonamide **34d** was isolated as a white solid (64 mg, 80%) with data as reported previously.

N-benzyl-*N*-dodecylmethanesulfonamide **33f** was recovered as a white solid (16 mg, 15%), with spectroscopic data matching those reported above for this compound.

Reaction of *N*-benzyl-*N*-(cyclohexylmethyl)methanesulfonamide 33g with donor 3

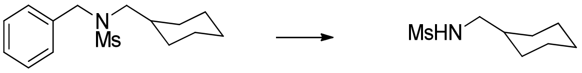

N-benzyl-*N*-(cyclohexylmethyl)methanesulfonamide **33g** (84 mg, 0.3 mmol), was reacted according to procedure C to afford the products given below.

N-cyclohexylmethylmethanesulfonamide **34a** was isolated as a white solid (41 mg, 71%). Data were as reported above in this paper.

N-benzyl-*N*-(cyclohexylmethyl)methanesulfonamide **33g** was recovered as a white solid (12 mg, 14%) with spectroscopic data matching those reported above for this compound.

Reaction of *N*-butyl-*N-(4*-(trifluoromethyl)benzyl)methanesulfonamide 33h with donor 3

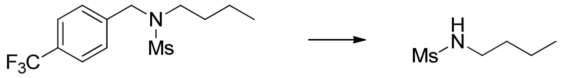

N-butyl-*N*-(4-(trifluoromethyl)benzyl)methanesulfonamide **33h** (92 mg, 0.3 mmol), was reacted according to procedure C to afford *N*-butylmethanesulfonamide **34h** as a pale yellow oil (38 mg, 84%). Data were as reported above in this paper.

Reaction of *N-cyclohexyl-N-(4-(trifluoromethyl)benzyl)methanesulfonamide* 33i with donor 3

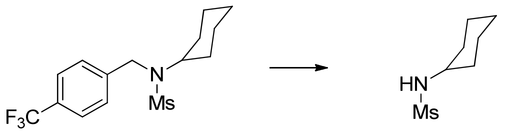

N-cyclohexyl-N-(4-(trifluoromethyl)benzyl)methanesulfonamide **33i** (102 mg, 0.3 mmol), was reacted according to procedure C to afford *N*-cyclohexyl)methanesulfonamide **34i** as a white solid (40 mg, 75%) with spectroscopic data matching those reported above for this compound.

Reaction of *N*,*N*-dioctylmethanesulfonamide 38 with photoactivated donor

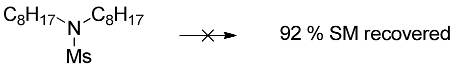

N,*N*-dioctylmethanesulfonamide **38** (96 mg, 0.3 mmol, 1 equiv.) was reacted according to general procedure C to afford *N*,*N*-dioctylmethanesulfonamide **38** as a pale yellow oil (88 mg, 92 %) with data matching those reported above.

Reaction of *N*-allyl-*N*-benzylmethanesulfonamide 35a with donor 3

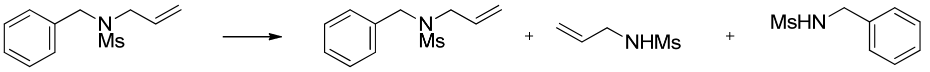

N-allyl-*N*-benzylmethanesulfonamide **35a** (68 mg, 0.3 mmol), was reacted according to procedure C to afford the mixture of products given below.

N-benzylmethanesulfonamide **36a** and *N*-allylmethanesulfonamide **37** were obtained as a mixture as a colourless oil (30.5 mg) which was tentatively identified by NMR in the following amounts: *N*-allylmethanesulfonamide (25.1 mg, 62%) and *N*-benzylmethanesulfonamide (5.4 mg, 10%).

N-allyl-*N*-benzylmethanesulfonamide **35a** was recovered as a white solid (12 mg, 15%) with data as reported above.

Reaction of *N*-allyl-*N*-phenethylmethanesulfonamide 35b with donor 9

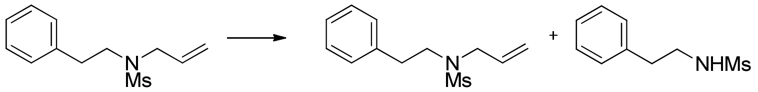

N-allyl-*N*-phenethylmethanesulfonamide **35b** (72 mg, 0.3 mmol), was reacted according to procedure C to afford the products given below.

N-phenethylmethanesulfonamide **36b**[25] was isolated as a colourless oil (24 mg, 41%); ν_{max} (ATR)/cm^{-1} 3288, 3026, 2929, 2858, 1408, 1309, 1139, 1072, 970, 893, 815, 765, 748, 698; δ_H (400 MHz, $CDCl_3$) 2.83 (3H, s, SO_2CH_3), 2.88 (2H, t, J = 6.6 Hz, NCH_2CH_2Ar), 3.41 (2H, t, J = 6.6 Hz, NCH_2CH_2Ar), 4.30-4.40 (1H, bs, N*H*), 7.20-7.27 (3H, m, Ar*H*), 7.30-7.36 (2H, m, Ar*H*); δ_C (100 MHz, $CDCl_3$) 36.5, 40.4, 44.4, 127.0, 128.9, 137.8; *m/z* (APCI$^+$) 198.1 ([M+H]$^+$, 100%).

N-allyl-*N*-phenethylmethanesulfonamide **35b** was recovered as a white solid (41 mg, 57%). Data were as reported above for this compound.

Blank reaction of *N*-allyl-*N*-phenethylmethanesulfonamide 35b in the absence of photoactivation

In parallel, *N*-allyl-*N*-phenethylmethanesulfonamide **35b** (72 mg, 0.3 mmol), was reacted according to procedure C with the omission of the UV activation, to afford quantative recovery of *N*-allyl-*N*-phenethylmethanesulfonamide **35b** with data as reported above for this compound.

Reaction of *N*-allyl-*N*-(3-phenylpropyl)methanesulfonamide 35c with donor 3

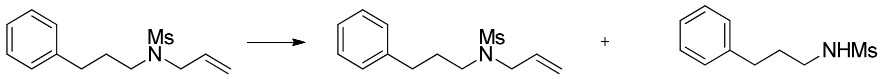

N-allyl-*N*-(3-phenylpropyl)methanesulfonamide **35c** (91 mg, 0.3 mmol), was reacted according to procedure C to afford the mixture of products given below.

N-(3-phenylpropyl)methanesulfonamide **36c**[26] was isolated as a white solid (33 mg, 42%); [Found: (HNESP$^+$) (M+H)$^+$, 214.0898. $C_{10}H_{16}NO_2S^+$ (M+H), requires 214.0896]; m.p. 44-46 °C (no literature m.p. reported); ν_{max} (ATR)/cm^{-1} 3251, 3022, 2933, 2864, 1438, 1415, 1307, 1136, 1058, 1022, 970, 846, 777, 761, 705; δ_H (400 MHz, $CDCl_3$) 1.89-1.96 (2H, m, $ArCH_2CH_2CH_2N$), 2.71 (2H, t, J = 7.6 Hz, $ArCH_2CH_2CH_2N$), 2.94 (3H, s, SO_2CH_3), 3.16 (2H, q, J = 6.7 Hz, $ArCH_2CH_2CH_2N$), 7.18-7.24 (3H, m, Ar*H*), 7.28-7.33 (2H, m, Ar*H*); δ_C (100 MHz, $CDCl_3$) 31.6, 32.8, 40.3, 42.7, 126.2, 128.3, 128.5, 140.8.

N-allyl-*N*-(3-phenylpropyl)methanesulfonamide **35c** was recovered as a colourless oil (42 mg, 27%).. Data were as reported above for this compound.

Reaction of *N*-allyl-*N*-dodecylmethanesulfonamide 35d with donor 3

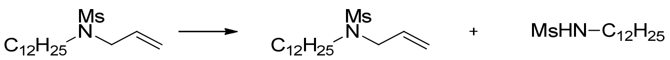

N-allyl-*N*-dodecylmethanesulfonamide **35d** (91 mg, 0.3 mmol), was reacted according to procedure C to afford the products given below.

N-dodecylmethanesulfonamide **36d** was isolated as a white solid (50 mg, 63%). Data were as reported above.

N-allyl-*N*-dodecylmethanesulfonamide **35d** was recovered as a white solid (29 mg, 32%). Data were as reported above for this compound.

Reaction of *N*-allyl-*N*-isopentylmethanesulfonamide 35e with donor 3

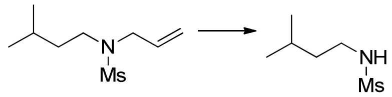

N-allyl-*N*-isopentylmethanesulfonamide **35e** (62 mg, 0.3 mmol, 1 equiv.) was reacted according to general procedure C to afford *N*-isopentylmethanesulfonamide **36e** (25 mg, 50%) and *N*-allyl-*N*-isopentylmethanesulfonamide **35e** (23 mg, 38 %) with data matching those reported above.

Synthesis of *N*-allyl-*N*-methylaniline 39a[27]

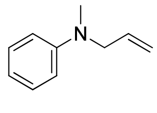

To a solution of *N*-methylaniline **40a** (5.41 mL, 50 mmol, 1 equiv.) in ethanol (100 mL) was added sodium carbonate (7.95 g, 55 mmol, 1.5 equiv.) and allyl bromide (6.08 mL, 55 mmol, 1.1 eq) and the

suspension stirred at reflux for 14 h. The reaction was quenched with water (100 mL) and extracted with ethyl acetate (4 x 50 mL). The combined organic fractions were washed with water (4 x 50 mL) and brine (25 mL) then, dried with anhydrous sodium sulfate, filtered and concentrated under vacuum. Silica column chromatography (0.5-1% ethyl acetate/ petroleum ether) afforded *N*-allyl-*N*-methylaniline **39a**[27] as a pale yellow oil (6.55 g, 89 %); ν_{max} (ATR)/cm^{-1} 2894, 2806, 1597, 1502, 1365, 1207, 991, 916, 744, 688; δ_H (400 MHz, $CDCl_3$) 2.98 (3H, s, NC*H*$_3$), 3.95 (2H, ddd, *J* = 5.1, 1.6, 1.5 Hz, NC*H*$_2$), 5.17-5.24 (2H, m, NCH_2CHCH_2), 5.88 (1H, ddt, *J* = 17.2, 10.3, 5.1 Hz, NCH_2CH), 6.72 (1H, t, *J* = 7.3 Hz, Ar*H*), 6.76 (2H, d, *J* = 8.4 Hz, Ar*H*), 7.26-7.28 (2H, m, Ar*H*); δ_C (100 MHz, $CDCl_3$) 37.5, 54.8, 112.0, 115.6, 115.9, 128.6, 133.4, 149.0; *m/z* (MM-ES+APCI$^+$) 148.21 ([M+H]$^+$, 18%), 107.2 (100).

Synthesis of *N*-allylaniline 40b[28] and *N,N*-diallylaniline 39b[29]

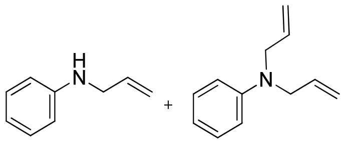

To DMF (25 mL) at 0 °C was added aniline (913 µL, 10 mmol, 1 equiv.), allyl bromide (950 µL, 11 mmol, 1.1 equiv.) and potassium carbonate (1.66 g, 12 mmol, 1.2 equiv.). The reaction mixture was allowed to come to room temperature and left stirring at room temperature for 24 h, and then quenched with water (50 mL). The aqueous fraction was extracted with diethyl ether (3 x 30 mL) and the combined organic fractions washed with additional water (4 x 25 mL) and brine (10 mL) then dried with anhydrous sodium sulfate, filtered and concentrated under reduced pressure. The resulting organic residue was purified by flash column chromatography (10-50 % ethyl acetate in petroleum ether) to yield *N*-allylaniline **40b**[28] and *N,N*-diallylaniline **39b**.[29] *N*-allylaniline **40b** was a pale yellow oil (480 mg, 36%); [Found: (HASP$^+$) (M+H)$^+$, 134.0962. $C_9H_{12}N^+$ (M+H), requires 134.0964]; ν_{max} (ATR)/cm^{-1} 3410, 3051, 2839, 1600, 1502, 1313, 1251, 991, 916, 746, 690; δ_H (400 MHz, $CDCl_3$) 3.81 (2H, dt, *J* = 5.4, 1.5 Hz, ArNC*H*$_2$) overlapping with bs (1H, 3.80-3.90, N*H*), 5.20 (1H, ddt, *J* = 10.4, 1.5, 1.5 Hz, NCH_2CHCH_2), 5.34 (1H, ddt, *J* = 17.2, 1.5, 1.5 Hz, NCH_2CHCH_2), 5.98 (1H, ddt, *J* = 17.2, 10.4, 5.4 Hz, NCH_2CH), 6.67 (2H, d, *J* = 8.0 Hz, Ar*H*), 6.75 (1H, t, *J* = 7.2 Hz, Ar*H*), 7.21 (2H, dd, *J* = 8.0, 7.2 Hz, Ar*H*) ; δ_C (100 MHz, $CDCl_3$) 46.1, 112.5, 115.7, 117.1, 128.8, 135.0, 147.6. *N,N*-diallylaniline **39b** was a pale yellow oil (918 mg, 53%); [Found: (HNESP$^+$) (M+H)$^+$, 174.1276. $C_{12}H_{16}N^+$ (M+H), requires 174.1277]; ν_{max} (ATR)/cm^{-1} 3078, 2860, 1597, 1502, 1230, 914, 744, 690; δ_H (400 MHz, $CDCl_3$) 3.95 (4H, ddd, *J* = 4.9, 1.8, 1.5 Hz, 2 x ArNC*H*$_2$), 5.17-5.24 (4H, m, NCH_2CHCH), 5.89 (2H, ddt, *J* = 17.2, 10.3, 4.9 Hz, NCH_2CH), 6.70-6.75 (3H, m, Ar*H*), 7.22-7.26 (2H, m, Ar*H*); δ_C (100 MHz, $CDCl_3$) 52.3, 111.9, 115.6, 115.9, 128.7, 133.6, 148.3.

Synthesis of *N*-allyl-*N*-phenylacetamide 39c[30]

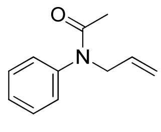

Sodium hydride (as a 60% suspension in mineral oil, 480 g, 12 mmol, 1.2 equiv.) was rinsed with dry hexane (3 x 30 mL) and blown dry under argon. To this was added dry DMF (10 mL) followed by a solution of *N*-phenylacetamide **40c** (1.49 g, 11 mmol, 1.1 equiv.) in dry DMF (10 mL). Allyl bromide (864 µl, 10 mmol, 1 equiv.) was added to the mixture which was then stirred overnight at room temperature. The reaction mixture was poured into 2N aqueous NaOH (30 mL) and diethyl ether (50 mL) and extracted with further diethyl ether (3 x 25 mL). This solution was then sequentially washed with 2N aqueous NaOH (2 x 25 mL), water (50 mL) and brine (25 mL), dried over sodium sulfate, filtered and evaporated under reduced pressure. The resulting precipitate was purified by flash column chromatography (25 % ethyl acetate in petroleum ether) to afford *N*-allyl-*N*-phenylacetamide **39c**[30] as a pale yellow oil (1.17 g, 67%); [Found: (HNESP$^+$) (M+H)$^+$, 176.1070. $C_{11}H_{14}NO^+$ (M+H), requires 176.1070]; ν_{max} (ATR)/cm^{-1} 2905, 1656, 1494, 1382, 1274, 920, 698; δ_H (400 MHz, $CDCl_3$) 1.86 (3H, s, C(O)C*H*$_3$), 4.29-4.31 (2H, m, NC*H*$_2$), 5.04-5.12 (2H, m, NCH_2CHC*H*), 5.86 (1H, ddt, *J* = 16.9, 10.4, 6.3 Hz, NCH_2C*H*), 7.15-7.17 (2H, m, Ar*H*), 7.32-7.42 (3H, m, Ar*H*); δ_C (100 MHz, $CDCl_3$) 22.2, 51.5, 117.3, 127.4, 127.6, 129.1, 132.7, 142.5, 169.7.

Synthesis of *N*-allyl-*N*-phenylpivalamide 39d[31]

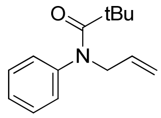

To a solution of *N*-allylaniline **40b** (266 mg, 2 mmol, 1 equiv.) and triethylamine (418 µL, 3 mmol, 1.5 equiv.) in dichloromethane (10 mL) was added pivaloyl chloride (271 µL, 2.2 mmol, 1.1 equiv.), slowly at 0 °C. The solution was then stirred at room temperature overnight before quenching with 2N aqueous HCl (25 mL). The aqueous fraction was extracted with further dichloromethane (3 x 10 mL) and the combined organic fractions washed with additional aqueous HCl (2 x 20 mL), water (20 mL) and brine (10 mL) then dried with anhydrous sodium sulfate, filtered and concentrated under reduced pressure. The resultant pale yellow oil was purified by flash column chromatography (10 % ethyl acetate in petroleum ether) to yield *N*-allyl-*N*-phenylpivalamide **39d**[31] (254 mg, 58%); [Found: (HNESP$^+$) (M+H)$^+$, 218.1537. $C_{14}H_{20}NO^+$ (M+H), requires 218.1539]; ν_{max} (ATR)/cm^{-1} 2956, 1631, 1593, 1494, 1195, 920, 702; δ_H (400 MHz, $CDCl_3$) 1.00 (9H, s, C*H*$_3$), 4.16 (2H, ddd, *J* = 6.3, 1.1, 1.1 Hz, NC*H*$_2$), 4.96 (1H, ddt, *J* = 17.1, 1.1, 1.1 Hz, NCH_2CHC*H*), 5.04 (1H, ddt, *J* = 10.2, 1.1, 1.1 Hz, NCH_2CHC*H*), 5.88 (1H, ddt, *J* = 17.1, 10.2, 6.3 Hz, NCH_2C*H*), 7.12-7.16 (2H, m, Ar*H*), 7.16-7.33 (3H, m, Ar*H*); δ_C (100 MHz, $CDCl_3$) 28.8, 40.2, 55.2, 116.9, 127.2, 128.3, 129.1, 132.7, 142.8, 176.4.

Synthesis of ethyl phenylcarbamate 40e[32]

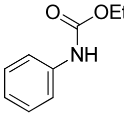

To a solution of aniline (1.00 mL, 11 mmol, 1.1 equiv.) and ethyl chloroformate (952 µL, 10 mmol, 1 equiv.) in chloroform (20 mL) was added potassium carbonate (2.76 g, 20 mmol, 2 equiv.). The solution was then stirred at reflux overnight before allowing to cool and quenching with water (25 mL). The aqueous fraction was extracted with dichloromethane (3 x 20 mL) and the combined organic fractions washed with additional aqueous HCl (2 x 20 mL), water (20 mL) and brine (10 mL), and then dried with anhydrous sodium sulfate, filtered and concentrated under reduced pressure. The precipitate was recrystallized (hot ethanol) to yield ethyl phenylcarbamate **40e** as pale pink crystals (1.62 g, 98%); m.p. 48-50 °C [lit.[32] m.p. 48-50 °C]; [Found: (HNESP$^+$) (M+H)$^+$, 166.0858. $C_9H_{12}NO_2^+$ (M+H), requires 166.0863]; ν_{max} (ATR)/cm^{-1} 3315, 2981, 1701, 1597, 1529, 1440, 1226, 1058, 900, 740, 692; δ_H (400 MHz, $CDCl_3$) 1.33 (3H, t, *J* = 7.1 Hz, OCH_2CH_3), 4.25 (2H, q, *J* = 7.1 Hz, OCH_2CH_3), 6.70-6.81 (1H, bs, N*H*), 7.05-7.10 (1H, m, Ar*H*), 7.29-7.35 (2H, m, Ar*H*), 7.40-7.43 (2H, m, Ar*H*); δ_C (100 MHz, $CDCl_3$) 14.1, 60.7, 118.2, 122.8, 128.5, 137.5, 153.2; *m*/*z* (APCI$^+$) 165.2 ([M+H]$^+$, 100%).

Synthesis of ethyl allyl(phenyl)carbamate 39e[33]

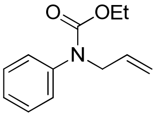

Sodium hydride (as a 60% suspension in mineral oil, 240 mg, 6 mmol, 1.2 equiv.) was rinsed with dry hexane (3 x 20 mL) and blown dry under argon. To this was added dry THF (20 mL) followed by a solution of ethyl phenylcarbamate **40e** (825 mg, 5 mmol, 1.1 equiv.) in dry THF (10 mL). To the resultant solution was slowly added allyl bromide (648 µL, 7.5 mmol, 1.5 equiv.). The solution was then stirred at room temperature overnight before quenching with water (35 mL). The aqueous fraction was extracted with further diethyl ether (3 x 10 mL) and the combined organic fractions washed with additional water (3 x 20 mL) and brine (10 mL) then dried with anhydrous sodium sulfate, filtered and concentrated under reduced pressure. The resultant pale yellow oil was purified by flash column chromatography (5 % ethyl acetate in petroleum ether) to yield ethyl allyl(phenyl)carbamate **39e**[33] (780 mg, 76%); ν_{max} (ATR)/cm^{-1} 2980, 1697, 1597, 1496, 1377, 1226, 1145, 1020, 921, 765, 696; δ_H (400 MHz, $CDCl_3$) 1.25 (3H, t, *J* = 7.1 Hz, OCH_2CH_3), 4.19 (2H, q, *J* = 7.1 Hz, OCH_2CH_3) 4.28 (2H, ddd, *J* = 5.8, 1.5, 1.3, NCH_2), 5.14-5.20 (2H, m, NCH_2CHCH_2), 5.89-5.99 (1H, m, NCH_2CH), 7.22-7.26 (3H, m, Ar*H*), 7.34-7.38 (2H, m, Ar*H*); δ_C (100 MHz, $CDCl_3$) 14.1, 52.7, 61.2, 116.4, 125.7, 126.2, 128.3, 113.4, 141.8, 155.0; *m*/*z* (MM-ES+APCI$^+$) 206.1 ([M+H]$^+$, 17%), 178.1 (34), 134.2 (100).

Synthesis of ethyl 2-[methyl(phenyl)amino]acetate 41a[34]

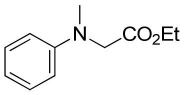

To a solution of *N*-methylaniline **40a** (1.08 mL, 10 mmol, 1 equiv.) in ethanol (20 mL) was added sodium carbonate (1.59 g, 15 mmol, 1.5 equiv.) and ethyl bromoacetate (1.22 mL, 11 mmol, 1.1 eq) and the suspension stirred at reflux for 14 h. The reaction was quenched with water (50 mL) and extracted with ethyl acetate (4 x 20 mL). The combined organic fractions were washed with water (4 x 25 mL) and brine (20 mL) and then dried with anhydrous sodium sulfate, filtered and concentrated under vacuum. Purification over silica (5% ethyl acetate/ petroleum ether) afforded ethyl 2-(methyl(phenyl)amino)acetate **41a** as a colourless oil (1.91 g, 99%); ν_{max} (ATR)/cm^{-1} 2980, 2899, 1743, 1598, 1504, 1365, 1184, 1026, 945, 746, 688; δ_H (400 MHz, $CDCl_3$) 1.29 (3H, t, *J* = 7.1 Hz, OCH_2CH_3), 3.11 (3H, s, NCH_3), 4.10 (2H, s, NCH_2), 4.22 (2H, q, *J* = 7.1 Hz, OCH_2CH_3), 6.73-6.76 (2H, m, Ar*H*), 6.76-6.82 (1H, m, Ar*H*), 7.27-7.31 (2H, m, Ar*H*); δ_C (100 MHz, $CDCl_3$) 13.8, 39.0, 54.0, 60.4, 111.8, 116.8, 128.7, 128.4, 170.6; *m/z* (MM-ES+APCI$^+$) 194.2 ($[M+H]^+$, 81%), 120.2 (100).

Synthesis of ethyl 2-(*N*-phenylacetamido)acetate 41b[35]

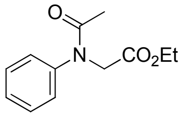

Sodium hydride (as a 60% suspension in mineral oil, 480 g, 12 mmol, 1.2 equiv.) was rinsed with dry hexane 3 x 30 mL and blown dry under argon. To this was added dry DMF (10 mL) followed by a solution of *N*-phenylacetamide **40c** (1.49 g, 11 mmol, 1.1 equiv.) in dry DMF (10 mL). Ethyl bromoacetate (1.11 mL, 10 mmol, 1 equiv.) was added to the mixture which was then stirred overnight at room temperature. The reaction mixture was poured into 2N aqueous NaOH (30 mL) and diethyl ether (50 mL) and extracted with further diethyl ether (3 x 25 mL). This solution was then sequentially washed with 2N aqueous NaOH (2 x 25 mL), water (50 mL) and brine (25 mL), dried over sodium sulfate, filtered and evaporated under reduced pressure. The resulting precipitate was purified over silica (25 % ethyl acetate in petroleum ether) to afford ethyl 2-(*N*-phenylacetamido)acetate **41b** as a pale yellow oil (1.61 g, 66%); [Found: (HNESP$^+$) $(M+H)^+$, 222.1127. $C_{12}H_{16}NO_3^+$ (M+H), requires 222.1125]; ν_{max} (ATR)/cm^{-1} 2981, 1745, 1664, 1375, 1190, 1020, 698; δ_H (400 MHz, $CDCl_3$) 1.29 (3H, t, *J* = 7.1 Hz, OCH_2CH_3), 1.94 (3H, s, $C(O)CH_3$), 4.22 (2H, q, *J* = 7.1 Hz, OCH_2CH_3), 4.39 (2H, s, NCH_2), 7.35-7.46 (5H, m, Ar*H*); δ_C (100 MHz, $CDCl_3$) 13.6, 21.6, 50.7, 60.7, 127.4, 127.7, 129.2, 142.9, 168.7, 170.4.

Synthesis of ethyl 2-((ethoxycarbonyl)(phenyl)amino)acetate 41c[36]

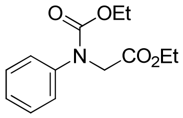

Sodium hydride (as a 60% suspension in mineral oil, 200 mg, 5 mmol, 1 equiv.) was rinsed with dry hexane 3 x 20 mL and blown dry under argon. To this was added dry THF (10 mL) followed by a solution of ethyl phenylcarbamate **40e** (826 mg, 5 mmol, 1 equiv.) in dry THF (10 mL). Ethyl bromoacetate (553 µl, 5 mmol, 1 equiv.) was dissolved in dry THF (10 mL) and added to the mixture which was then stirred overnight at room temperature. The reaction mixture was poured into water (40 mL) and ethyl acetate (40 mL) and extracted with further ethyl acetate (3 x 40 mL). This solution was then sequentially washed with water (3 x 30 mL), dried over sodium sulfate, filtered and evaporated under reduced pressure. The resulting precipitate was eluted over silica in 60-80 % ethyl acetate in petroleum ether to afford ethyl 2-((ethoxycarbonyl)(phenyl)amino)acetate **41c** (1.17 g, 93%); [Found: ($HNESP^+$) $(M+H)^+$, 252.1225. $C_{13}H_{18}NO_4^+$ (M+H), requires 252.1230]; ν_{max} (ATR)/cm^{-1} 2980, 1749, 1699, 1597, 1375, 1193, 1024, 767, 696; δ_H (400 MHz, $CDCl_3$) 1.24 (3H, bs, OCH_2CH_3), 1.30 (3H, J = 7.0 Hz, $OCH_2C\mathit{H}_3$), 4.15-4.27 (2H, m, $OC\mathit{H}_2CH_3$), overlapping with 4.22 (2H, q, J = 7.0 Hz, $OC\mathit{H}_2CH_3$), 4.35 (2H, s, $NC\mathit{H}_2$), 7.24-7.39 (5H, m, Ar*H*); δ_C (100 MHz, $CDCl_3$) 13.7, 14.0, 51.8, 60.7, 61.6, 126.2, 128.4, 141.6 (br.), 155.1 (br.), 169.1.

Synthesis of ethyl 2-(4-methyl-*N*-phenylphenylsulfonamido)acetate 41d

i) Synthesis of 4-methyl-*N*-phenylbenzenesulfonamide

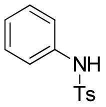

To a solution of aniline (1.86 g, 20 mmol, 1 equiv.) and pyridine (1.90 g, 24 mmol, 1.2 equiv.) in DCM (15 mL) was added toluenesulfonyl chloride (4.19 g, 22 mmol, 1.1 equiv.) portion-wise with stirring. The reaction mixture was then stirred at room temperature for 12 h before evaporation of the DCM and quenching with NaOH (aqueous, 2N, 50 mL). The aqueous solution was rinsed with diethyl ether (2 x 25 mL) then acidified with conc. HCl and extracted with DCM (3x 25 mL). The resulting organic washings were dried over sodium sulfate and concentrated in vacuo to yield an off white solid. Recrystallization from chloroform/hexane gave rise to 4-methyl-*N*-phenylbenzenesulfonamide as a white crystalline solid (4.85 g, 98%); m.p. 99-101 °C [lit.[37] 102-103 °C], ν_{max} (ATR)/cm^{-1} 3235, 3028, 2897, 1597, 1481, 1416, 1335, 1153, 1090, 908, 752, 709, 694; δ_H (400 MHz, $CDCl_3$) 2.40 (3H, s, $ArC\mathit{H}_3$), 6.65 (1H, s, N*H*), 7.07-7.15 (3H, m, Ar*H*), 7.23-7.29 (4H, m, Ar*H*); 7.66-7.69 (2H, m, Ar*H*); δ_C (100 MHz, $CDCl_3$) 21.0, 121.2, 124.9, 126.8, 128.8, 129.1, 135.6, 136.0, 143.4; *m*/*z* (MM-ES+$APCI^-$) 246.1 ($[M-H]^-$, 100%).

ii) Synthesis of ethyl 2-(4-methyl-*N*-phenylphenylsulfonamido)acetate 41d

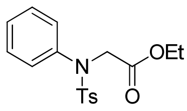

To sodium hydride (as a 60% suspension in mineral oil, 480 mg, 12 mmol, 1.2 equiv.) dispersed in dry THF (20 mL) was added a solution of 4-methyl-*N*-phenylbenzenesulfonamide (2.47 g, 10 mmol, 1 equiv.) in dry THF (15 mL) under argon. A dense slurry formed which did not disperse upon addition of more dry THF (20 mL) so dry DMF (50 mL) was added and stirred for 1 h before addition of ethyl bromoacetate (1.66 ml, 15 mmol, 1.5 equiv.) then stirring overnight at room temperature. The reaction mixture was quenched with HCl (aqueous, 2N, 100 mL) and diethyl ether (100 mL) then extracted with further ether (50 mL). This solution was then sequentially washed with HCl (aqueous, 2N, 50 mL), water (2 x 50 mL), brine (25 mL), dried over sodium sulfate, filtered and evaporated under reduced pressure. The resulting precipitate was recrystallized from ethyl acetate/hexane to afford ethyl 2-(4-methyl-*N*-phenylphenylsulfonamido)acetate **41d** as colourless cubes (2.80 g, 84%); m.p. 103-105 ^{o}C [lit.[35] 108-109 ^{o}C], ν_{max} (ATR)/cm^{-1} 2976, 2941, 1746, 1491, 1337, 1153, 1128, 881, 862, 740, 694; δ_H (400 MHz, $CDCl_3$) 1.24 (3H, t, *J* = 7.2 Hz, OCH_2CH_3), 2.44 (3H, s, $ArCH_3$), 4.16 (2H, q, *J* = 7.2 Hz, OCH_2CH_3), 4.42 (2H, s, NCH_2), 7.20-7.32 (7H, m, Ar*H*), 7.5. (2H, d, *J* = 8.3 Hz, Ar*H*); δ_C (100 MHz, $CDCl_3$) 13.5, 21.1, 52.3, 60.9, 127.3, 127.6, 128.2, 128.7, 128.9, 135.5, 139.4, 143.1, 168.2; *m*/*z* (MM-ES+APCI$^+$) 334.1 ([M+H]$^+$, 4%), 260.0 (7), 179.1 (100), 106.1 (37).

Ethyl 2-((2-(cyclohex-1-en-1-yl)ethyl)amino)acetate

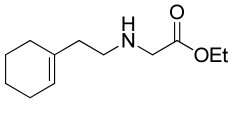

To a solution of 2-(cyclohex-1-en-1-yl)ethanamine (2.50 g, 20 mmol, 1 equiv.) in chloroform (25 mL) was added triethylamine (4.18 mL, 30 mmol, 1.5 equiv.) followed slowly by ethyl bromoacetate (2.21 mL, 20 mmol, 1 equiv.). The reaction mixture was stirred overnight at reflux then poured into water (50 ml) and rinsed with chloroform (3 x 30 mL). This solution was then sequentially rinsed with water (2 x 30 mL) and brine (2 x 30 mL), dried over sodium sulfate, filtered and evaporated under reduced pressure. The residue was eluted over silica (10 % diethyl ether in dichloromethane) to afford *ethyl 2-((2-(cyclohex-1-en-1-yl)ethyl)amino)acetate* as a colourless oil (3.71 g, 88%); [Found: (HNESP$^+$) (M+H)$^+$, 212.1646. $C_{12}H_{22}NO_2^+$ (M+H), requires 212.1645]; ν_{max} (ATR)/cm^{-1} 2922, 1735, 1436, 1369, 1182, 1026, 918, 742; δ_H (400 MHz, $CDCl_3$) 1.24 (3H, t, *J* = 7.1 Hz, OCH_2CH_3), 1.50-1.67 (4H, m, cyclohexyl), 1.88-1.97 (4H, m, cyclohexyl), 2.65 (2H, t, *J* = 6.8 Hz, NCH_2CH_2), 3.36 (2H, s, NCH_2COOEt), 4.14 (2H, q, *J* = 7.1 Hz, OCH_2CH_3), 5.41-5.46 (1H, m, C=C*H*); δ_C (100 MHz, $CDCl_3$)

13.7, 21.9, 22.4, 24.7, 27.5, 37.9, 46.7, 50.4, 60.0, 122.3, 134.6, 171.9; *m*/*z* (MM-ES+APCI$^+$) 212.1 ([M+H]$^+$, 28%), 116.2 (100).

Ethyl 2-(*N*-(2-(cyclohex-1-en-1-yl)ethyl)acetamido)acetate 43

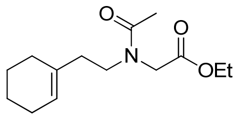

To a solution of ethyl 2-((2-(cyclohex-1-en-1-yl)ethyl)amino)acetate (2.13 g, 10 mmol, 1 equiv.) in chloroform (10 mL) was added imidazole (817 mg, 12 mmol, 1.2 equiv.) followed slowly by acetyl chloride (0.785 mL, 11 mmol, 1.1 equiv.). The reaction mixture was stirred overnight at reflux then poured into water (25 mL) and rinsed with chloroform (3 x 25 mL). This solution was then sequentially rinsed with water (25 mL) and brine (25 mL), dried over sodium sulfate, filtered and evaporated under reduced pressure. The residue was eluted over silica (10 % diethyl ether in dichloromethane) to afford *ethyl 2-(N-(2-(cyclohex-1-en-1-yl)ethyl)acetamido)acetate* **43** as a colourless oil (1.64 g, 65%); [Found: (HNESP$^+$) (M+H)$^+$, 254.1753. $C_{14}H_{24}NO_3^+$ (M+H), requires 254.1751]; ν_{max} (ATR)/cm^{-1} 2984, 2929, 2835, 1743, 1649, 1425, 1182, 1026, 920, 802, 711; δ_H (500 MHz, $CDCl_3$) [two isomers in a ratio of 1:0.3] 1.29 (3H, t *J* = 7.1 Hz, OCH_2CH_3, major isomer), 1.32 (0.9H, t *J* = 7.1 Hz, OCH_2CH_3, minor isomer), 1.62-1.67 (5.2H, m, CH_2CH_2, major and minor isomers), 2.01 (0.9H, s, NC(O)CH_3, minor isomer), 2.14 (3H, s, NC(O)CH_3, major isomer), 2.11-2.20 (5.2H, m, CH_2C=CCH_2, major and minor isomers), 3.36-3.01 (2H, m, NCH_2CH_2, major isomer), 3.41-3.47 (2H, m, NCH_2CH_2, minor isomer), 4.98 (0.6H, s, NCH_2CO_2Et, minor isomer), 4.04 (2H, s, NCH_2CO_2Et, major isomer), 4.17 (2H, q, *J* = 7.1 Hz, OCH_2CH_3, major isomer), 4.21 (0.6H, q, *J* = 7.1 Hz, OCH_2CH_3, minor isomer), 5.42-5.46 (0.3H, m, C=C*H*, minor isomer), 5.46-5.53 (1H, m, C=C*H*, major isomer); δ_C (125 MHz, $CDCl_3$) [two isomers] 14.1, 20.9, 21.6, 22.2, 22.3, 22.8, 22.9, 25.2, 25.3, 28.3, 28.5, 35.7, 36.9, 45.8, 47.5, 48.8, 50.7, 61.1, 61.6, 123.0, 124.0, 133.8, 134.8, 169.3, 169.5, 170.6, 170.9; *m*/*z* (MM-ES+APCI$^+$) 254.2 ([M+H]$^+$, 100%).

Methyl 1-phenylpyrrolidine-2-carboxylate 44[38]

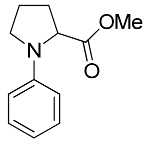

Under a flow of argon CuI (200 mg, 1 mmol, 0.1 equiv.) and potassium carbonate (4 g, 20 mmol, 2 eqiv.) were added to a solution of L-proline (1.14 g, 10 mmol, 1 equiv.) and bromobenzene (1.2 mL, 11.5 mmol, 1.15 equiv.) in DMF (10 mL). The reaction mixture was stirred overnight at 100 ^oC then allowed to cool to room temperature, diluted with additional DMF (10 mL) and dimethyl sulfate (1.5

mL, 15 mmol, 1.5 equiv.) added. The reaction mixture was stirred for a further 20 h then the DMF removed by vacuum distillation. The resulting residue was dissolved in ethyl acetate (50 mL) and water (50 mL) and extracted with further ethyl acetate (2 x 50 mL). The combined organic layers were washed with water (3 x 25 mL) and brine (25 mL), dried over sodium sulfate, filtered and evaporated under reduced pressure. The residue was eluted over a short column of silica (10 % ethyl acetate in petroleum ether) to afford methyl 1-phenylpyrrolidine-2-carboxylate **44** as a colourless oil (1.42 g, 69%); ν_{max} (ATR)/cm^{-1} 2957, 2841, 1729, 1597, 1504, 1361, 1343, 1272, 1188, 1154, 992, 743, δ_H (400 MHz, $CDCl_3$) 2.12-2.37 (4H, m, CH_2CH_2), 3.75 (3H, s, OCH_3), 4.29 (1H, dd, *J* = 8.5, 2.1 Hz, NC*H*CO_2Me), 6.58 (2H, d, *J* = 8.5 Hz, Ar*H*), 6.76 (1H, t, *J* = 7.2 Hz, Ar*H*), 7.26 (2H, dd, *J* = 8.5, 7.2 Hz, Ar*H*); δ_C (100 MHz, $CDCl_3$) 23.4, 30.4, 47.8, 51.6, 60.3, 111.5, 116.2, 128.8, 146.2, 174.5; *m*/*z* (MM-ES+APCI$^+$) 206.1 ([M+H]$^+$, 54%), 146.1 (100).

Reaction of *N*-allyl-*N*-methylaniline 39a with photoactivated donor 3

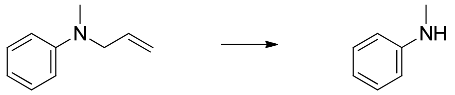

N-allyl-*N*-methylaniline (44 mg, 0.3 mmol, 1 equiv.) was reacted according to general procedure C to afford *N*-methylaniline **40a**[39] (2 mg, 6 %) and recovery of *N*-allyl-*N*-methylaniline **39a** as a colourless oil (27 mg, 62 %) with data as recorded above for this compound. *N*-methylaniline **40a** was a pale brown oil; ν_{max} (ATR)/cm^{-1} 3412, 3049, 2879, 2812, 1600, 1504, 1317, 1261, 1178, 1151, 1070, 857, 746, 690; δ_H (400 MHz, $CDCl_3$) 2.89 (3H, s, NCH_3), 3.50-3.71 (1H, bs, N*H*), 6.66-6.70 (2H, m, Ar*H*), 6.79 (1H, td, *J* = 7.2, 1.1 Hz, Ar*H*), 7.25-7.29 (2H, m, Ar*H*); δ_C (100 MHz, $CDCl_3$) 30.2, 111.9, 116.8, 128.7, 148.9; *m*/*z* (MM-ES+APCI$^+$) 108.2 ([M+H]$^+$, 100%).

Reaction of *N*,*N*-diallylaniline 39b with photoactivated donor 3

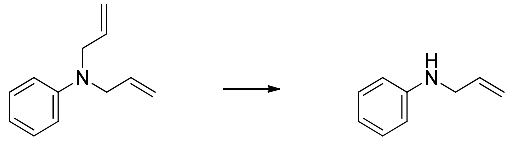

N,*N*-diallylaniline **39b** (52 mg, 0.3 mmol, 1 equiv.) was reacted according to general procedure C to afford *N*-allylaniline **40b** as a pale yellow oil (9 mg, 7 %) and recovered *N*,*N*-diallylaniline **39b** as a colourless oil (42 mg, 81 %) with data as reported above for this compound.

Reaction of *N*-allyl-*N*-phenylacetamide 39c with photoactivated donor 3

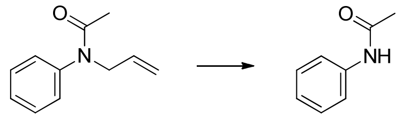

N-allyl-*N*-phenylacetamide **39c** (53 mg, 0.3 mmol, 1 equiv.) was reacted according to general procedure C to afford *N*-phenylacetamide **40c** (13 mg, 33%) and *N*-allyl-*N*-phenylacetamide **39c** (31 mg, 59%). *N*-phenylacetamide **40c**[40] was a white solid; m.p. 103-105 °C (lit.[40] 113-114 °C); ν_{max} (ATR)/cm^{-1} 3290, 1661, 1597, 1431, 1319, 1261, 750, 692; δ_H (400 MHz, $CDCl_3$) 2.18 (3H, s, C(O)C*H*$_3$), 7.12 (1H, t, *J* = 7.4 Hz, Ar*H*), 7.31 (2H, dd, *J* = 7.6, 7.4 Hz, Ar*H*), 7.52 (2H, d, *J* = 7.6 Hz, Ar*H*); 7.68-7.78 (1H, bs, N*H*); δ_C (100 MHz, $CDCl_3$) 24.0, 119.5, 123.8, 128.5, 137.5, 168.0; *m*/*z* (MM-ES+APCI$^+$) 136.1 ([M+H]$^+$, 100%).

Reaction of *N*-allyl-*N*-phenylacetamide 39c with donor 3 in the absence of UV light

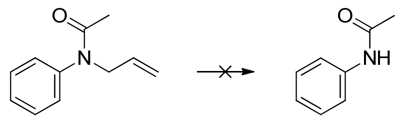

N-allyl-*N*-phenylacetamide **39c** (53 mg, 0.3 mmol, 1 equiv.) was reacted according to general procedure C with the omission of photoactivation to afford recovery of *N*-allyl-*N*-phenylacetamide **39c** (53 mg, 100%).

Reaction of *N*-allyl-*N*-phenylacetamide 39c with photoactivated donor: isotopic labelling reaction

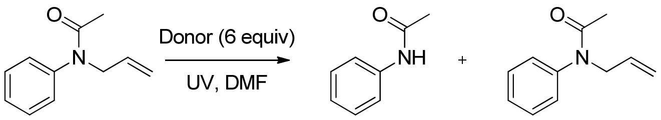

N-allyl-*N*-phenylacetamide **39c** (53 mg, 0.3 mmol, 1 equiv.) was reacted under standard conditions with donor 9 (512 mg, 1.8 mmol, 6 equiv.). When degassed D_2O (1 mL) was added to the sealed reaction mixture prior to work up, the isolated products (N-phenylacetamide **40c** (15 mg, 37%) and *N*-allyl-*N*-phenylacetamide **39c** (32 mg, 60%)) showed enrichment of the deuterated product by GC-MS and deuterium NMR:

Deuterium spectra of the N-phenylacetamide **40c** and *N*-allyl-*N*-phenylacetamide **39c** showed peaks at 2.18 (compared with 2.20) and 1.87 (compared with 1.88) respectively, corresponding to the peaks in the ^{1}H spectra.

GCMS (CI$^+$, CH_4) spectra of the N-phenylacetamide **40c** and *N*-allyl-*N*-phenylacetamide **39c** showed deuterium incorporation of 17.5 and 35% respectively.

Reaction of *N*-allyl-*N*-phenylpivalamide 39d with photoactivated donor 3

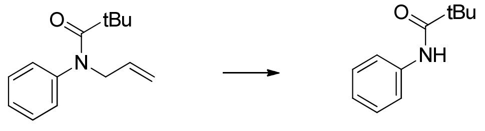

N-allyl-*N*-phenylpivalamide **39d** (65 mg, 0.3 mmol, 1 equiv.) was reacted according to general procedure C to afford *N*-phenylpivalamide **40d** as a white solid (44 mg, 83 %) and *N*-allyl-*N*-phenylpivalamide **39d**[31] (5 mg, 8 %) with data matching those reported above for this compound.

N-phenylpivalamide **40d**[41]; m.p. 122-124 °C [lit.[41] m.p. 121-124 °C]; ν_{max} (ATR)/cm^{-1} 3311, 2964, 1653, 1595, 1529, 1435, 1315, 1240, 1168, 927, 902, 752, 694; δ_H (400 MHz, $CDCl_3$) 1.34 (9H, s, C(O)CCH_3), 7.12 (1H, t, J = 7.4 Hz, Ar*H*), 7.34 (2H, dd, J = 7.4, 7.4 Hz, Ar*H*), overlapping with 7.32-7.40 (1H, bs, N*H*), 7.55 (2H, d, J = 7.4 Hz, Ar*H*); δ_C (100 MHz, $CDCl_3$) 27.1, 39.1, 119.5, 123.7, 128.4, 137.6, 176.1; *m/z* (MM-ES+APCI$^+$) 178.1 ([M+H]$^+$, 100%).

Reaction of *N*-allyl-*N*-phenylpivalamide 39d with photoactivated donor

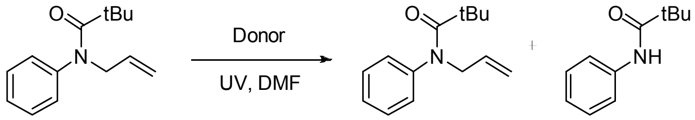

In a nitrogen glovebox, to *N*-allyl-*N*-phenylpivalamide **39d** (65 mg, 0.3 mmol, 1 equiv.) in dry DMF (4 mL) was added donor 9 (512 mg, 1.8 mmol, 6 equiv.). The reaction flask was sealed and removed to a fumehood where it was reacted for 72 h under UV light photoactivation. The reaction was then returned to the glovebox and a further portion of donor 9 (512 mg, 1.8 mmol, 6 equiv.) added before once more removing to a fumehood and reacting or 72 h under UV light photoactivation. Standard acidic work-up procedure afforded *N*-phenylpivalamide **40d** (53 mg, >99%). with data as reported above.

Reaction of *N*-allyl-*N*-phenylpivalamide 39d with photoactivated donor

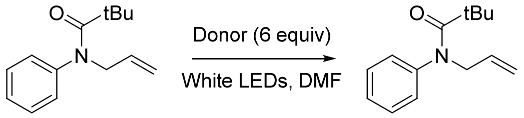

N-allyl-*N*-phenylpivalamide **39d** (65 mg, 0.3 mmol, 1 equiv.) was reacted under standard conditions with donor 9 (512 mg, 1.8 mmol, 6 equiv.) under white LED light photoactivation to afford recovery of *N*-allyl-*N*-phenylpivalamide **39d** (62 mg, 96%). Data were as reported previously.

Reaction of ethyl allyl(phenyl)carbamate 39e with photoactivated donor 3

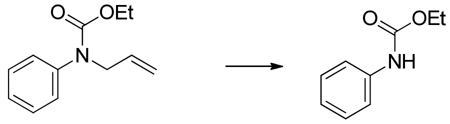

Ethyl allyl(phenyl)carbamate **39e** (62 mg, 0.3 mmol, 1 equiv.) was reacted according to general procedure C to afford ethyl phenylcarbamate **40e** as a colourless solid (39 mg, 58 %) and recovered

ethyl allyl(phenyl)carbamate **39e** as a colourless solid (23 mg, 37 %) with data matching those reported previously.

Reaction of ethyl 2-(methyl(phenyl)amino)acetate 41a with photoactivated donor 3

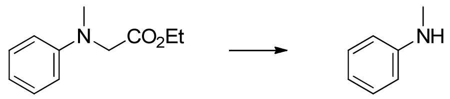

2-(Methyl(phenyl)amino)acetate **41a** (58 mg, 0.3 mmol, 1 equiv.) was reacted according to general procedure C to afford *N*-methylaniline **40a** as a pale yellow oil (11 mg, 34 %) and recovered 2-(methyl(phenyl)amino)acetate **41a** as a colourless oil (34 mg, 58 %).

Reaction of ethyl 2-(*N*-phenylacetamido)acetate 41b with photoactivated donor 3

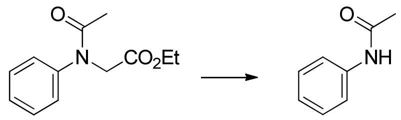

Ethyl 2-(*N*-phenylacetamido)acetate **41b** (66 mg, 0.3 mmol, 1 equiv.) was reacted according to general procedure C to afford *N*-phenylacetamide **40c** (30 mg, 74 %) and recovered ethyl 2-(*N*-phenylacetamido)acetate **41b** (17 mg, 25 %) with data as reported previously.

Reaction of ethyl 2-(*N*-phenylacetamido)acetate 41b with donor 3 in the absence of UV light

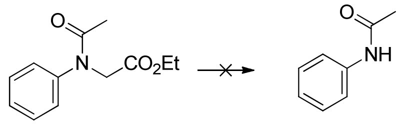

Ethyl 2-(*N*-phenylacetamido)acetate **41b** (66 mg, 0.3 mmol, 1 equiv.) was reacted according to general procedure C with the omission of UV activation to afford recovery of ethyl 2-(*N*-phenylacetamido)acetate **41b** (60 mg, 91 %).

Reaction of ethyl 2-((ethoxycarbonyl)(phenyl)amino)acetate 41c with photoactivated donor 3

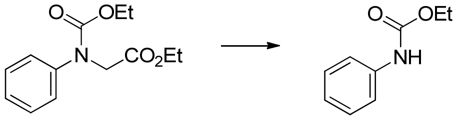

Ethyl 2-((ethoxycarbonyl)(phenyl)amino)acetate **41c** (75 mg, 0.3 mmol, 1 equiv.) was reacted according to general procedure C to afford ethyl phenylcarbamate **40e** as a colourless solid (46 mg, 92 %) with data as reported previously.

Reaction of ethyl 2-(4-methyl-*N*-phenylphenylsulfonamido)acetate 41d with photoactivated donor

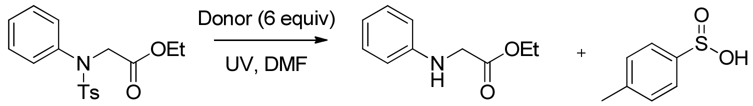

Ethyl 2-(4-methyl-*N*-phenylphenylsulfonamido)acetate (100 mg, 0.3 mmol, 1 equiv.) was reacted under standard conditions with donor 9 (512 mg, 1.8 mmol, 6 equiv.) to afford ethyl 2-(phenylamino)acetate **42** (48 mg, 89%) and 4-methylbenzenesulfinic acid (39 mg, 83%).

Ethyl 2-(phenylamino)acetate **42**[42] as an off-white, waxy solid; m.p. 54-56 °C [lit.[42] 57 °C]; ν_{max} (ATR)/cm^{-1} 3397, 3053, 2980, 2934, 1732, 1602, 1506, 1445, 1371, 1314, 1256, 1204, 1179, 1142, 1022, 748, 692; δ_H (400 MHz, $CDCl_3$) 1.32 (3H, t, *J* = 7.2 Hz, OCH_2CH_3), 3.93 (2H, s, NCH_2), 4.27 (2H, q, *J* = 7.2 Hz, OCH_2CH_3), 6.63-6.65 (2H, m, Ar*H*), 6.77-6.80 (1H, m, Ar*H*), 7.20-7.24 (2H, m, Ar*H*); δ_C (100 MHz, $CDCl_3$) 13.7, 45.4, 60.8, 112.5, 117.7, 128.8, 146.5, 170.6; *m*/*z* (GCMS, CI^+, CH_4) 208.1 ($[M+C_2H_5^+]$, 8%), 180.8 ($[M+H]^+$, 75), 134.0 (45), 106.0 (100).

4-Methylbenzenesulfinic acid **23**[43] was isolated as a pale yellow crystalline solid; m.p. 81-83 °C [lit.[43] 81 °C], ν_{max} (ATR)/cm^{-1} 2793, 2432, 1593, 1491, 1323, 1091, 1061, 1024, 1009, 808; δ_H (400 MHz, $CDCl_3$) 2.44 (3H, s, $ArCH_3$), 5.57-5.72 (1H, bs, SO_2H), 7.32 (2H, d, *J* = 8.1 Hz, Ar*H*), 7.60 (1H, d, *J* = 8.1 Hz, Ar*H*) ; δ_C (100 MHz, $CDCl_3$) 21.0, 124.5, 129.3, 142.2, 142.7; *m*/*z* (GCMS, CI^+, CH_4) 155.1 ($[M-H]^-$, 100%), 107.1 (7).

Reaction of ethyl 2-(*N*-(2-(cyclohex-1-en-1-yl)ethyl)acetamido)acetate 43 with donor 3

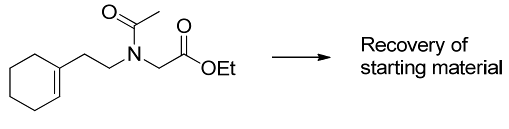

Ethyl 2-(*N*-(2-(cyclohex-1-en-1-yl)ethyl)acetamido)acetate **43** (76 mg, 0.3 mmol), was reacted according to procedure C to afford recovered ethyl 2-(*N*-(2-(cyclohex-1-en-1-yl)ethyl)acetamido)acetate **43** as a pale yellow oil (68 mg, 89%) with data matching those reported previously.

Reaction of methyl 1-phenylpyrrolidine-2-carboxylate 44 with donor 3

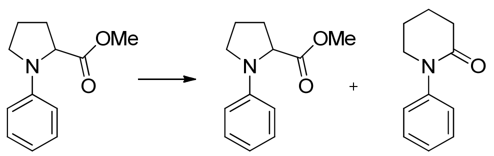

Methyl 1-phenylpyrrolidine-2-carboxylate **44** (62 mg, 0.3 mmol), was reacted according to procedure C to afford 1-phenylpiperidin-2-one **45**[44] as a white solid (16 mg, 30%); m.p. 100-101 °C (lit.[44] 100-101 °C); ν_{max} (ATR)/cm^{-1} 2956, 2916, 2851, 1638, 1487, 1427, 1304, 1161, 1069, 972, 825, 762; δ_H (400 MHz, $CDCl_3$) 1.92-2.04 (4H, m, $NCH_2(CH_2)_2CH_2$), 2.56-2.65 (2H, m, NCH_2), 3.65-3.71 (2H, m,

NC(O)C*H*2), 7.22-7.28 (3H, m, Ar*H*), 7.38-7.41 (2H, m Ar*H*); δ_C (100 MHz, $CDCl_3$); *m*/*z* (MM-ES+APCI$^+$) 176.2 ($[M+H]^+$, 100%), 124.1 (6), 100.2 (28).

Also recovered was methyl 1-phenylpyrrolidine-2-carboxylate **44** as a pale yellow oil (38 mg, 62%) with data matching those reported above for this compound.

Computational Study

Computational Methods

Density functional theory[45-46] (DFT) was employed to calculate the electronic structures for the molecules and complexes discussed below (see main text for numbering). All structures have been optimized with the gradient corrected B97-D functional with a long-range dispersion correction.[47] The long-range dispersion correction is particularly important here for the donor-acceptor π-stacking interactions, which are the dominant interactions between donor and acceptor. All atoms were described with the 6-311++G(*d*,*p*) basis set.[48-49] Subsequent single point energy calculation of the optimized geometry was performed at the same level of theory within a polarizable continuum model (CPCM)[50] with the dielectric constant of *N,N*-dimethylformamide (DMF, $\varepsilon = 37.219$). All calculations were performed in Gaussian 09 suite of quantum chemistry programs.[51]

Results and Discussion

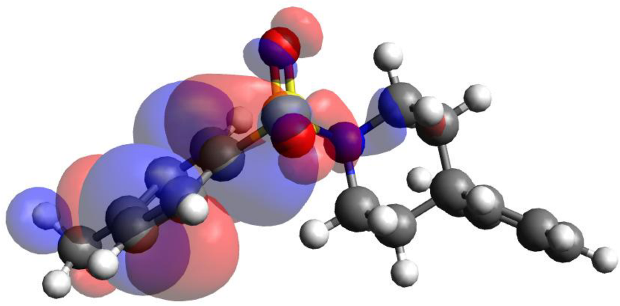

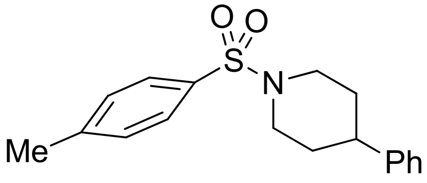

10

Figure S1. LUMO of **10**

The structural and energetic properties of the substrate **10** were initially investigated without the presence of the donor to establish the inherent reactivity of the molecule. The LUMO of the isolated substrate is located on the arenesulfonyl unit (Figure S1) and as such, Maslak's "regioconservation of spin" proposal[52,53] predicts that the donation of an electron to the LUMO of **10** would result in the dissociation of the substrate into the *p*-toluenesulfonyl radical and the dialkylamide anion. The energetics of this dissociation have been calculated to compare the relative energies of dissociating **10** into the *p*-toluenesulfonyl radical and the dialkylamide anion versus the *p*-toluenesulfinate anion and the dialkylaminyl radical (Scheme S1). In opposition to the Maslak proposals, the dissociation of **10** into the *p*-toluenesulfonyl anion and the dialkylamide radical was found to be favoured by 38.7 kcal/mol over the alternative mode of fragmentation (Scheme S1).

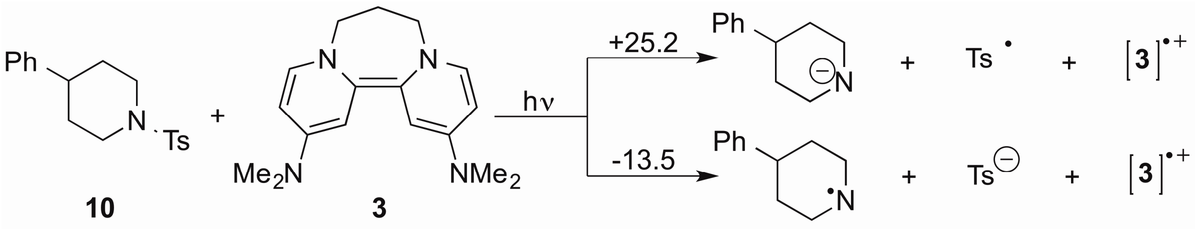

Scheme S1. Relative free energies for the dissociation of **10** upon electron transfer. Values (ΔG) are in kcal/mol and are calculated at the B97-D/6-311++G(d,p)/CPCM level of theory.

In order to determine whether the complex formed by the substrate, **10**, and the DMAP-based donor (**3**) affected the nature of the fragmentation, we investigated the energetics of the possible dissociation pathways. A complex between **3** and **10** can be formed through π-stacking of the phenyl ring of the toluene moiety of **10**, or alternatively through the phenyl ring attached to the dialkylamide moiety. Both of these possibilities were investigated and stable complexes were found to form. The most stable complex was formed through π-stacking and CH-π interactions of **3** with the phenyl ring, attached to the dialkylamide moiety of **10** (Figure S2a), which was found to be 6.1 kcal/mol more stable than the alternative arrangement (Figure S2b).

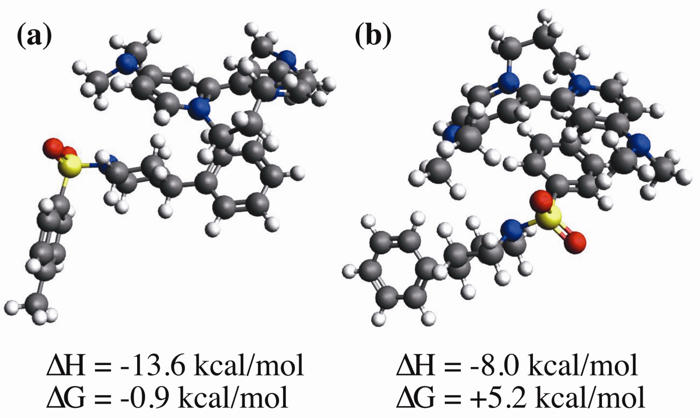

Figure S2. (a) Complex formed by π-stacking and CH-π interactions between **3** and the dialkylamide moiety of **10**. (b) Complex formed by π-stacking and CH-π interactions between **3** and the *p*-toluenesulfonyl moiety of **10**. Binding free enthalpies (ΔH) and energies (ΔG) are in kcal/mol and are calculated at the B97-D/6-311++G(d,p)/CPCM level of theory.

The significantly stronger complexation energy afforded through the interaction with the phenyl ring of the dialkylamide moiety suggests that this form of the complex will be the dominant species formed *in situ*. Nonetheless, in analogy to the isolated **10**, the LUMO of the complex is located on the arenesulfonyl unit (Figure S3) and as such, Maslak's "regioconservation of spin" rule[52-53] would again predict that the donation of an electron to **10** would result in the dissociation of the substrate into the *p*-toluenesulfonyl radical and the dialkylamide anion.

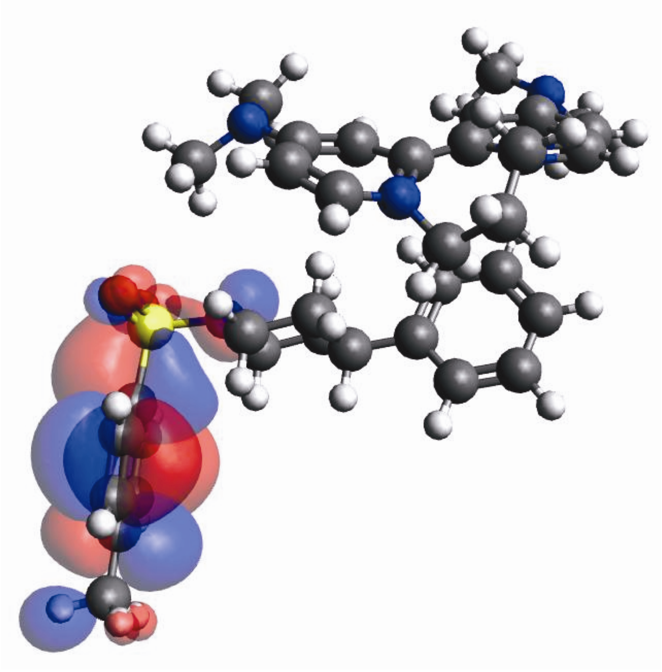

Figure S3. LUMO of the most stable complex between **10** and **3**.

In order to determine whether the dissociation predicted by the Maslak rules would be followed, we again calculated the energetics for the alternative dissociation pathways. In this case, this involved the formation of a complex between the radical cation of **3** and either the dialkylamide moiety as an anion or a radical. In the first case (dialkylamide anion) the overall complex is neutral and is calculated in the doublet state (Scheme S2a). In the second case (dialkylamide radical) the complex is calculated as a cation in the triplet state (Scheme S2b). As in the case of the isolated species, Maslak's rules are again found to not lead to the thermodynamically favoured product. Despite the fact that the LUMO of the complex resides predominantly on the aromatic group of the *p*-toluenesulfonyl group, the formation of the *p*-toluenesulfonyl radical is disfavoured by +38.7 kal/mol (ΔG = +29.0 kcal/mol, Scheme S2a) relative to the formation of the *p*-toluenesulfonyl anion (ΔG = −9.7 kcal/mol, Scheme S2b) and the complex between the dialkylamide radical and the radical cation of donor **3**.

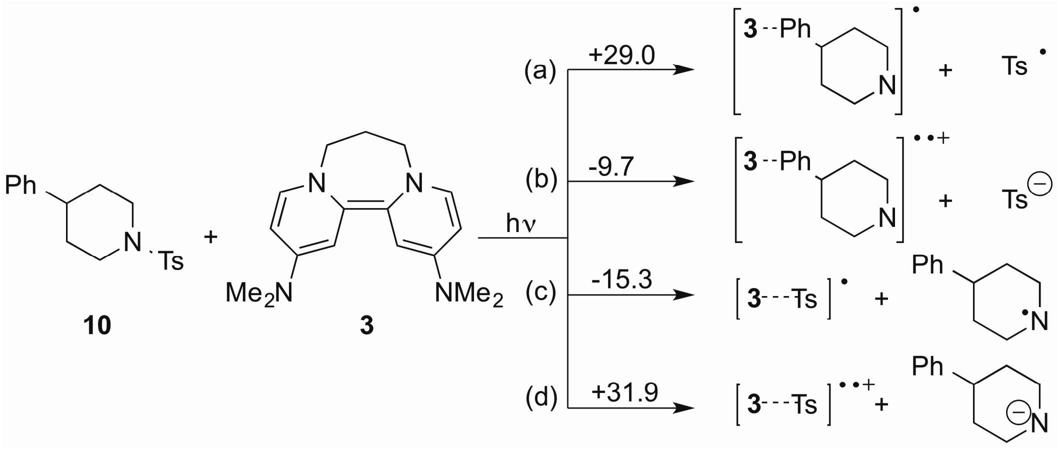

Scheme S2. Relative free energies for the dissociation of the complex formed between **10** and **3** upon electron transfer. Values (ΔG) are in kcal/mol and are calculated at the B97-D/6-311++G(d,p)/CPCM level of theory.

The favoured formation of the *p*-toluenesulfonyl anion was again confirmed for the case of the less stable complex between **3** and **10**, formed through π-stacking of the phenyl ring of the toluene moiety of **10** (Figure S2b). The dissociation of the dialkylamide moiety upon electron transfer again shows that the most favourable pathway involves the formation of the *p*-toluenesulfonyl anion and the dialkylaminyl radical, even when the *p*-toluenesulfonyl anion remains in complex with the radical cation of **3** (ΔG = −15.3 kcal/mol, Scheme S2c). The alternative formation of the dialkylamide anion is endergonic by 31.9 kcal/mol (Scheme 2d).

XYZ Coordinates

3

C -0.685774 0.470125 -0.132278
C -1.504850 -0.728188 -0.078948
C -2.881427 -0.704401 -0.135938
C -3.544086 0.590796 -0.273146
C -2.774983 1.700920 -0.459679
N -1.401675 1.679545 -0.468102
H -0.997826 -1.670888 0.088277
H -4.623875 0.684398 -0.311954
H -3.219900 2.675094 -0.655201
C 0.685756 0.470121 0.132281
C 1.504823 -0.728199 0.078949
N 1.401665 1.679537 0.468103
C 2.881400 -0.704419 0.135953
H 0.997792 -1.670892 -0.088284
C 2.774973 1.700902 0.459681
C 3.544068 0.590772 0.273151

H 3.219898 2.675075 0.655195
H 4.623859 0.684360 0.311944
C 0.670313 2.805380 1.064586
C 0.000001 3.688623 -0.000001
C -0.670315 2.805382 -1.064587
H -0.094672 2.380427 1.730777
H 1.364025 3.390575 1.680068
H -1.364023 3.390579 -1.680071
H 0.094668 2.380422 -1.730776
H -0.744725 4.335081 0.486754
H 0.744731 4.335077 -0.486758
N -3.681585 -1.869164 -0.135253
N 3.681579 -1.869166 0.135256
C 2.975815 -3.149435 0.132847
H 2.423783 -3.325333 -0.813157
H 3.708929 -3.955186 0.262301
H 2.257773 -3.179245 0.962453
C 4.821511 -1.882984 -0.797052
H 5.477265 -2.725310 -0.541166
H 4.485200 -2.003211 -1.846283
H 5.400344 -0.957617 -0.722646
C -4.821480 -1.883078 0.797102
H -5.477193 -2.725435 0.541213
H -4.485118 -2.003327 1.846314
H -5.400381 -0.957750 0.722763
C -2.975768 -3.149403 -0.132912
H -2.423695 -3.325308 0.813066
H -3.708853 -3.955181 -0.262369
H -2.257754 -3.179152 -0.962544

3* radical cation
C 0.711472 0.460805 0.106370
C 1.490102 -0.697612 -0.130652
C 2.885907 -0.720384 0.035505
C 3.516122 0.525062 0.417658
C 2.740633 1.621105 0.677440
N 1.376355 1.614549 0.577970
H 0.970068 -1.560008 -0.529446
H 4.590500 0.621886 0.513322
H 3.177362 2.561456 1.002189
C -0.711529 0.460815 -0.106385
C -1.490172 -0.697597 0.130615
N -1.376401 1.614574 -0.577964
C -2.885971 -0.720359 -0.035535
H -0.970156 -1.560026 0.529373
C -2.740679 1.621143 -0.677436
C -3.516181 0.525104 -0.417673
H -3.177397 2.561506 -1.002166
H -4.590552 0.621968 -0.513327

C -0.596554 2.750283 -1.113122
C -0.000013 3.623252 0.000022
C 0.596518 2.750255 1.113150
H 0.205244 2.324231 -1.729965
H -1.251198 3.335870 -1.765387
H 1.251168 3.335824 1.765426
H -0.205284 2.324200 1.729984
H 0.772420 4.267558 -0.440690
H -0.772439 4.267557 0.440745
N 3.631813 -1.848160 -0.179170
N -3.631778 -1.848199 0.179152
C -2.975167 -3.065589 0.661759
H -2.582137 -2.936466 1.684719
H -3.697853 -3.886281 0.658476
H -2.140818 -3.332507 -0.001413
C -5.095614 -1.767167 0.267425
H -5.497458 -2.778839 0.370911
H -5.417591 -1.166308 1.133566
H -5.514548 -1.325281 -0.645815
C 5.095629 -1.766874 -0.267447
H 5.497671 -2.778482 -0.370805
H 5.417496 -1.166057 -1.133657
H 5.514459 -1.324778 0.645742
C 2.975402 -3.065690 -0.661711
H 2.582284 -2.936667 -1.684650
H 3.698250 -3.886238 -0.658457
H 2.141153 -3.332775 0.001518

10
S -1.881126 -1.756135 0.207556
O -2.062864 -2.363272 1.546592
O -2.182671 -2.602234 -0.975461
C -2.853355 -0.242435 0.083530
C -2.703514 0.740809 1.073069
C -3.469674 1.904736 0.984923
C -4.383629 2.097868 -0.072731
C -4.507835 1.092758 -1.048060
C -3.745364 -0.081028 -0.980851
H -1.995128 0.600844 1.886341
H -3.357385 2.677337 1.744836
H -5.203842 1.230931 -1.874422
H -3.834678 -0.852465 -1.741539
C -5.215220 3.357734 -0.142627
H -5.740947 3.434381 -1.102393
H -5.965894 3.366688 0.662237
H -4.586412 4.249342 -0.010411
C 0.753204 -2.074644 0.630112
C 0.062142 -0.462959 -1.105924
C 2.060853 -1.304062 0.858777

H 0.908702 -2.856983 -0.134228
H 0.420173 -2.546981 1.559598
C 1.354358 0.342874 -0.914136
H 0.200826 -1.224457 -1.892968
H -0.756233 0.203462 -1.404002
C 2.507885 -0.561853 -0.421590
H 2.837101 -2.015720 1.172016
H 1.915528 -0.580574 1.673284
H 1.623432 0.810625 -1.871072
H 1.176514 1.143472 -0.182321
H 2.693561 -1.318912 -1.199837
N -0.294560 -1.114735 0.182722
C 3.789626 0.221335 -0.208247
C 4.940198 -0.064860 -0.963922
C 3.854294 1.253574 0.748117
C 6.128018 0.657746 -0.772130
H 4.901706 -0.861244 -1.707880
C 5.037287 1.978670 0.943910
H 2.973211 1.490855 1.343593
C 6.180133 1.682915 0.183451
H 7.009116 0.420193 -1.367636
H 5.068094 2.773544 1.688681
H 7.100315 2.245964 0.335095

10* radical anion (= **19)**
S -2.846394 0.286851 1.240886
O -4.304223 -0.143874 0.930549
O -2.468537 0.064659 2.728867
C -1.836026 -1.024347 0.366727
C -2.251991 -1.515571 -0.877559
C -1.425472 -2.396238 -1.591119
C -0.169826 -2.782546 -1.084832
C 0.226992 -2.283852 0.170225
C -0.596488 -1.411018 0.891074
H -3.217528 -1.214172 -1.282678
H -1.751198 -2.780226 -2.558901
H 1.199650 -2.563717 0.575663
H -0.275661 -1.020525 1.854732
C 0.756272 -3.672722 -1.884911
H 1.558120 -3.075912 -2.348020
H 1.238840 -4.422918 -1.242751
H 0.215661 -4.191323 -2.687516
C -0.208951 3.697484 -0.778436
C -0.972906 1.780506 -1.975285
C 1.073394 2.958427 -0.288829
H -0.996596 3.530259 -0.014980
H -0.017151 4.777196 -0.851402
C 0.280889 0.951997 -1.573286
H -1.744472 1.610860 -1.196722

H -1.364768 1.428393 -2.939776
C 0.808122 1.436875 -0.212478
H 1.361962 3.356207 0.694205
H 1.899010 3.152522 -0.990277
H 0.013647 -0.109020 -1.531121
H 1.048613 1.084451 -2.349207
H 0.001349 1.286898 0.521303
N -0.658805 3.194600 -2.063342
C 2.025904 0.685174 0.298334
C 2.219763 0.567655 1.687720
C 2.977806 0.104670 -0.558247
C 3.322682 -0.119137 2.210885
H 1.483464 1.006438 2.361976
C 4.085348 -0.584778 -0.041157
H 2.851141 0.172121 -1.636953
C 4.261657 -0.702293 1.344970
H 3.445594 -0.207028 3.290181
H 4.806029 -1.035935 -0.723059
H 5.117277 -1.244380 1.746413

Dialkylamide anion fragment derived from **10,** i.e. **22**
C 2.859315 0.010102 1.175664
C 2.862048 0.229614 -1.151310
C 1.321237 -0.238099 1.247436
H 2.974590 1.130233 1.330602
H 3.330540 -0.470265 2.054370
C 1.323296 -0.000219 -1.271729
H 2.977067 1.358895 -1.094528
H 3.334590 -0.078908 -2.103395
C 0.626091 0.436257 0.039689
H 0.895319 0.161881 2.186787
H 1.130113 -1.323340 1.220698
H 0.899668 0.568719 -2.120575
H 1.132218 -1.071292 -1.449159
H 0.791650 1.523180 0.142604
N 3.487580 -0.443140 -0.040361
C -0.871011 0.199731 0.017195
C -1.775975 1.273806 0.108634
C -1.397904 -1.103048 -0.094659
C -3.163524 1.060229 0.090046
H -1.383116 2.288041 0.195390
C -2.781652 -1.324799 -0.113511
H -0.713179 -1.947647 -0.167083
C -3.672356 -0.242218 -0.020890
H -3.844574 1.908401 0.162322
H -3.166904 -2.340990 -0.199922
H -4.748420 -0.413071 -0.034975

Dialkylaminyl radical fragment derived from **10**, i.e. **20**

C 2.858727 0.038701 -1.195595
C 2.853790 -0.290188 1.166356
C 1.320357 0.281310 -1.232614
H 3.031350 -1.047802 -1.335150
H 3.343840 0.578709 -2.020227
C 1.314205 -0.067830 1.261385
H 3.026782 -1.373619 1.004644
H 3.334367 0.004427 2.109334
C 0.629921 -0.461679 -0.066901
H 0.927082 -0.060561 -2.199985
H 1.125746 1.360251 -1.150713
H 0.917575 -0.662383 2.095936
H 1.117234 0.992008 1.477435
H 0.788510 -1.540850 -0.217897
N 3.419708 0.474169 0.070192
C -0.867287 -0.206312 -0.032428
C -1.775919 -1.271512 -0.162925
C -1.378756 1.095516 0.133621
C -3.160992 -1.047476 -0.129282
H -1.391027 -2.283714 -0.291365
C -2.760716 1.325379 0.167538
H -0.691671 1.934635 0.237343
C -3.658162 0.253326 0.036204
H -3.848526 -1.886636 -0.231837
H -3.137654 2.339669 0.296402
H -4.732583 0.431273 0.062584

p-Toluenesulfinate anion **21**
S -2.234534 -0.017435 -0.350426
O -2.656571 -1.268130 0.466699
O -2.656439 1.305785 0.342613
C -0.369781 -0.007305 -0.129562
C 0.336867 -1.217412 -0.116940
C 1.737149 -1.209416 -0.027313
C 2.453204 0.000883 0.038690
C 1.726860 1.207651 0.020448
C 0.327528 1.207532 -0.071683
H -0.207600 -2.160629 -0.154532
H 2.283056 -2.153710 -0.001813
H 2.264060 2.155284 0.083975
H -0.227969 2.145008 -0.074459
C 3.965744 0.009264 0.107356
H 4.327149 0.796165 0.784015
H 4.351996 -0.958207 0.454490
H 4.400509 0.206626 -0.885339

p-Toluenesulfonyl radical **23**
S 2.169679 -0.000262 0.250008
O 2.695418 -1.302745 -0.285146

O 2.695988 1.302285 -0.284407
C 0.351330 0.000176 0.085367
C -0.325191 -1.227395 0.074680
C -1.721049 -1.212411 -0.002277
C -2.438399 0.000716 -0.037007
C -1.720716 1.213292 -0.002695
C -0.324536 1.227791 0.074352
H 0.223176 -2.165685 0.099646
H -2.261769 -2.157141 -0.039351
H -2.261173 2.158131 -0.040211
H 0.224114 2.165917 0.099230
C -3.947614 -0.000199 -0.080284
H -4.330823 0.911564 -0.555561
H -4.326902 -0.876065 -0.622177
H -4.355693 -0.040669 0.941894

Complex between **3** and **10** dialkylamide moiety

C 0.422457 -2.232918 -0.621762
C 1.559635 -1.536678 -1.068661
C 2.818663 -2.151270 -1.077438
C 2.963869 -3.471084 -0.627481
C 1.839287 -4.175097 -0.171104
C 0.580512 -3.557532 -0.172843
H 1.470368 -0.498067 -1.381427
H 3.689050 -1.584301 -1.398140
H 1.940948 -5.199582 0.186777
H -0.294179 -4.103309 0.183523
C 0.904414 2.575133 1.138786
C 1.918591 1.916165 0.476324
C 2.662019 0.830120 1.097237
C 1.020203 0.895756 2.889212
C 0.514394 2.088440 2.457563
H 2.213665 2.240194 -0.514899
H 0.668397 0.415763 3.801679
H -0.208259 2.615990 3.070836
C 3.916090 0.408952 0.669676
C 4.504812 0.839177 -0.587639
C 5.758923 -1.186584 0.813507
C 5.594013 0.219133 -1.158420
H 4.020942 1.653963 -1.110747
C 6.210663 -0.891152 -0.438422
H 6.250044 -1.935521 1.432341
H 7.069179 -1.425821 -0.829434
N 1.964215 0.193833 2.185637
N 4.698488 -0.551120 1.408923
C 2.158011 -1.240267 2.443006
C 4.559155 -0.612291 2.873155
C 3.394247 -1.509635 3.316341

H 1.249171 -1.638498 2.910265
H 2.274535 -1.729402 1.470748
H 3.670877 -2.570341 3.226552
H 3.175049 -1.305334 4.374703
H 5.506592 -0.960544 3.301900
H 4.389759 0.416319 3.224342
N 0.255422 3.719323 0.628255
N 6.192683 0.646428 -2.365111
C 0.730762 4.224516 -0.658291
H 0.521365 3.521472 -1.490247
H 0.224946 5.174727 -0.870452
H 1.814099 4.397170 -0.616068
C -1.217725 3.721426 0.689468
H -1.579045 4.737850 0.483498
H -1.652227 3.027946 -0.050963
H -1.569257 3.422062 1.680389
C 5.626385 1.841115 -2.988729
H 6.251240 2.119943 -3.846171
H 4.589568 1.676656 -3.347209
H 5.616624 2.669005 -2.268358
C 6.456102 -0.403037 -3.364708
H 5.517535 -0.751985 -3.839377
H 7.111191 0.007289 -4.144137
H 6.954925 -1.263705 -2.909642
H 3.947333 -3.939780 -0.619105
C -0.941528 -1.574836 -0.558490
C -1.045125 -0.586844 0.623901
C -1.332277 -0.819957 -1.844809
H -1.690599 -2.367494 -0.397825
C -2.462626 -0.009576 0.731844
H -0.318072 0.224594 0.492432
H -0.811234 -1.094314 1.568364
C -2.753854 -0.255823 -1.719339
H -0.626359 0.002646 -2.024581
H -1.302985 -1.492109 -2.713370
H -2.508822 0.732719 1.534003
H -3.175530 -0.825343 0.951778
H -3.025939 0.308274 -2.616914
H -3.465369 -1.092318 -1.594511
S -4.313508 1.522970 -0.471657
O -4.285569 2.321124 0.770066
O -4.465993 2.203277 -1.774509
N -2.834157 0.662657 -0.546458
C -5.610510 0.280057 -0.322393
C -6.177196 -0.267747 -1.482398
C -5.951377 -0.200116 0.950860
C -7.113767 -1.299142 -1.355511
H -5.897928 0.113068 -2.461513
C -6.889751 -1.232675 1.054937

H -5.501566 0.233596 1.840482
C -7.484300 -1.795531 -0.090736
H -7.562490 -1.726190 -2.251701
H -7.163948 -1.608298 2.040116
C -8.518152 -2.890551 0.036288
H -8.358581 -3.480858 0.947776
H -9.528581 -2.456163 0.093121
H -8.494484 -3.560440 -0.833110

Complex between **3** and **10** *p*-toluenesulfonyl moiety

C 1.980459 -0.269182 -2.908682
C 2.141347 -1.636601 -2.598062
C 1.433443 -2.220810 -1.548632
C 0.578552 -1.419570 -0.778059
C 0.417789 -0.053226 -1.043288
C 1.108929 0.501859 -2.125511
H 2.819087 -2.247774 -3.192674
H 1.536900 -3.279264 -1.326550
H -0.229817 0.565384 -0.429191
H 0.995293 1.563725 -2.332339
C 4.117519 -1.389520 0.584257
C 3.275646 -0.359203 0.945948
C 3.201638 0.909267 0.249585
C 4.918607 0.008284 -1.217019
C 4.977060 -1.186519 -0.564528
H 2.538409 -0.530264 1.718626
H 5.566283 0.203299 -2.067608
H 5.695936 -1.928751 -0.893651
C 2.298719 1.890833 0.650393
C 1.457907 1.747449 1.832233
C 0.689881 3.491161 -0.185538
C 0.197366 2.293077 1.873533
H 1.827615 1.161864 2.668716
C -0.238482 3.090394 0.723576
H 0.460417 4.232477 -0.953463
H -1.255067 3.461875 0.638688
N 4.085105 1.043156 -0.870945
N 2.006018 3.051811 -0.174636
C 4.252672 2.316103 -1.614853
C 3.014443 4.114273 -0.339511
C 4.387510 3.520770 -0.674821
H 5.161401 2.215124 -2.216917
H 3.409255 2.458143 -2.302443
H 5.001871 4.294278 -1.153654
H 4.908548 3.206395 0.239020
H 2.671460 4.747886 -1.171674
H 3.075217 4.751740 0.561790
N 4.202771 -2.596458 1.294635

N -0.659932 2.242506 3.004452
C 3.419118 -2.714501 2.525369
H 2.332848 -2.760408 2.331557
H 3.727981 -3.629639 3.046701
H 3.618126 -1.849722 3.171911
C 4.226239 -3.854032 0.532539
H 4.632935 -4.648851 1.171991
H 3.209955 -4.149155 0.210849
H 4.857096 -3.764426 -0.356190
C -0.119408 1.570314 4.186942
H -0.837543 1.676853 5.009939
H 0.059905 0.491068 4.010754
H 0.830946 2.037058 4.476699
C -2.034826 1.790553 2.727912
H -2.059526 0.717388 2.464981
H -2.647788 1.948344 3.625077
H -2.478630 2.358653 1.904278
S -0.364697 -2.198114 0.543200
O -0.289845 -3.672884 0.353270
O 0.013294 -1.682829 1.879875
C 2.751859 0.338384 -4.054934
H 2.570115 -0.214235 -4.988421
H 2.476173 1.388422 -4.212084
H 3.833096 0.289910 -3.856685
C -2.488035 -1.976981 -1.089770
C -2.904058 -1.755004 1.323409
C -3.678884 -1.067055 -1.416830
H -2.811663 -3.032391 -1.053650
H -1.713835 -1.874595 -1.858471
C -4.119303 -0.853829 1.059339
H -3.223691 -2.811306 1.379531
H -2.425947 -1.480059 2.269195
C -4.761986 -1.150279 -0.315394
H -4.102776 -1.367987 -2.384825
H -3.323878 -0.030530 -1.508047
H -4.854780 -1.009529 1.860811
H -3.799022 0.195572 1.094358
H -5.136360 -2.185939 -0.295640
C -5.930751 -0.227671 -0.605020
C -7.214819 -0.747374 -0.844669
C -5.752920 1.169189 -0.646189
C -8.297808 0.102370 -1.118509
H -7.363604 -1.827314 -0.815222
C -6.830427 2.022605 -0.918189
H -4.764666 1.590647 -0.463834
C -8.108527 1.491436 -1.155859
H -9.285620 -0.319907 -1.301026
H -6.674115 3.100729 -0.944780
H -8.946955 2.154216 -1.367032

N -1.924369 -1.566052 0.221813

Complex between radical cation of **3** and **22** (dialkylamide anion moiety of **10**)

C -2.043947 -1.979697 -0.817363
C -1.292461 -0.880265 -1.277291
C 0.088520 -0.984474 -1.476932
C 0.753930 -2.192660 -1.217078
C 0.020954 -3.295372 -0.756848
C -1.365250 -3.184618 -0.560265
H -1.793730 0.068605 -1.464436
H 0.654065 -0.118233 -1.812835
H 0.525908 -4.237700 -0.544005
H -1.933133 -4.042959 -0.198251
C -0.387231 3.090601 0.202775
C 0.776661 2.309092 0.171919
C 0.902369 1.083355 0.879570
C -1.358628 1.395871 1.645548
C -1.485226 2.597403 1.005684
H 1.657587 2.664537 -0.347709
H -2.178013 0.973918 2.218783
H -2.419182 3.136903 1.103469
C 2.112088 0.318985 0.838961
C 2.971723 0.347417 -0.291819
C 3.551161 -1.371508 1.779646
C 4.091613 -0.483755 -0.416843
H 2.655557 0.958613 -1.127077
C 4.348913 -1.405700 0.670529
H 3.739447 -2.016421 2.633886
H 5.160833 -2.121755 0.644120
N -0.236694 0.620129 1.580695
N 2.490354 -0.519562 1.915909
C -0.311173 -0.788138 2.022149
C 1.852861 -0.354419 3.236448
C 0.481632 -1.040118 3.308465
H -1.365128 -1.048898 2.147247
H 0.085001 -1.397200 1.202689
H 0.600946 -2.125116 3.432629
H -0.056752 -0.653164 4.184253
H 2.533161 -0.748605 3.997610
H 1.743264 0.725120 3.404210
N -0.484918 4.289617 -0.456903
N 4.905002 -0.463719 -1.525911
C 0.585681 4.694417 -1.369407
H 0.701135 3.976331 -2.199303
H 0.353167 5.680740 -1.779838
H 1.543387 4.760448 -0.833358
C -1.771514 4.990700 -0.534524
H -1.619240 5.950811 -1.035553

H -2.519884 4.409967 -1.098592
H -2.165301 5.189995 0.470826
C 4.545865 0.391945 -2.657691
H 5.360782 0.377964 -3.387428
H 3.616212 0.054030 -3.149132
H 4.402262 1.426167 -2.316929
C 5.823536 -1.578525 -1.787778
H 5.282205 -2.524074 -1.959699
H 6.413908 -1.342719 -2.677984
H 6.517514 -1.715636 -0.949268
H 1.832310 -2.260194 -1.351569
C -3.538186 -1.862365 -0.589195
C -3.879971 -0.915771 0.585138
C -4.309827 -1.417484 -1.853683
H -3.915458 -2.862793 -0.315002
C -5.425019 -0.786007 0.737357
H -3.450842 0.077950 0.386537
H -3.429570 -1.304658 1.516102
C -5.824054 -1.254423 -1.524002
H -3.916759 -0.449137 -2.201862
H -4.156457 -2.157600 -2.659663
H -5.636613 -0.107601 1.584375
H -5.773637 -1.813638 1.057698
H -6.351088 -0.941958 -2.444187
H -6.189096 -2.298711 -1.288292
N -6.102158 -0.327930 -0.451400

Complex between radical cation of **3** and **20** (dialkylaminyl radical moiety of **10**)

C -2.074745 -2.007448 -0.716042
C -1.349434 -0.925900 -1.250656
C 0.027973 -1.027430 -1.472966
C 0.709427 -2.214231 -1.163103
C -0.001233 -3.298537 -0.630074
C -1.383783 -3.192137 -0.409251
H -1.863736 0.005602 -1.483228
H 0.573820 -0.175446 -1.871730
H 0.518674 -4.222589 -0.378809
H -1.935240 -4.034257 0.010030
C -0.394248 3.064498 0.068279
C 0.781750 2.294409 0.076819
C 0.909283 1.100713 0.826887
C -1.328307 1.456153 1.629536
C -1.464634 2.626740 0.936300
H 1.662949 2.637091 -0.450916
H -2.127111 1.075676 2.258811
H -2.381287 3.191502 1.053964
C 2.124685 0.333373 0.805491
C 2.946386 0.290666 -0.346237

C 3.598109 -1.272927 1.823502
C 4.077891 -0.535096 -0.441827
H 2.603413 0.844981 -1.210113
C 4.370610 -1.374013 0.699727
H 3.812623 -1.856768 2.714436
H 5.190039 -2.082252 0.702482
N -0.215349 0.666640 1.561083
N 2.529750 -0.425371 1.924627
C -0.273820 -0.715933 2.082404
C 1.903090 -0.191854 3.242247
C 0.543412 -0.891563 3.368761
H -1.323506 -0.972881 2.244070
H 0.111014 -1.368025 1.291052
H 0.678779 -1.966888 3.546778
H 0.011594 -0.466173 4.230313
H 2.598183 -0.532363 4.015666
H 1.781132 0.893652 3.348750
N -0.525956 4.191161 -0.696893
N 4.855961 -0.581874 -1.568696
C 0.609586 4.659758 -1.494698
H 0.982701 3.846630 -2.132649
H 0.280527 5.482174 -2.135782
H 1.437721 5.011486 -0.855910
C -1.659376 5.102462 -0.494657
H -1.621270 5.884058 -1.258481
H -2.610495 4.565084 -0.602062
H -1.628120 5.575011 0.500623
C 4.457900 0.188224 -2.749607
H 5.245020 0.115143 -3.505118
H 3.511471 -0.185162 -3.177271
H 4.326751 1.246255 -2.483979
C 5.848172 -1.650214 -1.742220
H 5.370503 -2.641291 -1.811743
H 6.408548 -1.462017 -2.662257
H 6.558653 -1.656812 -0.905841
H 1.784699 -2.280397 -1.318553
C -3.569900 -1.895346 -0.472735
C -3.899815 -0.806995 0.572716
C -4.344126 -1.597754 -1.776804
H -3.928315 -2.859585 -0.080338
C -5.439912 -0.648214 0.738921
H -3.469971 0.152816 0.254175
H -3.466855 -1.064947 1.548884
C -5.861315 -1.408926 -1.479781
H -3.952769 -0.678475 -2.235195
H -4.217912 -2.415796 -2.499399
H -5.657299 0.144097 1.468336
H -5.836583 -1.607002 1.130321
H -6.401424 -1.190037 -2.411005

H -6.245022 -2.362568 -1.063836
N -6.067786 -0.331185 -0.530132

Complex between radical cation of **3** and **21** (*p*-toluenesulfinate anion moiety of **10**)

C -1.838435 0.279502 2.505289
C -2.743282 -0.562532 1.832889
C -2.300328 -1.726045 1.192129
C -0.942078 -2.062210 1.220707
C -0.027802 -1.242261 1.889329
C -0.476726 -0.075960 2.521325
H -3.800006 -0.295409 1.800755
H -3.009929 -2.358804 0.657016
H 1.025340 -1.518326 1.910017
H 0.235987 0.570656 3.035262
C -2.387376 0.376479 -1.356353
C -0.988306 0.302874 -1.249913
C -0.222118 1.304295 -0.610694
C -2.269594 2.492355 -0.168987
C -3.021183 1.565623 -0.832169
H -0.446324 -0.533534 -1.674321
H -2.717130 3.376150 0.277091
H -4.083909 1.748223 -0.931394
C 1.217520 1.265126 -0.589612
C 1.936347 0.044095 -0.558972
C 3.320408 2.433328 -0.418593
C 3.340104 0.002235 -0.451138
H 1.353655 -0.871874 -0.539605
C 4.028751 1.269595 -0.322824
H 3.809495 3.402976 -0.375536
H 5.098508 1.333019 -0.166277
N -0.921600 2.364576 0.012112
N 1.966293 2.468542 -0.604542
C -0.232507 3.262695 0.956863
C 1.321199 3.749294 -0.957167
C 0.563430 4.355785 0.231578
H -0.981835 3.694795 1.625974
H 0.437019 2.637828 1.560373
H 1.262592 4.815366 0.943280
H -0.108918 5.139892 -0.142347
H 2.093892 4.431127 -1.325636
H 0.628707 3.544836 -1.783626
N -3.139671 -0.611428 -1.938753
N 4.050231 -1.170074 -0.437677
C -2.478677 -1.687040 -2.685567
H -1.688738 -2.146617 -2.081340
H -3.222255 -2.452459 -2.930609
H -2.035802 -1.306707 -3.624013

C -4.563992 -0.389612 -2.206650
H -5.001801 -1.325371 -2.566636
H -5.087910 -0.101043 -1.286568
H -4.725965 0.393265 -2.967738
C 3.340097 -2.453266 -0.443400
H 4.061566 -3.253025 -0.642371
H 2.827672 -2.653512 0.510607
H 2.569519 -2.464939 -1.221619
C 5.462781 -1.174993 -0.040525
H 5.595519 -0.845716 1.003815
H 5.849335 -2.193340 -0.139154
H 6.055134 -0.519989 -0.693335
S -0.380802 -3.592305 0.309101
O 0.175077 -2.991391 -1.013828
O 0.769170 -4.116731 1.207759
C -2.329078 1.531711 3.196509
H -2.997515 1.283180 4.034899
H -1.493320 2.123159 3.591651
H -2.905438 2.160296 2.502029

Complex between radical cation of **3** and **23** (*p*-toluenesulfonyl radical moiety of **10**)

C -1.998459 0.946193 2.325752
C -2.182620 -0.428490 2.083296
C -1.090225 -1.282442 1.918850
C 0.199928 -0.740901 1.995742
C 0.423851 0.612570 2.263317
C -0.686552 1.451229 2.405895
H -3.190857 -0.829877 2.001410
H -1.242194 -2.340339 1.717572
H 1.435902 1.003506 2.317715
H -0.529865 2.515164 2.579374
C -2.394473 -1.345295 -1.199490
C -1.111990 -0.772902 -1.180038
C -0.864946 0.619746 -1.043963
C -3.254858 0.888352 -0.926659
C -3.506299 -0.443439 -1.096733
H -0.261352 -1.435506 -1.152468
H -4.073676 1.592038 -0.816721
H -4.536461 -0.776394 -1.124466
C 0.497014 1.097224 -1.051074
C 1.568630 0.193429 -1.318431
C 2.119695 2.549228 -0.027521
C 2.872643 0.407941 -0.851492
H 1.353780 -0.712469 -1.868405
C 3.087780 1.590711 -0.043258
H 2.297302 3.526008 0.418804
H 4.016987 1.773742 0.481901
N -2.001964 1.437630 -0.879725

N 0.883346 2.403632 -0.619207
C -1.921411 2.901726 -0.672064
C 0.393291 3.659547 -1.257603
C -1.046707 3.567290 -1.734694
H -2.941989 3.288406 -0.713528
H -1.527975 3.092624 0.334799
H -1.405695 4.585271 -1.926953
H -1.119963 3.009004 -2.676767
H 0.480565 4.444350 -0.494354
H 1.064051 3.914748 -2.091644
N -2.580080 -2.693685 -1.259686
N 3.905404 -0.437895 -1.146531
C -1.429080 -3.582947 -1.456174
H -0.712436 -3.482623 -0.628997
H -1.780768 -4.616776 -1.498978
H -0.908299 -3.342449 -2.396812
C -3.935244 -3.258250 -1.277506
H -3.864697 -4.346746 -1.207990
H -4.515474 -2.892855 -0.419395
H -4.469408 -2.994945 -2.204053
C 3.635103 -1.673771 -1.885345
H 4.579927 -2.190904 -2.072641
H 2.961136 -2.332320 -1.312617
H 3.164635 -1.442098 -2.851284
C 5.182085 -0.318240 -0.433158
H 5.048992 -0.522752 0.640288
H 5.889551 -1.038174 -0.853783
H 5.599882 0.688952 -0.559097
S 1.615216 -1.883408 1.832739
O 1.397569 -2.709735 0.583270
O 2.874926 -1.063504 1.955590
C -3.188527 1.854352 2.518661
H -3.487898 1.869927 3.578167
H -2.955306 2.886160 2.226921
H -4.050542 1.502834 1.937489

NMR Spectra

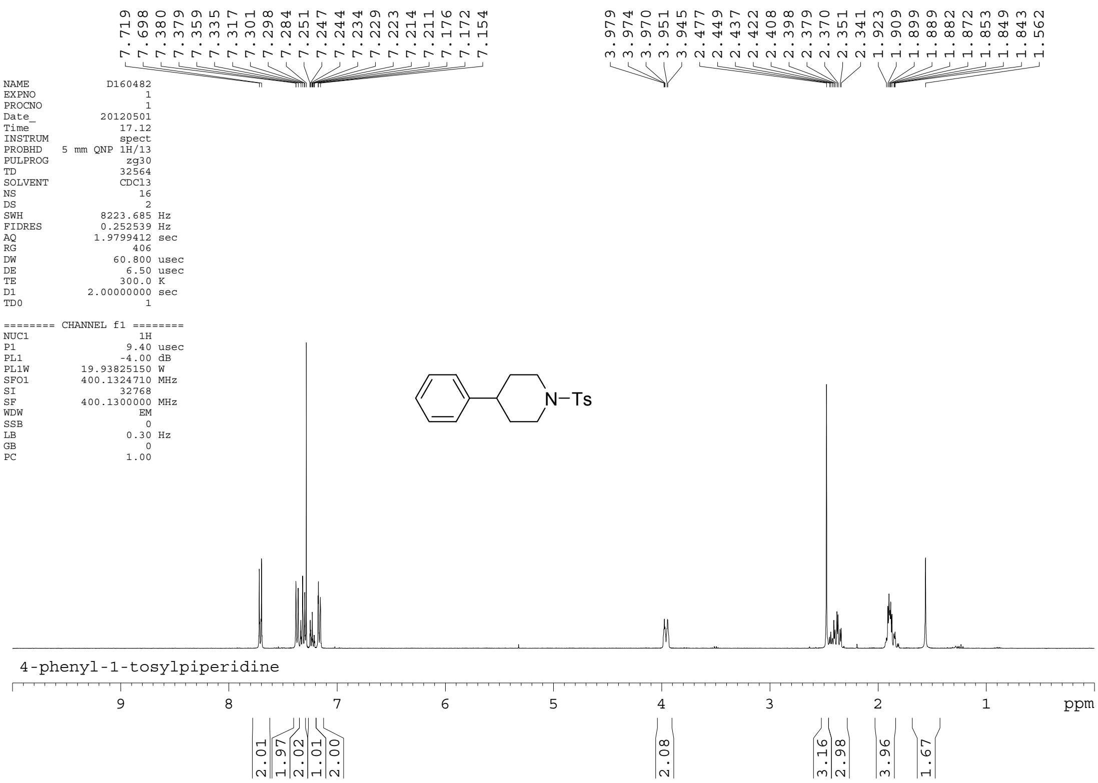
7.719 7.698 7.380 7.379 7.359 7.335 7.317 7.301 7.298 7.284 7.251 7.247 7.244 7.234 7.229 7.223 7.214 7.211 7.176 7.172 7.154
3.979 3.974 3.970 3.951 3.945 2.477 2.449 2.437 2.422 2.408 2.398 2.379 2.370 2.351 2.341 1.923 1.909 1.899 1.889 1.882 1.872 1.853 1.849 1.843 1.562
NAME D160482
EXPNO 1
PROCNO 1
Date_ 20120501
Time 17.12
INSTRUM spect
PROBHD 5 mm QNP 1H/13
PULPROG zg30
TD 32564
SOLVENT CDCl3
NS 16
DS 2
SWH 8223.685 Hz
FIDRES 0.252539 Hz
AQ 1.9799412 sec
RG 406
DW 60.800 usec
DE 6.50 usec
TE 300.0 K
D1 2.00000000 sec
TD0 1
======== CHANNEL f1 ========
NUC1 1H
P1 9.40 usec
PL1 -4.00 dB
PL1W 19.93825150 W
SFO1 400.1324710 MHz
SI 32768
SF 400.1300000 MHz
WDW EM
SSB 0
LB 0.30 Hz
GB 0
PC 1.00
N−Ts
4-phenyl-1-tosylpiperidine
9 8 7 6 5 4 3 2 1 ppm
2.01 1.97 2.02 1.01 2.00 2.08 3.16 2.98 3.96 1.67

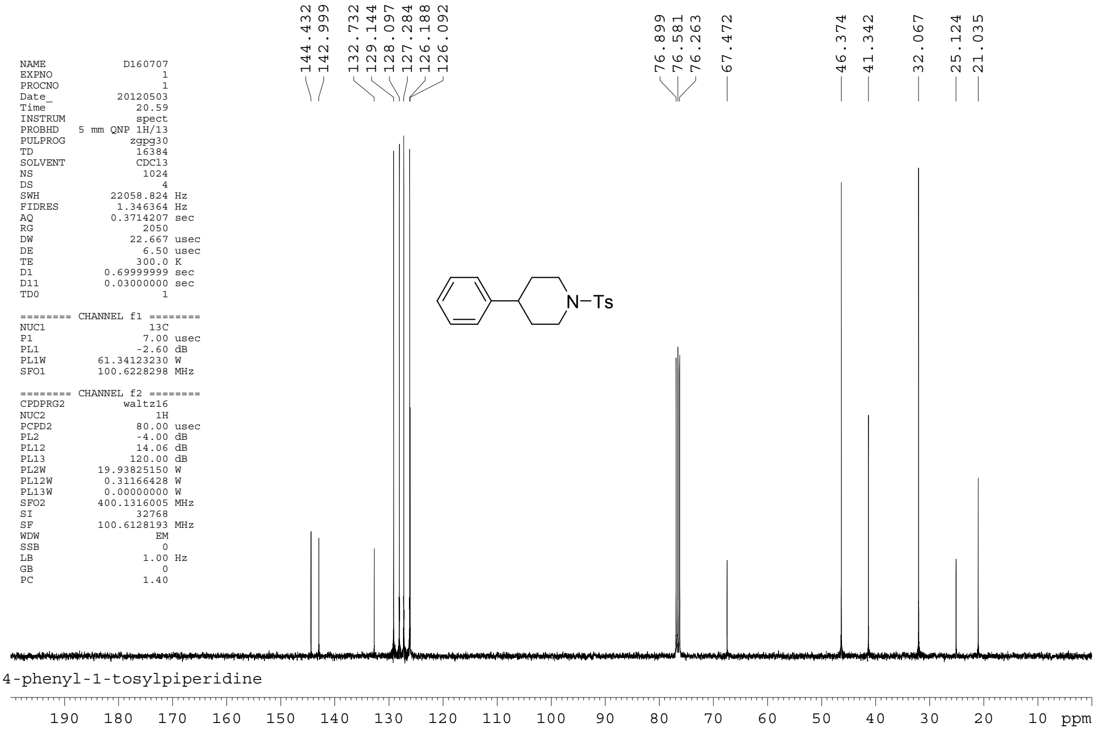
144.432 142.999 132.732 129.144 128.097 127.284 126.188 126.092 76.899 76.581 76.263 67.472 46.374 41.342 32.067 25.124 21.035
NAME D160707
EXPNO 1
PROCNO 1
Date_ 20120503
Time 20.59
INSTRUM spect
PROBHD 5 mm QNP 1H/13
PULPROG zgpg30
TD 16384
SOLVENT CDCl3
NS 1024
DS 4
SWH 22058.824 Hz
FIDRES 1.346364 Hz
AQ 0.3714207 sec
RG 2050
DW 22.667 usec
DE 6.50 usec
TE 300.0 K
D1 0.69999999 sec
D11 0.03000000 sec
TD0 1
======== CHANNEL f1 ========
NUC1 13C
P1 7.00 usec
PL1 -2.60 dB
PL1W 61.34123230 W
SFO1 100.6228298 MHz
======== CHANNEL f2 ========
CPDPRG2 waltz16
NUC2 1H
PCPD2 80.00 usec
PL2 -4.00 dB
PL12 14.06 dB
PL13 120.00 dB
PL2W 19.93825150 W
PL12W 0.31166428 W
PL13W 0.00000000 W
SFO2 400.1316005 MHz
SI 32768
SF 100.6128193 MHz
WDW EM
SSB 0
LB 1.00 Hz
GB 0
PC 1.40
N−Ts
4-phenyl-1-tosylpiperidine
190 180 170 160 150 140 130 120 110 100 90 80 70 60 50 40 30 20 10 ppm

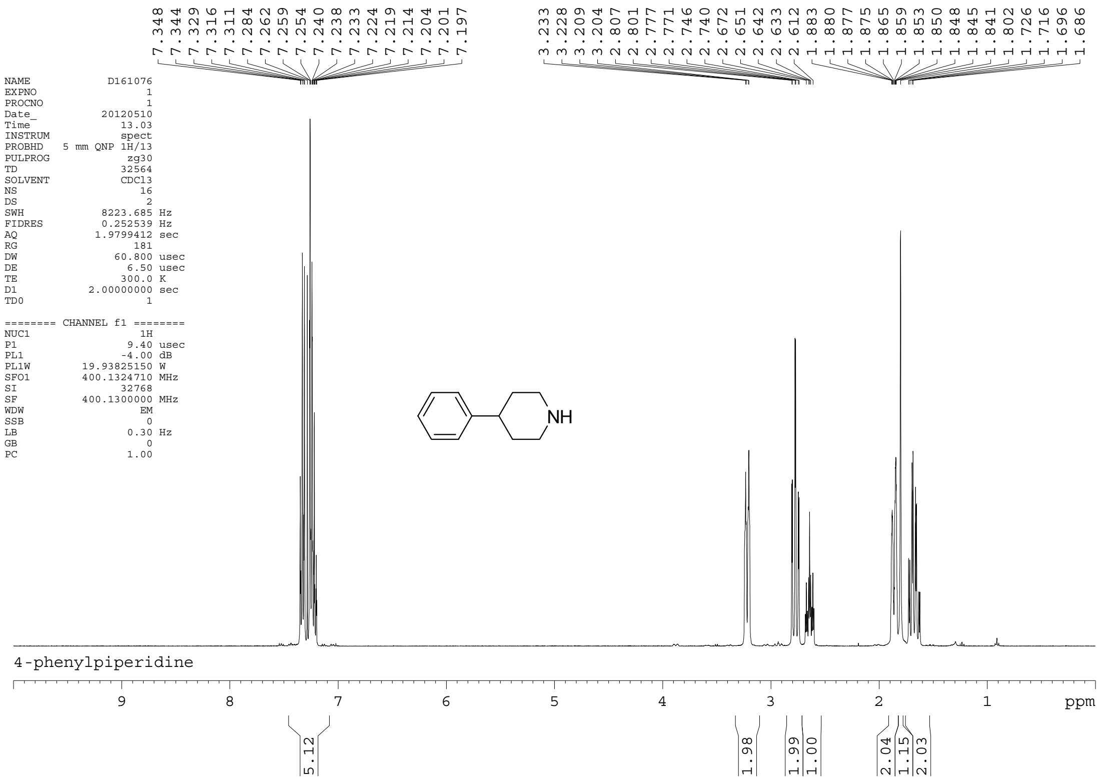
7.348 7.344 7.329 7.316 7.311 7.284 7.262 7.259 7.254 7.240 7.238 7.233 7.224 7.219 7.214 7.204 7.201 7.197
3.233 3.228 3.209 3.204 2.807 2.801 2.777 2.771 2.746 2.740 2.672 2.651 2.642 2.633 2.612 1.883 1.880 1.877 1.875 1.865 1.859 1.853 1.850 1.848 1.845 1.841 1.802 1.726 1.716 1.696 1.686
NAME D161076
EXPNO 1
PROCNO 1
Date_ 20120510
Time 13.03
INSTRUM spect
PROBHD 5 mm QNP 1H/13
PULPROG zg30
TD 32564
SOLVENT CDCl3
NS 16
DS 2
SWH 8223.685 Hz
FIDRES 0.252539 Hz
AQ 1.9799412 sec
RG 181
DW 60.800 usec
DE 6.50 usec
TE 300.0 K
D1 2.00000000 sec
TD0 1
======== CHANNEL f1 ========
NUC1 1H
P1 9.40 usec
PL1 -4.00 dB
PL1W 19.93825150 W
SFO1 400.1324710 MHz
SI 32768
SF 400.1300000 MHz
WDW EM
SSB 0
LB 0.30 Hz
GB 0
PC 1.00
NH
4-phenylpiperidine
9 8 7 6 5 4 3 2 1 ppm
5.12 1.98 1.99 1.00 2.04 1.15 2.03

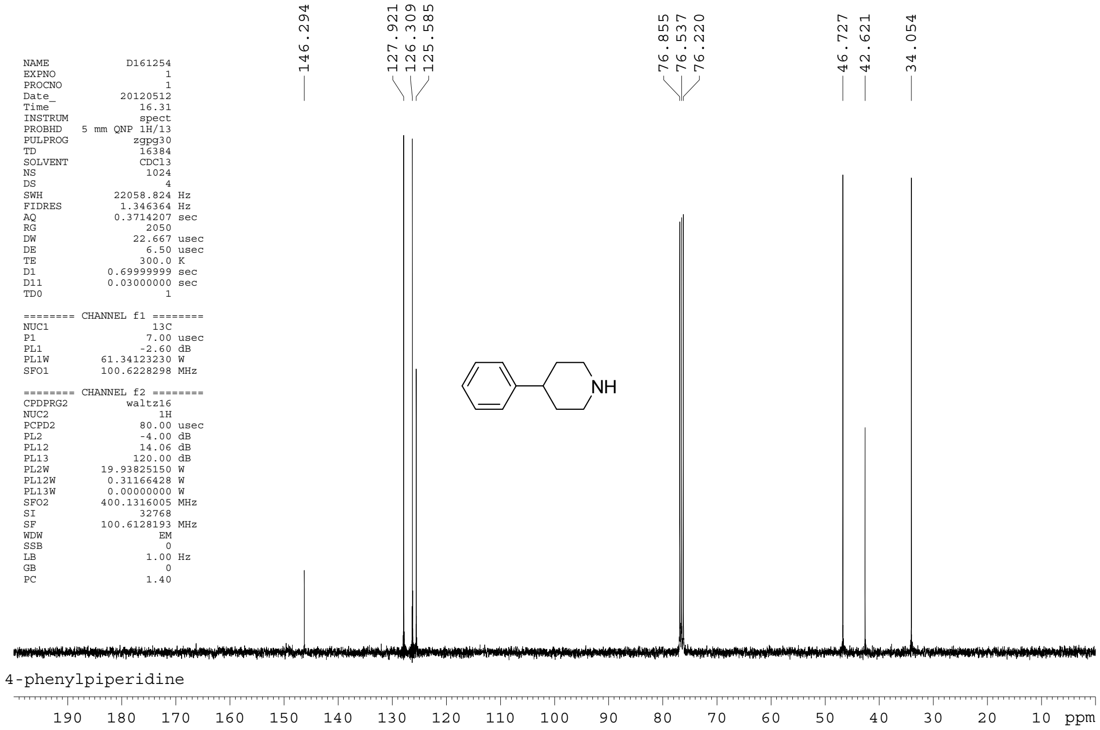
146.294
127.921 126.309 125.585
76.855 76.537 76.220
46.727 42.621 34.054
NAME D161254
EXPNO 1
PROCNO 1
Date_ 20120512
Time 16.31
INSTRUM spect
PROBHD 5 mm QNP 1H/13
PULPROG zgpg30
TD 16384
SOLVENT CDCl3
NS 1024
DS 4
SWH 22058.824 Hz
FIDRES 1.346364 Hz
AQ 0.3714207 sec
RG 2050
DW 22.667 usec
DE 6.50 usec
TE 300.0 K
D1 0.69999999 sec
D11 0.03000000 sec
TD0 1
======== CHANNEL f1 ========
NUC1 13C
P1 7.00 usec
PL1 -2.60 dB
PL1W 61.34123230 W
SFO1 100.6228298 MHz
======== CHANNEL f2 ========
CPDPRG2 waltz16
NUC2 1H
PCPD2 80.00 usec
PL2 -4.00 dB
PL12 14.06 dB
PL13 120.00 dB
PL2W 19.93825150 W
PL12W 0.31166428 W
PL13W 0.00000000 W
SFO2 400.1316005 MHz
SI 32768
SF 100.6128193 MHz
WDW EM
SSB 0
LB 1.00 Hz
GB 0
PC 1.40
NH
4-phenylpiperidine
190 180 170 160 150 140 130 120 110 100 90 80 70 60 50 40 30 20 10 ppm

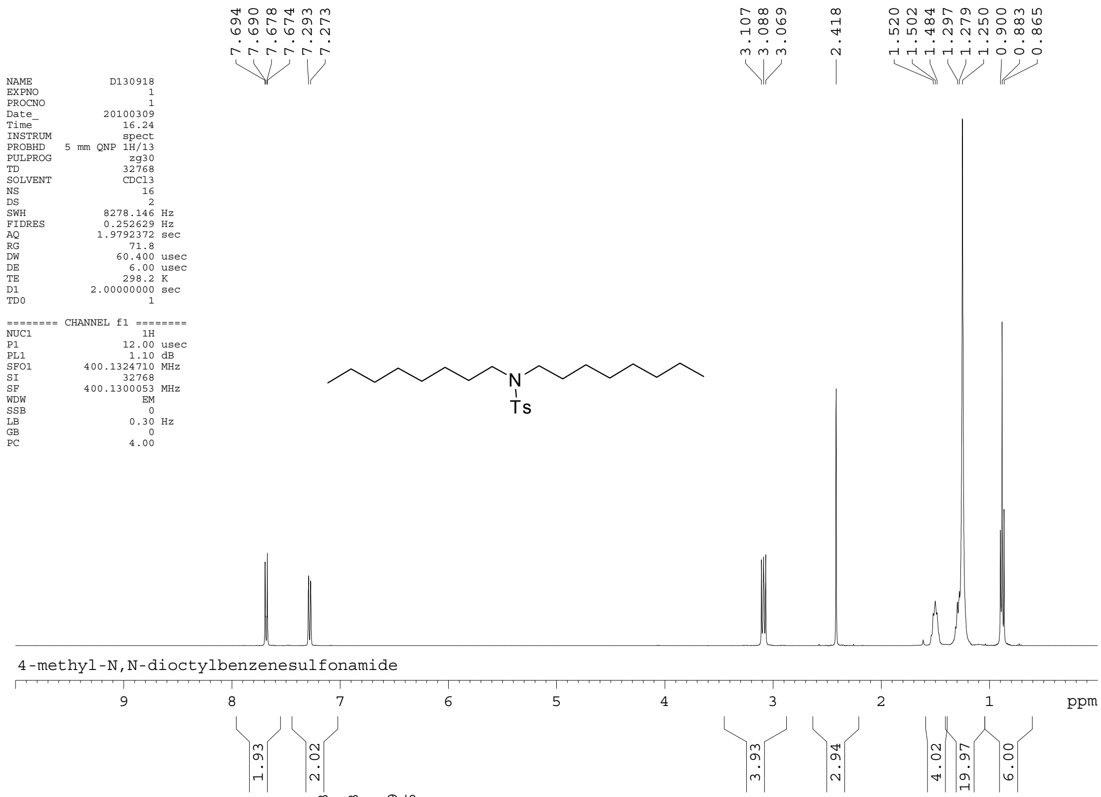
7.694 7.690 7.678 7.674 7.293 7.273
3.107 3.088 3.069
2.418
1.520 1.502 1.484 1.297 1.279 1.250 0.900 0.883 0.865
NAME D130918
EXPNO 1
PROCNO 1
Date_ 20100309
Time 16.24
INSTRUM spect
PROBHD 5 mm QNP 1H/13
PULPROG zg30
TD 32768
SOLVENT CDCl3
NS 16
DS 2
SWH 8278.146 Hz
FIDRES 0.252629 Hz
AQ 1.9792372 sec
RG 71.8
DW 60.400 usec
DE 6.00 usec
TE 298.2 K
D1 2.00000000 sec
TD0 1
======== CHANNEL f1 ========
NUC1 1H
P1 12.00 usec
PL1 1.10 dB
SFO1 400.1324710 MHz
SI 32768
SF 400.1300053 MHz
WDW EM
SSB 0
LB 0.30 Hz
GB 0
PC 4.00
N
Ts
4-methyl-N,N-dioctylbenzenesulfonamide
9 8 7 6 5 4 3 2 1 ppm
1.93 2.02 3.93 2.94 4.02 19.97 6.00

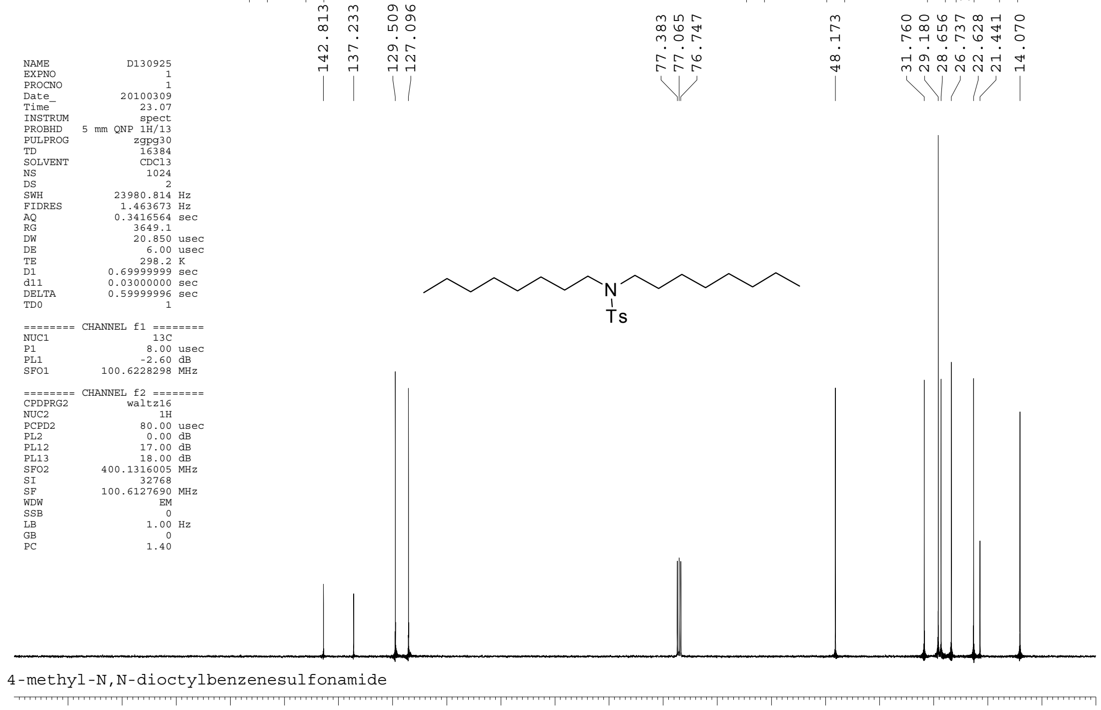
142.813 137.233 129.509 127.096
77.383 77.065 76.747
48.173
31.760 29.180 28.656 26.737 22.628 21.441 14.070
NAME D130925
EXPNO 1
PROCNO 1
Date_ 20100309
Time 23.07
INSTRUM spect
PROBHD 5 mm QNP 1H/13
PULPROG zgpg30
TD 16384
SOLVENT CDCl3
NS 1024
DS 2
SWH 23980.814 Hz
FIDRES 1.463673 Hz
AQ 0.3416564 sec
RG 3649.1
DW 20.850 usec
DE 6.00 usec
TE 298.2 K
D1 0.69999999 sec
d11 0.03000000 sec
DELTA 0.59999996 sec
TD0 1
======== CHANNEL f1 ========
NUC1 13C
P1 8.00 usec
PL1 -2.60 dB
SFO1 100.6228298 MHz
======== CHANNEL f2 ========
CPDPRG2 waltz16
NUC2 1H
PCPD2 80.00 usec
PL2 0.00 dB
PL12 17.00 dB
PL13 18.00 dB
SFO2 400.1316005 MHz
SI 32768
SF 100.6127690 MHz
WDW EM
SSB 0
LB 1.00 Hz
GB 0
PC 1.40
N
Ts
4-methyl-N,N-dioctylbenzenesulfonamide
190 180 170 160 150 140 130 120 110 100 90 80 70 60 50 40 30 20 10 ppm

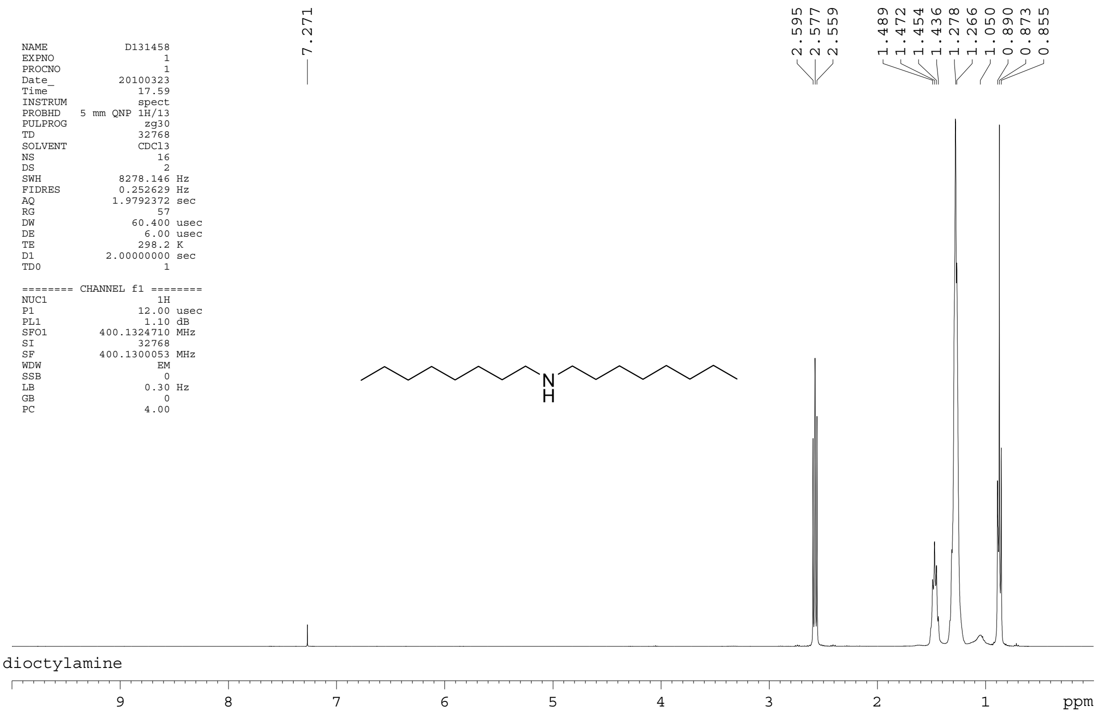
NAME D131458
EXPNO 1
PROCNO 1
Date_ 20100323
Time 17.59
INSTRUM spect
PROBHD 5 mm QNP 1H/13
PULPROG zg30
TD 32768
SOLVENT CDCl3
NS 16
DS 2
SWH 8278.146 Hz
FIDRES 0.252629 Hz
AQ 1.9792372 sec
RG 57
DW 60.400 usec
DE 6.00 usec
TE 298.2 K
D1 2.00000000 sec
TD0 1
======== CHANNEL f1 ========
NUC1 1H
P1 12.00 usec
PL1 1.10 dB
SFO1 400.1324710 MHz
SI 32768
SF 400.1300053 MHz
WDW EM
SSB 0
LB 0.30 Hz
GB 0
PC 4.00
7.271
2.595
2.577
2.559
1.489
1.472
1.454
1.436
1.278
1.266
1.050
0.890
0.873
0.855
N
H
dioctylamine
9 8 7 6 5 4 3 2 1 ppm
4.01
4.20
20.27
1.11
6.00

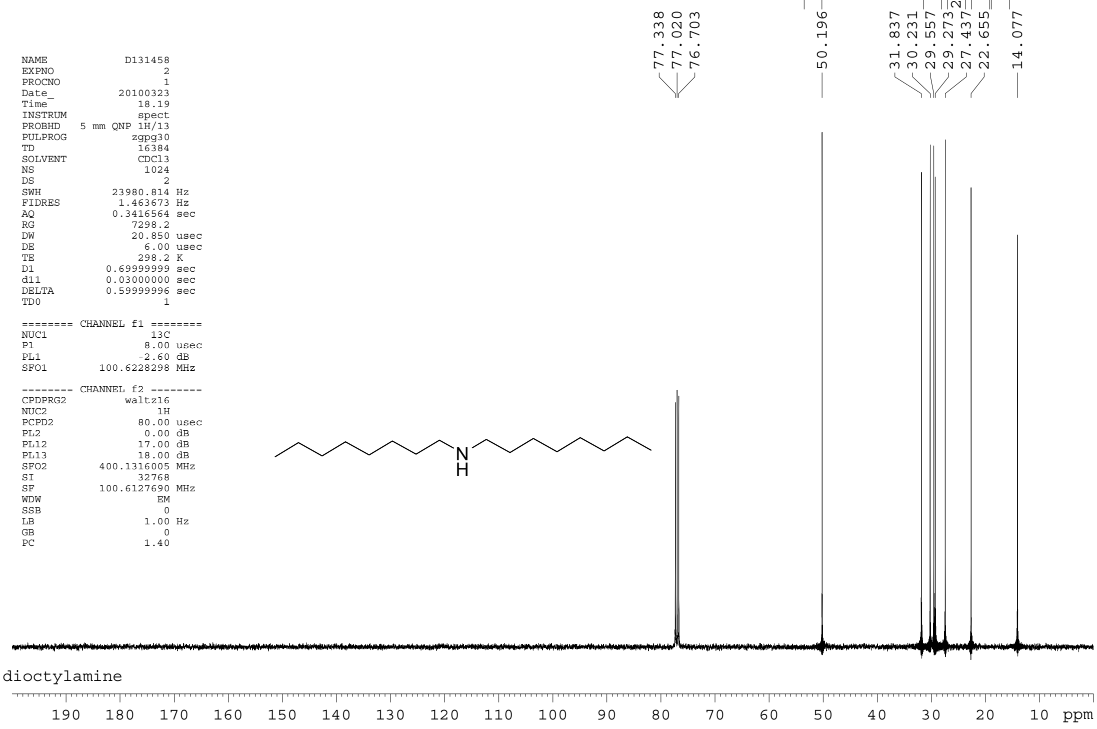
NAME D131458
EXPNO 2
PROCNO 1
Date_ 20100323
Time 18.19
INSTRUM spect
PROBHD 5 mm QNP 1H/13
PULPROG zgpg30
TD 16384
SOLVENT CDCl3
NS 1024
DS 2
SWH 23980.814 Hz
FIDRES 1.463673 Hz
AQ 0.3416564 sec
RG 7298.2
DW 20.850 usec
DE 6.00 usec
TE 298.2 K
D1 0.69999999 sec
d11 0.03000000 sec
DELTA 0.59999996 sec
TD0 1
======== CHANNEL f1 ========
NUC1 13C
P1 8.00 usec
PL1 -2.60 dB
SFO1 100.6228298 MHz
======== CHANNEL f2 ========
CPDPRG2 waltz16
NUC2 1H
PCPD2 80.00 usec
PL2 0.00 dB
PL12 17.00 dB
PL13 18.00 dB
SFO2 400.1316005 MHz
SI 32768
SF 100.6127690 MHz
WDW EM
SSB 0
LB 1.00 Hz
GB 0
PC 1.40
77.338
77.020
76.703
50.196
31.837
30.231
29.557
29.273
27.437
22.655
14.077
N
H
dioctylamine
190 180 170 160 150 140 130 120 110 100 90 80 70 60 50 40 30 20 10 ppm

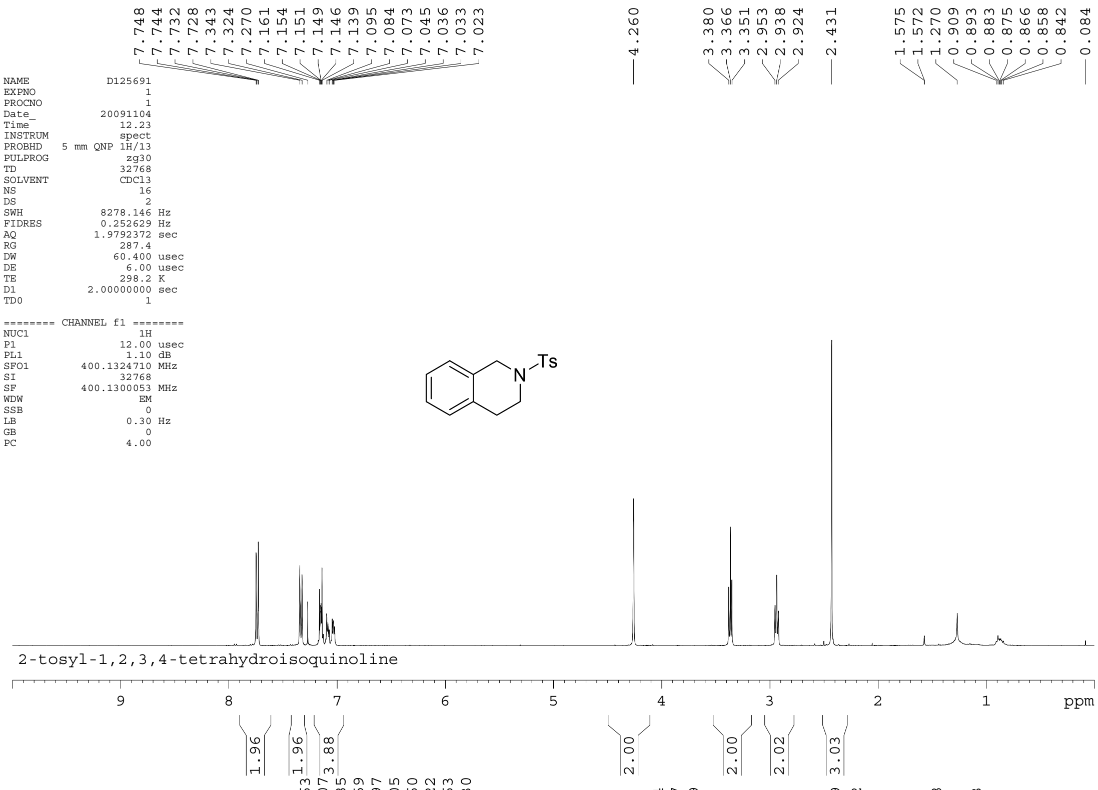
7.748 7.744 7.732 7.728 7.343 7.324 7.270 7.161 7.154 7.151 7.149 7.146 7.139 7.095 7.084 7.073 7.045 7.036 7.033 7.023
4.260
3.380 3.366 3.351 2.953 2.938 2.924
2.431
1.575 1.572 1.270 0.909 0.893 0.883 0.875 0.866 0.858 0.842 0.084
NAME D125691
EXPNO 1
PROCNO 1
Date_ 20091104
Time 12.23
INSTRUM spect
PROBHD 5 mm QNP 1H/13
PULPROG zg30
TD 32768
SOLVENT CDCl3
NS 16
DS 2
SWH 8278.146 Hz
FIDRES 0.252629 Hz
AQ 1.9792372 sec
RG 287.4
DW 60.400 usec
DE 6.00 usec
TE 298.2 K
D1 2.00000000 sec
TD0 1
======== CHANNEL f1 ========
NUC1 1H
P1 12.00 usec
PL1 1.10 dB
SFO1 400.1324710 MHz
SI 32768
SF 400.1300053 MHz
WDW EM
SSB 0
LB 0.30 Hz
GB 0
PC 4.00
N
Ts
2-tosyl-1,2,3,4-tetrahydroisoquinoline
9 8 7 6 5 4 3 2 1 ppm
1.96 1.96 3.88 2.00 2.00 2.02 3.03

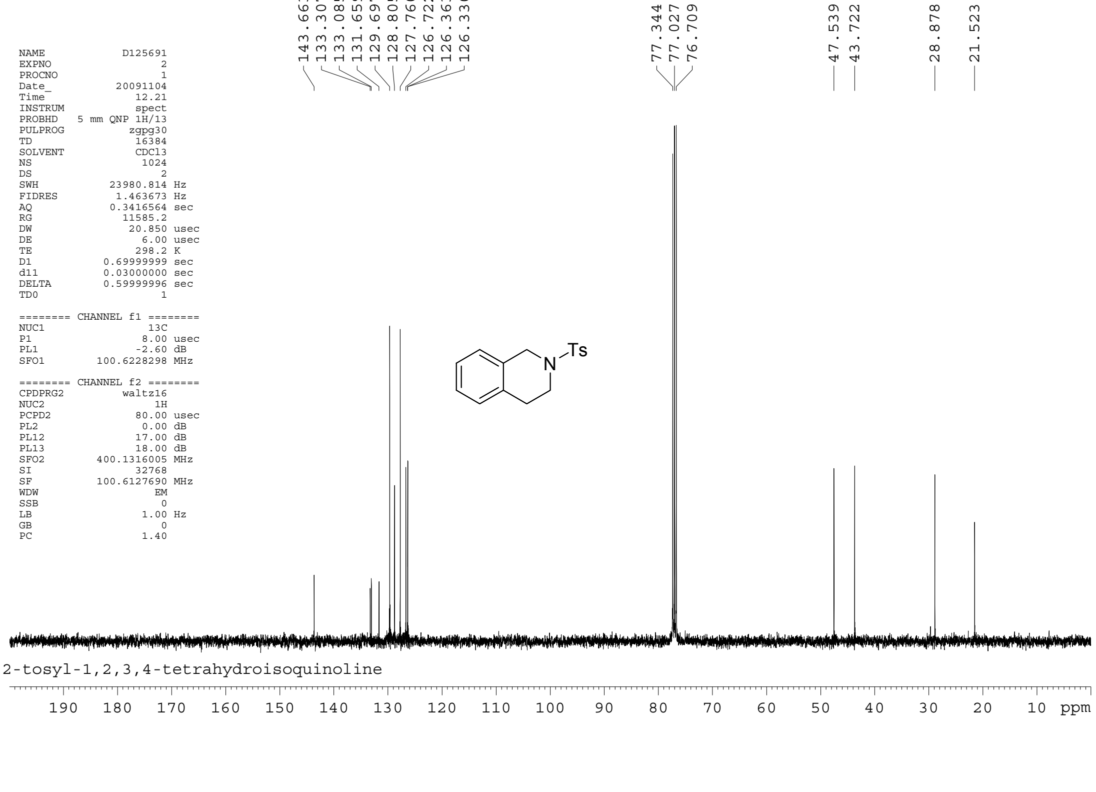
143.663 133.307 133.085 131.659 129.697 128.805 127.760 126.722 126.363 126.330
77.344 77.027 76.709
47.539 43.722
28.878 21.523
NAME D125691
EXPNO 2
PROCNO 1
Date_ 20091104
Time 12.21
INSTRUM spect
PROBHD 5 mm QNP 1H/13
PULPROG zgpg30
TD 16384
SOLVENT CDCl3
NS 1024
DS 2
SWH 23980.814 Hz
FIDRES 1.463673 Hz
AQ 0.3416564 sec
RG 11585.2
DW 20.850 usec
DE 6.00 usec
TE 298.2 K
D1 0.69999999 sec
d11 0.03000000 sec
DELTA 0.59999996 sec
TD0 1
======== CHANNEL f1 ========
NUC1 13C
P1 8.00 usec
PL1 -2.60 dB
SFO1 100.6228298 MHz
======== CHANNEL f2 ========
CPDPRG2 waltz16
NUC2 1H
PCPD2 80.00 usec
PL2 0.00 dB
PL12 17.00 dB
PL13 18.00 dB
SFO2 400.1316005 MHz
SI 32768
SF 100.6127690 MHz
WDW EM
SSB 0
LB 1.00 Hz
GB 0
PC 1.40
N
Ts
2-tosyl-1,2,3,4-tetrahydroisoquinoline
190 180 170 160 150 140 130 120 110 100 90 80 70 60 50 40 30 20 10 ppm

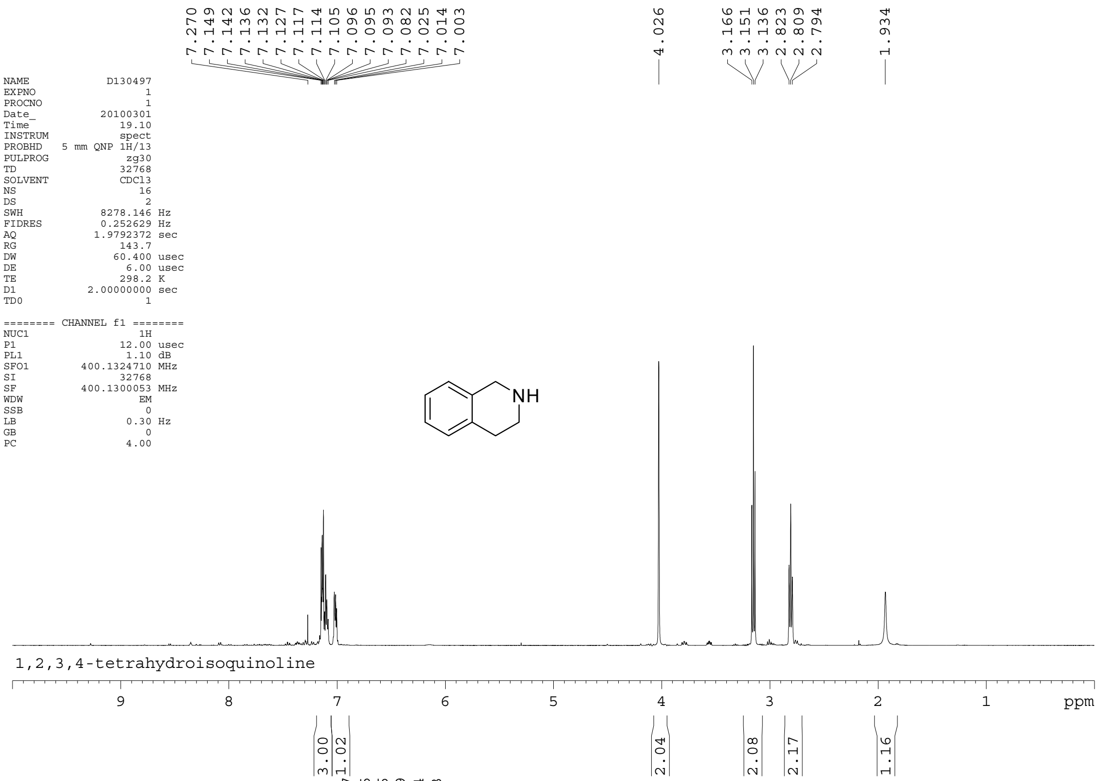
7.270 7.149 7.142 7.136 7.132 7.127 7.117 7.114 7.105 7.096 7.095 7.093 7.082 7.025 7.014 7.003
4.026
3.166 3.151 3.136 2.823 2.809 2.794
1.934
NAME D130497
EXPNO 1
PROCNO 1
Date_ 20100301
Time 19.10
INSTRUM spect
PROBHD 5 mm QNP 1H/13
PULPROG zg30
TD 32768
SOLVENT CDCl3
NS 16
DS 2
SWH 8278.146 Hz
FIDRES 0.252629 Hz
AQ 1.9792372 sec
RG 143.7
DW 60.400 usec
DE 6.00 usec
TE 298.2 K
D1 2.00000000 sec
TD0 1
======== CHANNEL f1 ========
NUC1 1H
P1 12.00 usec
PL1 1.10 dB
SFO1 400.1324710 MHz
SI 32768
SF 400.1300053 MHz
WDW EM
SSB 0
LB 0.30 Hz
GB 0
PC 4.00
NH
1,2,3,4-tetrahydroisoquinoline
9 8 7 6 5 4 3 2 1 ppm
3.00 1.02 2.04 2.08 2.17 1.16

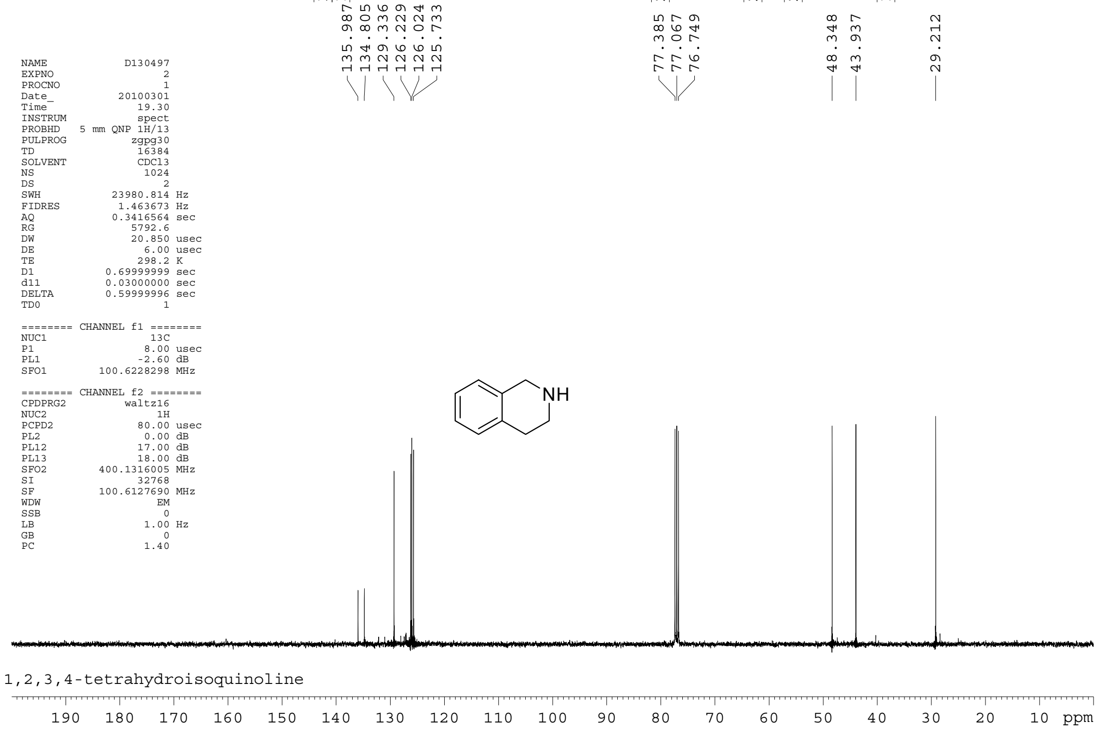
135.987 134.805 129.336 126.229 126.024 125.733
77.385 77.067 76.749
48.348 43.937
29.212
NAME D130497
EXPNO 2
PROCNO 1
Date_ 20100301
Time 19.30
INSTRUM spect
PROBHD 5 mm QNP 1H/13
PULPROG zgpg30
TD 16384
SOLVENT CDCl3
NS 1024
DS 2
SWH 23980.814 Hz
FIDRES 1.463673 Hz
AQ 0.3416564 sec
RG 5792.6
DW 20.850 usec
DE 6.00 usec
TE 298.2 K
D1 0.69999999 sec
d11 0.03000000 sec
DELTA 0.59999996 sec
TD0 1
======== CHANNEL f1 ========
NUC1 13C
P1 8.00 usec
PL1 -2.60 dB
SFO1 100.6228298 MHz
======== CHANNEL f2 ========
CPDPRG2 waltz16
NUC2 1H
PCPD2 80.00 usec
PL2 0.00 dB
PL12 17.00 dB
PL13 18.00 dB
SFO2 400.1316005 MHz
SI 32768
SF 100.6127690 MHz
WDW EM
SSB 0
LB 1.00 Hz
GB 0
PC 1.40
NH
1,2,3,4-tetrahydroisoquinoline
190 180 170 160 150 140 130 120 110 100 90 80 70 60 50 40 30 20 10 ppm

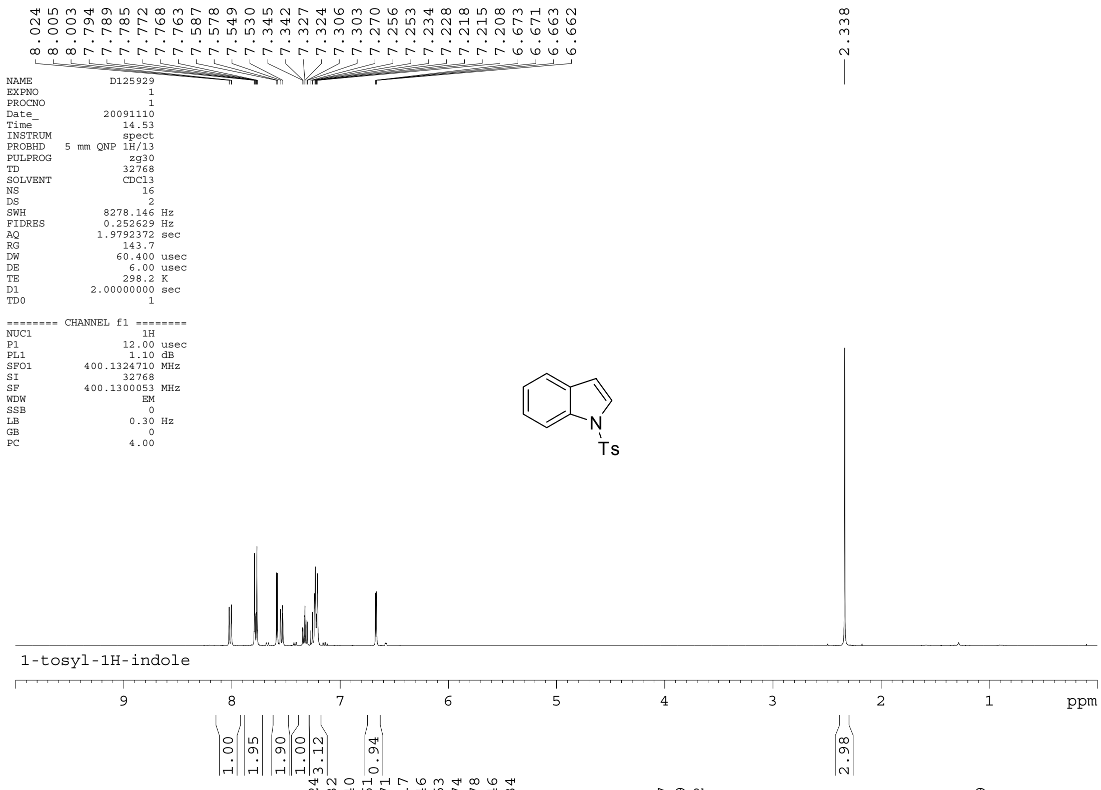
8.024 8.005 8.003 7.794 7.789 7.785 7.772 7.768 7.763 7.587 7.578 7.549 7.530 7.345 7.342 7.327 7.324 7.306 7.303 7.270 7.256 7.253 7.234 7.228 7.218 7.215 7.208 6.673 6.671 6.663 6.662
2.338
NAME D125929
EXPNO 1
PROCNO 1
Date_ 20091110
Time 14.53
INSTRUM spect
PROBHD 5 mm QNP 1H/13
PULPROG zg30
TD 32768
SOLVENT CDCl3
NS 16
DS 2
SWH 8278.146 Hz
FIDRES 0.252629 Hz
AQ 1.9792372 sec
RG 143.7
DW 60.400 usec
DE 6.00 usec
TE 298.2 K
D1 2.00000000 sec
TD0 1
======== CHANNEL f1 ========
NUC1 1H
P1 12.00 usec
PL1 1.10 dB
SFO1 400.1324710 MHz
SI 32768
SF 400.1300053 MHz
WDW EM
SSB 0
LB 0.30 Hz
GB 0
PC 4.00
N
Ts
1-tosyl-1H-indole
9 8 7 6 5 4 3 2 1 ppm
1.00 1.95 1.90 1.00 3.12 0.94 2.98

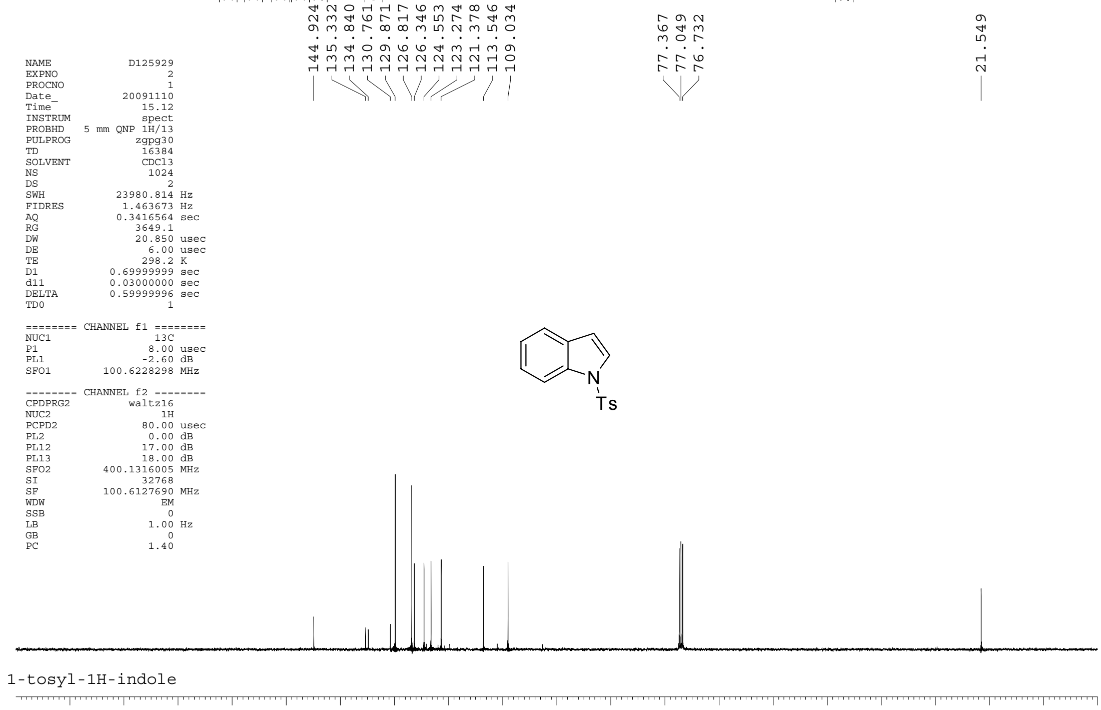
144.924 135.332 134.840 130.761 129.871 126.817 126.346 124.553 123.274 121.378 113.546 109.034
77.367 77.049 76.732
21.549
NAME D125929
EXPNO 2
PROCNO 1
Date_ 20091110
Time 15.12
INSTRUM spect
PROBHD 5 mm QNP 1H/13
PULPROG zgpg30
TD 16384
SOLVENT CDCl3
NS 1024
DS 2
SWH 23980.814 Hz
FIDRES 1.463673 Hz
AQ 0.3416564 sec
RG 3649.1
DW 20.850 usec
DE 6.00 usec
TE 298.2 K
D1 0.69999999 sec
d11 0.03000000 sec
DELTA 0.59999996 sec
TD0 1
======== CHANNEL f1 ========
NUC1 13C
P1 8.00 usec
PL1 -2.60 dB
SFO1 100.6228298 MHz
======== CHANNEL f2 ========
CPDPRG2 waltz16
NUC2 1H
PCPD2 80.00 usec
PL2 0.00 dB
PL12 17.00 dB
PL13 18.00 dB
SFO2 400.1316005 MHz
SI 32768
SF 100.6127690 MHz
WDW EM
SSB 0
LB 1.00 Hz
GB 0
PC 1.40
N
Ts
1-tosyl-1H-indole
190 180 170 160 150 140 130 120 110 100 90 80 70 60 50 40 30 20 10 ppm

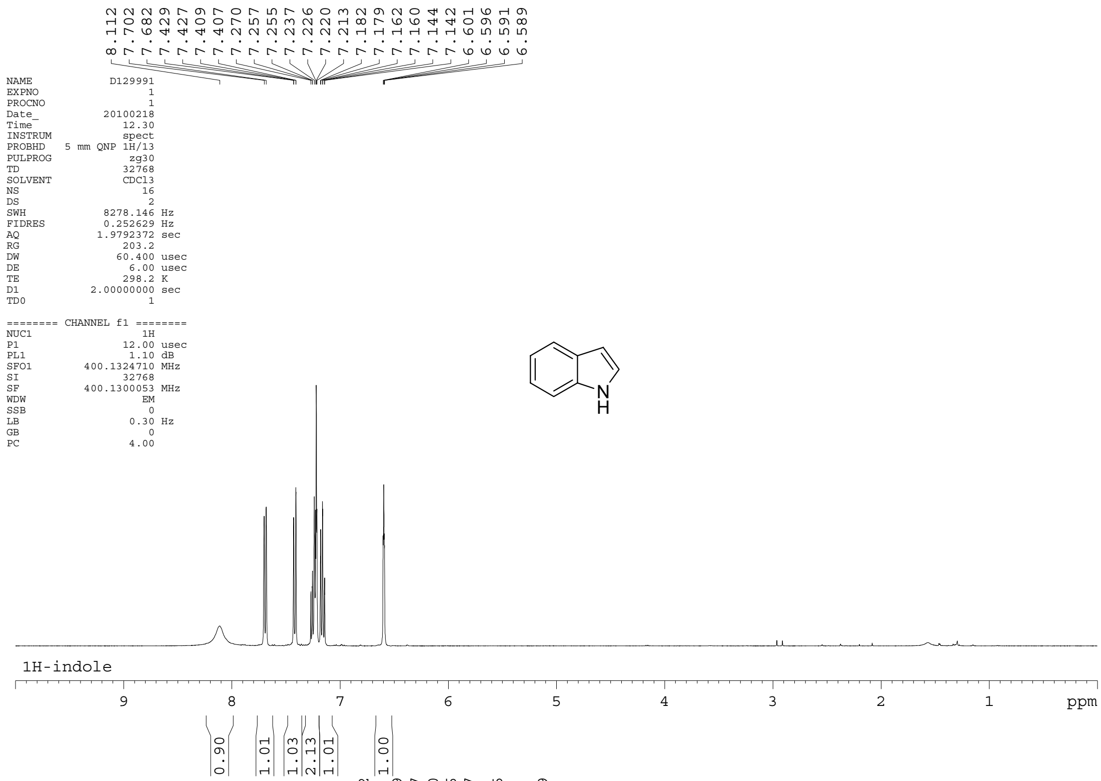
8.112
7.702
7.682
7.429
7.427
7.409
7.407
7.270
7.257
7.255
7.237
7.226
7.220
7.213
7.182
7.179
7.162
7.160
7.144
7.142
6.601
6.596
6.591
6.589
NAME D129991
EXPNO 1
PROCNO 1
Date_ 20100218
Time 12.30
INSTRUM spect
PROBHD 5 mm QNP 1H/13
PULPROG zg30
TD 32768
SOLVENT CDCl3
NS 16
DS 2
SWH 8278.146 Hz
FIDRES 0.252629 Hz
AQ 1.9792372 sec
RG 203.2
DW 60.400 usec
DE 6.00 usec
TE 298.2 K
D1 2.00000000 sec
TD0 1
======== CHANNEL f1 ========
NUC1 1H
P1 12.00 usec
PL1 1.10 dB
SFO1 400.1324710 MHz
SI 32768
SF 400.1300053 MHz
WDW EM
SSB 0
LB 0.30 Hz
GB 0
PC 4.00
N
H
1H-indole
9
8
7
6
5
4
3
2
1
ppm
0.90
1.01
1.03
2.13
1.01
1.00

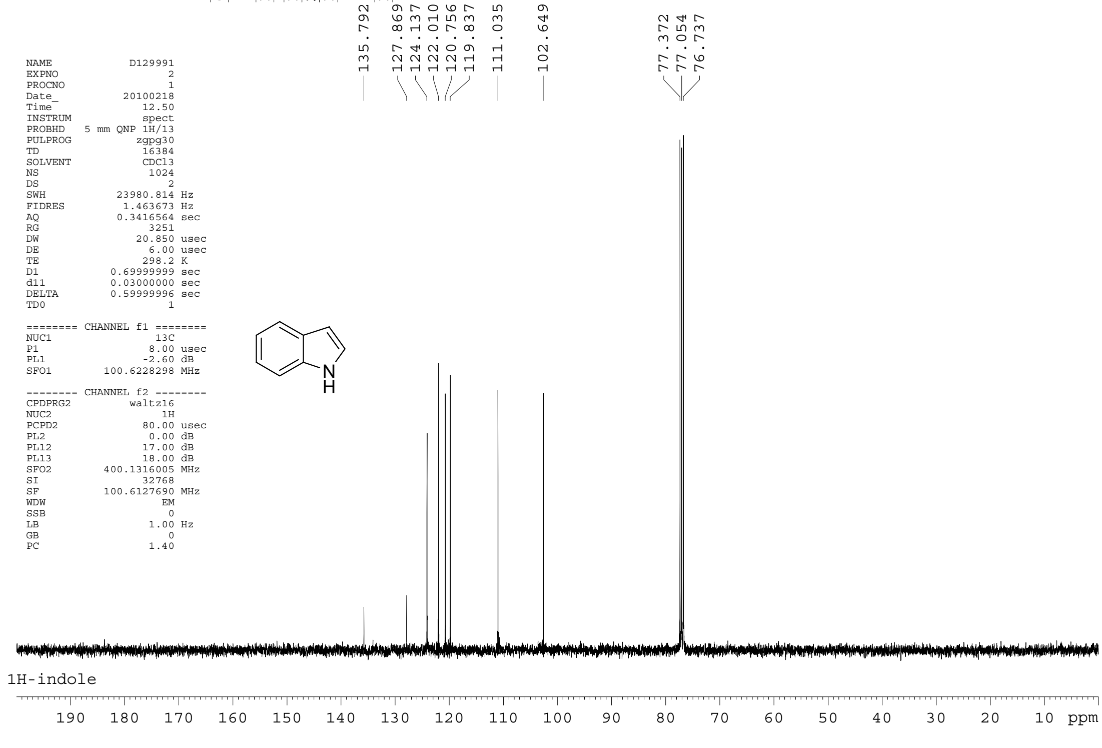
135.792
127.869
124.137
122.010
120.756
119.837
111.035
102.649
77.372
77.054
76.737
NAME D129991
EXPNO 2
PROCNO 1
Date_ 20100218
Time 12.50
INSTRUM spect
PROBHD 5 mm QNP 1H/13
PULPROG zgpg30
TD 16384
SOLVENT CDCl3
NS 1024
DS 2
SWH 23980.814 Hz
FIDRES 1.463673 Hz
AQ 0.3416564 sec
RG 3251
DW 20.850 usec
DE 6.00 usec
TE 298.2 K
D1 0.69999999 sec
d11 0.03000000 sec
DELTA 0.59999996 sec
TD0 1
======== CHANNEL f1 ========
NUC1 13C
P1 8.00 usec
PL1 -2.60 dB
SFO1 100.6228298 MHz
======== CHANNEL f2 ========
CPDPRG2 waltz16
NUC2 1H
PCPD2 80.00 usec
PL2 0.00 dB
PL12 17.00 dB
PL13 18.00 dB
SFO2 400.1316005 MHz
SI 32768
SF 100.6127690 MHz
WDW EM
SSB 0
LB 1.00 Hz
GB 0
PC 1.40
N
H
1H-indole
190
180
170
160
150
140
130
120
110
100
90
80
70
60
50
40
30
20
10
ppm

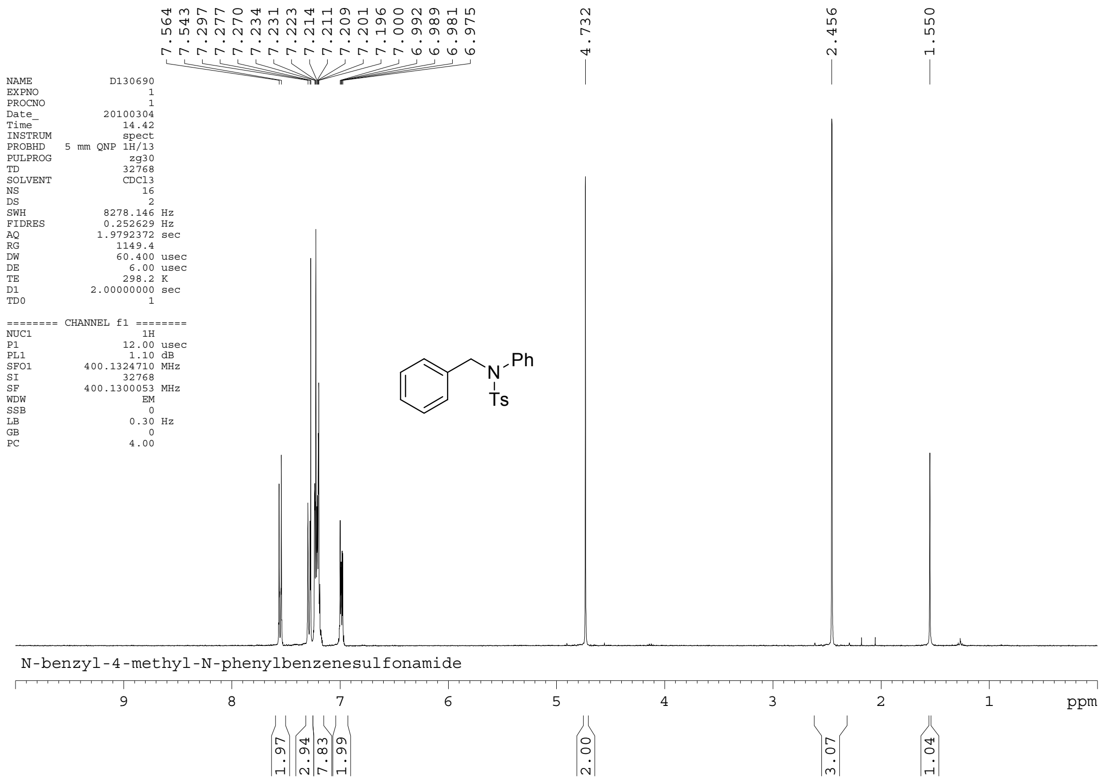
7.564
7.543
7.297
7.277
7.270
7.234
7.231
7.223
7.214
7.211
7.209
7.201
7.196
7.000
6.992
6.989
6.981
6.975
4.732
2.456
1.550
NAME D130690
EXPNO 1
PROCNO 1
Date_ 20100304
Time 14.42
INSTRUM spect
PROBHD 5 mm QNP 1H/13
PULPROG zg30
TD 32768
SOLVENT CDCl3
NS 16
DS 2
SWH 8278.146 Hz
FIDRES 0.252629 Hz
AQ 1.9792372 sec
RG 1149.4
DW 60.400 usec
DE 6.00 usec
TE 298.2 K
D1 2.00000000 sec
TD0 1
======== CHANNEL f1 ========
NUC1 1H
P1 12.00 usec
PL1 1.10 dB
SFO1 400.1324710 MHz
SI 32768
SF 400.1300053 MHz
WDW EM
SSB 0
LB 0.30 Hz
GB 0
PC 4.00
Ph
N
Ts
N-benzyl-4-methyl-N-phenylbenzenesulfonamide
9 8 7 6 5 4 3 2 1 ppm
1.97
2.94
7.83
1.99
2.00
3.07
1.04

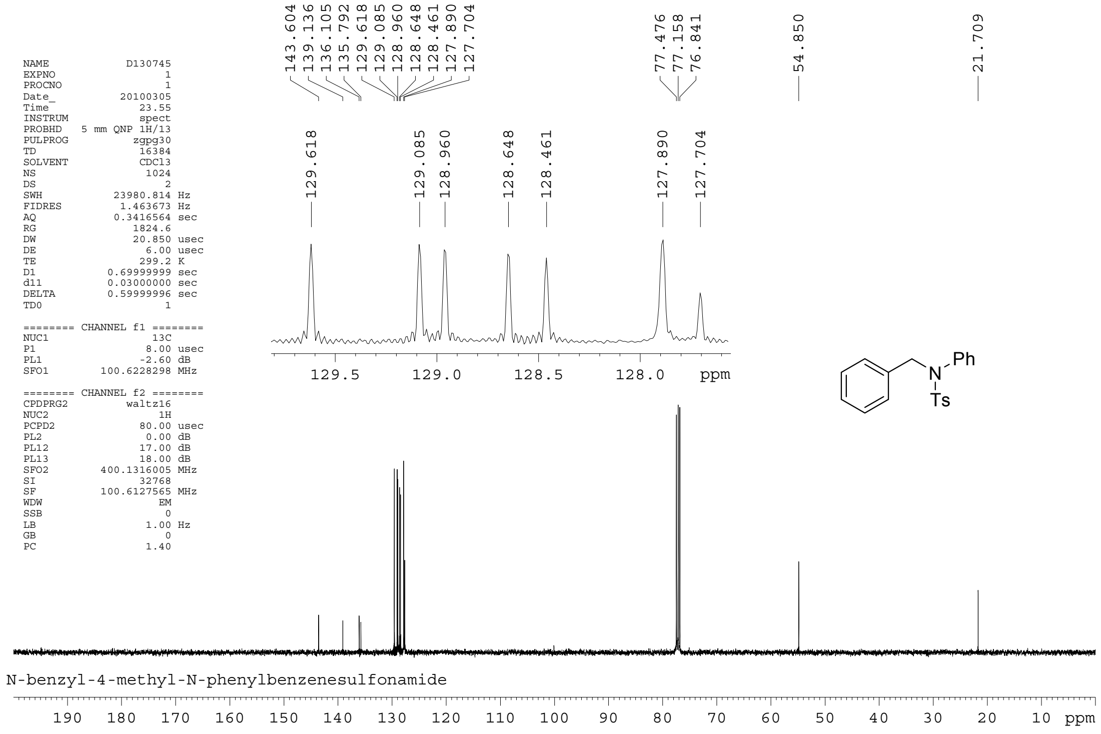
143.604
139.136
136.105
135.792
129.618
129.085
128.960
128.648
128.461
127.890
127.704
77.476
77.158
76.841
54.850
21.709
NAME D130745
EXPNO 1
PROCNO 1
Date_ 20100305
Time 23.55
INSTRUM spect
PROBHD 5 mm QNP 1H/13
PULPROG zgpg30
TD 16384
SOLVENT CDCl3
NS 1024
DS 2
SWH 23980.814 Hz
FIDRES 1.463673 Hz
AQ 0.3416564 sec
RG 1824.6
DW 20.850 usec
DE 6.00 usec
TE 299.2 K
D1 0.69999999 sec
d11 0.03000000 sec
DELTA 0.59999996 sec
TD0 1
======== CHANNEL f1 ========
NUC1 13C
P1 8.00 usec
PL1 -2.60 dB
SFO1 100.6228298 MHz
======== CHANNEL f2 ========
CPDPRG2 waltz16
NUC2 1H
PCPD2 80.00 usec
PL2 0.00 dB
PL12 17.00 dB
PL13 18.00 dB
SFO2 400.1316005 MHz
SI 32768
SF 100.6127565 MHz
WDW EM
SSB 0
LB 1.00 Hz
GB 0
PC 1.40
129.618
129.085
128.960
128.648
128.461
127.890
127.704
129.5 129.0 128.5 128.0 ppm
Ph
N
Ts
N-benzyl-4-methyl-N-phenylbenzenesulfonamide
190 180 170 160 150 140 130 120 110 100 90 80 70 60 50 40 30 20 10 ppm

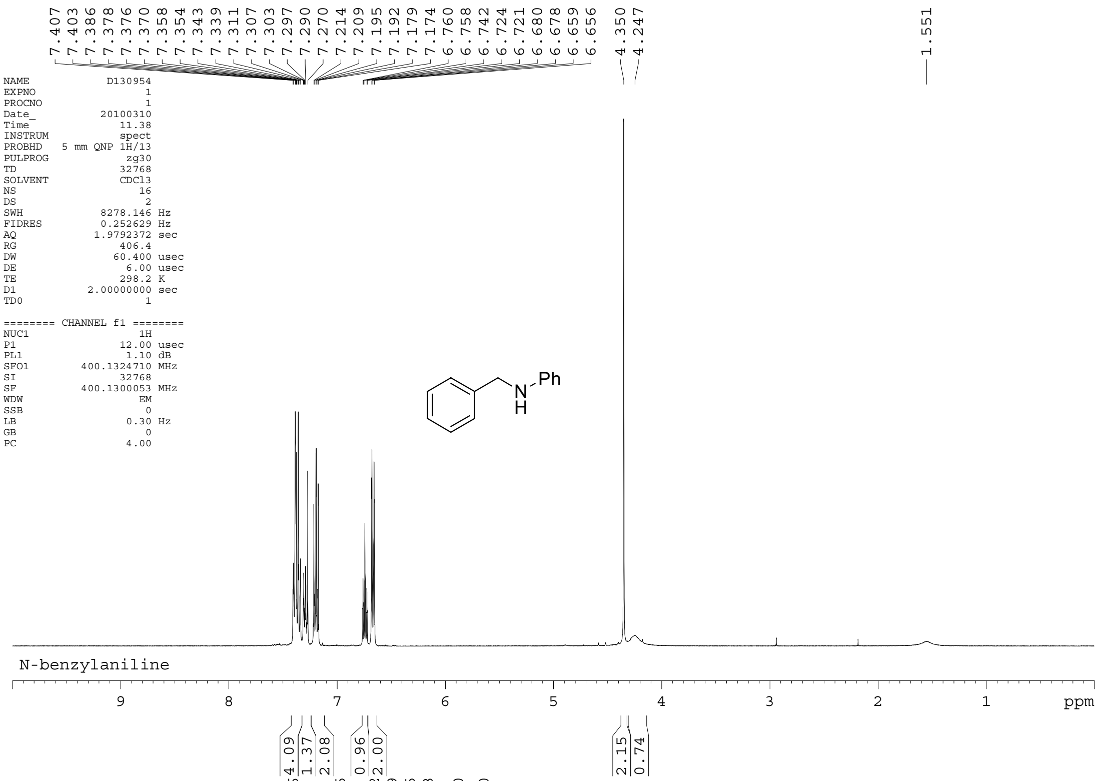
7.407 7.403 7.386 7.378 7.376 7.370 7.358 7.354 7.343 7.339 7.311 7.307 7.303 7.297 7.290 7.270 7.214 7.209 7.195 7.192 7.179 7.174 6.760 6.758 6.742 6.724 6.721 6.680 6.678 6.659 6.656 4.350 4.247 1.551
NAME D130954
EXPNO 1
PROCNO 1
Date_ 20100310
Time 11.38
INSTRUM spect
PROBHD 5 mm QNP 1H/13
PULPROG zg30
TD 32768
SOLVENT CDCl3
NS 16
DS 2
SWH 8278.146 Hz
FIDRES 0.252629 Hz
AQ 1.9792372 sec
RG 406.4
DW 60.400 usec
DE 6.00 usec
TE 298.2 K
D1 2.00000000 sec
TD0 1
======== CHANNEL f1 ========
NUC1 1H
P1 12.00 usec
PL1 1.10 dB
SFO1 400.1324710 MHz
SI 32768
SF 400.1300053 MHz
WDW EM
SSB 0
LB 0.30 Hz
GB 0
PC 4.00
N-Ph
H
N-benzylaniline
9 8 7 6 5 4 3 2 1 ppm
4.09 1.37 2.08 0.96 2.00 2.15 0.74

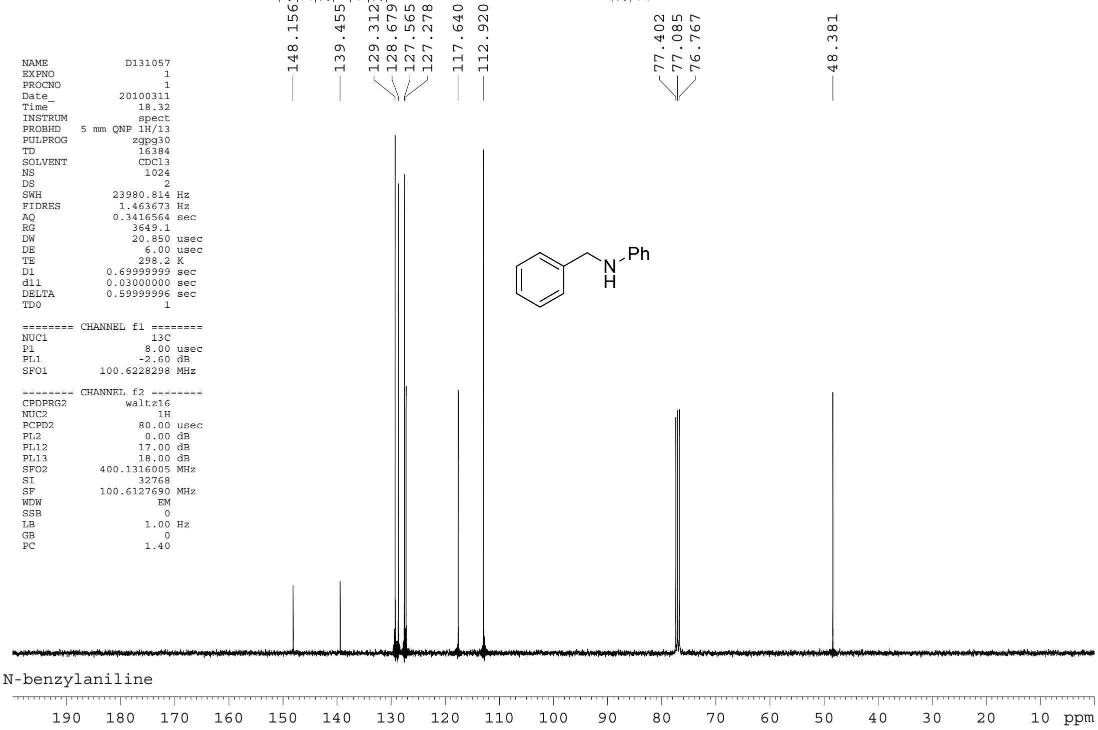
148.156 139.455 129.312 128.679 127.565 127.278 117.640 112.920 77.402 77.085 76.767 48.381
NAME D131057
EXPNO 1
PROCNO 1
Date_ 20100311
Time 18.32
INSTRUM spect
PROBHD 5 mm QNP 1H/13
PULPROG zgpg30
TD 16384
SOLVENT CDCl3
NS 1024
DS 2
SWH 23980.814 Hz
FIDRES 1.463673 Hz
AQ 0.3416564 sec
RG 3649.1
DW 20.850 usec
DE 6.00 usec
TE 298.2 K
D1 0.69999999 sec
d11 0.03000000 sec
DELTA 0.59999996 sec
TD0 1
======== CHANNEL f1 ========
NUC1 13C
P1 8.00 usec
PL1 -2.60 dB
SFO1 100.6228298 MHz
======== CHANNEL f2 ========
CPDPRG2 waltz16
NUC2 1H
PCPD2 80.00 usec
PL2 0.00 dB
PL12 17.00 dB
PL13 18.00 dB
SFO2 400.1316005 MHz
SI 32768
SF 100.6127690 MHz
WDW EM
SSB 0
LB 1.00 Hz
GB 0
PC 1.40
N-Ph
H
N-benzylaniline
190 180 170 160 150 140 130 120 110 100 90 80 70 60 50 40 30 20 10 ppm

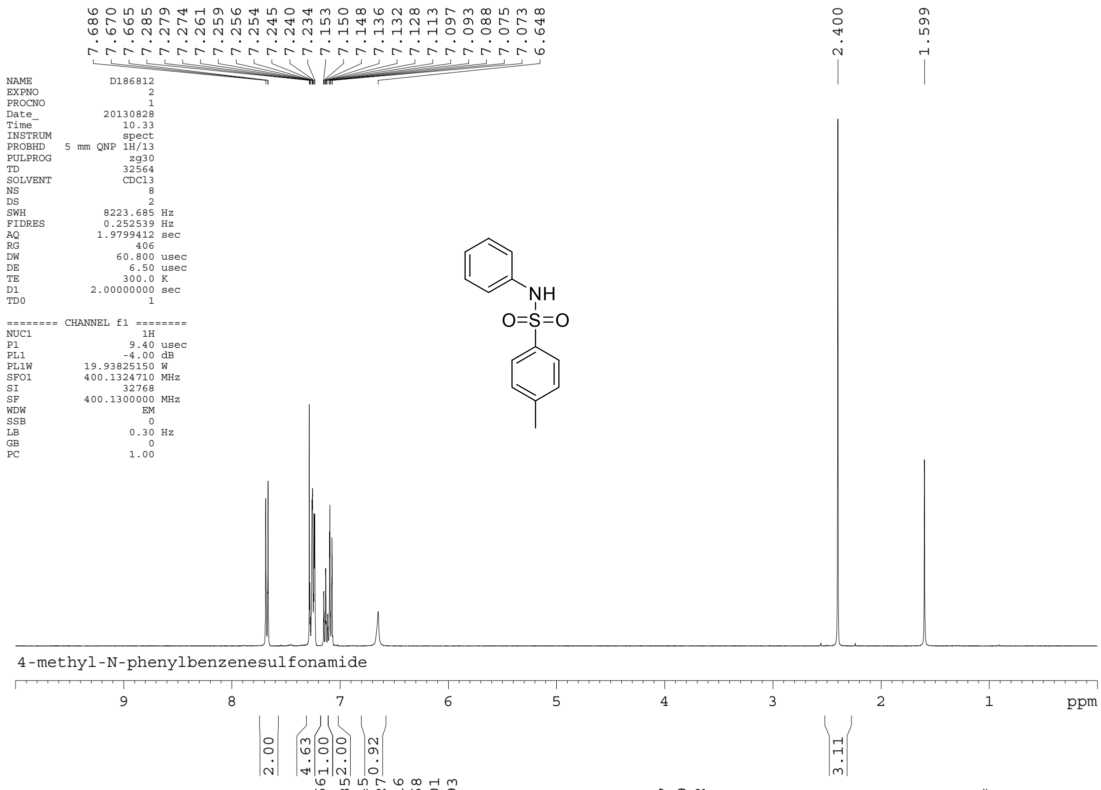
7.686
7.670
7.665
7.285
7.279
7.274
7.261
7.259
7.256
7.254
7.245
7.240
7.234
7.153
7.150
7.148
7.136
7.132
7.128
7.113
7.097
7.093
7.088
7.075
7.073
6.648
2.400
1.599
NAME D186812
EXPNO 2
PROCNO 1
Date_ 20130828
Time 10.33
INSTRUM spect
PROBHD 5 mm QNP 1H/13
PULPROG zg30
TD 32564
SOLVENT CDCl3
NS 8
DS 2
SWH 8223.685 Hz
FIDRES 0.252539 Hz
AQ 1.9799412 sec
RG 406
DW 60.800 usec
DE 6.50 usec
TE 300.0 K
D1 2.00000000 sec
TD0 1
======== CHANNEL f1 ========
NUC1 1H
P1 9.40 usec
PL1 -4.00 dB
PL1W 19.93825150 W
SFO1 400.1324710 MHz
SI 32768
SF 400.1300000 MHz
WDW EM
SSB 0
LB 0.30 Hz
GB 0
PC 1.00
NH
O=S=O
4-methyl-N-phenylbenzenesulfonamide
9
8
7
6
5
4
3
2
1
ppm
2.00
4.63
1.00
2.00
0.92
3.11

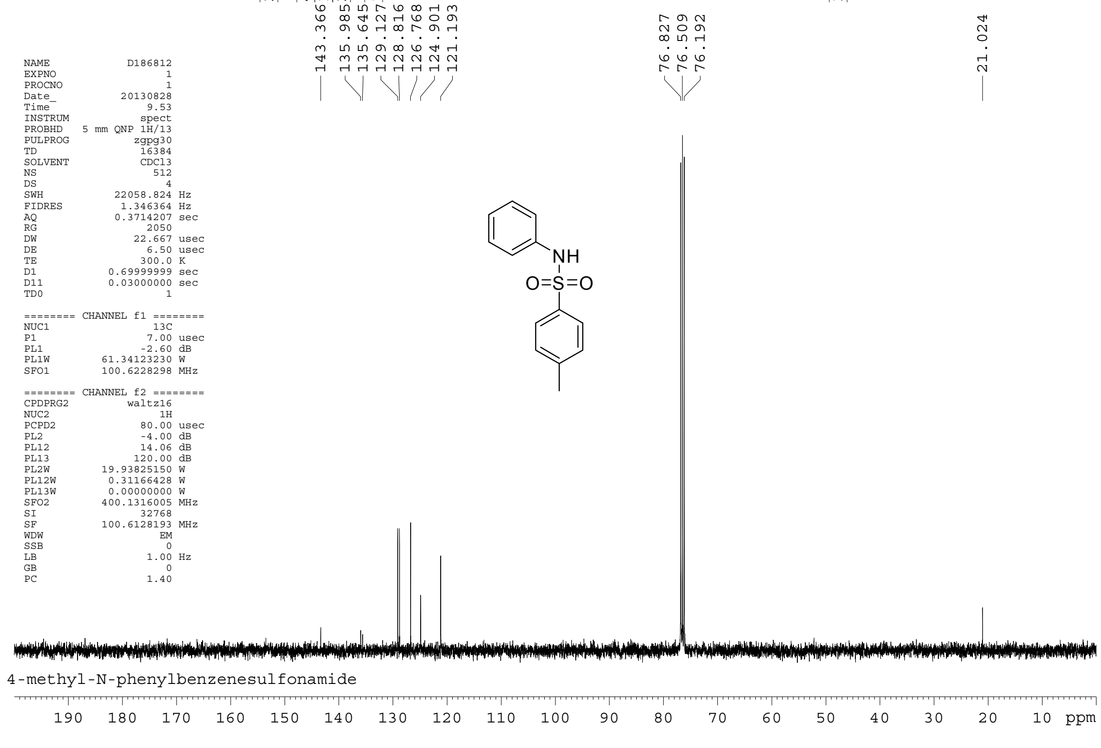
143.366
135.985
135.645
129.127
128.816
126.768
124.901
121.193
76.827
76.509
76.192
21.024
NAME D186812
EXPNO 1
PROCNO 1
Date_ 20130828
Time 9.53
INSTRUM spect
PROBHD 5 mm QNP 1H/13
PULPROG zgpg30
TD 16384
SOLVENT CDCl3
NS 512
DS 4
SWH 22058.824 Hz
FIDRES 1.346364 Hz
AQ 0.3714207 sec
RG 2050
DW 22.667 usec
DE 6.50 usec
TE 300.0 K
D1 0.69999999 sec
D11 0.03000000 sec
TD0 1
======== CHANNEL f1 ========
NUC1 13C
P1 7.00 usec
PL1 -2.60 dB
PL1W 61.34123230 W
SFO1 100.6228298 MHz
======== CHANNEL f2 ========
CPDPRG2 waltz16
NUC2 1H
PCPD2 80.00 usec
PL2 -4.00 dB
PL12 14.06 dB
PL13 120.00 dB
PL2W 19.93825150 W
PL12W 0.31166428 W
PL13W 0.00000000 W
SFO2 400.1316005 MHz
SI 32768
SF 100.6128193 MHz
WDW EM
SSB 0
LB 1.00 Hz
GB 0
PC 1.40
NH
O=S=O
4-methyl-N-phenylbenzenesulfonamide
190
180
170
160
150
140
130
120
110
100
90
80
70
60
50
40
30
20
10
ppm

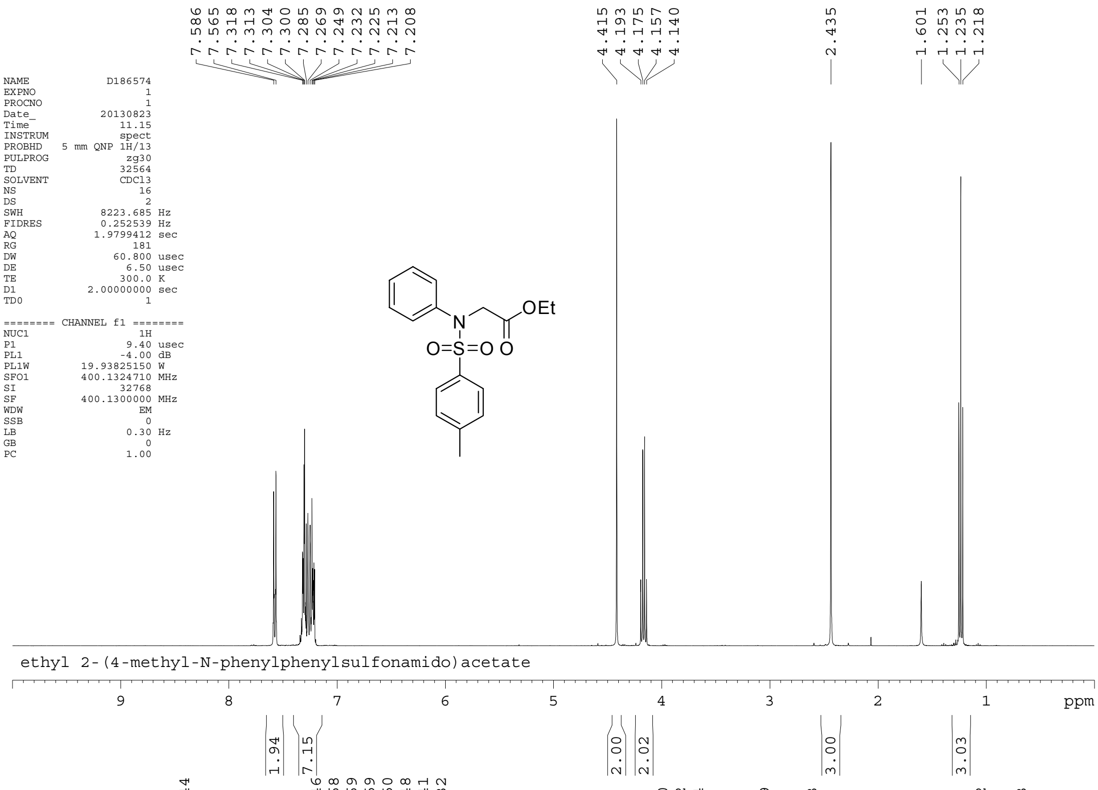
7.586 7.565 7.318 7.313 7.304 7.300 7.285 7.269 7.249 7.232 7.225 7.213 7.208
4.415 4.193 4.175 4.157 4.140
2.435
1.601 1.253 1.235 1.218
NAME D186574
EXPNO 1
PROCNO 1
Date_ 20130823
Time 11.15
INSTRUM spect
PROBHD 5 mm QNP 1H/13
PULPROG zg30
TD 32564
SOLVENT CDCl3
NS 16
DS 2
SWH 8223.685 Hz
FIDRES 0.252539 Hz
AQ 1.9799412 sec
RG 181
DW 60.800 usec
DE 6.50 usec
TE 300.0 K
D1 2.00000000 sec
TD0 1
======== CHANNEL f1 ========
NUC1 1H
P1 9.40 usec
PL1 -4.00 dB
PL1W 19.93825150 W
SFO1 400.1324710 MHz
SI 32768
SF 400.1300000 MHz
WDW EM
SSB 0
LB 0.30 Hz
GB 0
PC 1.00
N
OEt
O=S=O
O
ethyl 2-(4-methyl-N-phenylphenylsulfonamido)acetate
9 8 7 6 5 4 3 2 1 ppm
1.94 7.15 2.00 2.02 3.00 3.03

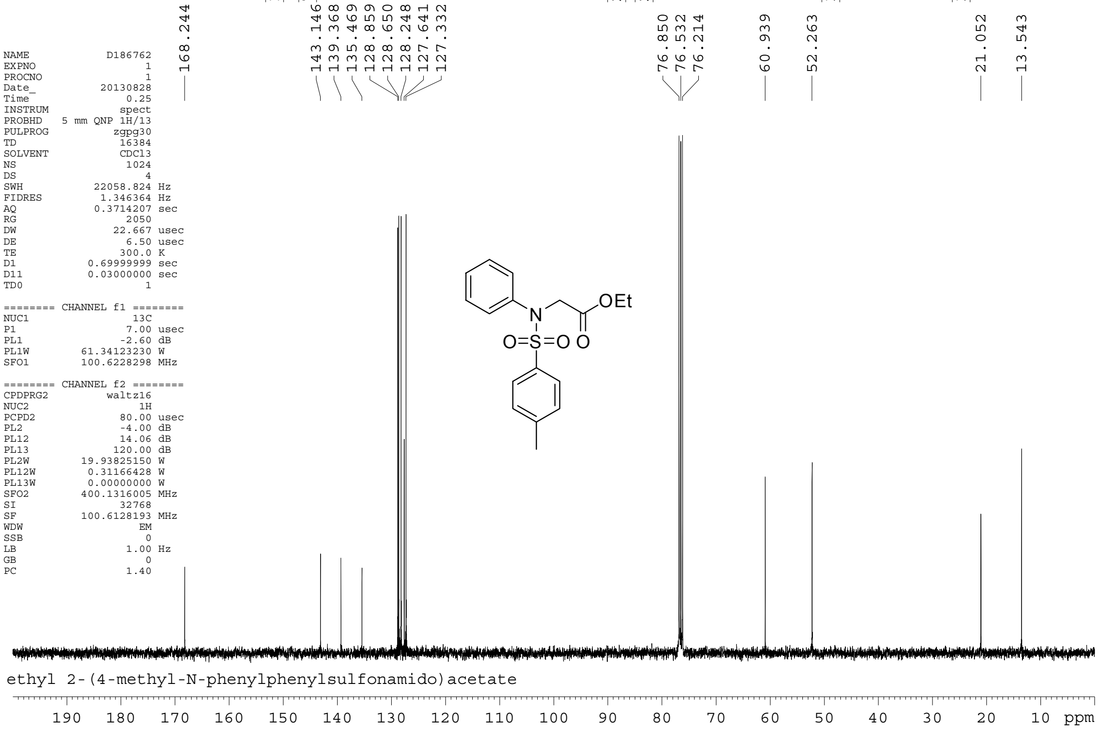
168.244
143.146 139.368 135.469 128.859 128.650 128.248 127.641 127.332
76.850 76.532 76.214
60.939
52.263
21.052
13.543
NAME D186762
EXPNO 1
PROCNO 1
Date_ 20130828
Time 0.25
INSTRUM spect
PROBHD 5 mm QNP 1H/13
PULPROG zgpg30
TD 16384
SOLVENT CDCl3
NS 1024
DS 4
SWH 22058.824 Hz
FIDRES 1.346364 Hz
AQ 0.3714207 sec
RG 2050
DW 22.667 usec
DE 6.50 usec
TE 300.0 K
D1 0.69999999 sec
D11 0.03000000 sec
TD0 1
======== CHANNEL f1 ========
NUC1 13C
P1 7.00 usec
PL1 -2.60 dB
PL1W 61.34123230 W
SFO1 100.6228298 MHz
======== CHANNEL f2 ========
CPDPRG2 waltz16
NUC2 1H
PCPD2 80.00 usec
PL2 -4.00 dB
PL12 14.06 dB
PL13 120.00 dB
PL2W 19.93825150 W
PL12W 0.31166428 W
PL13W 0.00000000 W
SFO2 400.1316005 MHz
SI 32768
SF 100.6128193 MHz
WDW EM
SSB 0
LB 1.00 Hz
GB 0
PC 1.40
N
OEt
O=S=O
O
ethyl 2-(4-methyl-N-phenylphenylsulfonamido)acetate
190 180 170 160 150 140 130 120 110 100 90 80 70 60 50 40 30 20 10 ppm

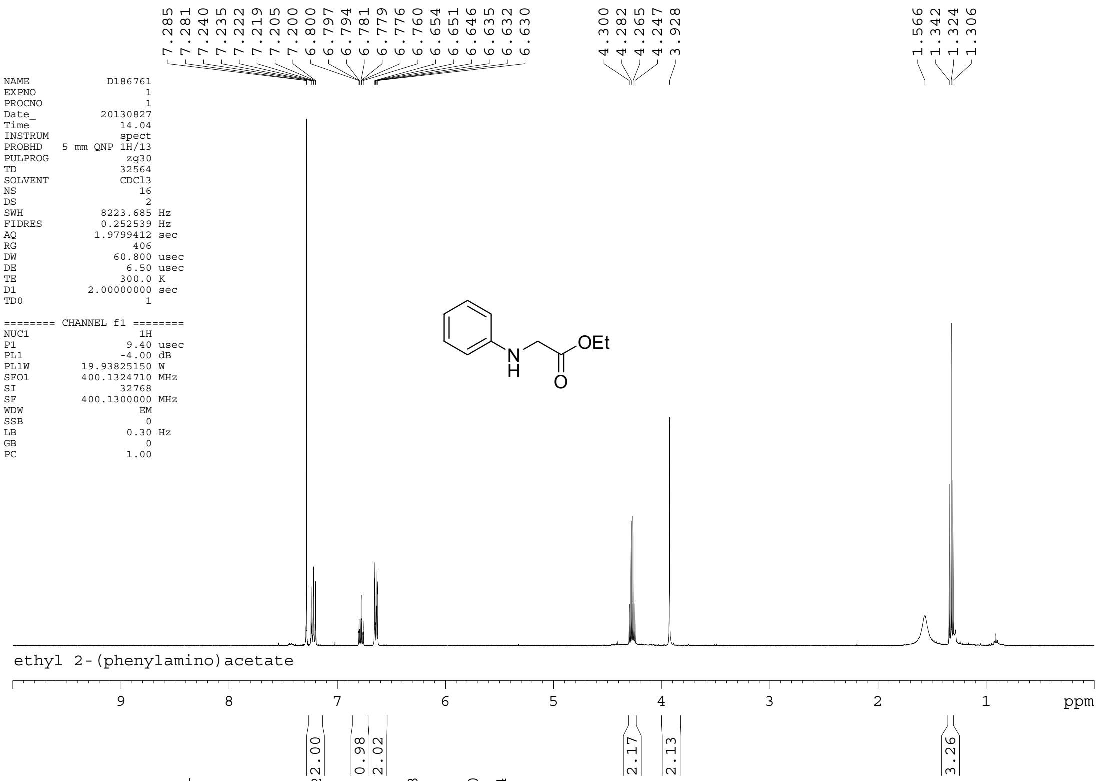
7.285
7.281
7.240
7.235
7.222
7.219
7.205
7.200
6.800
6.797
6.794
6.781
6.779
6.776
6.760
6.654
6.651
6.646
6.635
6.632
6.630
4.300
4.282
4.265
4.247
3.928
1.566
1.342
1.324
1.306
NAME D186761
EXPNO 1
PROCNO 1
Date_ 20130827
Time 14.04
INSTRUM spect
PROBHD 5 mm QNP 1H/13
PULPROG zg30
TD 32564
SOLVENT CDCl3
NS 16
DS 2
SWH 8223.685 Hz
FIDRES 0.252539 Hz
AQ 1.9799412 sec
RG 406
DW 60.800 usec
DE 6.50 usec
TE 300.0 K
D1 2.00000000 sec
TD0 1
======== CHANNEL f1 ========
NUC1 1H
P1 9.40 usec
PL1 -4.00 dB
PL1W 19.93825150 W
SFO1 400.1324710 MHz
SI 32768
SF 400.1300000 MHz
WDW EM
SSB 0
LB 0.30 Hz
GB 0
PC 1.00
OEt
N
H
O
ethyl 2-(phenylamino)acetate
9
8
7
6
5
4
3
2
1
ppm
2.00
0.98
2.02
2.17
2.13
3.26

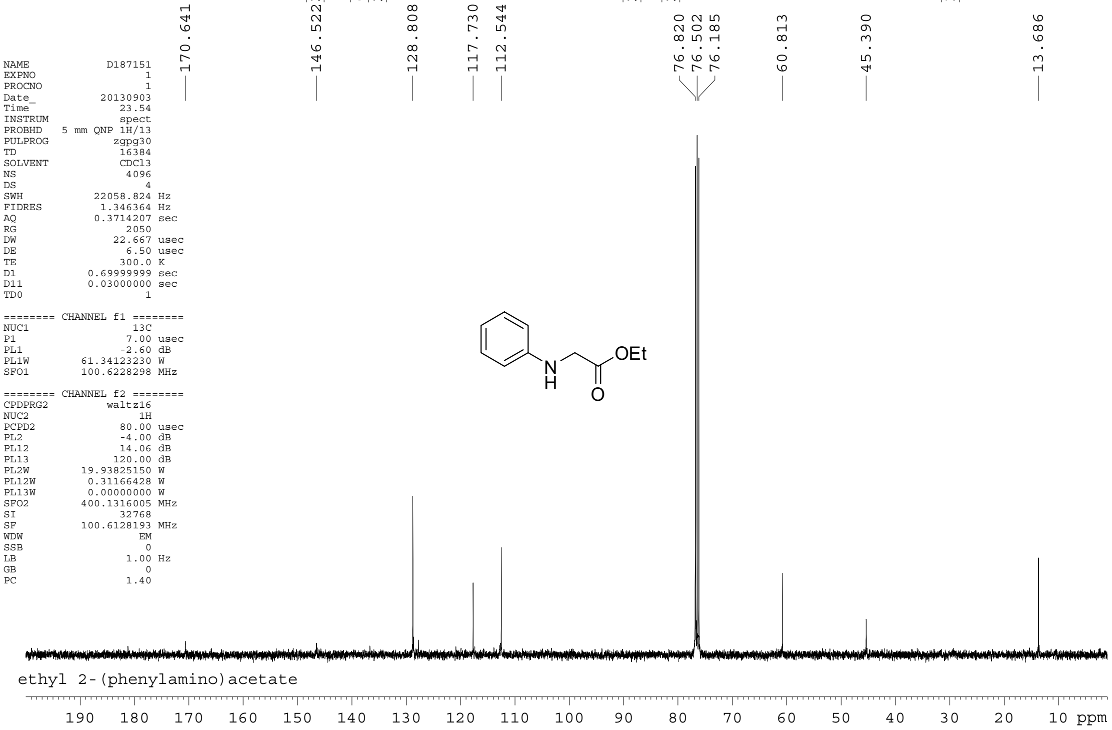
170.641
146.522
128.808
117.730
112.544
76.820
76.502
76.185
60.813
45.390
13.686
NAME D187151
EXPNO 1
PROCNO 1
Date_ 20130903
Time 23.54
INSTRUM spect
PROBHD 5 mm QNP 1H/13
PULPROG zgpg30
TD 16384
SOLVENT CDCl3
NS 4096
DS 4
SWH 22058.824 Hz
FIDRES 1.346364 Hz
AQ 0.3714207 sec
RG 2050
DW 22.667 usec
DE 6.50 usec
TE 300.0 K
D1 0.69999999 sec
D11 0.03000000 sec
TD0 1
======== CHANNEL f1 ========
NUC1 13C
P1 7.00 usec
PL1 -2.60 dB
PL1W 61.34123230 W
SFO1 100.6228298 MHz
======== CHANNEL f2 ========
CPDPRG2 waltz16
NUC2 1H
PCPD2 80.00 usec
PL2 -4.00 dB
PL12 14.06 dB
PL13 120.00 dB
PL2W 19.93825150 W
PL12W 0.31166428 W
PL13W 0.00000000 W
SFO2 400.1316005 MHz
SI 32768
SF 100.6128193 MHz
WDW EM
SSB 0
LB 1.00 Hz
GB 0
PC 1.40
OEt
N
H
O
ethyl 2-(phenylamino)acetate
190
180
170
160
150
140
130
120
110
100
90
80
70
60
50
40
30
20
10 ppm

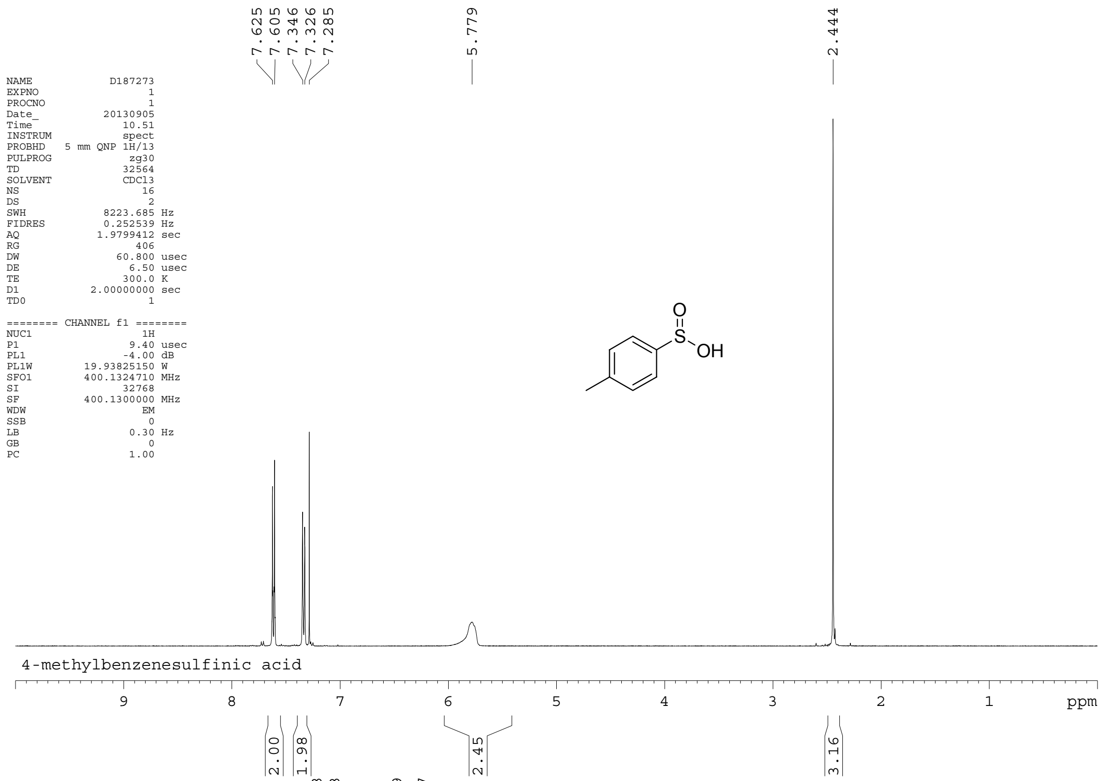
7.625
7.605
7.346
7.326
7.285
5.779
2.444
NAME D187273
EXPNO 1
PROCNO 1
Date_ 20130905
Time 10.51
INSTRUM spect
PROBHD 5 mm QNP 1H/13
PULPROG zg30
TD 32564
SOLVENT CDCl3
NS 16
DS 2
SWH 8223.685 Hz
FIDRES 0.252539 Hz
AQ 1.9799412 sec
RG 406
DW 60.800 usec
DE 6.50 usec
TE 300.0 K
D1 2.00000000 sec
TD0 1
======== CHANNEL f1 ========
NUC1 1H
P1 9.40 usec
PL1 -4.00 dB
PL1W 19.93825150 W
SFO1 400.1324710 MHz
SI 32768
SF 400.1300000 MHz
WDW EM
SSB 0
LB 0.30 Hz
GB 0
PC 1.00
O
S
OH
4-methylbenzenesulfinic acid
9 8 7 6 5 4 3 2 1 ppm
2.00
1.98
2.45
3.16

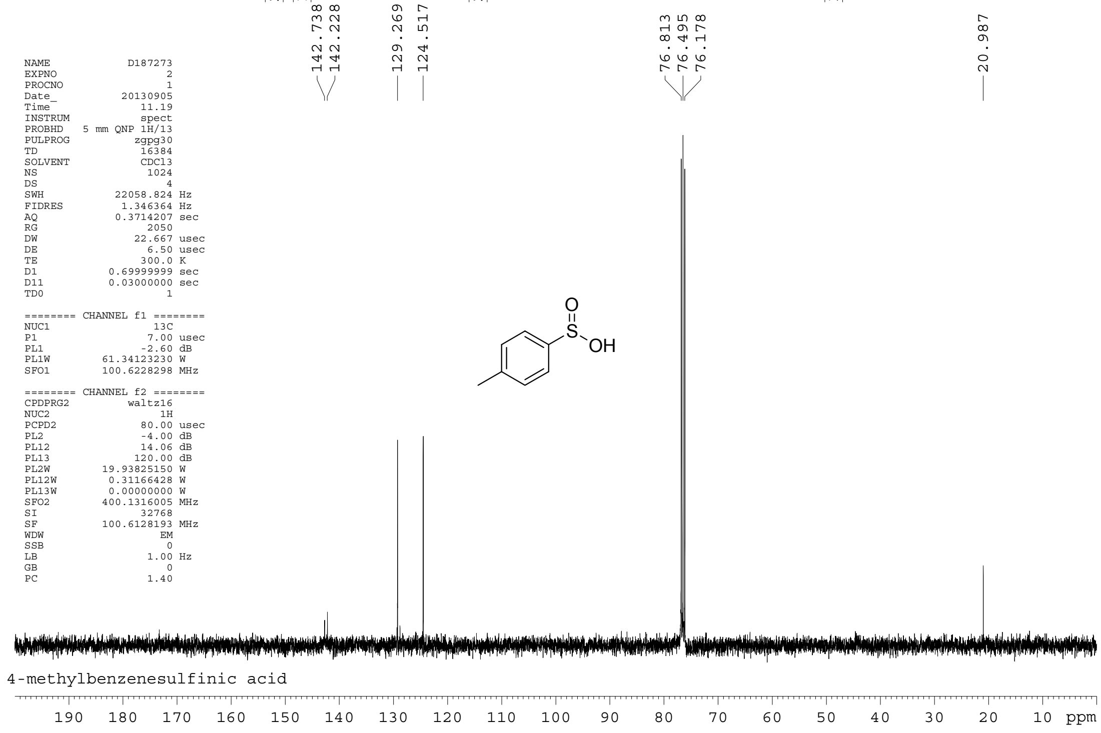
142.738
142.228
129.269
124.517
76.813
76.495
76.178
20.987
NAME D187273
EXPNO 2
PROCNO 1
Date_ 20130905
Time 11.19
INSTRUM spect
PROBHD 5 mm QNP 1H/13
PULPROG zgpg30
TD 16384
SOLVENT CDCl3
NS 1024
DS 4
SWH 22058.824 Hz
FIDRES 1.346364 Hz
AQ 0.3714207 sec
RG 2050
DW 22.667 usec
DE 6.50 usec
TE 300.0 K
D1 0.69999999 sec
D11 0.03000000 sec
TD0 1
======== CHANNEL f1 ========
NUC1 13C
P1 7.00 usec
PL1 -2.60 dB
PL1W 61.34123230 W
SFO1 100.6228298 MHz
======== CHANNEL f2 ========
CPDPRG2 waltz16
NUC2 1H
PCPD2 80.00 usec
PL2 -4.00 dB
PL12 14.06 dB
PL13 120.00 dB
PL2W 19.93825150 W
PL12W 0.31166428 W
PL13W 0.00000000 W
SFO2 400.1316005 MHz
SI 32768
SF 100.6128193 MHz
WDW EM
SSB 0
LB 1.00 Hz
GB 0
PC 1.40
O
S
OH
4-methylbenzenesulfinic acid
190 180 170 160 150 140 130 120 110 100 90 80 70 60 50 40 30 20 10 ppm

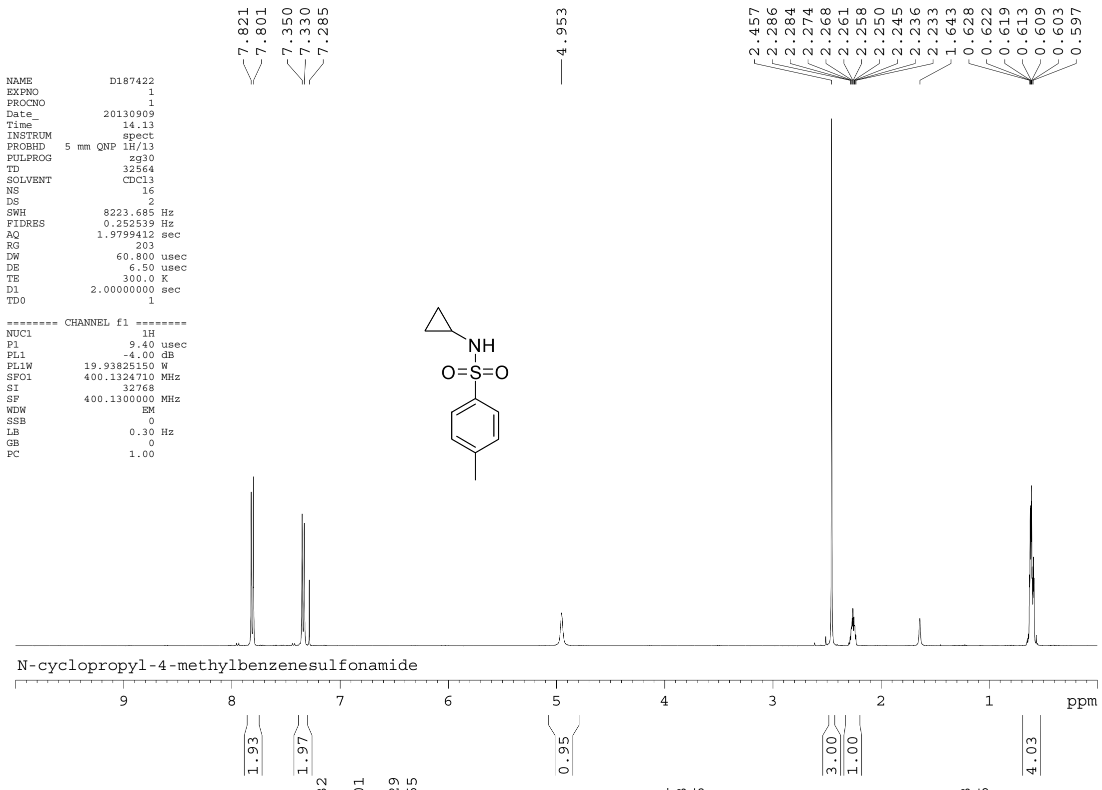
7.821
7.801
7.350
7.330
7.285
4.953
2.457
2.286
2.284
2.274
2.268
2.261
2.258
2.250
2.245
2.236
2.233
1.643
0.628
0.622
0.619
0.613
0.609
0.603
0.597
NAME D187422
EXPNO 1
PROCNO 1
Date_ 20130909
Time 14.13
INSTRUM spect
PROBHD 5 mm QNP 1H/13
PULPROG zg30
TD 32564
SOLVENT CDCl3
NS 16
DS 2
SWH 8223.685 Hz
FIDRES 0.252539 Hz
AQ 1.9799412 sec
RG 203
DW 60.800 usec
DE 6.50 usec
TE 300.0 K
D1 2.00000000 sec
TD0 1
======== CHANNEL f1 ========
NUC1 1H
P1 9.40 usec
PL1 -4.00 dB
PL1W 19.93825150 W
SFO1 400.1324710 MHz
SI 32768
SF 400.1300000 MHz
WDW EM
SSB 0
LB 0.30 Hz
GB 0
PC 1.00
NH
O=S=O
N-cyclopropyl-4-methylbenzenesulfonamide
9
8
7
6
5
4
3
2
1
ppm
1.93
1.97
0.95
3.00
1.00
4.03

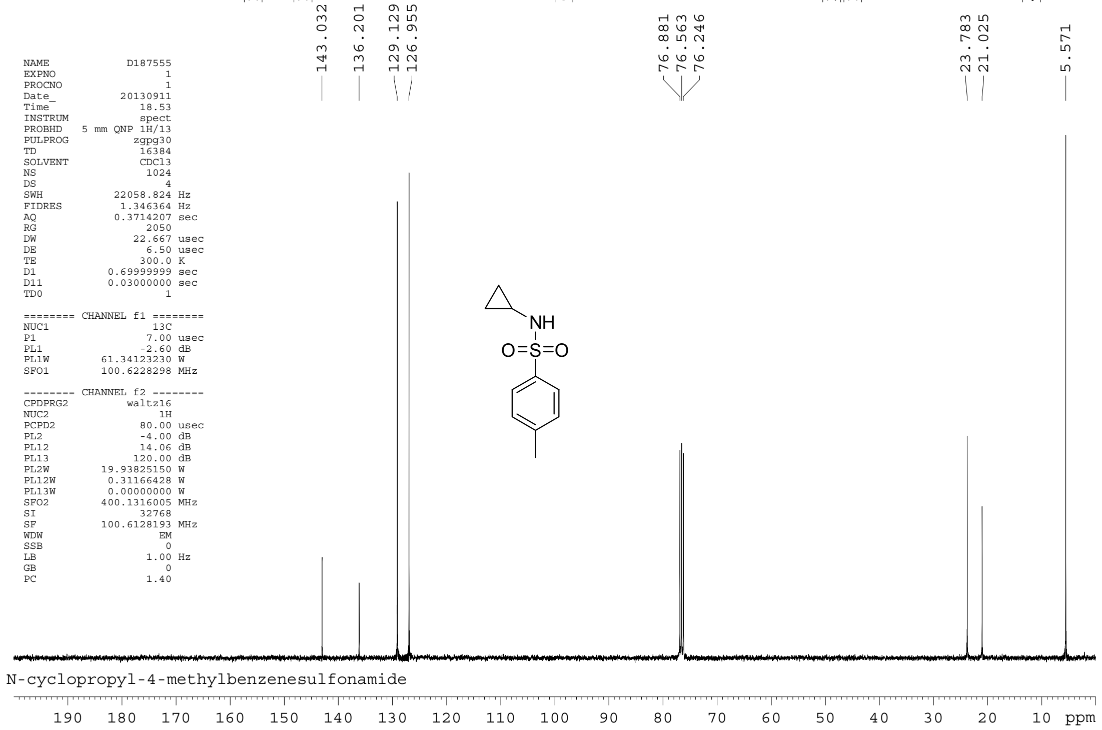
143.032
136.201
129.129
126.955
76.881
76.563
76.246
23.783
21.025
5.571
NAME D187555
EXPNO 1
PROCNO 1
Date_ 20130911
Time 18.53
INSTRUM spect
PROBHD 5 mm QNP 1H/13
PULPROG zgpg30
TD 16384
SOLVENT CDCl3
NS 1024
DS 4
SWH 22058.824 Hz
FIDRES 1.346364 Hz
AQ 0.3714207 sec
RG 2050
DW 22.667 usec
DE 6.50 usec
TE 300.0 K
D1 0.69999999 sec
D11 0.03000000 sec
TD0 1
======== CHANNEL f1 ========
NUC1 13C
P1 7.00 usec
PL1 -2.60 dB
PL1W 61.34123230 W
SFO1 100.6228298 MHz
======== CHANNEL f2 ========
CPDPRG2 waltz16
NUC2 1H
PCPD2 80.00 usec
PL2 -4.00 dB
PL12 14.06 dB
PL13 120.00 dB
PL2W 19.93825150 W
PL12W 0.31166428 W
PL13W 0.00000000 W
SFO2 400.1316005 MHz
SI 32768
SF 100.6128193 MHz
WDW EM
SSB 0
LB 1.00 Hz
GB 0
PC 1.40
NH
O=S=O
N-cyclopropyl-4-methylbenzenesulfonamide
190
180
170
160
150
140
130
120
110
100
90
80
70
60
50
40
30
20
10
ppm

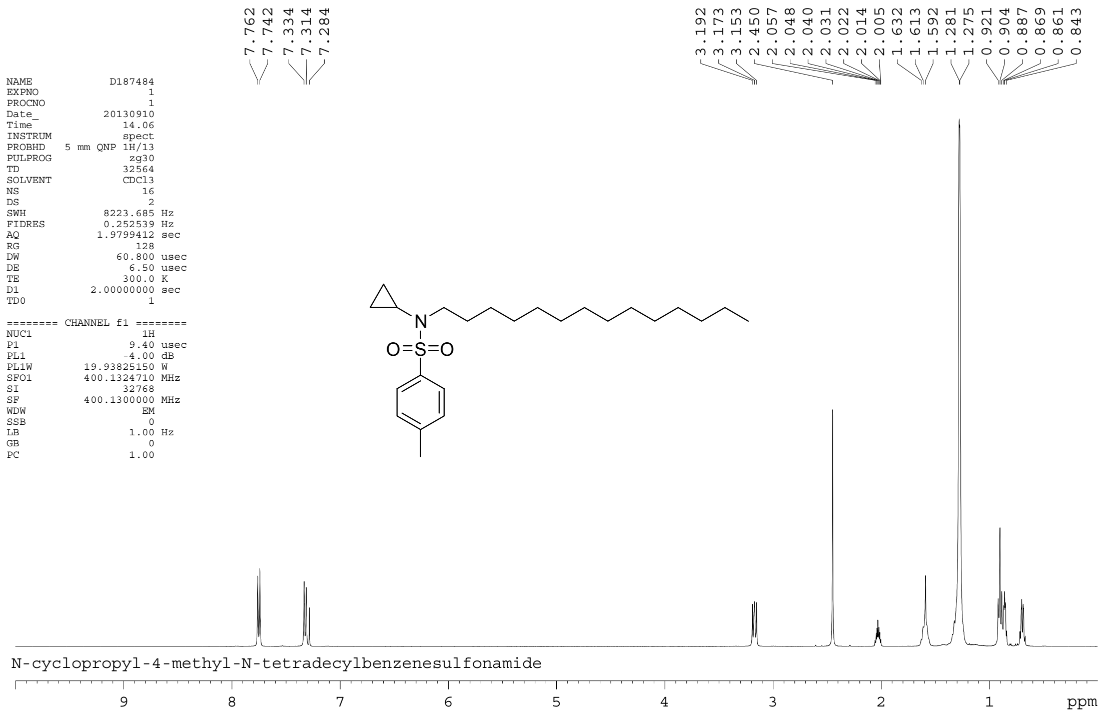
7.762
7.742
7.334
7.314
7.284
3.192
3.173
3.153
2.450
2.057
2.048
2.040
2.031
2.022
2.014
2.005
1.632
1.613
1.592
1.281
1.275
0.921
0.904
0.887
0.869
0.861
0.843
NAME D187484
EXPNO 1
PROCNO 1
Date_ 20130910
Time 14.06
INSTRUM spect
PROBHD 5 mm QNP 1H/13
PULPROG zg30
TD 32564
SOLVENT CDCl3
NS 16
DS 2
SWH 8223.685 Hz
FIDRES 0.252539 Hz
AQ 1.9799412 sec
RG 128
DW 60.800 usec
DE 6.50 usec
TE 300.0 K
D1 2.00000000 sec
TD0 1
======== CHANNEL f1 ========
NUC1 1H
P1 9.40 usec
PL1 -4.00 dB
PL1W 19.93825150 W
SFO1 400.1324710 MHz
SI 32768
SF 400.1300000 MHz
WDW EM
SSB 0
LB 1.00 Hz
GB 0
PC 1.00
N
O=S=O
N-cyclopropyl-4-methyl-N-tetradecylbenzenesulfonamide
9
8
7
6
5
4
3
2
1
ppm
3.95
3.99
4.06
6.04
2.03
5.58
43.58
10.67
4.11

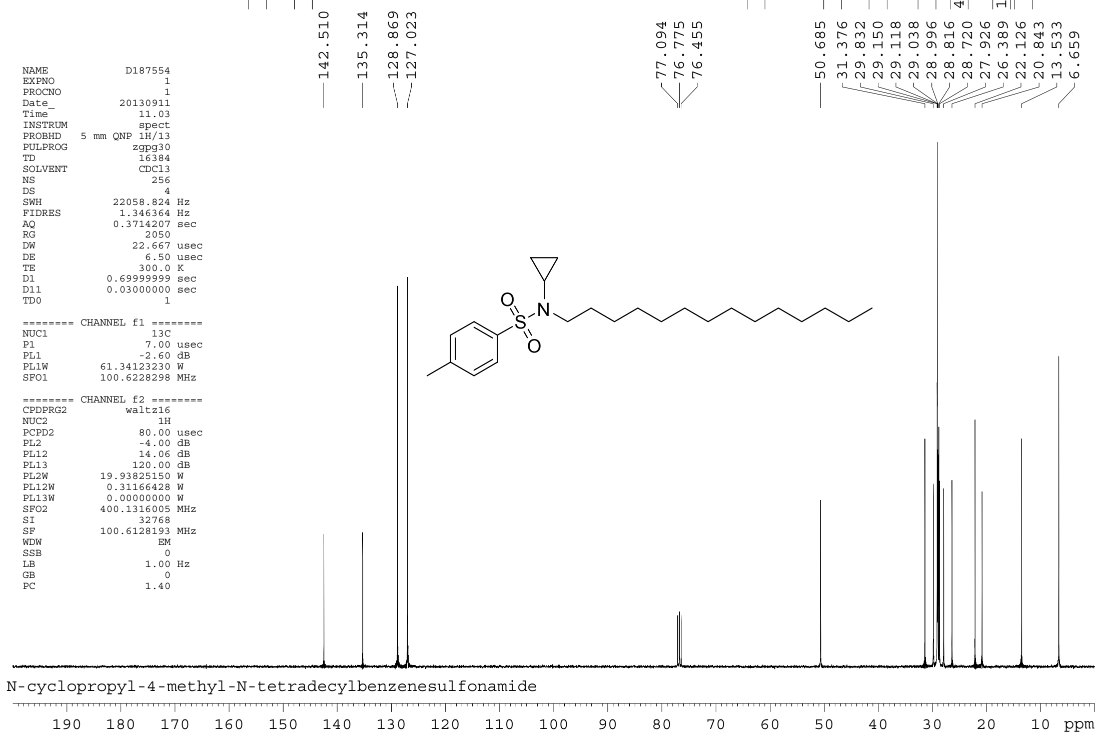
142.510
135.314
128.869
127.023
77.094
76.775
76.455
50.685
31.376
29.832
29.150
29.118
29.038
28.996
28.816
28.720
27.926
26.389
22.126
20.843
13.533
6.659
NAME D187554
EXPNO 1
PROCNO 1
Date_ 20130911
Time 11.03
INSTRUM spect
PROBHD 5 mm QNP 1H/13
PULPROG zgpg30
TD 16384
SOLVENT CDCl3
NS 256
DS 4
SWH 22058.824 Hz
FIDRES 1.346364 Hz
AQ 0.3714207 sec
RG 2050
DW 22.667 usec
DE 6.50 usec
TE 300.0 K
D1 0.69999999 sec
D11 0.03000000 sec
TD0 1
======== CHANNEL f1 ========
NUC1 13C
P1 7.00 usec
PL1 -2.60 dB
PL1W 61.34123230 W
SFO1 100.6228298 MHz
======== CHANNEL f2 ========
CPDPRG2 waltz16
NUC2 1H
PCPD2 80.00 usec
PL2 -4.00 dB
PL12 14.06 dB
PL13 120.00 dB
PL2W 19.93825150 W
PL12W 0.31166428 W
PL13W 0.00000000 W
SFO2 400.1316005 MHz
SI 32768
SF 100.6128193 MHz
WDW EM
SSB 0
LB 1.00 Hz
GB 0
PC 1.40
O
S
N
O
N-cyclopropyl-4-methyl-N-tetradecylbenzenesulfonamide
190
180
170
160
150
140
130
120
110
100
90
80
70
60
50
40
30
20
10
ppm

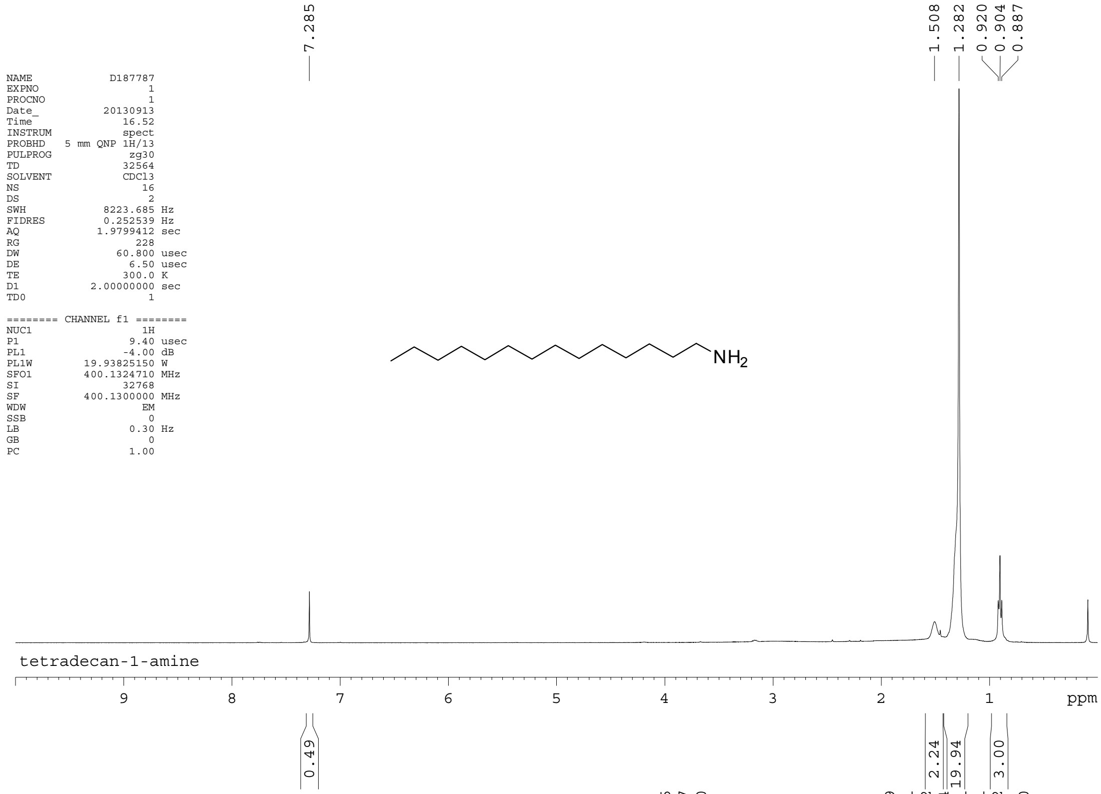
7.285
1.508
1.282
0.920
0.904
0.887
NAME D187787
EXPNO 1
PROCNO 1
Date_ 20130913
Time 16.52
INSTRUM spect
PROBHD 5 mm QNP 1H/13
PULPROG zg30
TD 32564
SOLVENT CDCl3
NS 16
DS 2
SWH 8223.685 Hz
FIDRES 0.252539 Hz
AQ 1.9799412 sec
RG 228
DW 60.800 usec
DE 6.50 usec
TE 300.0 K
D1 2.00000000 sec
TD0 1
======== CHANNEL f1 ========
NUC1 1H
P1 9.40 usec
PL1 -4.00 dB
PL1W 19.93825150 W
SFO1 400.1324710 MHz
SI 32768
SF 400.1300000 MHz
WDW EM
SSB 0
LB 0.30 Hz
GB 0
PC 1.00
NH2
tetradecan-1-amine
9
8
7
6
5
4
3
2
1
ppm
0.49
2.24
19.94
3.00

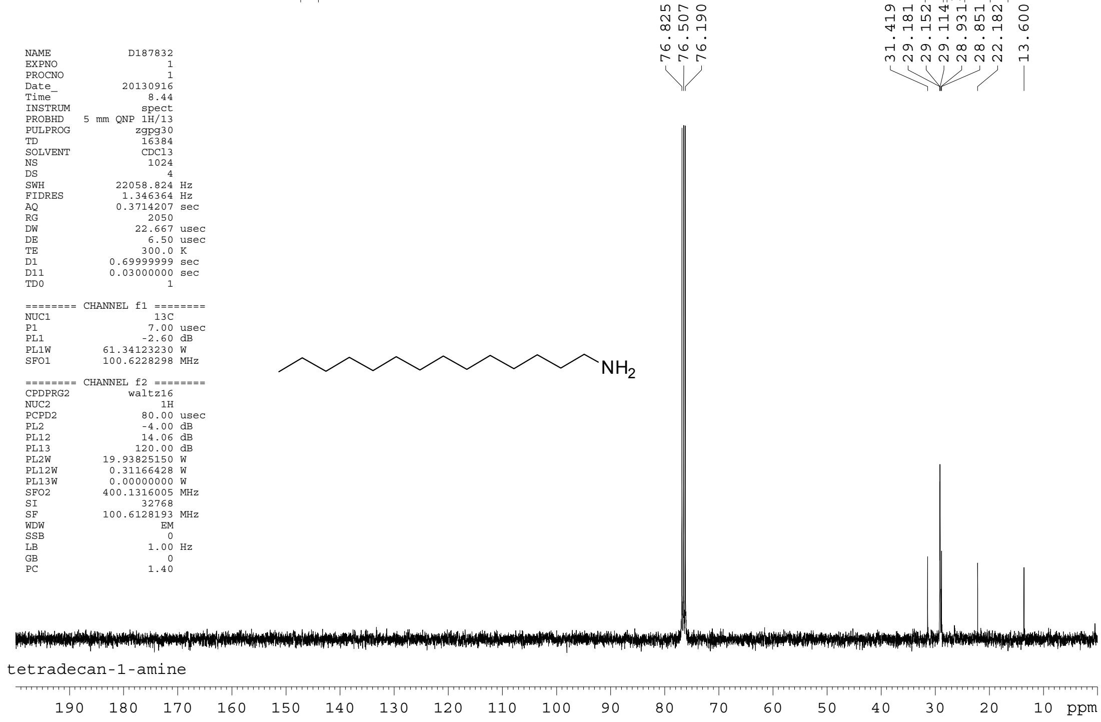
76.825
76.507
76.190
31.419
29.181
29.152
29.114
28.931
28.851
22.182
13.600
NAME D187832
EXPNO 1
PROCNO 1
Date_ 20130916
Time 8.44
INSTRUM spect
PROBHD 5 mm QNP 1H/13
PULPROG zgpg30
TD 16384
SOLVENT CDCl3
NS 1024
DS 4
SWH 22058.824 Hz
FIDRES 1.346364 Hz
AQ 0.3714207 sec
RG 2050
DW 22.667 usec
DE 6.50 usec
TE 300.0 K
D1 0.69999999 sec
D11 0.03000000 sec
TD0 1
======== CHANNEL f1 ========
NUC1 13C
P1 7.00 usec
PL1 -2.60 dB
PL1W 61.34123230 W
SFO1 100.6228298 MHz
======== CHANNEL f2 ========
CPDPRG2 waltz16
NUC2 1H
PCPD2 80.00 usec
PL2 -4.00 dB
PL12 14.06 dB
PL13 120.00 dB
PL2W 19.93825150 W
PL12W 0.31166428 W
PL13W 0.00000000 W
SFO2 400.1316005 MHz
SI 32768
SF 100.6128193 MHz
WDW EM
SSB 0
LB 1.00 Hz
GB 0
PC 1.40
NH2
tetradecan-1-amine
190
180
170
160
150
140
130
120
110
100
90
80
70
60
50
40
30
20
10
ppm

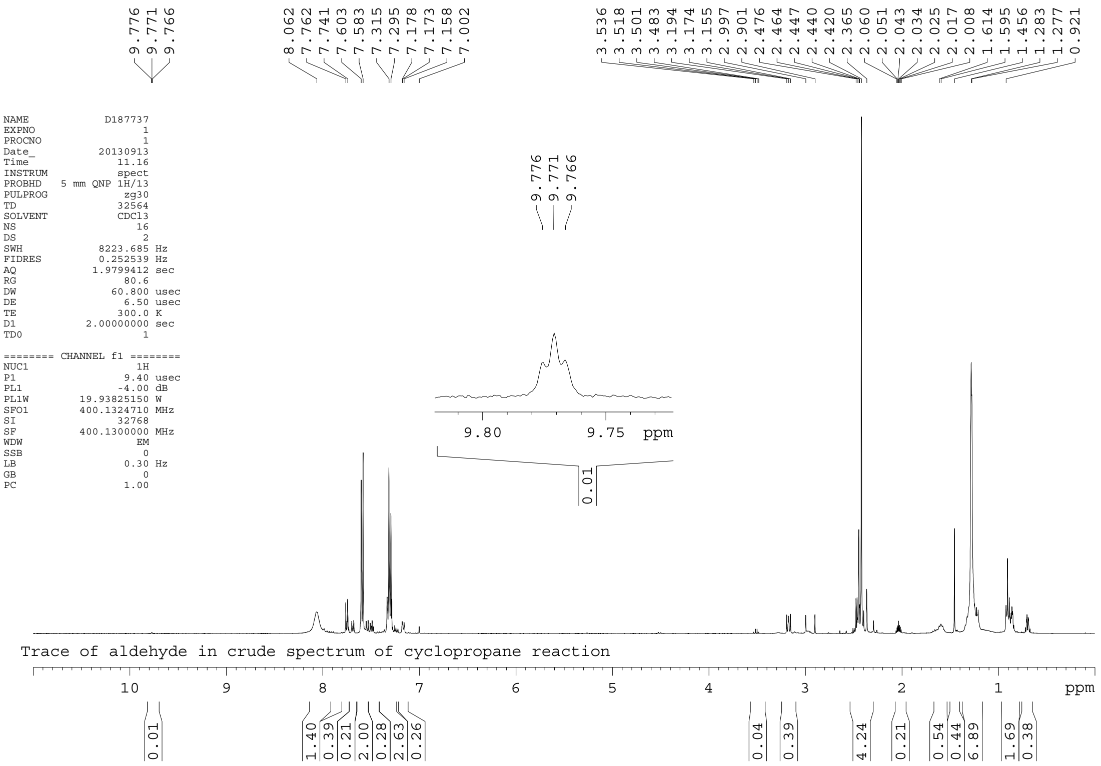

Crude ^{1}H NMR of reaction of *N*-cyclopropyl-4-methyl-*N*-tetradecylbenzenesulfonamide 28 with photoactivated donor 9

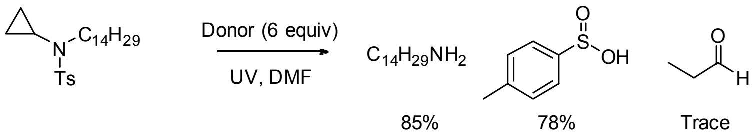

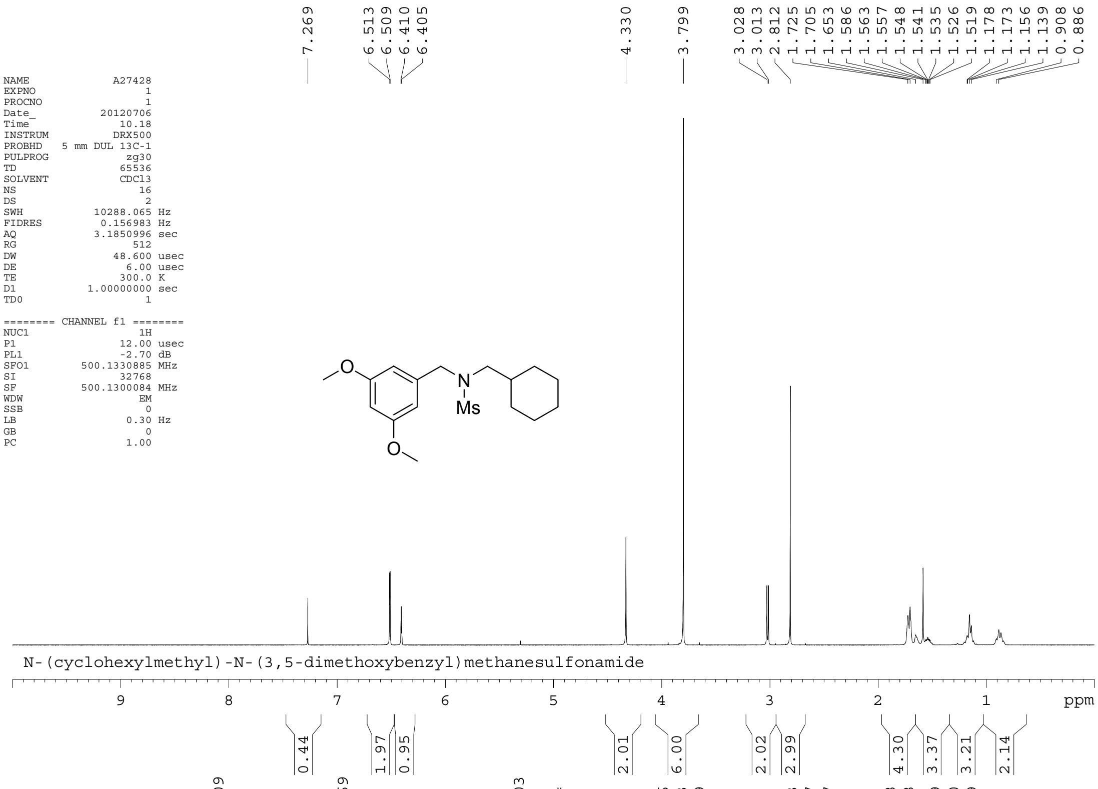
7.269
6.513
6.509
6.410
6.405
4.330
3.799
3.028
3.013
2.812
1.725
1.705
1.653
1.586
1.563
1.557
1.548
1.541
1.535
1.526
1.519
1.178
1.173
1.156
1.139
0.908
0.886
NAME A27428
EXPNO 1
PROCNO 1
Date_ 20120706
Time 10.18
INSTRUM DRX500
PROBHD 5 mm DUL 13C-1
PULPROG zg30
TD 65536
SOLVENT CDCl3
NS 16
DS 2
SWH 10288.065 Hz
FIDRES 0.156983 Hz
AQ 3.1850996 sec
RG 512
DW 48.600 usec
DE 6.00 usec
TE 300.0 K
D1 1.00000000 sec
TD0 1
======== CHANNEL f1 ========
NUC1 1H
P1 12.00 usec
PL1 -2.70 dB
SFO1 500.1330885 MHz
SI 32768
SF 500.1300084 MHz
WDW EM
SSB 0
LB 0.30 Hz
GB 0
PC 1.00
O
N
Ms
O
N-(cyclohexylmethyl)-N-(3,5-dimethoxybenzyl)methanesulfonamide
9
8
7
6
5
4
3
2
1
ppm
0.44
1.97
0.95
2.01
6.00
2.02
2.99
4.30
3.37
3.21
2.14

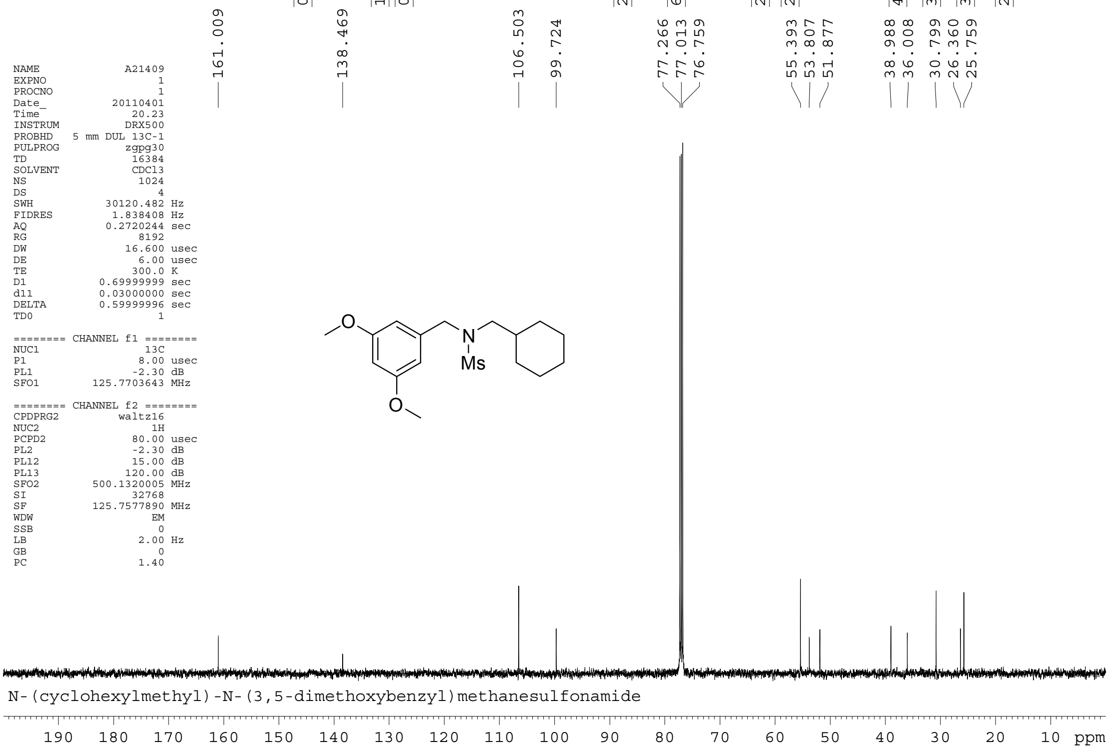
161.009
138.469
106.503
99.724
77.266
77.013
76.759
55.393
53.807
51.877
38.988
36.008
30.799
26.360
25.759
NAME A21409
EXPNO 1
PROCNO 1
Date_ 20110401
Time 20.23
INSTRUM DRX500
PROBHD 5 mm DUL 13C-1
PULPROG zgpg30
TD 16384
SOLVENT CDCl3
NS 1024
DS 4
SWH 30120.482 Hz
FIDRES 1.838408 Hz
AQ 0.2720244 sec
RG 8192
DW 16.600 usec
DE 6.00 usec
TE 300.0 K
D1 0.69999999 sec
d11 0.03000000 sec
DELTA 0.59999996 sec
TD0 1
======== CHANNEL f1 ========
NUC1 13C
P1 8.00 usec
PL1 -2.30 dB
SFO1 125.7703643 MHz
======== CHANNEL f2 ========
CPDPRG2 waltz16
NUC2 1H
PCPD2 80.00 usec
PL2 -2.30 dB
PL12 15.00 dB
PL13 120.00 dB
SFO2 500.1320005 MHz
SI 32768
SF 125.7577890 MHz
WDW EM
SSB 0
LB 2.00 Hz
GB 0
PC 1.40
O
N
Ms
O
N-(cyclohexylmethyl)-N-(3,5-dimethoxybenzyl)methanesulfonamide
190
180
170
160
150
140
130
120
110
100
90
80
70
60
50
40
30
20
10
ppm

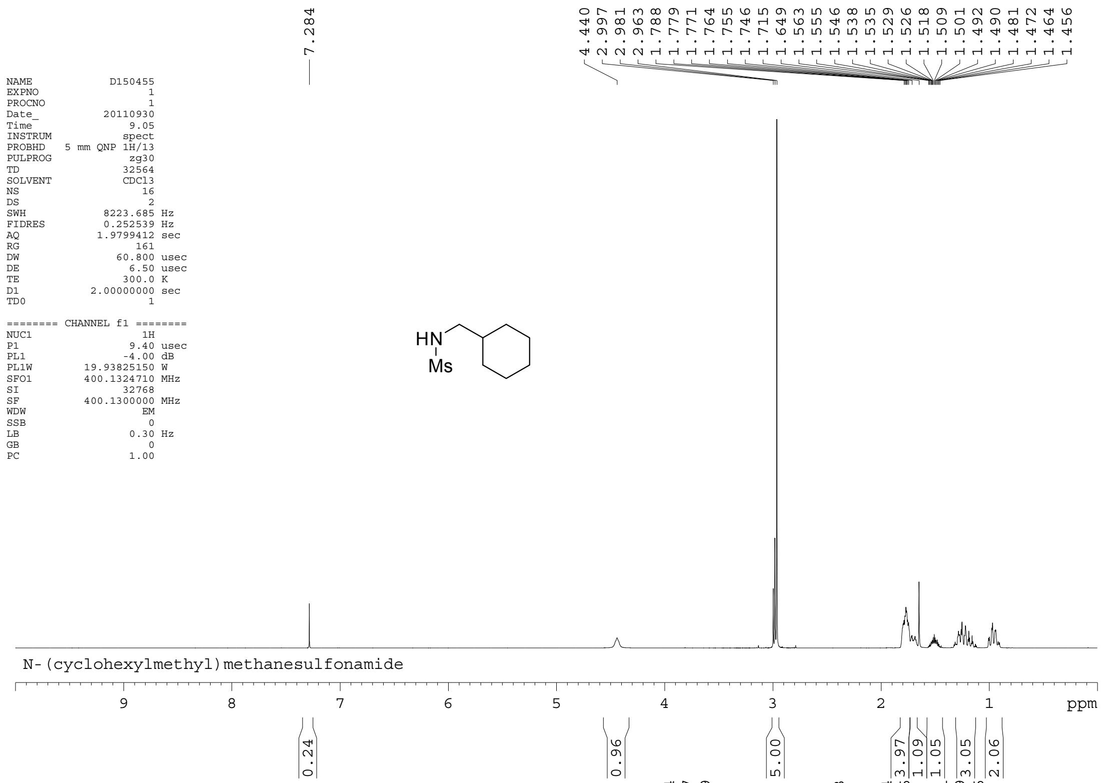
7.284
4.440 2.997 2.981 2.963 1.788 1.779 1.771 1.764 1.755 1.746 1.715 1.649 1.563 1.555 1.546 1.538 1.535 1.529 1.526 1.518 1.509 1.501 1.492 1.490 1.481 1.472 1.464 1.456
HN
Ms
N-(cyclohexylmethyl)methanesulfonamide
9 8 7 6 5 4 3 2 1 ppm
0.24 0.96 5.00 3.97 1.09 1.05 3.05 2.06

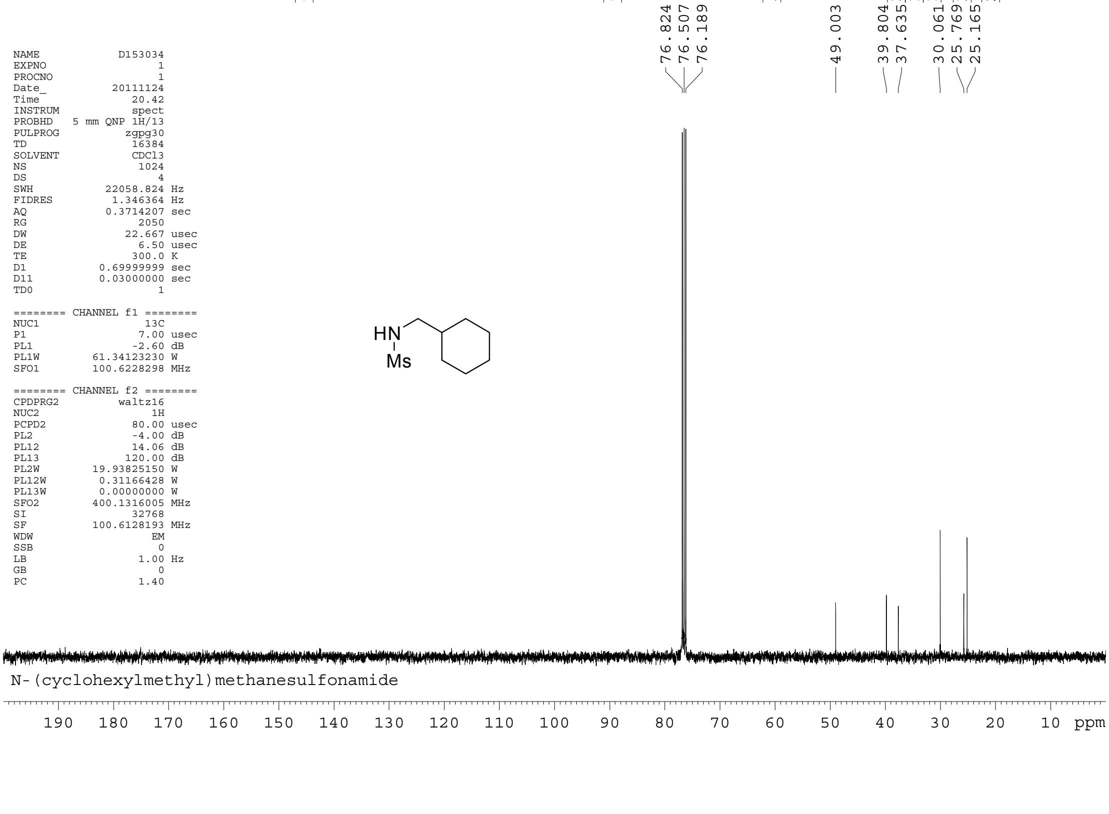
76.824 76.507 76.189 49.003 39.804 37.635 30.061 25.769 25.165
HN
Ms
N-(cyclohexylmethyl)methanesulfonamide
190 180 170 160 150 140 130 120 110 100 90 80 70 60 50 40 30 20 10 ppm

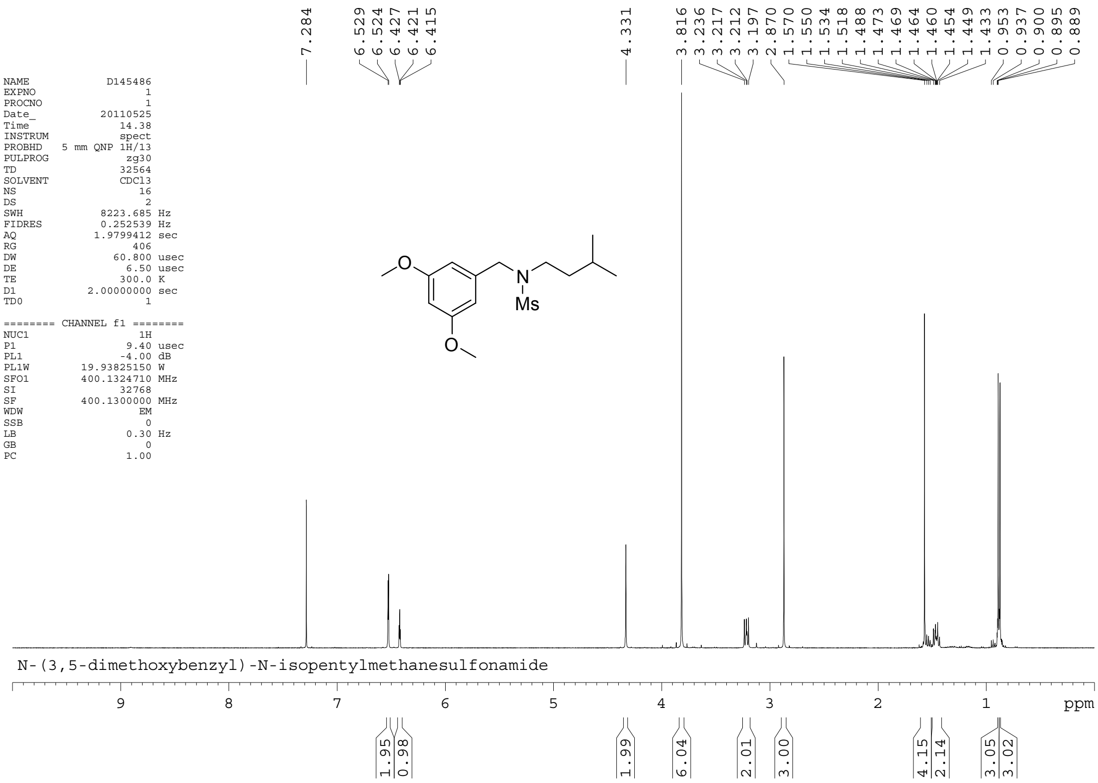
Ms
N-(3,5-dimethoxybenzyl)-N-isopentylmethanesulfonamide
ppm

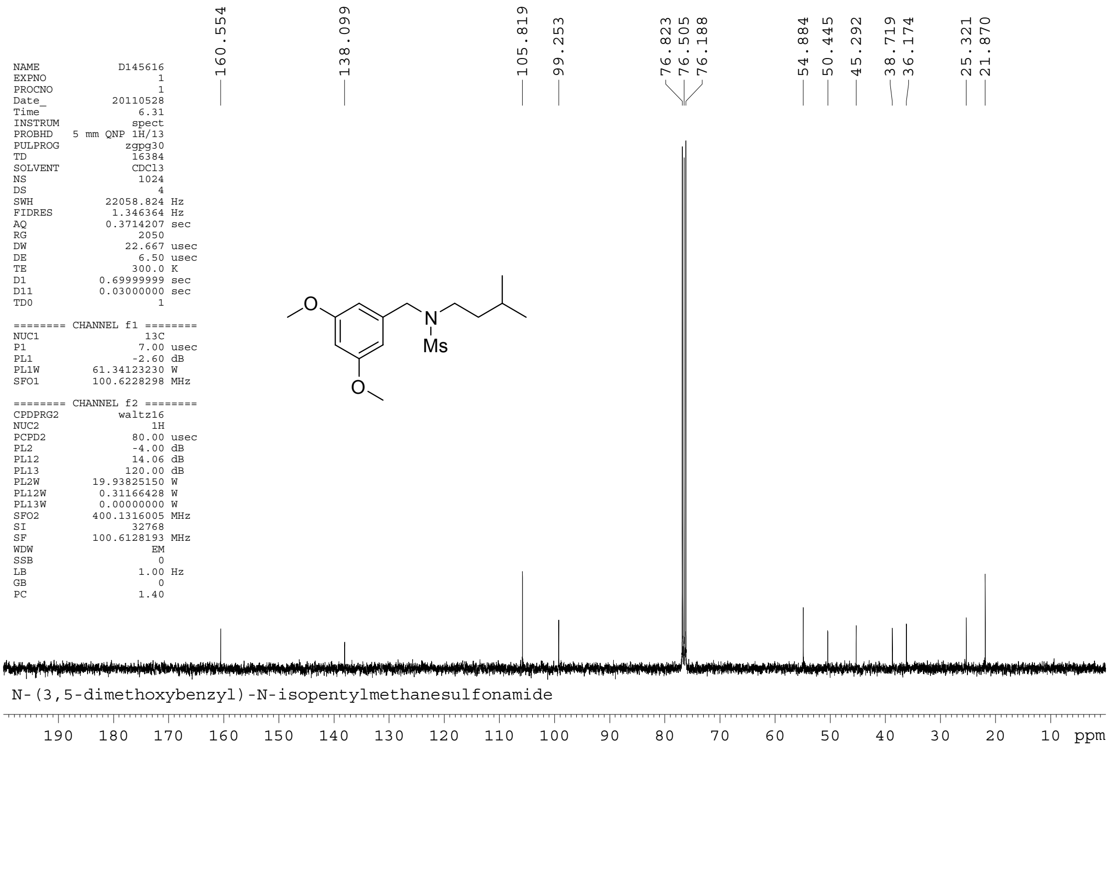
Ms
N-(3,5-dimethoxybenzyl)-N-isopentylmethanesulfonamide
ppm

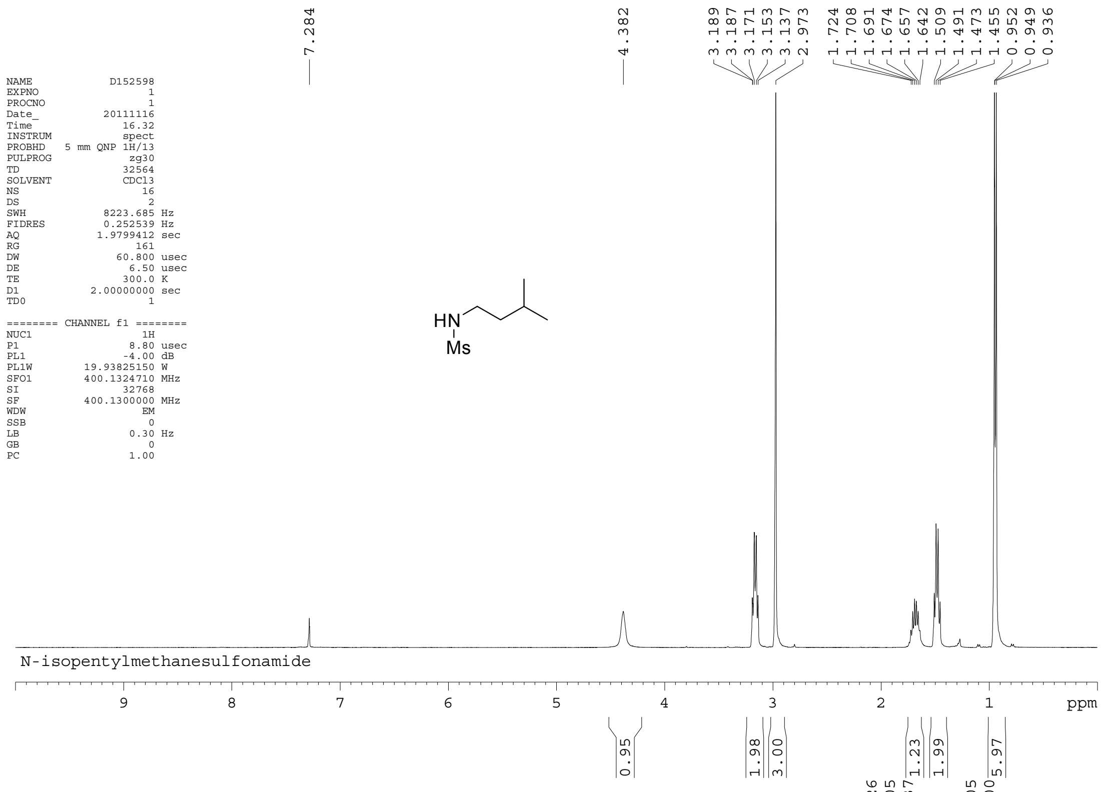
7.284
4.382
3.189 3.187 3.171 3.153 3.137 2.973
1.724 1.708 1.691 1.674 1.657 1.642 1.509 1.491 1.473 1.455 0.952 0.949 0.936
NAME D152598
EXPNO 1
PROCNO 1
Date_ 20111116
Time 16.32
INSTRUM spect
PROBHD 5 mm QNP 1H/13
PULPROG zg30
TD 32564
SOLVENT CDCl3
NS 16
DS 2
SWH 8223.685 Hz
FIDRES 0.252539 Hz
AQ 1.9799412 sec
RG 161
DW 60.800 usec
DE 6.50 usec
TE 300.0 K
D1 2.00000000 sec
TD0 1
======== CHANNEL f1 ========
NUC1 1H
P1 8.80 usec
PL1 -4.00 dB
PL1W 19.93825150 W
SFO1 400.1324710 MHz
SI 32768
SF 400.1300000 MHz
WDW EM
SSB 0
LB 0.30 Hz
GB 0
PC 1.00
HN
Ms
N-isopentylmethanesulfonamide
9 8 7 6 5 4 3 2 1 ppm
0.95 1.98 3.00 1.23 1.99 5.97

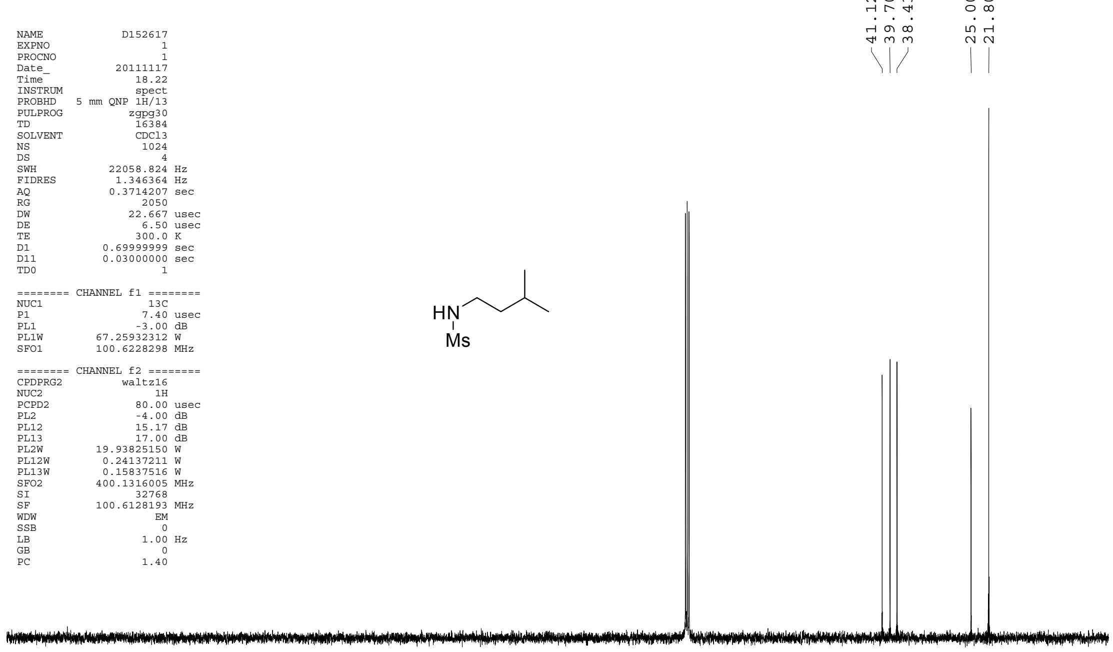
41.126 39.705 38.437 25.005 21.800
NAME D152617
EXPNO 1
PROCNO 1
Date_ 20111117
Time 18.22
INSTRUM spect
PROBHD 5 mm QNP 1H/13
PULPROG zgpg30
TD 16384
SOLVENT CDCl3
NS 1024
DS 4
SWH 22058.824 Hz
FIDRES 1.346364 Hz
AQ 0.3714207 sec
RG 2050
DW 22.667 usec
DE 6.50 usec
TE 300.0 K
D1 0.69999999 sec
D11 0.03000000 sec
TD0 1
======== CHANNEL f1 ========
NUC1 13C
P1 7.40 usec
PL1 -3.00 dB
PL1W 67.25932312 W
SFO1 100.6228298 MHz
======== CHANNEL f2 ========
CPDPRG2 waltz16
NUC2 1H
PCPD2 80.00 usec
PL2 -4.00 dB
PL12 15.17 dB
PL13 17.00 dB
PL2W 19.93825150 W
PL12W 0.24137211 W
PL13W 0.15837516 W
SFO2 400.1316005 MHz
SI 32768
SF 100.6128193 MHz
WDW EM
SSB 0
LB 1.00 Hz
GB 0
PC 1.40
HN
Ms
N-isopentylmethanesulfonamide
190 180 170 160 150 140 130 120 110 100 90 80 70 60 50 40 30 20 10 ppm

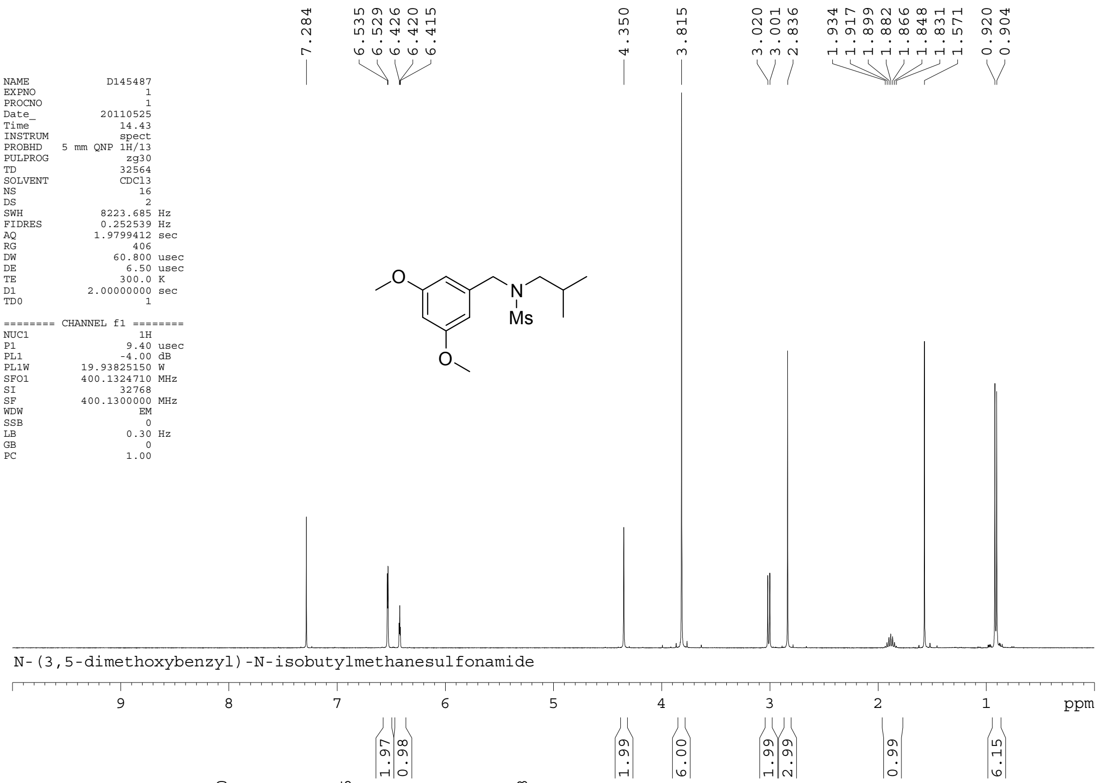
NAME D145487
EXPNO 1
PROCNO 1
Date_ 20110525
Time 14.43
INSTRUM spect
PROBHD 5 mm QNP 1H/13
PULPROG zg30
TD 32564
SOLVENT CDCl3
NS 16
DS 2
SWH 8223.685 Hz
FIDRES 0.252539 Hz
AQ 1.9799412 sec
RG 406
DW 60.800 usec
DE 6.50 usec
TE 300.0 K
D1 2.00000000 sec
TD0 1
======== CHANNEL f1 ========
NUC1 1H
P1 9.40 usec
PL1 -4.00 dB
PL1W 19.93825150 W
SFO1 400.1324710 MHz
SI 32768
SF 400.1300000 MHz
WDW EM
SSB 0
LB 0.30 Hz
GB 0
PC 1.00
7.284
6.535 6.529 6.426 6.420 6.415
4.350
3.815
3.020 3.001 2.836
1.934 1.917 1.899 1.882 1.866 1.848 1.831 1.571
0.920 0.904
O
N
Ms
O
N-(3,5-dimethoxybenzyl)-N-isobutylmethanesulfonamide
9 8 7 6 5 4 3 2 1 ppm
1.97 0.98 1.99 6.00 1.99 2.99 0.99 6.15

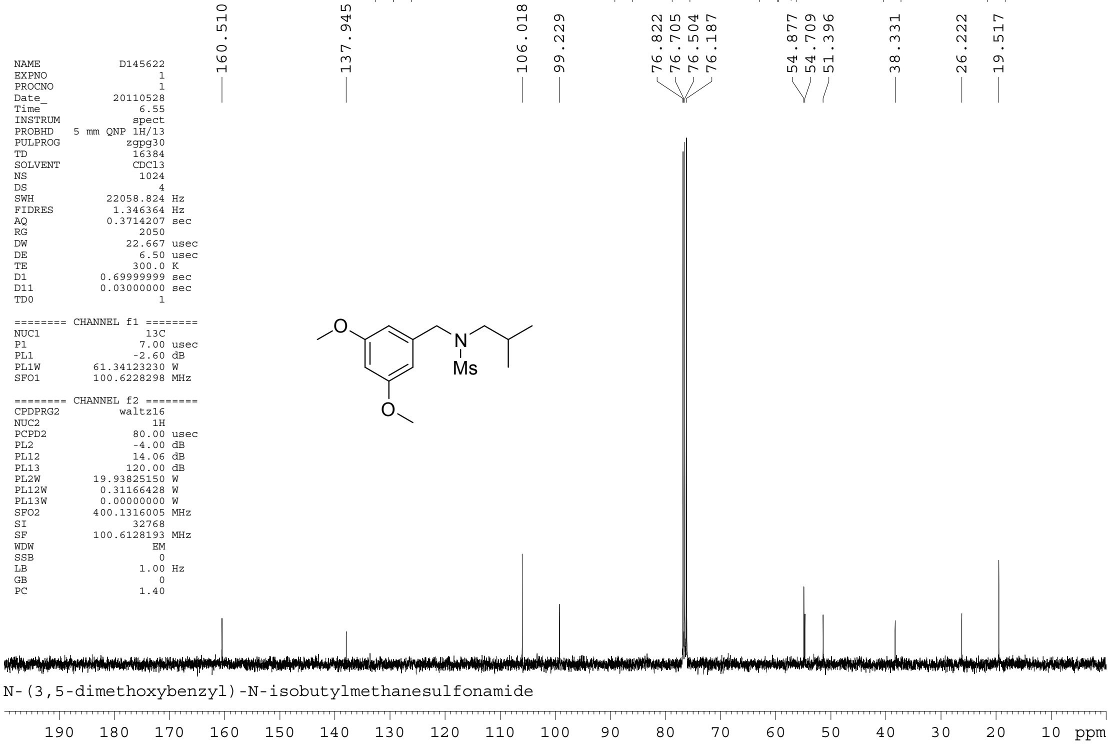
NAME D145622
EXPNO 1
PROCNO 1
Date_ 20110528
Time 6.55
INSTRUM spect
PROBHD 5 mm QNP 1H/13
PULPROG zgpg30
TD 16384
SOLVENT CDCl3
NS 1024
DS 4
SWH 22058.824 Hz
FIDRES 1.346364 Hz
AQ 0.3714207 sec
RG 2050
DW 22.667 usec
DE 6.50 usec
TE 300.0 K
D1 0.69999999 sec
D11 0.03000000 sec
TD0 1
======== CHANNEL f1 ========
NUC1 13C
P1 7.00 usec
PL1 -2.60 dB
PL1W 61.34123230 W
SFO1 100.6228298 MHz
======== CHANNEL f2 ========
CPDPRG2 waltz16
NUC2 1H
PCPD2 80.00 usec
PL2 -4.00 dB
PL12 14.06 dB
PL13 120.00 dB
PL2W 19.93825150 W
PL12W 0.31166428 W
PL13W 0.00000000 W
SFO2 400.1316005 MHz
SI 32768
SF 100.6128193 MHz
WDW EM
SSB 0
LB 1.00 Hz
GB 0
PC 1.40
160.510
137.945
106.018
99.229
76.822 76.705 76.504 76.187
54.877 54.709 51.396
38.331
26.222
19.517
O
N
Ms
O
N-(3,5-dimethoxybenzyl)-N-isobutylmethanesulfonamide
190 180 170 160 150 140 130 120 110 100 90 80 70 60 50 40 30 20 10 ppm

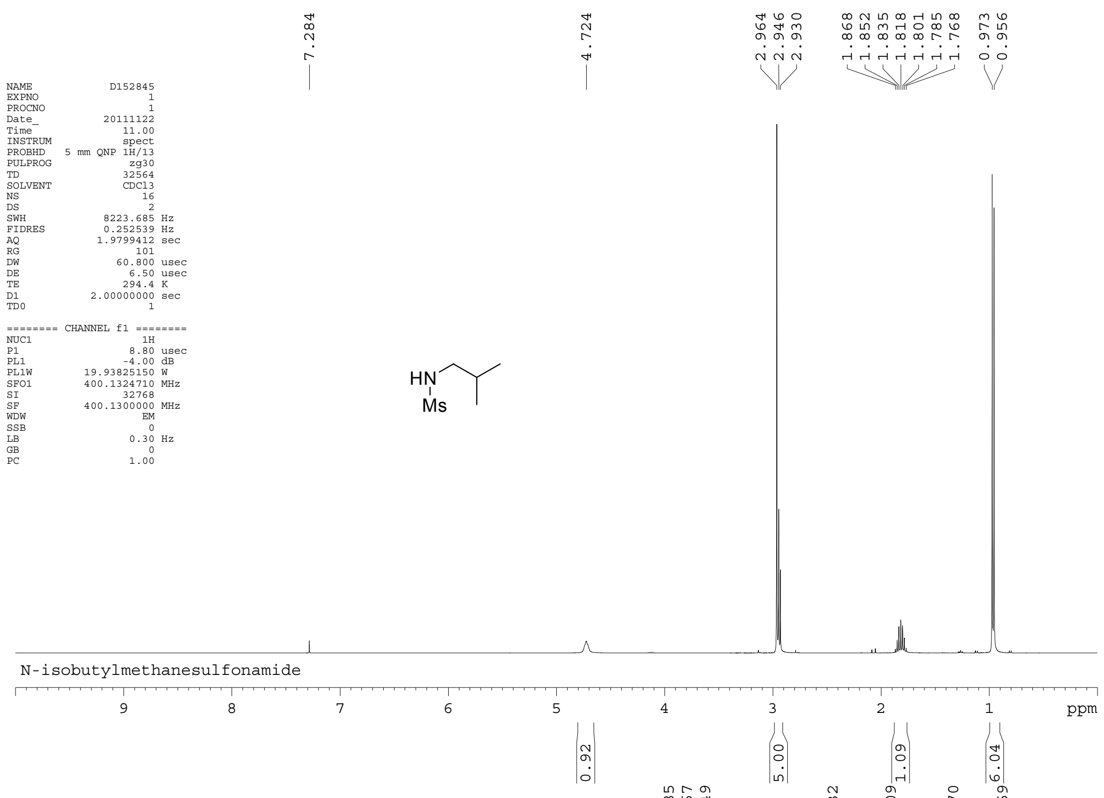
7.284
4.724
2.964
2.946
2.930
1.868
1.852
1.835
1.818
1.801
1.785
1.768
0.973
0.956
NAME D152845
EXPNO 1
PROCNO 1
Date_ 20111122
Time 11.00
INSTRUM spect
PROBHD 5 mm QNP 1H/13
PULPROG zg30
TD 32564
SOLVENT CDCl3
NS 16
DS 2
SWH 8223.685 Hz
FIDRES 0.252539 Hz
AQ 1.9799412 sec
RG 101
DW 60.800 usec
DE 6.50 usec
TE 294.4 K
D1 2.00000000 sec
TD0 1
======== CHANNEL f1 ========
NUC1 1H
P1 8.80 usec
PL1 -4.00 dB
PL1W 19.93825150 W
SFO1 400.1324710 MHz
SI 32768
SF 400.1300000 MHz
WDW EM
SSB 0
LB 0.30 Hz
GB 0
PC 1.00
HN
Ms
N-isobutylmethanesulfonamide
9 8 7 6 5 4 3 2 1 ppm
0.92
5.00
1.09
6.04

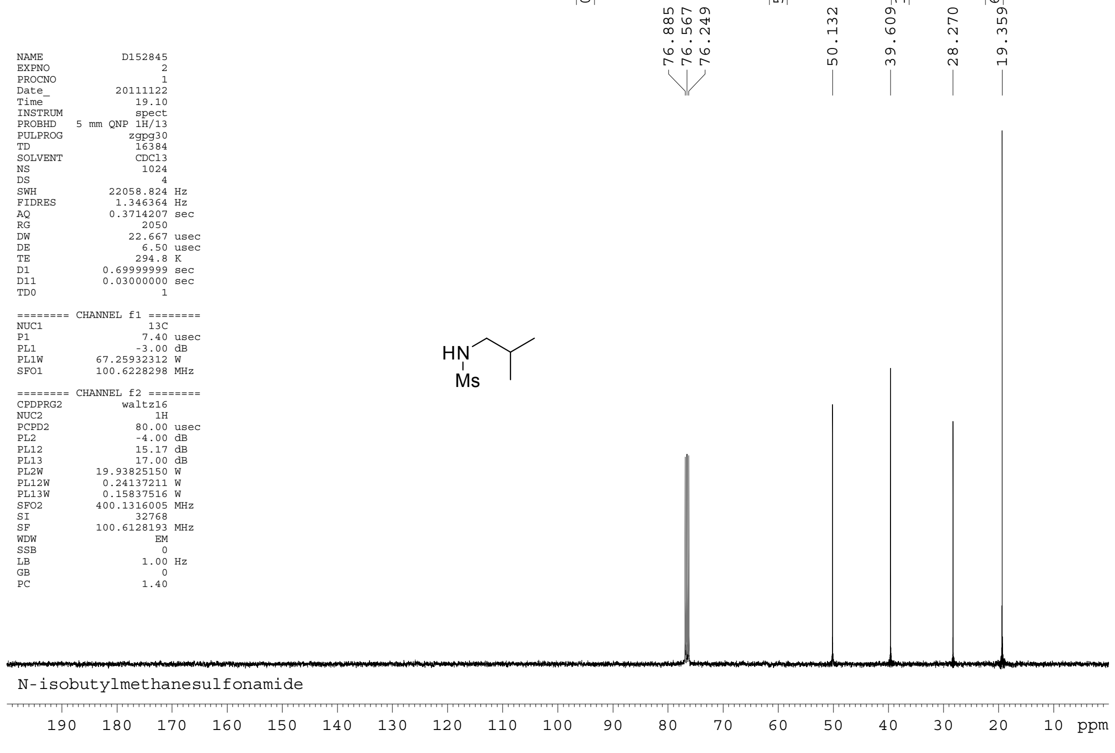
76.885
76.567
76.249
50.132
39.609
28.270
19.359
NAME D152845
EXPNO 2
PROCNO 1
Date_ 20111122
Time 19.10
INSTRUM spect
PROBHD 5 mm QNP 1H/13
PULPROG zgpg30
TD 16384
SOLVENT CDCl3
NS 1024
DS 4
SWH 22058.824 Hz
FIDRES 1.346364 Hz
AQ 0.3714207 sec
RG 2050
DW 22.667 usec
DE 6.50 usec
TE 294.8 K
D1 0.69999999 sec
D11 0.03000000 sec
TD0 1
======== CHANNEL f1 ========
NUC1 13C
P1 7.40 usec
PL1 -3.00 dB
PL1W 67.25932312 W
SFO1 100.6228298 MHz
======== CHANNEL f2 ========
CPDPRG2 waltz16
NUC2 1H
PCPD2 80.00 usec
PL2 -4.00 dB
PL12 15.17 dB
PL13 17.00 dB
PL2W 19.93825150 W
PL12W 0.24137211 W
PL13W 0.15837516 W
SFO2 400.1316005 MHz
SI 32768
SF 100.6128193 MHz
WDW EM
SSB 0
LB 1.00 Hz
GB 0
PC 1.40
HN
Ms
N-isobutylmethanesulfonamide
190 180 170 160 150 140 130 120 110 100 90 80 70 60 50 40 30 20 10 ppm

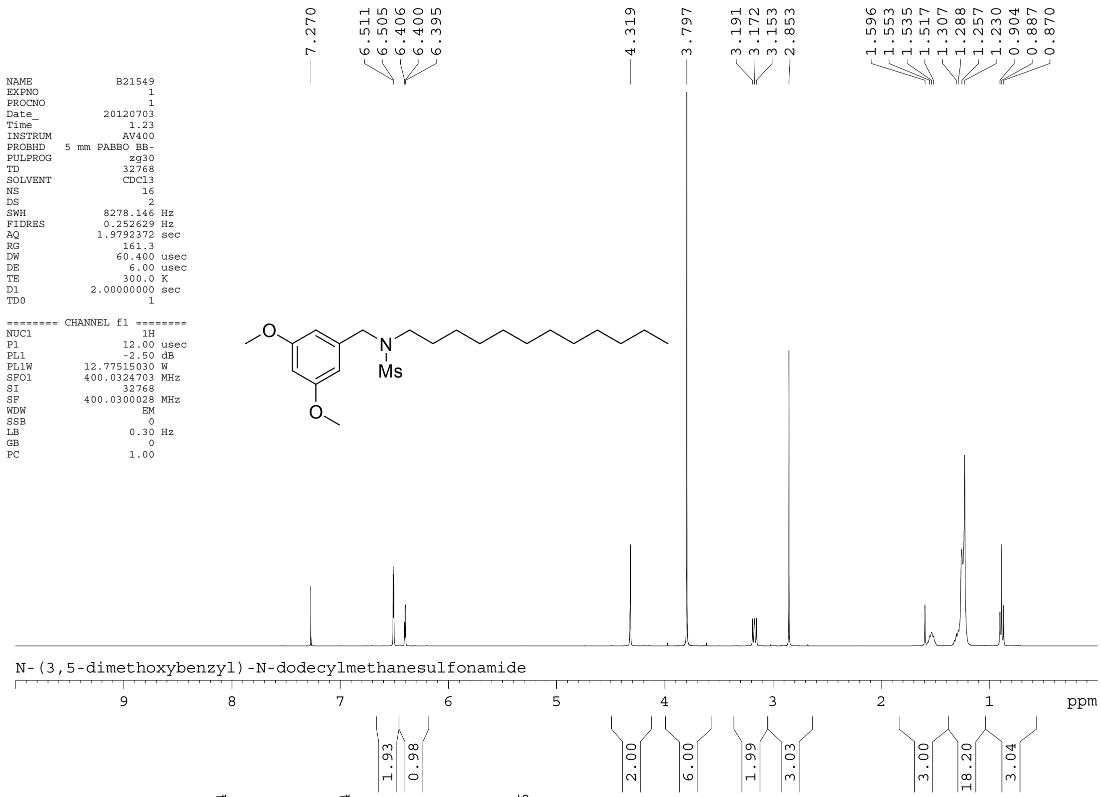
NAME B21549
EXPNO 1
PROCNO 1
Date_ 20120703
Time 1.23
INSTRUM AV400
PROBHD 5 mm PABBO BB-
PULPROG zg30
TD 32768
SOLVENT CDCl3
NS 16
DS 2
SWH 8278.146 Hz
FIDRES 0.252629 Hz
AQ 1.9792372 sec
RG 161.3
DW 60.400 usec
DE 6.00 usec
TE 300.0 K
D1 2.00000000 sec
TD0 1
======== CHANNEL f1 ========
NUC1 1H
P1 12.00 usec
PL1 -2.50 dB
PL1W 12.77515030 W
SFO1 400.0324703 MHz
SI 32768
SF 400.0300028 MHz
WDW EM
SSB 0
LB 0.30 Hz
GB 0
PC 1.00
7.270
6.511
6.505
6.406
6.400
6.395
4.319
3.797
3.191
3.172
3.153
2.853
1.596
1.553
1.535
1.517
1.307
1.288
1.257
1.230
0.904
0.887
0.870
O
N
Ms
O
N-(3,5-dimethoxybenzyl)-N-dodecylmethanesulfonamide
9 8 7 6 5 4 3 2 1 ppm
1.93
0.98
2.00
6.00
1.99
3.03
3.00
18.20
3.04

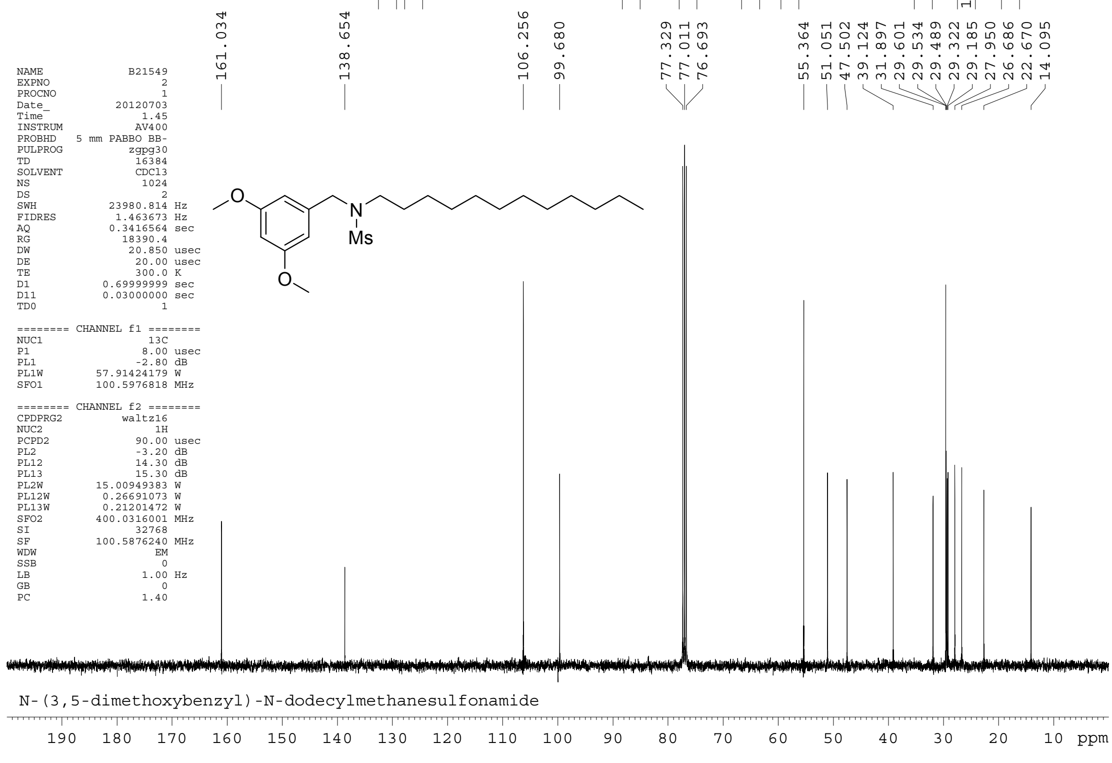
NAME B21549
EXPNO 2
PROCNO 1
Date_ 20120703
Time 1.45
INSTRUM AV400
PROBHD 5 mm PABBO BB-
PULPROG zgpg30
TD 16384
SOLVENT CDCl3
NS 1024
DS 2
SWH 23980.814 Hz
FIDRES 1.463673 Hz
AQ 0.3416564 sec
RG 18390.4
DW 20.850 usec
DE 20.00 usec
TE 300.0 K
D1 0.69999999 sec
D11 0.03000000 sec
TD0 1
======== CHANNEL f1 ========
NUC1 13C
P1 8.00 usec
PL1 -2.80 dB
PL1W 57.91424179 W
SFO1 100.5976818 MHz
======== CHANNEL f2 ========
CPDPRG2 waltz16
NUC2 1H
PCPD2 90.00 usec
PL2 -3.20 dB
PL12 14.30 dB
PL13 15.30 dB
PL2W 15.00949383 W
PL12W 0.26691073 W
PL13W 0.21201472 W
SFO2 400.0316001 MHz
SI 32768
SF 100.5876240 MHz
WDW EM
SSB 0
LB 1.00 Hz
GB 0
PC 1.40
161.034
138.654
106.256
99.680
77.329
77.011
76.693
55.364
51.051
47.502
39.124
31.897
29.601
29.534
29.489
29.322
29.185
27.950
26.686
22.670
14.095
O
N
Ms
O
N-(3,5-dimethoxybenzyl)-N-dodecylmethanesulfonamide
190 180 170 160 150 140 130 120 110 100 90 80 70 60 50 40 30 20 10 ppm

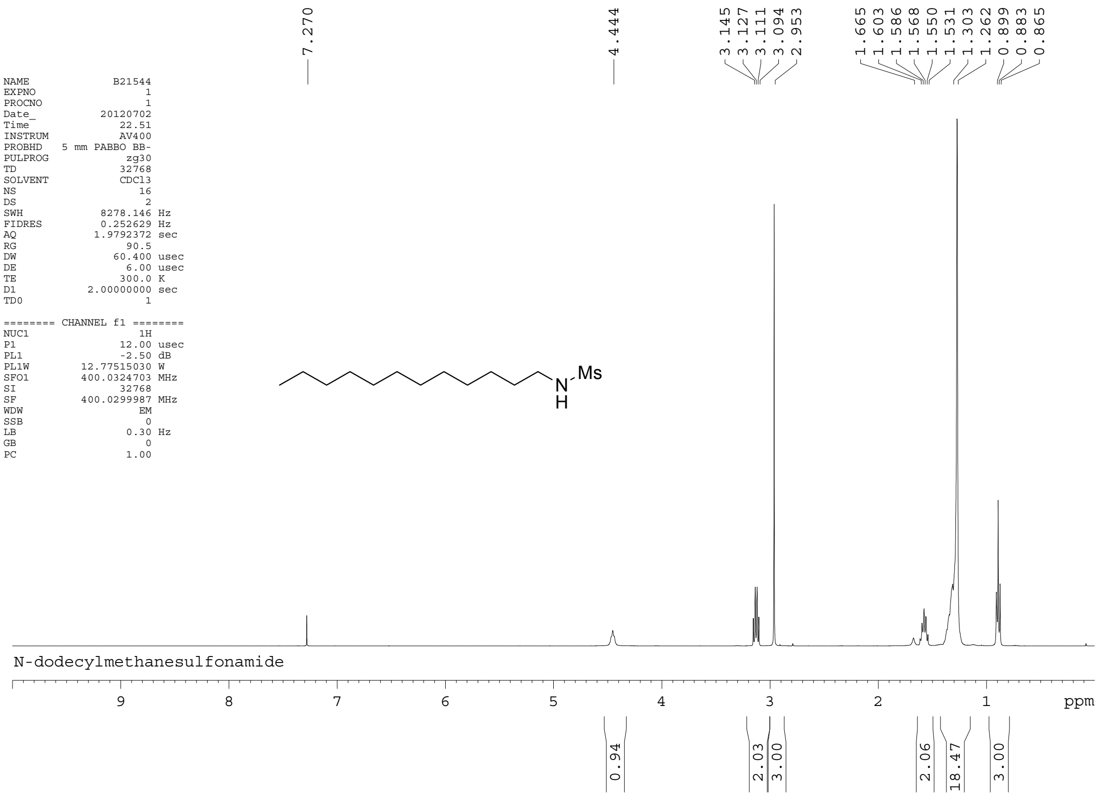
NAME B21544
EXPNO 1
PROCNO 1
Date_ 20120702
Time 22.51
INSTRUM AV400
PROBHD 5 mm PABBO BB-
PULPROG zg30
TD 32768
SOLVENT CDCl3
NS 16
DS 2
SWH 8278.146 Hz
FIDRES 0.252629 Hz
AQ 1.9792372 sec
RG 90.5
DW 60.400 usec
DE 6.00 usec
TE 300.0 K
D1 2.00000000 sec
TD0 1
======== CHANNEL f1 ========
NUC1 1H
P1 12.00 usec
PL1 -2.50 dB
PL1W 12.77515030 W
SFO1 400.0324703 MHz
SI 32768
SF 400.0299987 MHz
WDW EM
SSB 0
LB 0.30 Hz
GB 0
PC 1.00
7.270
4.444
3.145
3.127
3.111
3.094
2.953
1.665
1.603
1.586
1.568
1.550
1.531
1.303
1.262
0.899
0.883
0.865
N
H
Ms
N-dodecylmethanesulfonamide
9 8 7 6 5 4 3 2 1 ppm
0.94
2.03
3.00
2.06
18.47
3.00

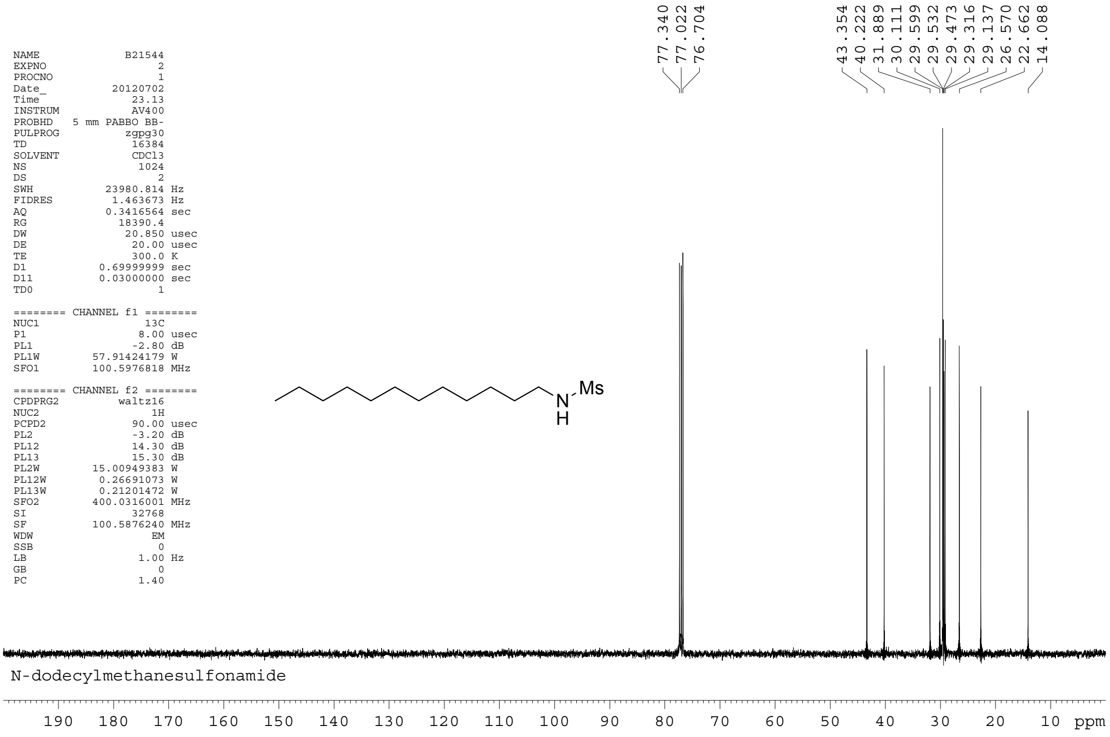
NAME B21544
EXPNO 2
PROCNO 1
Date_ 20120702
Time 23.13
INSTRUM AV400
PROBHD 5 mm PABBO BB-
PULPROG zgpg30
TD 16384
SOLVENT CDCl3
NS 1024
DS 2
SWH 23980.814 Hz
FIDRES 1.463673 Hz
AQ 0.3416564 sec
RG 18390.4
DW 20.850 usec
DE 20.00 usec
TE 300.0 K
D1 0.69999999 sec
D11 0.03000000 sec
TD0 1
======== CHANNEL f1 ========
NUC1 13C
P1 8.00 usec
PL1 -2.80 dB
PL1W 57.91424179 W
SFO1 100.5976818 MHz
======== CHANNEL f2 ========
CPDPRG2 waltz16
NUC2 1H
PCPD2 90.00 usec
PL2 -3.20 dB
PL12 14.30 dB
PL13 15.30 dB
PL2W 15.00949383 W
PL12W 0.26691073 W
PL13W 0.21201472 W
SFO2 400.0316001 MHz
SI 32768
SF 100.5876240 MHz
WDW EM
SSB 0
LB 1.00 Hz
GB 0
PC 1.40
77.340
77.022
76.704
43.354
40.222
31.889
30.111
29.599
29.532
29.473
29.316
29.137
26.570
22.662
14.088
N
H
Ms
N-dodecylmethanesulfonamide
190 180 170 160 150 140 130 120 110 100 90 80 70 60 50 40 30 20 10 ppm

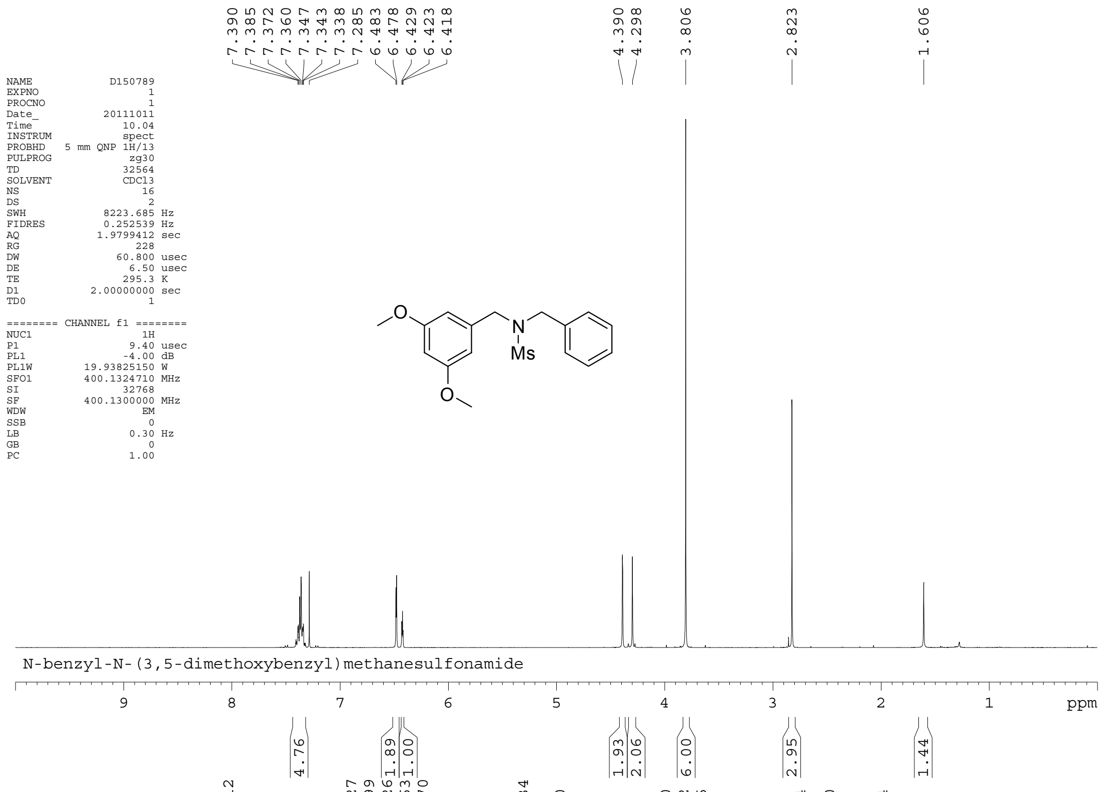
7.390
7.385
7.372
7.360
7.347
7.343
7.338
7.285
6.483
6.478
6.429
6.423
6.418
4.390
4.298
3.806
2.823
1.606
NAME D150789
EXPNO 1
PROCNO 1
Date_ 20111011
Time 10.04
INSTRUM spect
PROBHD 5 mm QNP 1H/13
PULPROG zg30
TD 32564
SOLVENT CDCl3
NS 16
DS 2
SWH 8223.685 Hz
FIDRES 0.252539 Hz
AQ 1.9799412 sec
RG 228
DW 60.800 usec
DE 6.50 usec
TE 295.3 K
D1 2.00000000 sec
TD0 1
======== CHANNEL f1 ========
NUC1 1H
P1 9.40 usec
PL1 -4.00 dB
PL1W 19.93825150 W
SFO1 400.1324710 MHz
SI 32768
SF 400.1300000 MHz
WDW EM
SSB 0
LB 0.30 Hz
GB 0
PC 1.00
O
N
Ms
O
N-benzyl-N-(3,5-dimethoxybenzyl)methanesulfonamide
9
8
7
6
5
4
3
2
1
ppm
4.76
1.89
1.00
1.93
2.06
6.00
2.95
1.44

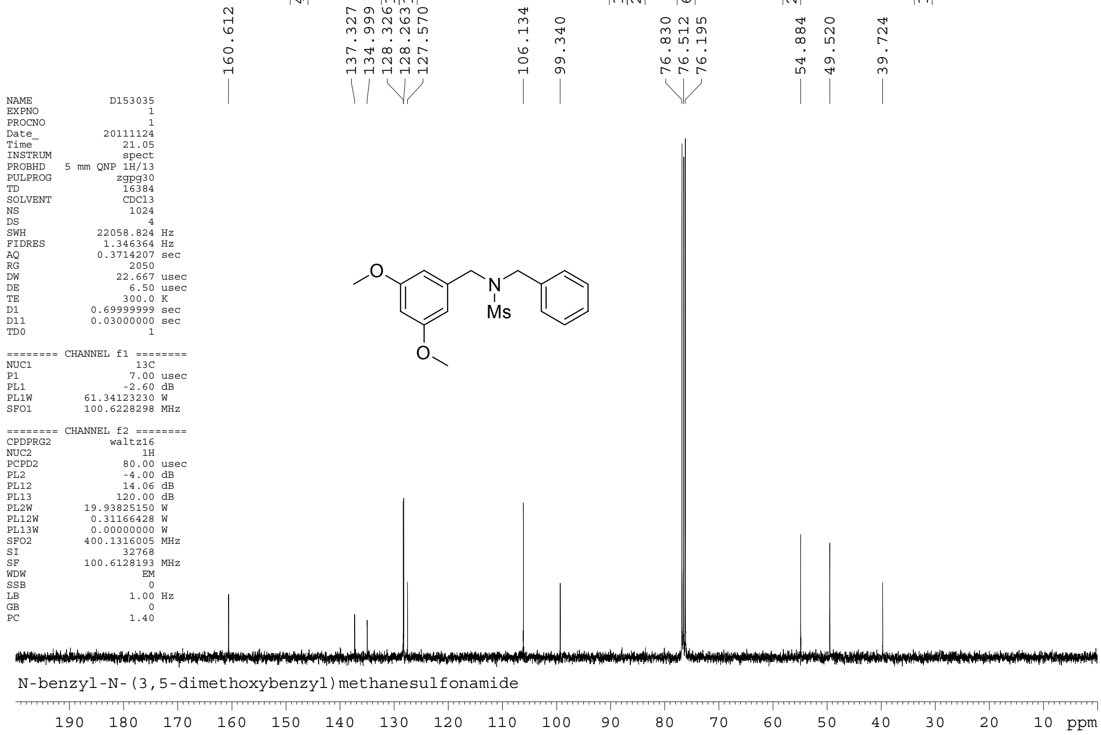
160.612
137.327
134.999
128.326
128.263
127.570
106.134
99.340
76.830
76.512
76.195
54.884
49.520
39.724
NAME D153035
EXPNO 1
PROCNO 1
Date_ 20111124
Time 21.05
INSTRUM spect
PROBHD 5 mm QNP 1H/13
PULPROG zgpg30
TD 16384
SOLVENT CDCl3
NS 1024
DS 4
SWH 22058.824 Hz
FIDRES 1.346364 Hz
AQ 0.3714207 sec
RG 2050
DW 22.667 usec
DE 6.50 usec
TE 300.0 K
D1 0.69999999 sec
D11 0.03000000 sec
TD0 1
======== CHANNEL f1 ========
NUC1 13C
P1 7.00 usec
PL1 -2.60 dB
PL1W 61.34123230 W
SFO1 100.6228298 MHz
======== CHANNEL f2 ========
CPDPRG2 waltz16
NUC2 1H
PCPD2 80.00 usec
PL2 -4.00 dB
PL12 14.06 dB
PL13 120.00 dB
PL2W 19.93825150 W
PL12W 0.31166428 W
PL13W 0.00000000 W
SFO2 400.1316005 MHz
SI 32768
SF 100.6128193 MHz
WDW EM
SSB 0
LB 1.00 Hz
GB 0
PC 1.40
O
N
Ms
O
N-benzyl-N-(3,5-dimethoxybenzyl)methanesulfonamide
190
180
170
160
150
140
130
120
110
100
90
80
70
60
50
40
30
20
10
ppm

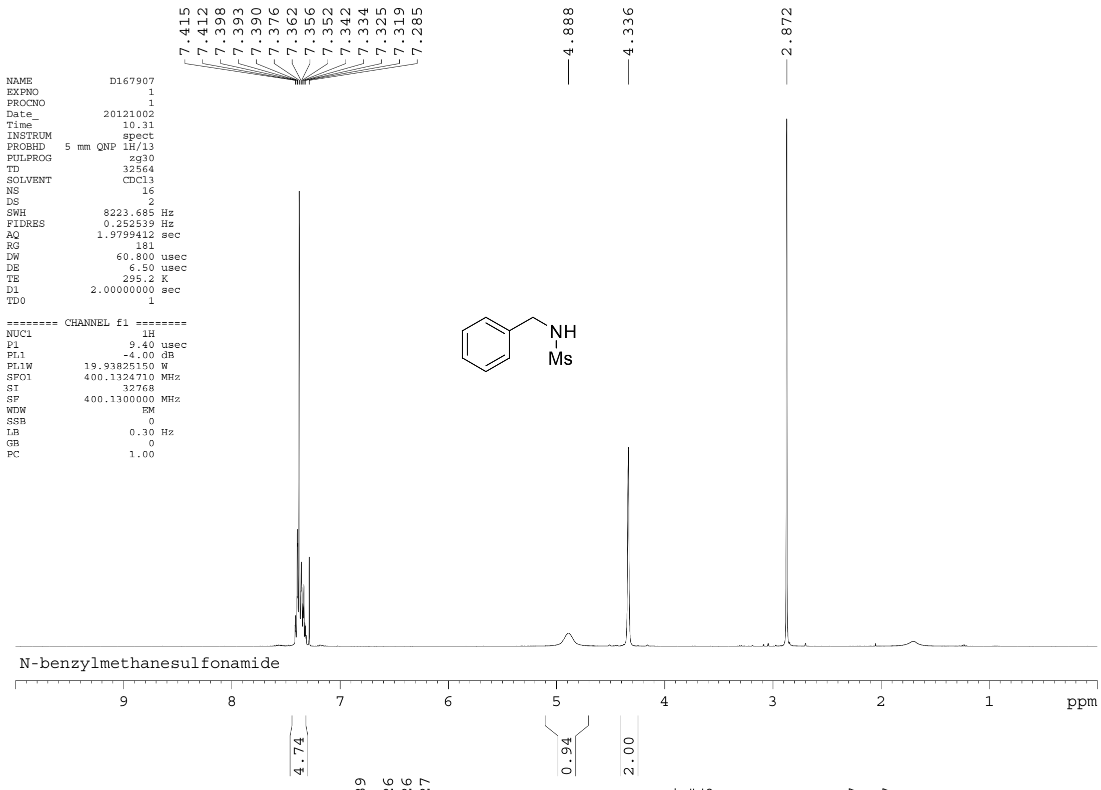
7.415
7.412
7.398
7.393
7.390
7.376
7.362
7.356
7.352
7.342
7.334
7.325
7.319
7.285
4.888
4.336
2.872
NAME D167907
EXPNO 1
PROCNO 1
Date_ 20121002
Time 10.31
INSTRUM spect
PROBHD 5 mm QNP 1H/13
PULPROG zg30
TD 32564
SOLVENT CDCl3
NS 16
DS 2
SWH 8223.685 Hz
FIDRES 0.252539 Hz
AQ 1.9799412 sec
RG 181
DW 60.800 usec
DE 6.50 usec
TE 295.2 K
D1 2.00000000 sec
TD0 1
======== CHANNEL f1 ========
NUC1 1H
P1 9.40 usec
PL1 -4.00 dB
PL1W 19.93825150 W
SFO1 400.1324710 MHz
SI 32768
SF 400.1300000 MHz
WDW EM
SSB 0
LB 0.30 Hz
GB 0
PC 1.00
NH
Ms
N-benzylmethanesulfonamide
9
8
7
6
5
4
3
2
1
ppm
4.74
0.94
2.00

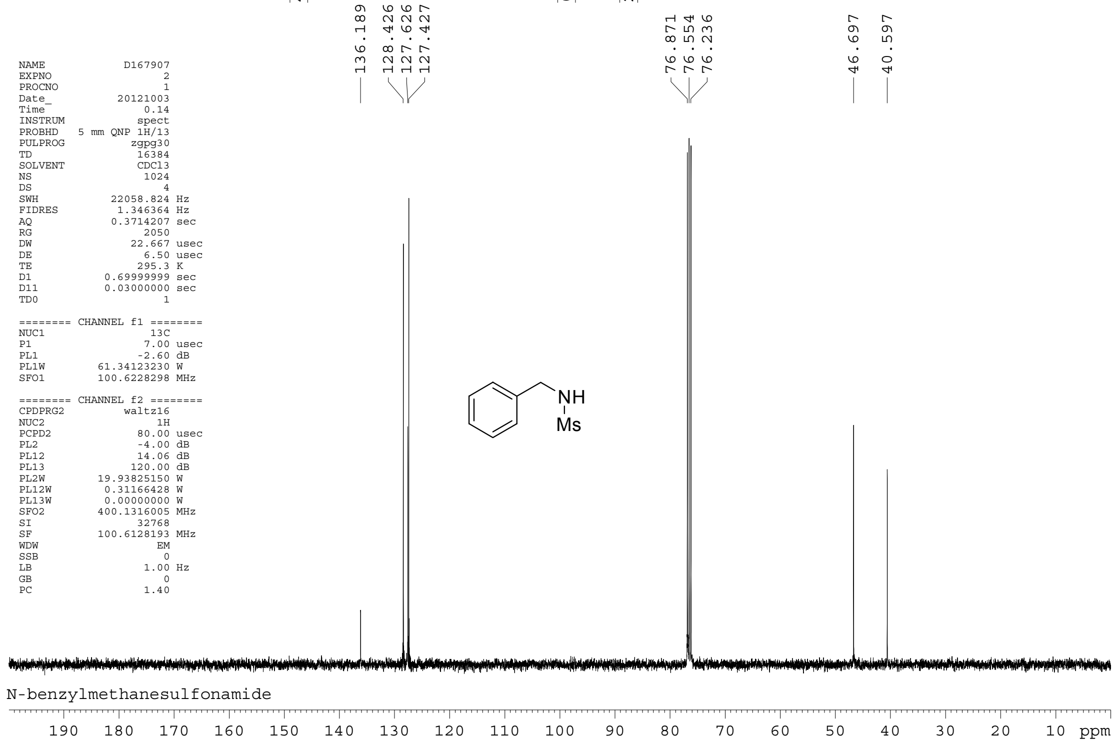
136.189
128.426
127.626
127.427
76.871
76.554
76.236
46.697
40.597
NAME D167907
EXPNO 2
PROCNO 1
Date_ 20121003
Time 0.14
INSTRUM spect
PROBHD 5 mm QNP 1H/13
PULPROG zgpg30
TD 16384
SOLVENT CDCl3
NS 1024
DS 4
SWH 22058.824 Hz
FIDRES 1.346364 Hz
AQ 0.3714207 sec
RG 2050
DW 22.667 usec
DE 6.50 usec
TE 295.3 K
D1 0.69999999 sec
D11 0.03000000 sec
TD0 1
======== CHANNEL f1 ========
NUC1 13C
P1 7.00 usec
PL1 -2.60 dB
PL1W 61.34123230 W
SFO1 100.6228298 MHz
======== CHANNEL f2 ========
CPDPRG2 waltz16
NUC2 1H
PCPD2 80.00 usec
PL2 -4.00 dB
PL12 14.06 dB
PL13 120.00 dB
PL2W 19.93825150 W
PL12W 0.31166428 W
PL13W 0.00000000 W
SFO2 400.1316005 MHz
SI 32768
SF 100.6128193 MHz
WDW EM
SSB 0
LB 1.00 Hz
GB 0
PC 1.40
NH
Ms
N-benzylmethanesulfonamide
190
180
170
160
150
140
130
120
110
100
90
80
70
60
50
40
30
20
10
ppm

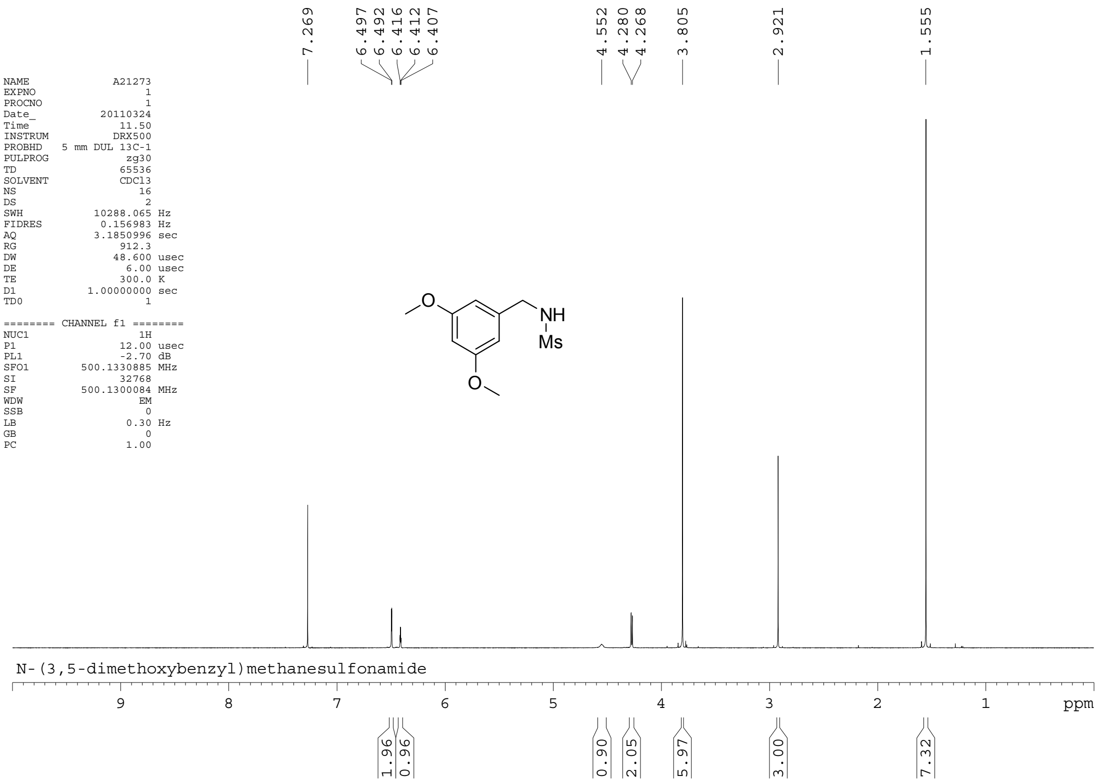
7.269
6.497
6.492
6.416
6.412
6.407
4.552
4.280
4.268
3.805
2.921
1.555
NAME A21273
EXPNO 1
PROCNO 1
Date_ 20110324
Time 11.50
INSTRUM DRX500
PROBHD 5 mm DUL 13C-1
PULPROG zg30
TD 65536
SOLVENT CDCl3
NS 16
DS 2
SWH 10288.065 Hz
FIDRES 0.156983 Hz
AQ 3.1850996 sec
RG 912.3
DW 48.600 usec
DE 6.00 usec
TE 300.0 K
D1 1.00000000 sec
TD0 1
======== CHANNEL f1 ========
NUC1 1H
P1 12.00 usec
PL1 -2.70 dB
SFO1 500.1330885 MHz
SI 32768
SF 500.1300084 MHz
WDW EM
SSB 0
LB 0.30 Hz
GB 0
PC 1.00
NH
Ms
O
O
N-(3,5-dimethoxybenzyl)methanesulfonamide
9
8
7
6
5
4
3
2
1
ppm
1.96
0.96
0.90
2.05
5.97
3.00
7.32

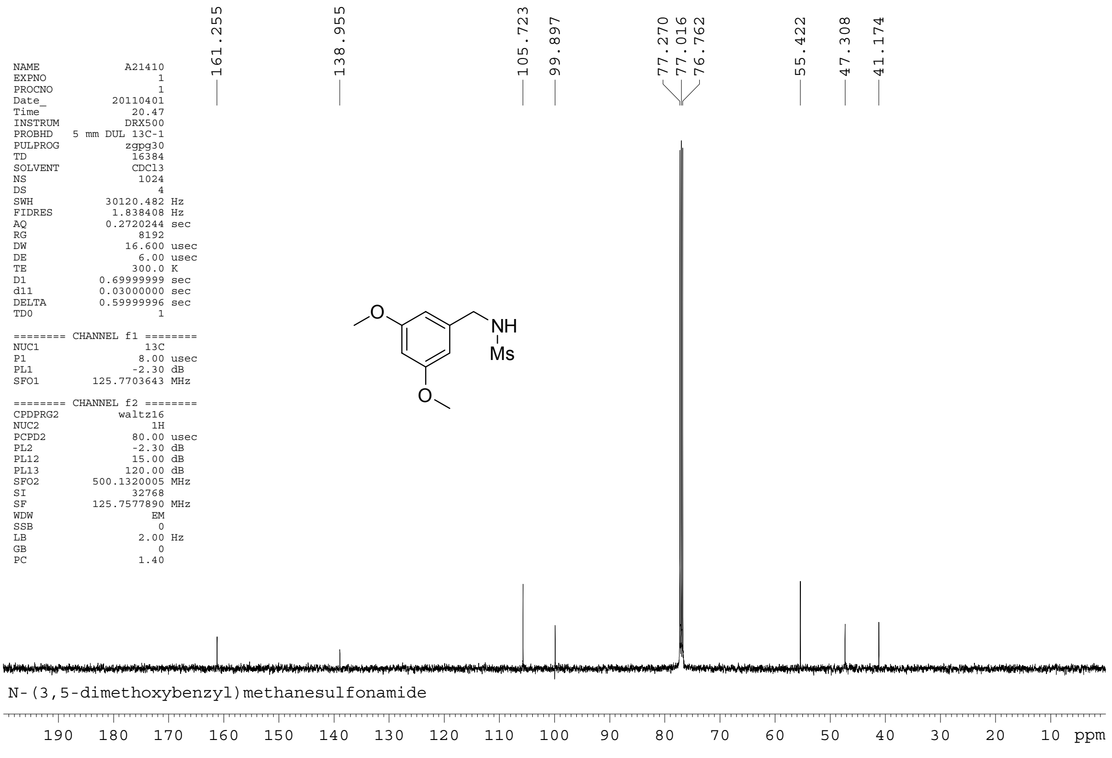
161.255
138.955
105.723
99.897
77.270
77.016
76.762
55.422
47.308
41.174
NAME A21410
EXPNO 1
PROCNO 1
Date_ 20110401
Time 20.47
INSTRUM DRX500
PROBHD 5 mm DUL 13C-1
PULPROG zgpg30
TD 16384
SOLVENT CDCl3
NS 1024
DS 4
SWH 30120.482 Hz
FIDRES 1.838408 Hz
AQ 0.2720244 sec
RG 8192
DW 16.600 usec
DE 6.00 usec
TE 300.0 K
D1 0.69999999 sec
d11 0.03000000 sec
DELTA 0.59999996 sec
TD0 1
======== CHANNEL f1 ========
NUC1 13C
P1 8.00 usec
PL1 -2.30 dB
SFO1 125.7703643 MHz
======== CHANNEL f2 ========
CPDPRG2 waltz16
NUC2 1H
PCPD2 80.00 usec
PL2 -2.30 dB
PL12 15.00 dB
PL13 120.00 dB
SFO2 500.1320005 MHz
SI 32768
SF 125.7577890 MHz
WDW EM
SSB 0
LB 2.00 Hz
GB 0
PC 1.40
NH
Ms
O
O
N-(3,5-dimethoxybenzyl)methanesulfonamide
190
180
170
160
150
140
130
120
110
100
90
80
70
60
50
40
30
20
10
ppm

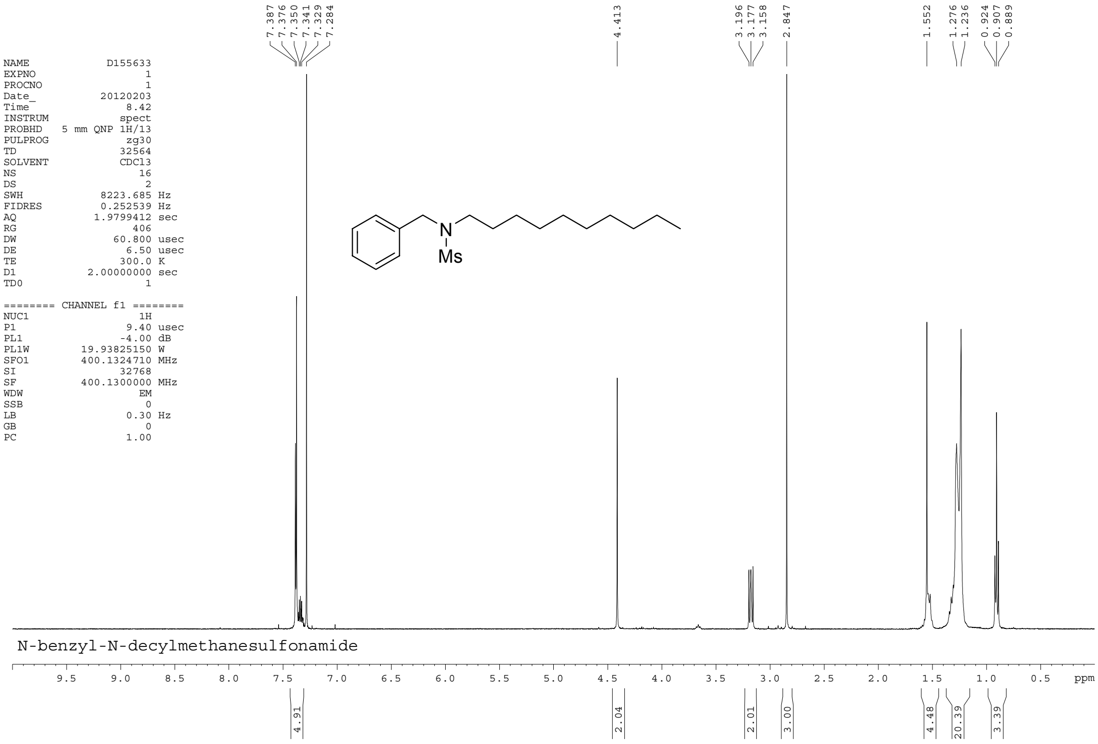
7.387
7.376
7.350
7.341
7.329
7.284
4.413
3.196
3.177
3.158
2.847
1.552
1.276
1.236
0.924
0.907
0.889
NAME D155633
EXPNO 1
PROCNO 1
Date_ 20120203
Time 8.42
INSTRUM spect
PROBHD 5 mm QNP 1H/13
PULPROG zg30
TD 32564
SOLVENT CDCl3
NS 16
DS 2
SWH 8223.685 Hz
FIDRES 0.252539 Hz
AQ 1.9799412 sec
RG 406
DW 60.800 usec
DE 6.50 usec
TE 300.0 K
D1 2.00000000 sec
TD0 1
======== CHANNEL f1 ========
NUC1 1H
P1 9.40 usec
PL1 -4.00 dB
PL1W 19.93825150 W
SFO1 400.1324710 MHz
SI 32768
SF 400.1300000 MHz
WDW EM
SSB 0
LB 0.30 Hz
GB 0
PC 1.00
N
Ms
N-benzyl-N-decylmethanesulfonamide
9.5 9.0 8.5 8.0 7.5 7.0 6.5 6.0 5.5 5.0 4.5 4.0 3.5 3.0 2.5 2.0 1.5 1.0 0.5 ppm
4.91
2.04
2.01
3.00
4.48
20.39
3.39

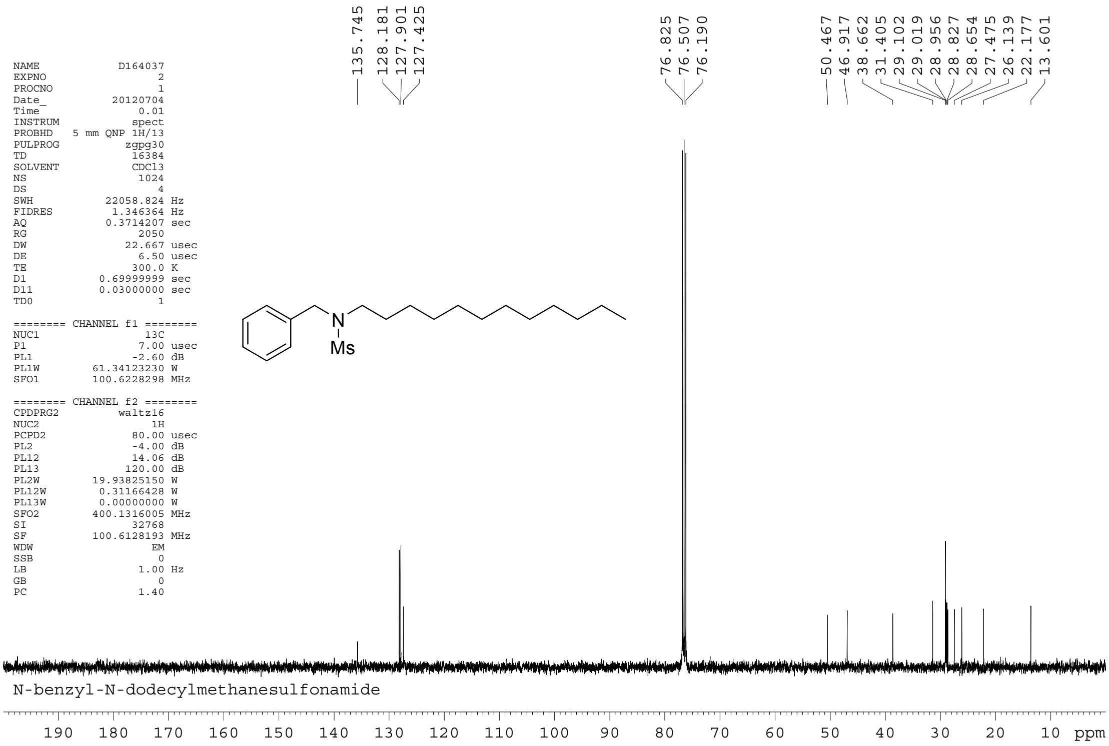
135.745
128.181
127.901
127.425
76.825
76.507
76.190
50.467
46.917
38.662
31.405
29.102
29.019
28.956
28.827
28.654
27.475
26.139
22.177
13.601
NAME D164037
EXPNO 2
PROCNO 1
Date_ 20120704
Time 0.01
INSTRUM spect
PROBHD 5 mm QNP 1H/13
PULPROG zgpg30
TD 16384
SOLVENT CDCl3
NS 1024
DS 4
SWH 22058.824 Hz
FIDRES 1.346364 Hz
AQ 0.3714207 sec
RG 2050
DW 22.667 usec
DE 6.50 usec
TE 300.0 K
D1 0.69999999 sec
D11 0.03000000 sec
TD0 1
======== CHANNEL f1 ========
NUC1 13C
P1 7.00 usec
PL1 -2.60 dB
PL1W 61.34123230 W
SFO1 100.6228298 MHz
======== CHANNEL f2 ========
CPDPRG2 waltz16
NUC2 1H
PCPD2 80.00 usec
PL2 -4.00 dB
PL12 14.06 dB
PL13 120.00 dB
PL2W 19.93825150 W
PL12W 0.31166428 W
PL13W 0.00000000 W
SFO2 400.1316005 MHz
SI 32768
SF 100.6128193 MHz
WDW EM
SSB 0
LB 1.00 Hz
GB 0
PC 1.40
N
Ms
N-benzyl-N-dodecylmethanesulfonamide
190 180 170 160 150 140 130 120 110 100 90 80 70 60 50 40 30 20 10 ppm

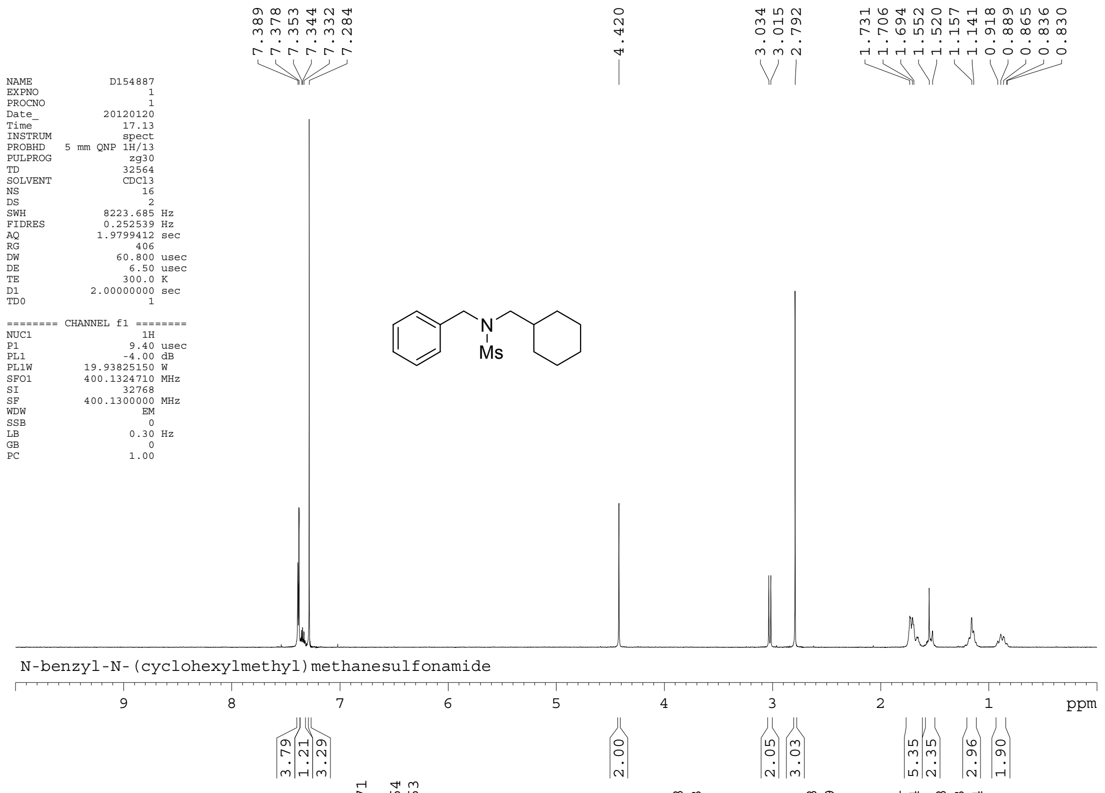
Ms
N-benzyl-N-(cyclohexylmethyl)methanesulfonamide

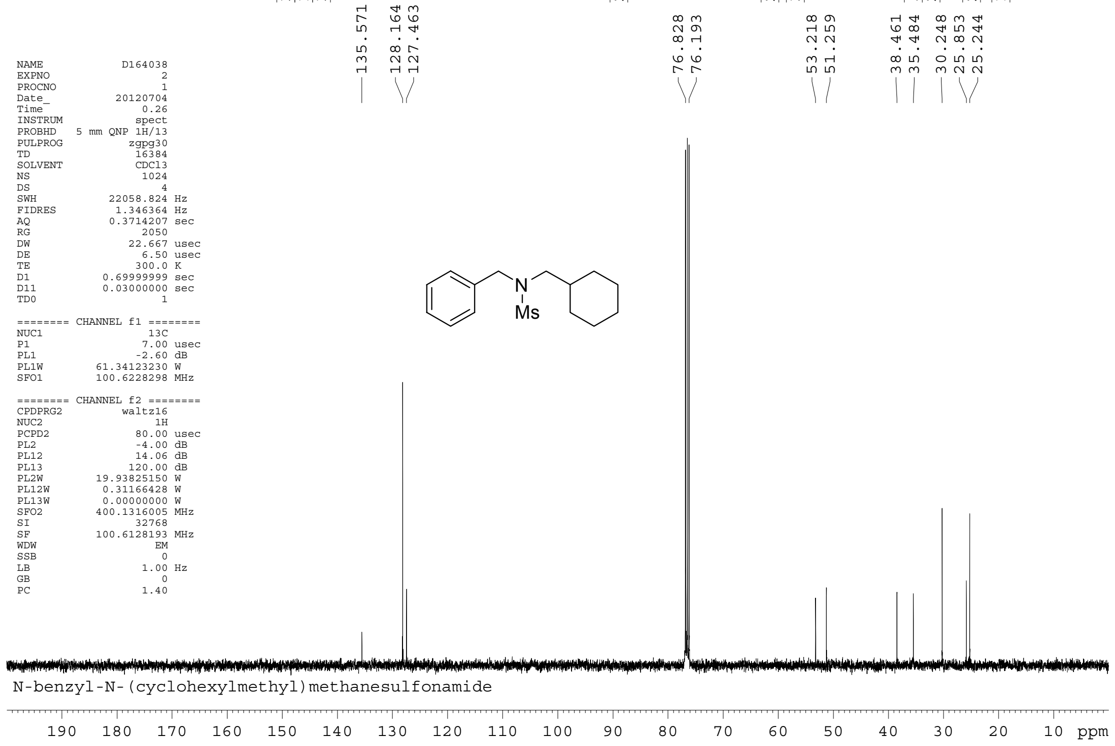
Ms
N-benzyl-N-(cyclohexylmethyl)methanesulfonamide

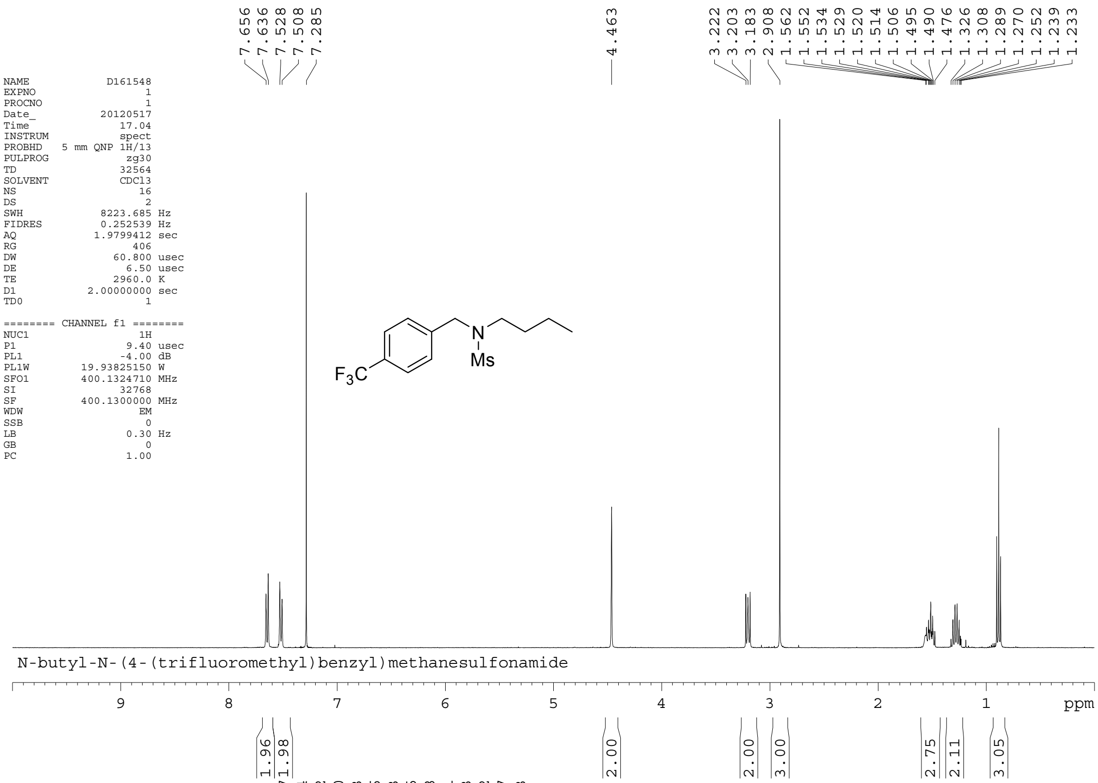

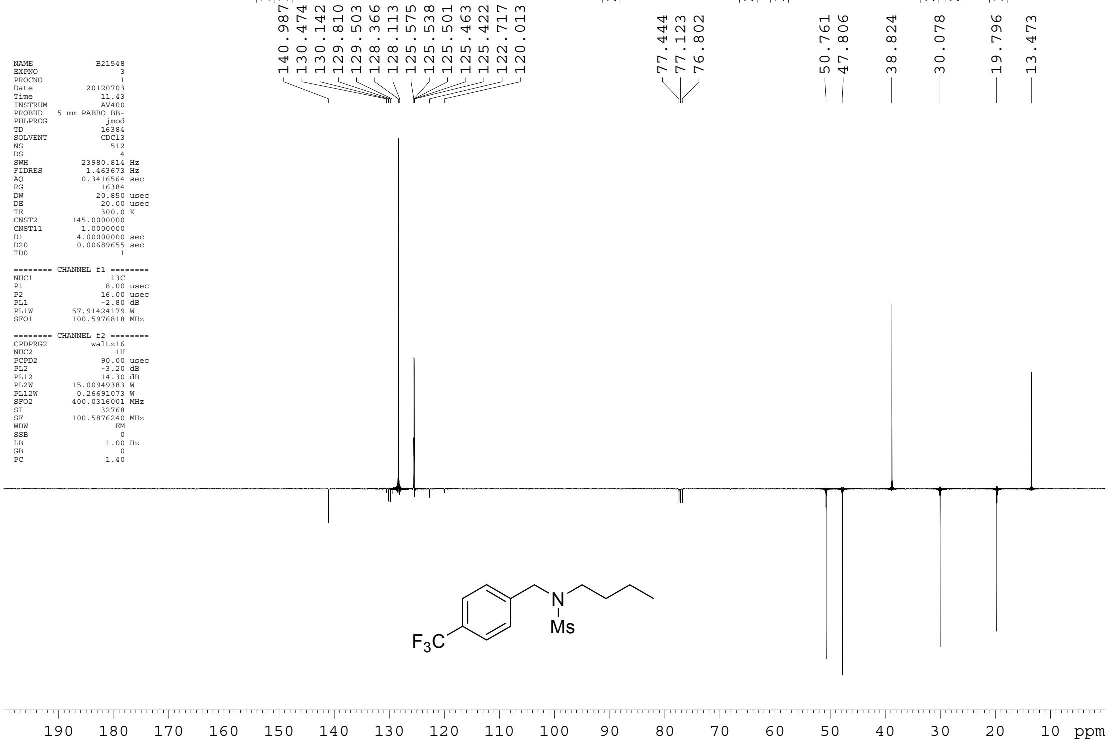

N-butyl-N-(4-(trifluoromethyl)benzyl)methanesulfonamide

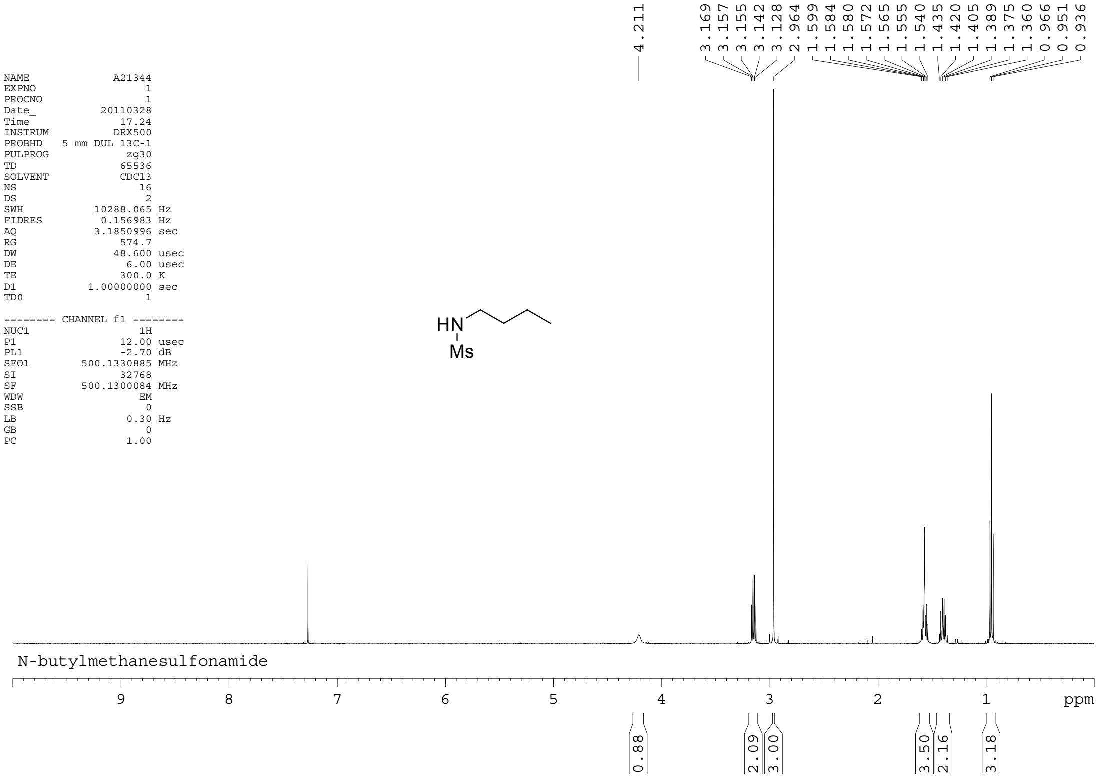
NAME A21344
EXPNO 1
PROCNO 1
Date_ 20110328
Time 17.24
INSTRUM DRX500
PROBHD 5 mm DUL 13C-1
PULPROG zg30
TD 65536
SOLVENT CDCl3
NS 16
DS 2
SWH 10288.065 Hz
FIDRES 0.156983 Hz
AQ 3.1850996 sec
RG 574.7
DW 48.600 usec
DE 6.00 usec
TE 300.0 K
D1 1.00000000 sec
TD0 1
======== CHANNEL f1 ========
NUC1 1H
P1 12.00 usec
PL1 -2.70 dB
SFO1 500.1330885 MHz
SI 32768
SF 500.1300084 MHz
WDW EM
SSB 0
LB 0.30 Hz
GB 0
PC 1.00
HN
Ms
4.211
3.169
3.157
3.155
3.142
3.128
2.964
1.599
1.584
1.580
1.572
1.565
1.555
1.540
1.435
1.420
1.405
1.389
1.375
1.360
0.966
0.951
0.936
N-butylmethanesulfonamide
9 8 7 6 5 4 3 2 1 ppm
0.88
2.09
3.00
3.50
2.16
3.18

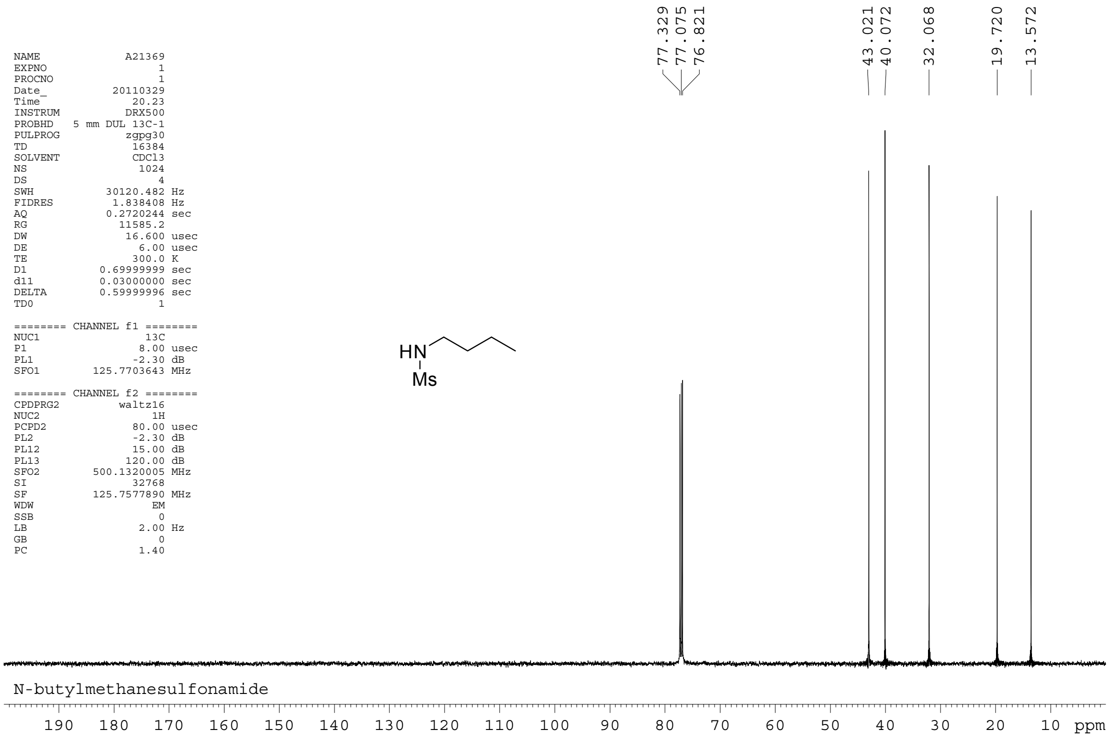
NAME A21369
EXPNO 1
PROCNO 1
Date_ 20110329
Time 20.23
INSTRUM DRX500
PROBHD 5 mm DUL 13C-1
PULPROG zgpg30
TD 16384
SOLVENT CDCl3
NS 1024
DS 4
SWH 30120.482 Hz
FIDRES 1.838408 Hz
AQ 0.2720244 sec
RG 11585.2
DW 16.600 usec
DE 6.00 usec
TE 300.0 K
D1 0.69999999 sec
d11 0.03000000 sec
DELTA 0.59999996 sec
TD0 1
======== CHANNEL f1 ========
NUC1 13C
P1 8.00 usec
PL1 -2.30 dB
SFO1 125.7703643 MHz
======== CHANNEL f2 ========
CPDPRG2 waltz16
NUC2 1H
PCPD2 80.00 usec
PL2 -2.30 dB
PL12 15.00 dB
PL13 120.00 dB
SFO2 500.1320005 MHz
SI 32768
SF 125.7577890 MHz
WDW EM
SSB 0
LB 2.00 Hz
GB 0
PC 1.40
HN
Ms
77.329
77.075
76.821
43.021
40.072
32.068
19.720
13.572
N-butylmethanesulfonamide
190 180 170 160 150 140 130 120 110 100 90 80 70 60 50 40 30 20 10 ppm

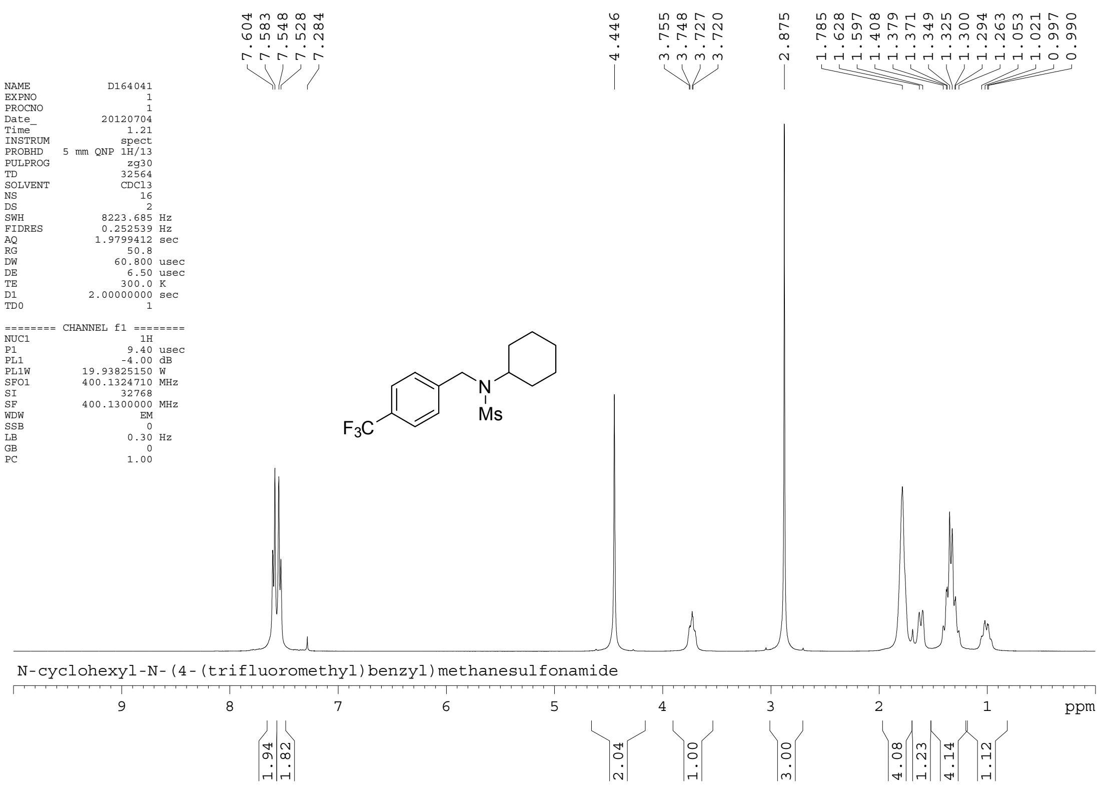
N-cyclohexyl-N-(4-(trifluoromethyl)benzyl)methanesulfonamide

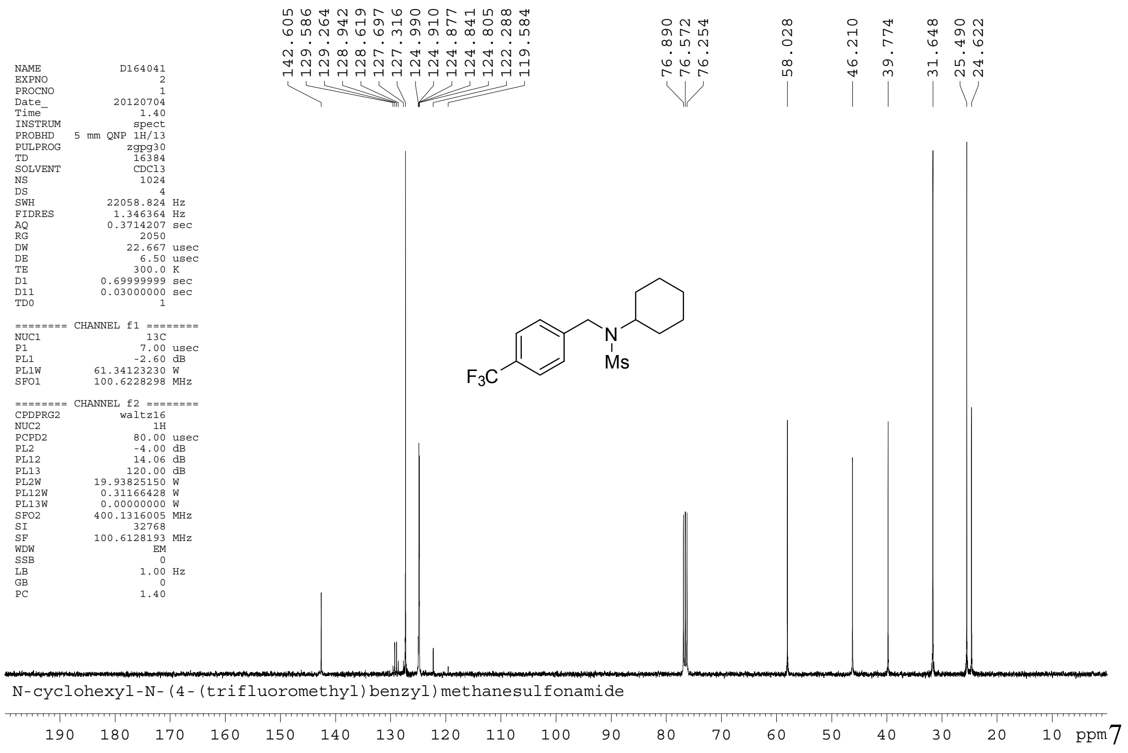
N-cyclohexyl-N-(4-(trifluoromethyl)benzyl)methanesulfonamide

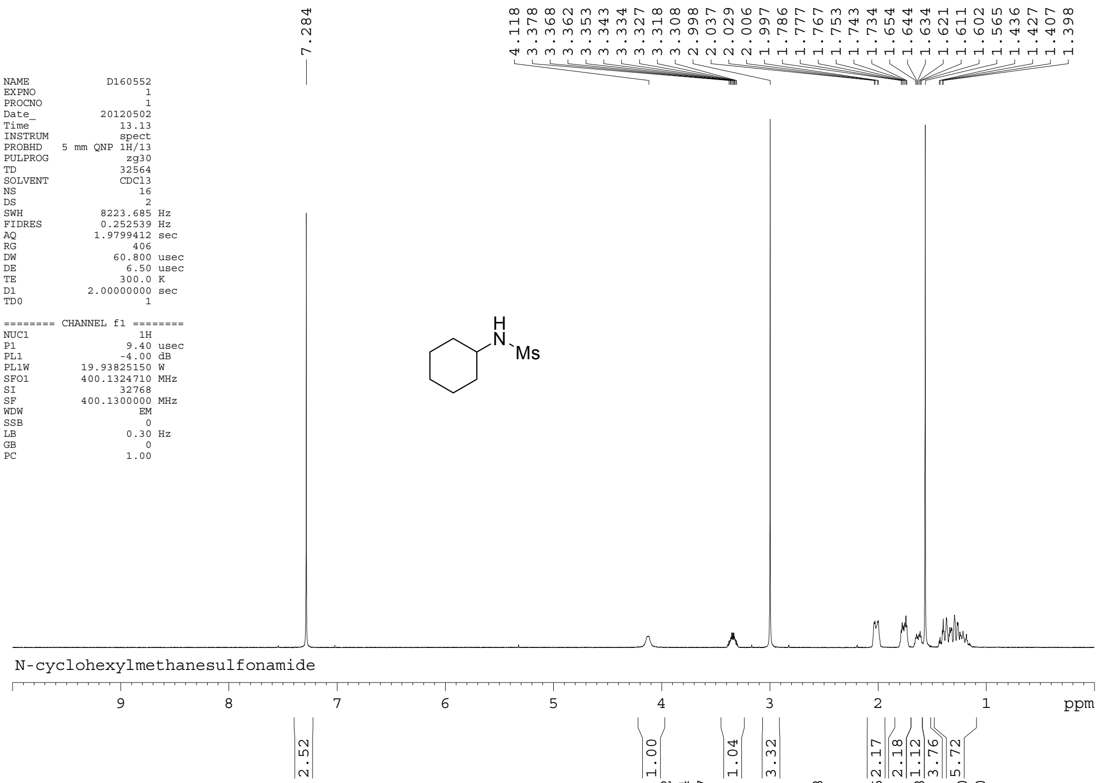
NAME D160552
EXPNO 1
PROCNO 1
Date_ 20120502
Time 13.13
INSTRUM spect
PROBHD 5 mm QNP 1H/13
PULPROG zg30
TD 32564
SOLVENT CDCl3
NS 16
DS 2
SWH 8223.685 Hz
FIDRES 0.252539 Hz
AQ 1.9799412 sec
RG 406
DW 60.800 usec
DE 6.50 usec
TE 300.0 K
D1 2.00000000 sec
TD0 1
======== CHANNEL f1 ========
NUC1 1H
P1 9.40 usec
PL1 -4.00 dB
PL1W 19.93825150 W
SFO1 400.1324710 MHz
SI 32768
SF 400.1300000 MHz
WDW EM
SSB 0
LB 0.30 Hz
GB 0
PC 1.00
7.284
4.118 3.378 3.368 3.362 3.353 3.343 3.334 3.327 3.318 3.308 2.998 2.037 2.029 2.006 1.997 1.786 1.777 1.767 1.753 1.743 1.734 1.654 1.644 1.634 1.621 1.611 1.602 1.565 1.436 1.427 1.407 1.398
H
N
Ms
N-cyclohexylmethanesulfonamide
9 8 7 6 5 4 3 2 1 ppm
2.52 1.00 1.04 3.32 2.17 2.18 1.12 3.76 5.72

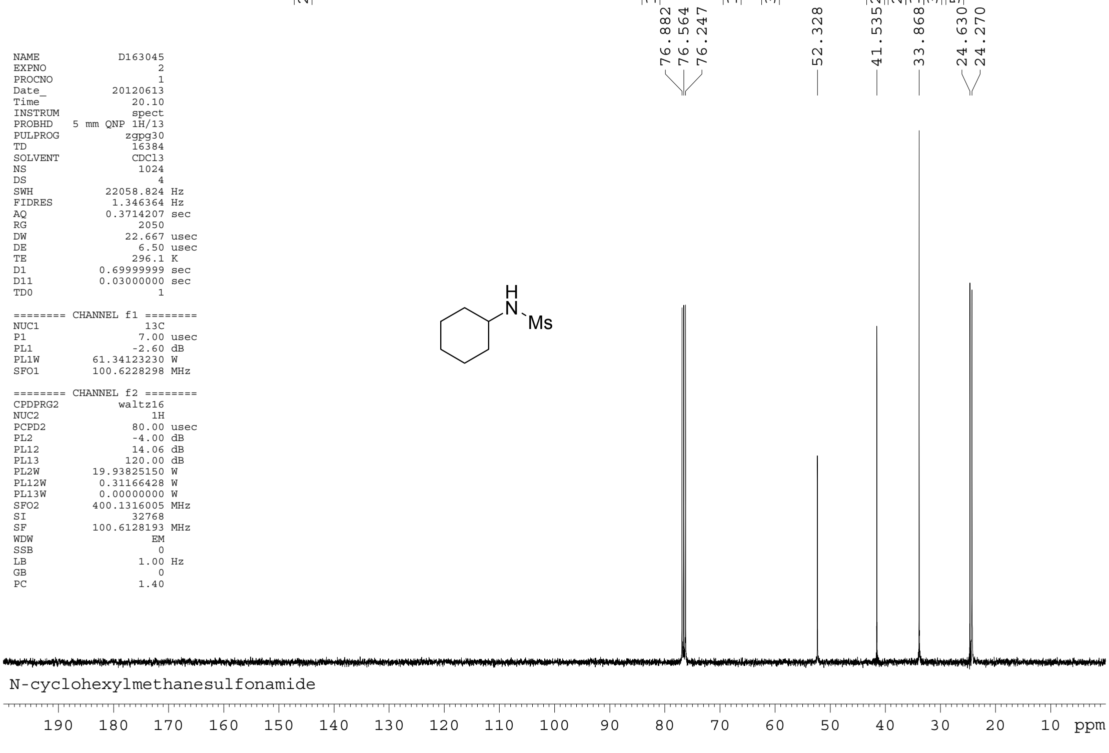
NAME D163045
EXPNO 2
PROCNO 1
Date_ 20120613
Time 20.10
INSTRUM spect
PROBHD 5 mm QNP 1H/13
PULPROG zgpg30
TD 16384
SOLVENT CDCl3
NS 1024
DS 4
SWH 22058.824 Hz
FIDRES 1.346364 Hz
AQ 0.3714207 sec
RG 2050
DW 22.667 usec
DE 6.50 usec
TE 296.1 K
D1 0.69999999 sec
D11 0.03000000 sec
TD0 1
======== CHANNEL f1 ========
NUC1 13C
P1 7.00 usec
PL1 -2.60 dB
PL1W 61.34123230 W
SFO1 100.6228298 MHz
======== CHANNEL f2 ========
CPDPRG2 waltz16
NUC2 1H
PCPD2 80.00 usec
PL2 -4.00 dB
PL12 14.06 dB
PL13 120.00 dB
PL2W 19.93825150 W
PL12W 0.31166428 W
PL13W 0.00000000 W
SFO2 400.1316005 MHz
SI 32768
SF 100.6128193 MHz
WDW EM
SSB 0
LB 1.00 Hz
GB 0
PC 1.40
76.882 76.564 76.247
52.328
41.535
33.868
24.630 24.270
H
N
Ms
N-cyclohexylmethanesulfonamide
190 180 170 160 150 140 130 120 110 100 90 80 70 60 50 40 30 20 10 ppm

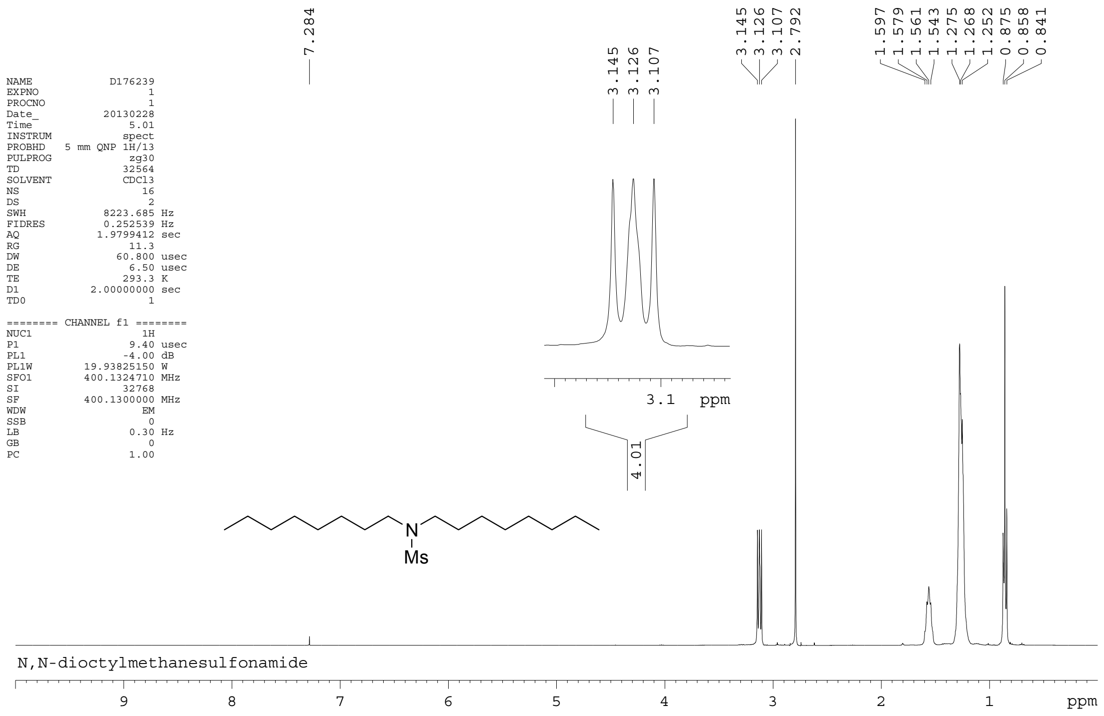
NAME D176239
EXPNO 1
PROCNO 1
Date_ 20130228
Time 5.01
INSTRUM spect
PROBHD 5 mm QNP 1H/13
PULPROG zg30
TD 32564
SOLVENT CDCl3
NS 16
DS 2
SWH 8223.685 Hz
FIDRES 0.252539 Hz
AQ 1.9799412 sec
RG 11.3
DW 60.800 usec
DE 6.50 usec
TE 293.3 K
D1 2.00000000 sec
TD0 1
======== CHANNEL f1 ========
NUC1 1H
P1 9.40 usec
PL1 -4.00 dB
PL1W 19.93825150 W
SFO1 400.1324710 MHz
SI 32768
SF 400.1300000 MHz
WDW EM
SSB 0
LB 0.30 Hz
GB 0
PC 1.00
7.284
3.145
3.126
3.107
2.792
1.597
1.579
1.561
1.543
1.275
1.268
1.252
0.875
0.858
0.841
3.1 ppm
4.01
N
Ms
N,N-dioctylmethanesulfonamide
9 8 7 6 5 4 3 2 1 ppm
4.01
3.00
4.07
20.35
6.03

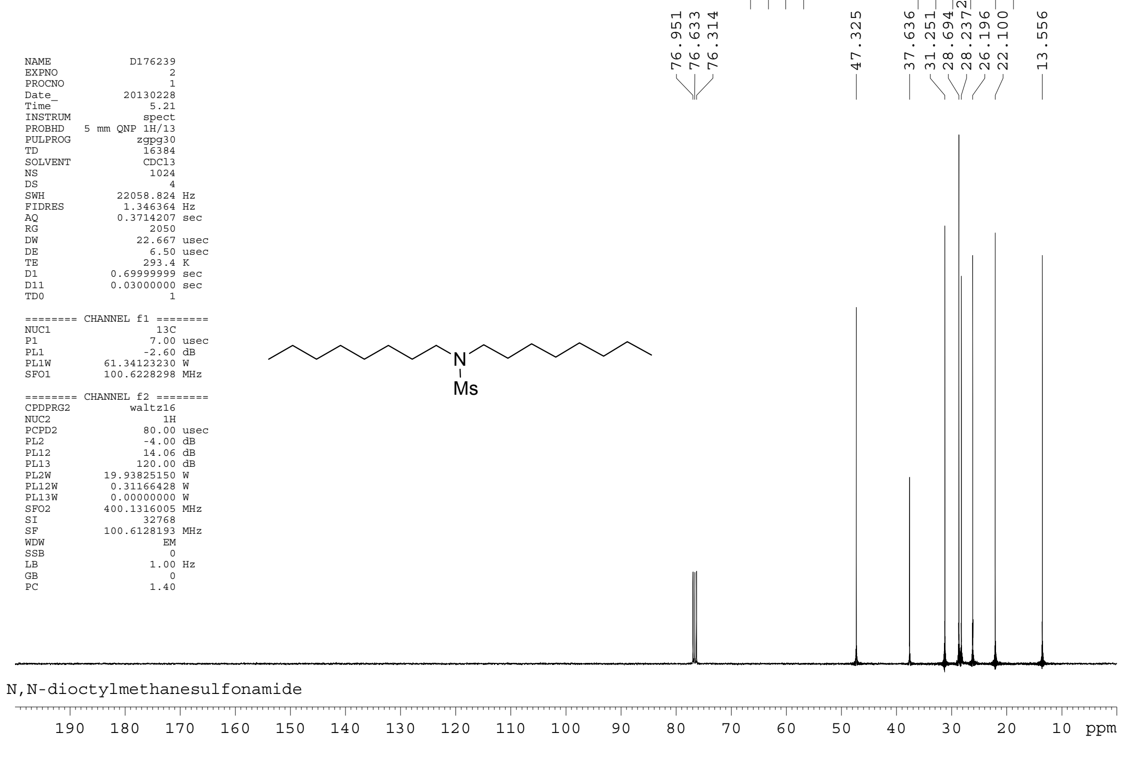
NAME D176239
EXPNO 2
PROCNO 1
Date_ 20130228
Time 5.21
INSTRUM spect
PROBHD 5 mm QNP 1H/13
PULPROG zgpg30
TD 16384
SOLVENT CDCl3
NS 1024
DS 4
SWH 22058.824 Hz
FIDRES 1.346364 Hz
AQ 0.3714207 sec
RG 2050
DW 22.667 usec
DE 6.50 usec
TE 293.4 K
D1 0.69999999 sec
D11 0.03000000 sec
TD0 1
======== CHANNEL f1 ========
NUC1 13C
P1 7.00 usec
PL1 -2.60 dB
PL1W 61.34123230 W
SFO1 100.6228298 MHz
======== CHANNEL f2 ========
CPDPRG2 waltz16
NUC2 1H
PCPD2 80.00 usec
PL2 -4.00 dB
PL12 14.06 dB
PL13 120.00 dB
PL2W 19.93825150 W
PL12W 0.31166428 W
PL13W 0.00000000 W
SFO2 400.1316005 MHz
SI 32768
SF 100.6128193 MHz
WDW EM
SSB 0
LB 1.00 Hz
GB 0
PC 1.40
76.951
76.633
76.314
47.325
37.636
31.251
28.694
28.237
26.196
22.100
13.556
N
Ms
N,N-dioctylmethanesulfonamide
190 180 170 160 150 140 130 120 110 100 90 80 70 60 50 40 30 20 10 ppm

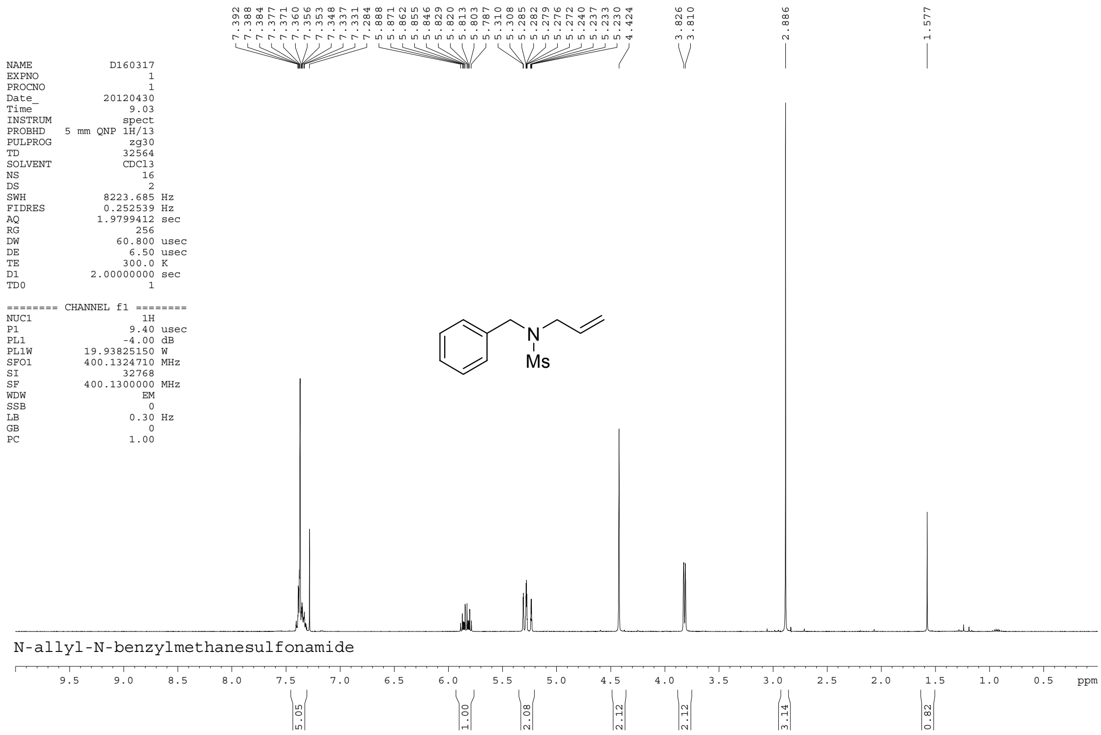
NAME D160317
EXPNO 1
PROCNO 1
Date_ 20120430
Time 9.03
INSTRUM spect
PROBHD 5 mm QNP 1H/13
PULPROG zg30
TD 32564
SOLVENT CDCl3
NS 16
DS 2
SWH 8223.685 Hz
FIDRES 0.252539 Hz
AQ 1.9799412 sec
RG 256
DW 60.800 usec
DE 6.50 usec
TE 300.0 K
D1 2.00000000 sec
TD0 1
======== CHANNEL f1 ========
NUC1 1H
P1 9.40 usec
PL1 -4.00 dB
PL1W 19.93825150 W
SFO1 400.1324710 MHz
SI 32768
SF 400.1300000 MHz
WDW EM
SSB 0
LB 0.30 Hz
GB 0
PC 1.00
N
Ms
N-allyl-N-benzylmethanesulfonamide
ppm

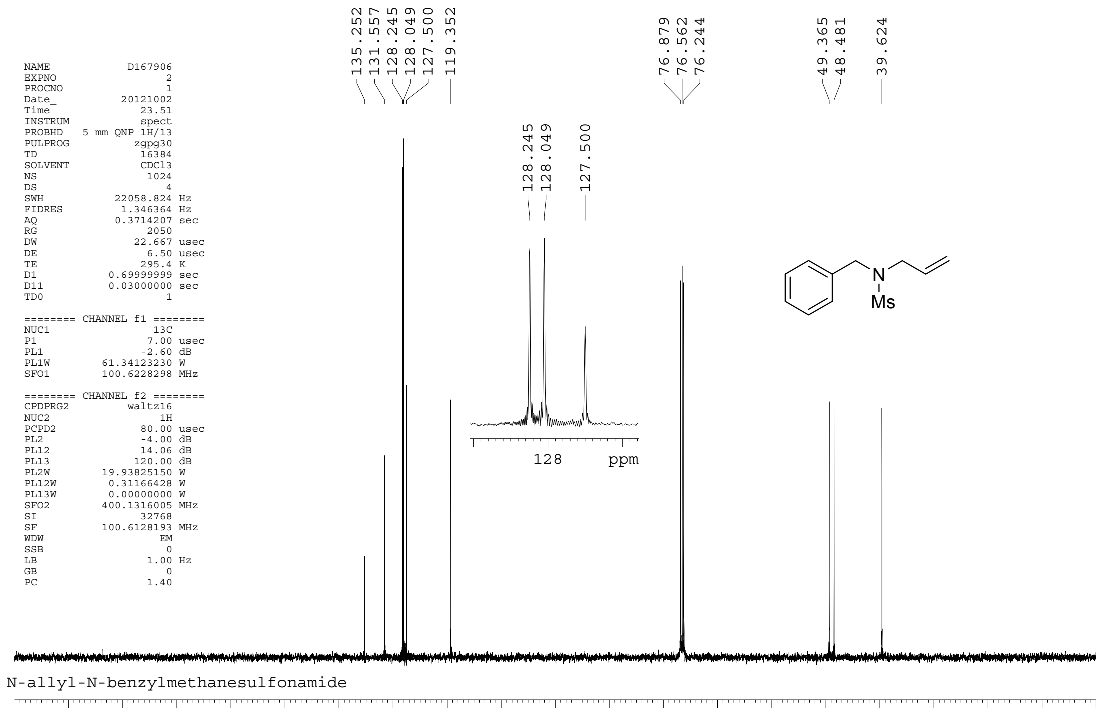
NAME D167906
EXPNO 2
PROCNO 1
Date_ 20121002
Time 23.51
INSTRUM spect
PROBHD 5 mm QNP 1H/13
PULPROG zgpg30
TD 16384
SOLVENT CDCl3
NS 1024
DS 4
SWH 22058.824 Hz
FIDRES 1.346364 Hz
AQ 0.3714207 sec
RG 2050
DW 22.667 usec
DE 6.50 usec
TE 295.4 K
D1 0.69999999 sec
D11 0.03000000 sec
TD0 1
======== CHANNEL f1 ========
NUC1 13C
P1 7.00 usec
PL1 -2.60 dB
PL1W 61.34123230 W
SFO1 100.6228298 MHz
======== CHANNEL f2 ========
CPDPRG2 waltz16
NUC2 1H
PCPD2 80.00 usec
PL2 -4.00 dB
PL12 14.06 dB
PL13 120.00 dB
PL2W 19.93825150 W
PL12W 0.31166428 W
PL13W 0.00000000 W
SFO2 400.1316005 MHz
SI 32768
SF 100.6128193 MHz
WDW EM
SSB 0
LB 1.00 Hz
GB 0
PC 1.40
N
Ms
N-allyl-N-benzylmethanesulfonamide
ppm

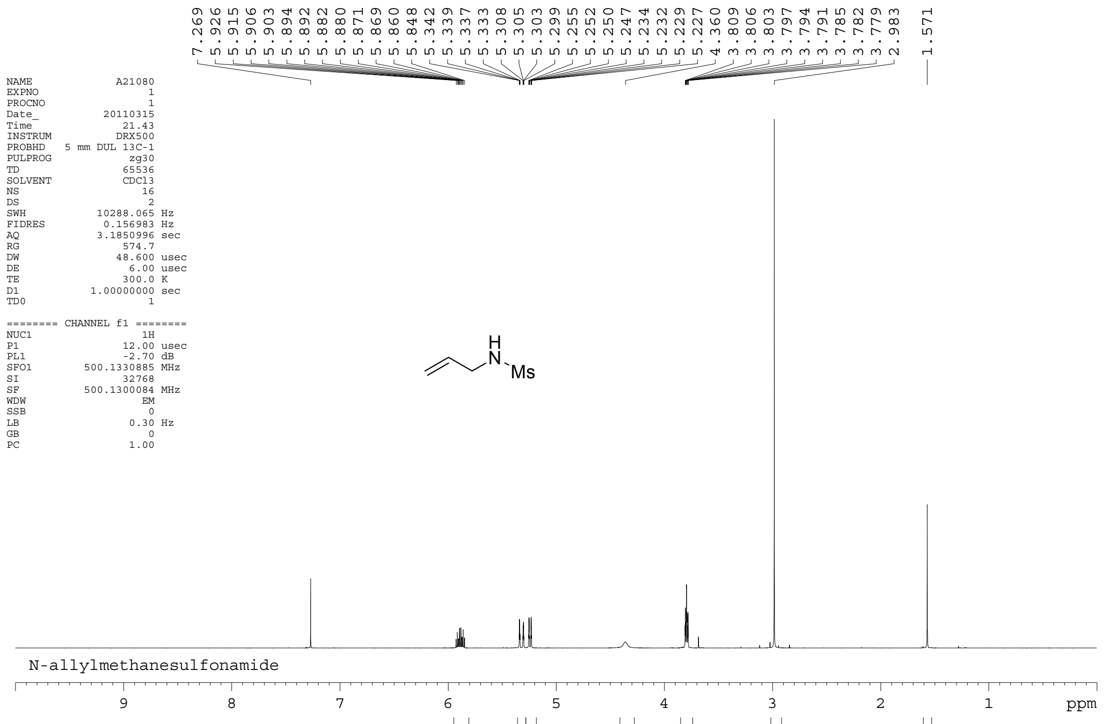
7.269 5.926 5.915 5.906 5.903 5.894 5.892 5.882 5.880 5.871 5.869 5.860 5.848 5.342 5.339 5.337 5.333 5.308 5.305 5.303 5.299 5.255 5.252 5.250 5.247 5.234 5.232 5.229 5.227 4.360 3.809 3.806 3.803 3.797 3.794 3.791 3.785 3.782 3.779 2.983 1.571
NAME A21080
EXPNO 1
PROCNO 1
Date_ 20110315
Time 21.43
INSTRUM DRX500
PROBHD 5 mm DUL 13C-1
PULPROG zg30
TD 65536
SOLVENT CDCl3
NS 16
DS 2
SWH 10288.065 Hz
FIDRES 0.156983 Hz
AQ 3.1850996 sec
RG 574.7
DW 48.600 usec
DE 6.00 usec
TE 300.0 K
D1 1.00000000 sec
TD0 1
======== CHANNEL f1 ========
NUC1 1H
P1 12.00 usec
PL1 -2.70 dB
SFO1 500.1330885 MHz
SI 32768
SF 500.1300084 MHz
WDW EM
SSB 0
LB 0.30 Hz
GB 0
PC 1.00
H
N
Ms
N-allylmethanesulfonamide
9 8 7 6 5 4 3 2 1 ppm
1.00 1.04 1.04 0.90 2.13 3.26 1.07

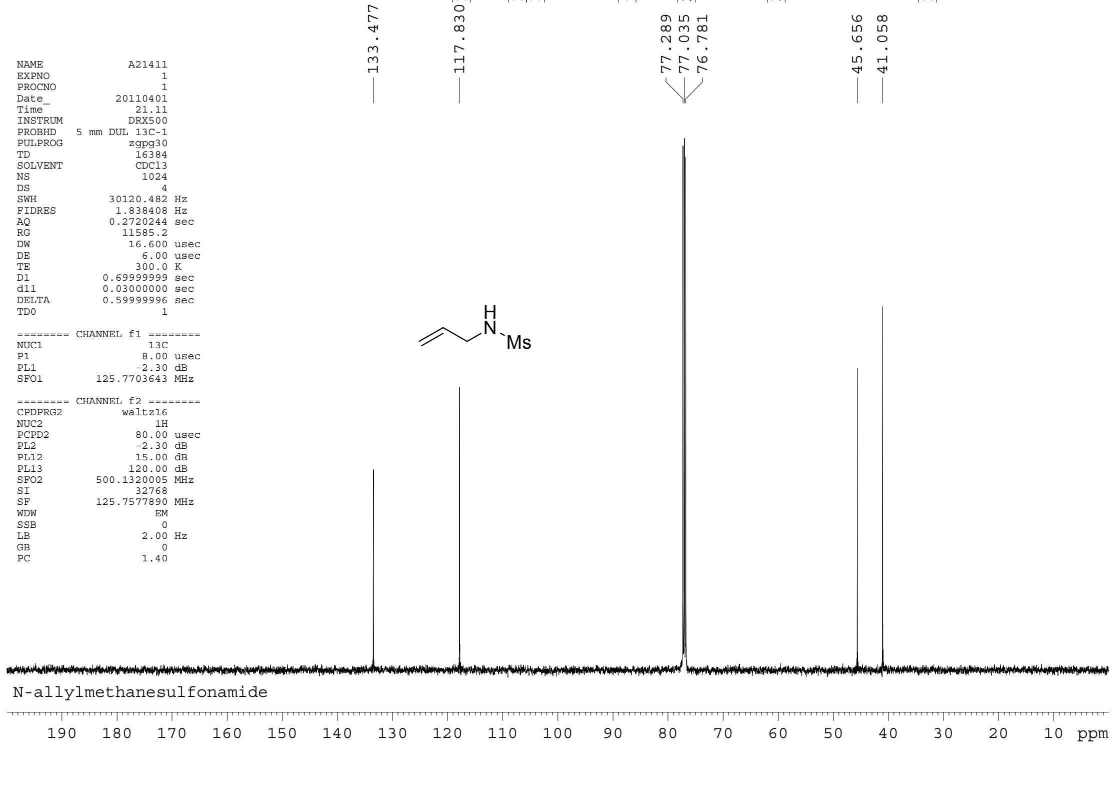
133.477 117.830 77.289 77.035 76.781 45.656 41.058
NAME A21411
EXPNO 1
PROCNO 1
Date_ 20110401
Time 21.11
INSTRUM DRX500
PROBHD 5 mm DUL 13C-1
PULPROG zgpg30
TD 16384
SOLVENT CDCl3
NS 1024
DS 4
SWH 30120.482 Hz
FIDRES 1.838408 Hz
AQ 0.2720244 sec
RG 11585.2
DW 16.600 usec
DE 6.00 usec
TE 300.0 K
D1 0.69999999 sec
d11 0.03000000 sec
DELTA 0.59999996 sec
TD0 1
======== CHANNEL f1 ========
NUC1 13C
P1 8.00 usec
PL1 -2.30 dB
SFO1 125.7703643 MHz
======== CHANNEL f2 ========
CPDPRG2 waltz16
NUC2 1H
PCPD2 80.00 usec
PL2 -2.30 dB
PL12 15.00 dB
PL13 120.00 dB
SFO2 500.1320005 MHz
SI 32768
SF 125.7577890 MHz
WDW EM
SSB 0
LB 2.00 Hz
GB 0
PC 1.40
H
N
Ms
N-allylmethanesulfonamide
190 180 170 160 150 140 130 120 110 100 90 80 70 60 50 40 30 20 10 ppm

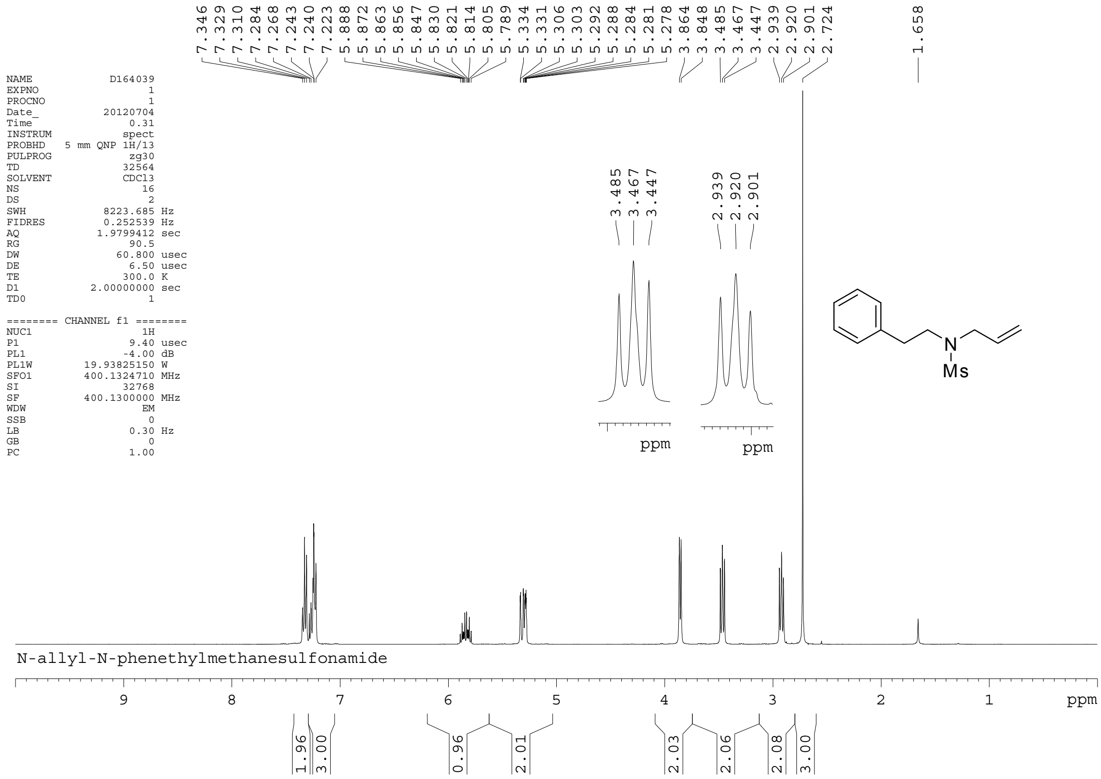
7.346 7.329 7.310 7.284 7.268 7.243 7.240 7.223 5.888 5.872 5.863 5.856 5.847 5.830 5.821 5.814 5.805 5.789 5.334 5.331 5.306 5.303 5.292 5.288 5.284 5.281 5.278 3.864 3.848 3.485 3.467 3.447 2.939 2.920 2.901 2.724 1.658
NAME D164039
EXPNO 1
PROCNO 1
Date_ 20120704
Time 0.31
INSTRUM spect
PROBHD 5 mm QNP 1H/13
PULPROG zg30
TD 32564
SOLVENT CDCl3
NS 16
DS 2
SWH 8223.685 Hz
FIDRES 0.252539 Hz
AQ 1.9799412 sec
RG 90.5
DW 60.800 usec
DE 6.50 usec
TE 300.0 K
D1 2.00000000 sec
TD0 1
======== CHANNEL f1 ========
NUC1 1H
P1 9.40 usec
PL1 -4.00 dB
PL1W 19.93825150 W
SFO1 400.1324710 MHz
SI 32768
SF 400.1300000 MHz
WDW EM
SSB 0
LB 0.30 Hz
GB 0
PC 1.00
3.485 3.467 3.447
2.939 2.920 2.901
ppm
ppm
N
Ms
N-allyl-N-phenethylmethanesulfonamide
9 8 7 6 5 4 3 2 1 ppm
1.96 3.00 0.96 2.01 2.03 2.06 2.08 3.00

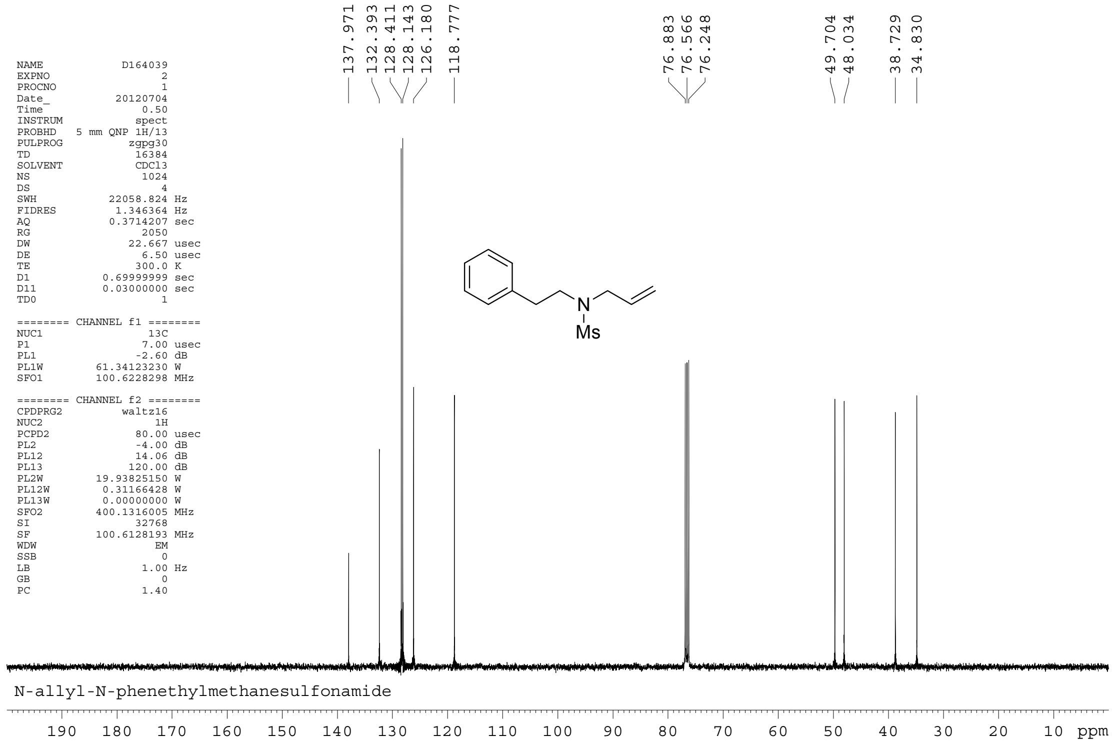
137.971 132.393 128.411 128.143 126.180 118.777 76.883 76.566 76.248 49.704 48.034 38.729 34.830
NAME D164039
EXPNO 2
PROCNO 1
Date_ 20120704
Time 0.50
INSTRUM spect
PROBHD 5 mm QNP 1H/13
PULPROG zgpg30
TD 16384
SOLVENT CDCl3
NS 1024
DS 4
SWH 22058.824 Hz
FIDRES 1.346364 Hz
AQ 0.3714207 sec
RG 2050
DW 22.667 usec
DE 6.50 usec
TE 300.0 K
D1 0.69999999 sec
D11 0.03000000 sec
TD0 1
======== CHANNEL f1 ========
NUC1 13C
P1 7.00 usec
PL1 -2.60 dB
PL1W 61.34123230 W
SFO1 100.6228298 MHz
======== CHANNEL f2 ========
CPDPRG2 waltz16
NUC2 1H
PCPD2 80.00 usec
PL2 -4.00 dB
PL12 14.06 dB
PL13 120.00 dB
PL2W 19.93825150 W
PL12W 0.31166428 W
PL13W 0.00000000 W
SFO2 400.1316005 MHz
SI 32768
SF 100.6128193 MHz
WDW EM
SSB 0
LB 1.00 Hz
GB 0
PC 1.40
N
Ms
N-allyl-N-phenethylmethanesulfonamide
190 180 170 160 150 140 130 120 110 100 90 80 70 60 50 40 30 20 10 ppm

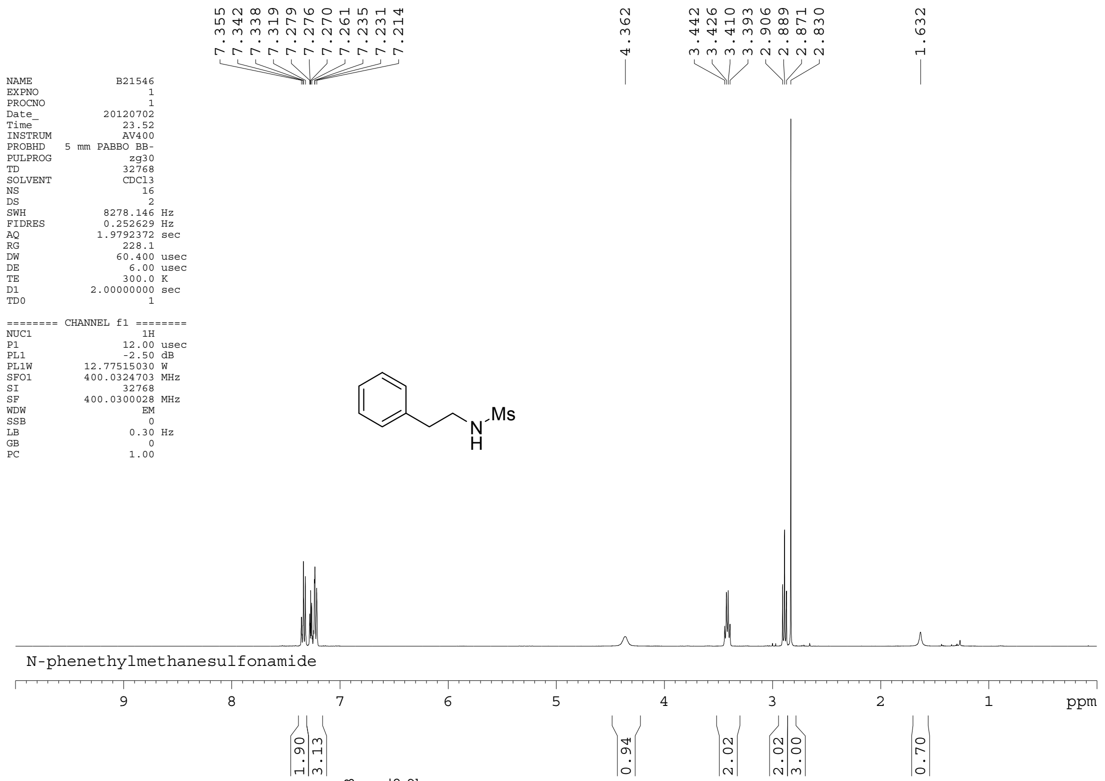
7.355
7.342
7.338
7.319
7.279
7.276
7.270
7.261
7.235
7.231
7.214
4.362
3.442
3.426
3.410
3.393
2.906
2.889
2.871
2.830
1.632
NAME B21546
EXPNO 1
PROCNO 1
Date_ 20120702
Time 23.52
INSTRUM AV400
PROBHD 5 mm PABBO BB-
PULPROG zg30
TD 32768
SOLVENT CDCl3
NS 16
DS 2
SWH 8278.146 Hz
FIDRES 0.252629 Hz
AQ 1.9792372 sec
RG 228.1
DW 60.400 usec
DE 6.00 usec
TE 300.0 K
D1 2.00000000 sec
TD0 1
======== CHANNEL f1 ========
NUC1 1H
P1 12.00 usec
PL1 -2.50 dB
PL1W 12.77515030 W
SFO1 400.0324703 MHz
SI 32768
SF 400.0300028 MHz
WDW EM
SSB 0
LB 0.30 Hz
GB 0
PC 1.00
N
H
Ms
N-phenethylmethanesulfonamide
9
8
7
6
5
4
3
2
1
ppm
1.90
3.13
0.94
2.02
2.02
3.00
0.70

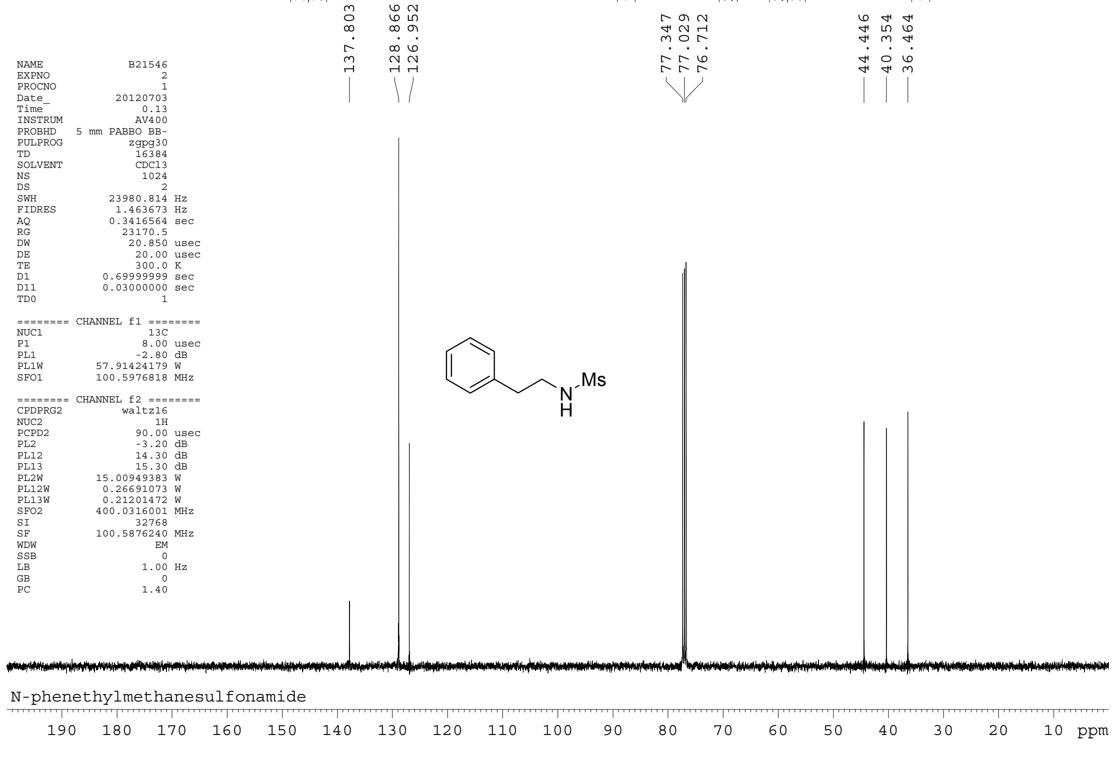
137.803
128.866
126.952
77.347
77.029
76.712
44.446
40.354
36.464
NAME B21546
EXPNO 2
PROCNO 1
Date_ 20120703
Time 0.13
INSTRUM AV400
PROBHD 5 mm PABBO BB-
PULPROG zgpg30
TD 16384
SOLVENT CDCl3
NS 1024
DS 2
SWH 23980.814 Hz
FIDRES 1.463673 Hz
AQ 0.3416564 sec
RG 23170.5
DW 20.850 usec
DE 20.00 usec
TE 300.0 K
D1 0.69999999 sec
D11 0.03000000 sec
TD0 1
======== CHANNEL f1 ========
NUC1 13C
P1 8.00 usec
PL1 -2.80 dB
PL1W 57.91424179 W
SFO1 100.5976818 MHz
======== CHANNEL f2 ========
CPDPRG2 waltz16
NUC2 1H
PCPD2 90.00 usec
PL2 -3.20 dB
PL12 14.30 dB
PL13 15.30 dB
PL2W 15.00949383 W
PL12W 0.26691073 W
PL13W 0.21201472 W
SFO2 400.0316001 MHz
SI 32768
SF 100.5876240 MHz
WDW EM
SSB 0
LB 1.00 Hz
GB 0
PC 1.40
N
H
Ms
N-phenethylmethanesulfonamide
190
180
170
160
150
140
130
120
110
100
90
80
70
60
50
40
30
20
10
ppm

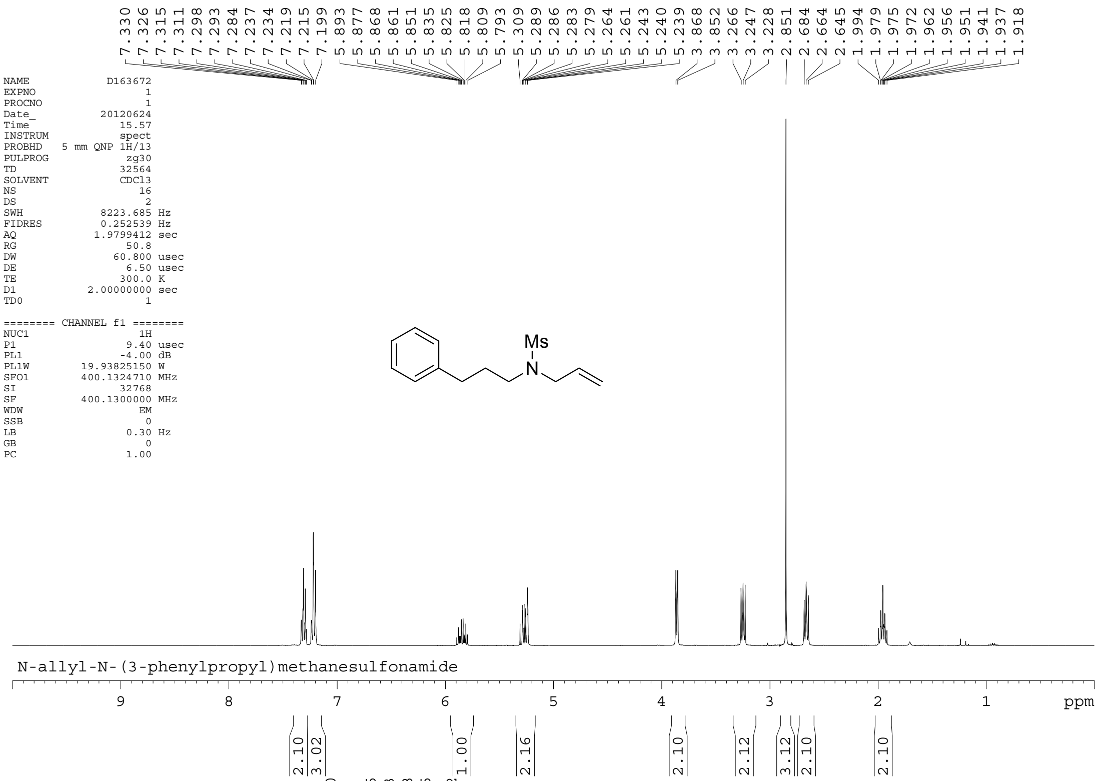
7.330
7.326
7.315
7.311
7.298
7.293
7.284
7.237
7.234
7.219
7.215
7.199
5.893
5.877
5.868
5.861
5.851
5.835
5.825
5.818
5.809
5.793
5.309
5.289
5.286
5.283
5.279
5.264
5.261
5.243
5.240
5.239
3.868
3.852
3.266
3.247
3.228
2.851
2.684
2.664
2.645
1.994
1.979
1.975
1.972
1.962
1.956
1.951
1.941
1.937
1.918
NAME D163672
EXPNO 1
PROCNO 1
Date_ 20120624
Time 15.57
INSTRUM spect
PROBHD 5 mm QNP 1H/13
PULPROG zg30
TD 32564
SOLVENT CDCl3
NS 16
DS 2
SWH 8223.685 Hz
FIDRES 0.252539 Hz
AQ 1.9799412 sec
RG 50.8
DW 60.800 usec
DE 6.50 usec
TE 300.0 K
D1 2.00000000 sec
TD0 1
======== CHANNEL f1 ========
NUC1 1H
P1 9.40 usec
PL1 -4.00 dB
PL1W 19.93825150 W
SFO1 400.1324710 MHz
SI 32768
SF 400.1300000 MHz
WDW EM
SSB 0
LB 0.30 Hz
GB 0
PC 1.00
Ms
N
N-allyl-N-(3-phenylpropyl)methanesulfonamide
9
8
7
6
5
4
3
2
1
ppm
2.10
3.02
1.00
2.16
2.10
2.12
3.12
2.10
2.10

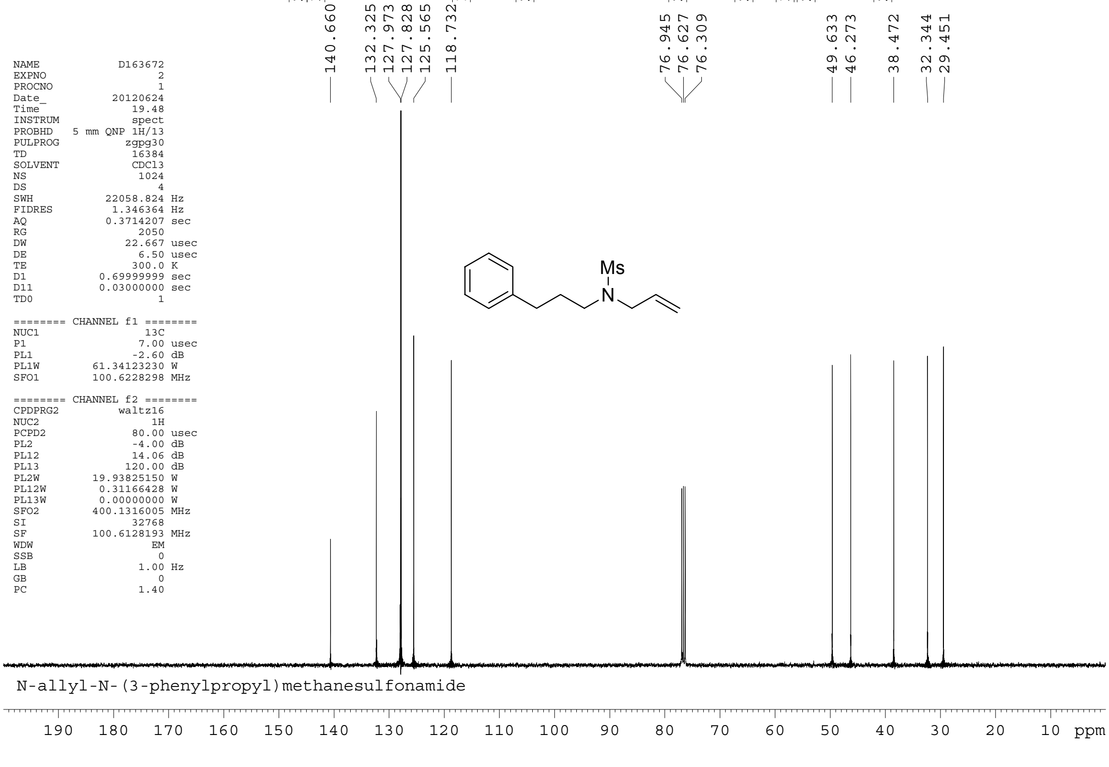
140.660
132.325
127.973
127.828
125.565
118.732
76.945
76.627
76.309
49.633
46.273
38.472
32.344
29.451
NAME D163672
EXPNO 2
PROCNO 1
Date_ 20120624
Time 19.48
INSTRUM spect
PROBHD 5 mm QNP 1H/13
PULPROG zgpg30
TD 16384
SOLVENT CDCl3
NS 1024
DS 4
SWH 22058.824 Hz
FIDRES 1.346364 Hz
AQ 0.3714207 sec
RG 2050
DW 22.667 usec
DE 6.50 usec
TE 300.0 K
D1 0.69999999 sec
D11 0.03000000 sec
TD0 1
======== CHANNEL f1 ========
NUC1 13C
P1 7.00 usec
PL1 -2.60 dB
PL1W 61.34123230 W
SFO1 100.6228298 MHz
======== CHANNEL f2 ========
CPDPRG2 waltz16
NUC2 1H
PCPD2 80.00 usec
PL2 -4.00 dB
PL12 14.06 dB
PL13 120.00 dB
PL2W 19.93825150 W
PL12W 0.31166428 W
PL13W 0.00000000 W
SFO2 400.1316005 MHz
SI 32768
SF 100.6128193 MHz
WDW EM
SSB 0
LB 1.00 Hz
GB 0
PC 1.40
Ms
N
N-allyl-N-(3-phenylpropyl)methanesulfonamide
190
180
170
160
150
140
130
120
110
100
90
80
70
60
50
40
30
20
10
ppm

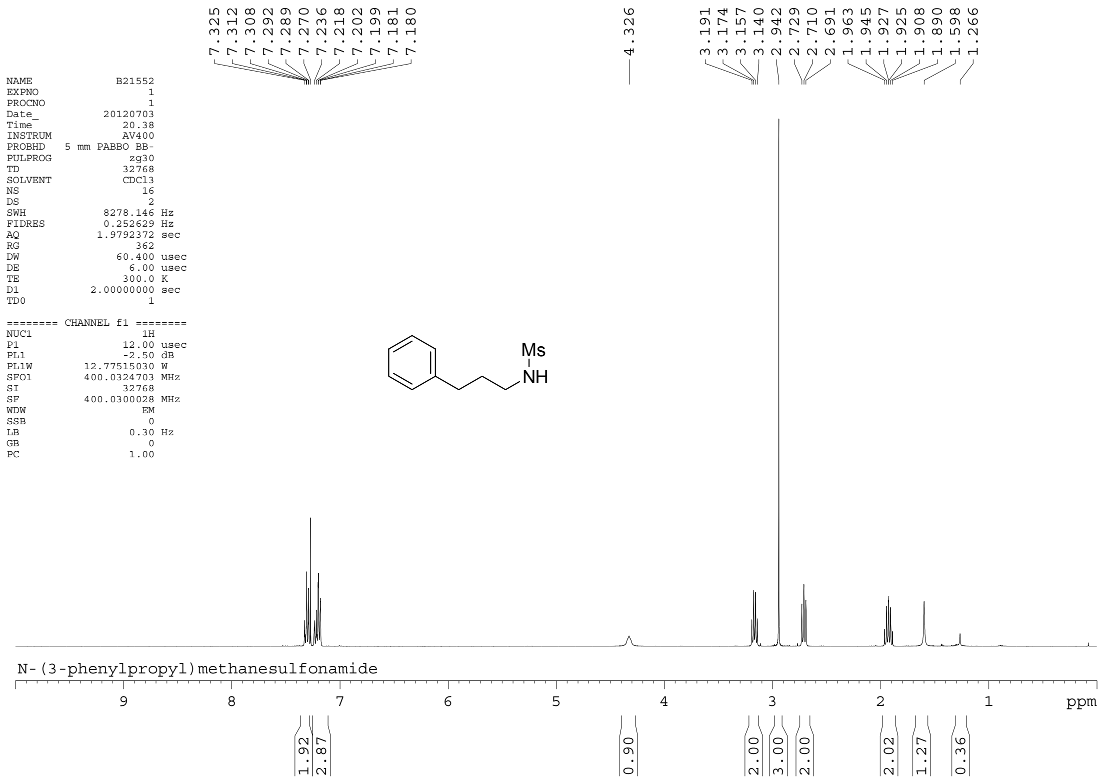
7.325 7.312 7.308 7.292 7.289 7.270 7.236 7.218 7.202 7.199 7.181 7.180
4.326
3.191 3.174 3.157 3.140 2.942 2.729 2.710 2.691 1.963 1.945 1.927 1.925 1.908 1.890 1.598 1.266
NAME B21552
SOLVENT CDCl3
Ms
NH
N-(3-phenylpropyl)methanesulfonamide
ppm
1.92 2.87 0.90 2.00 3.00 2.00 2.02 1.27 0.36

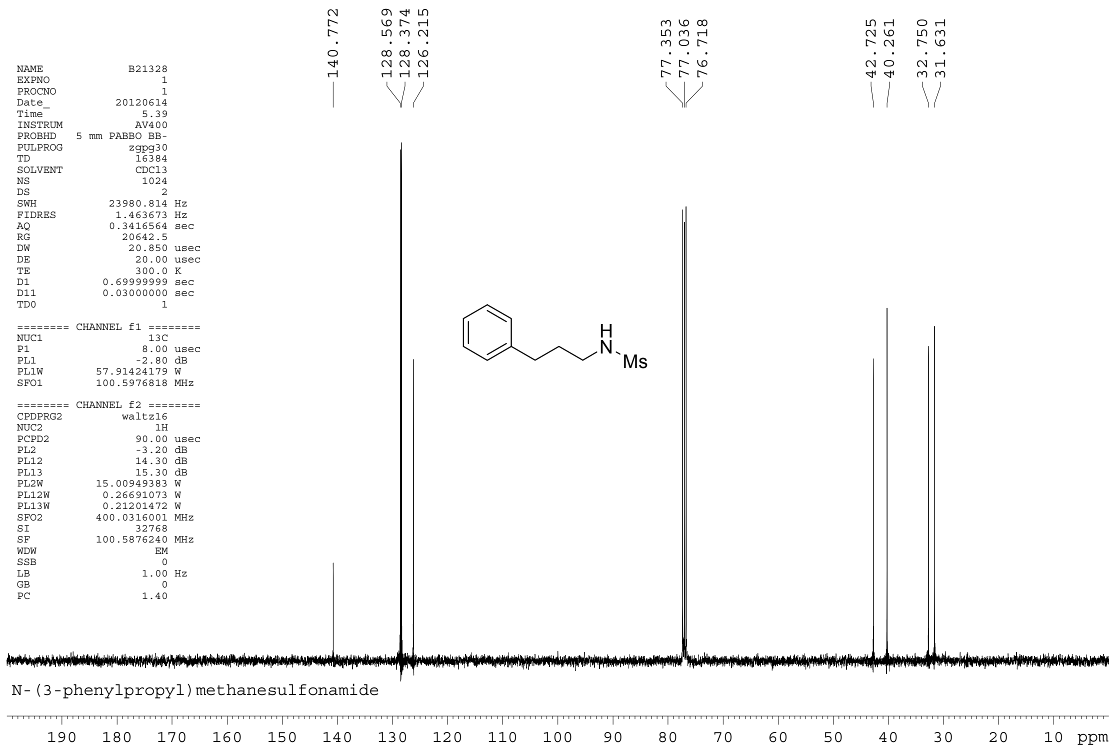
140.772
128.569 128.374 126.215
77.353 77.036 76.718
42.725 40.261 32.750 31.631
NAME B21328
SOLVENT CDCl3
H
N
Ms
N-(3-phenylpropyl)methanesulfonamide
ppm

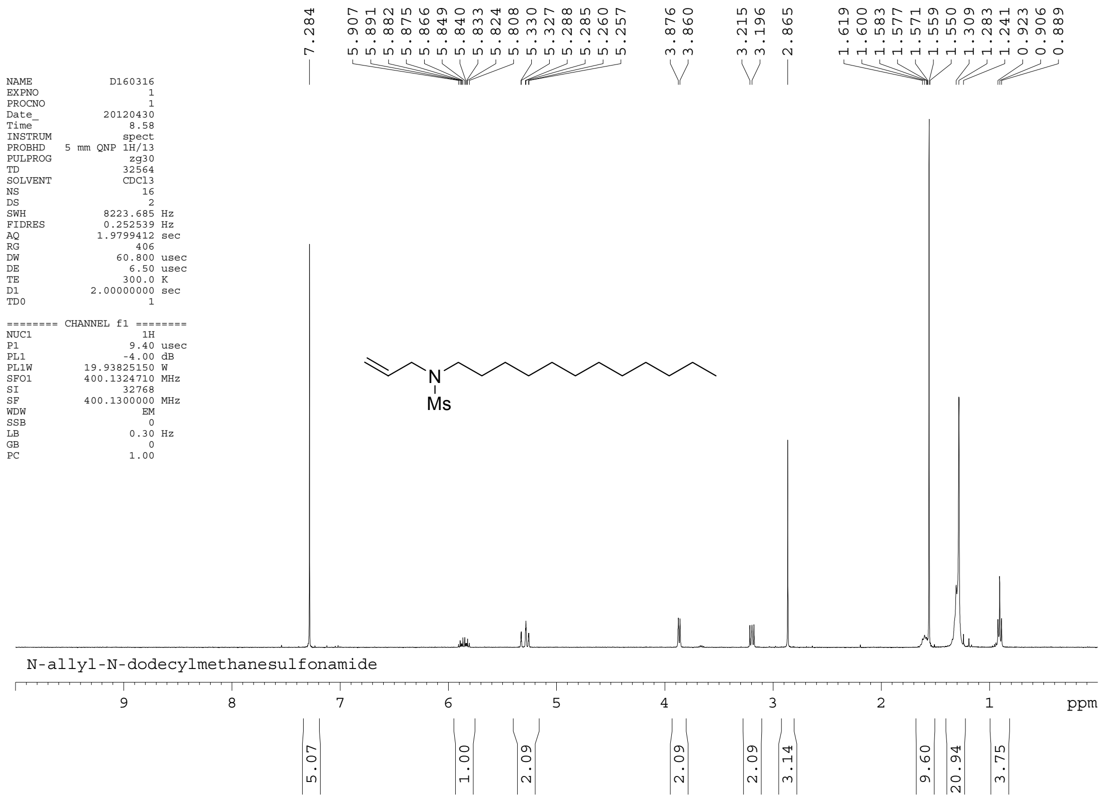
NAME D160316
EXPNO 1
PROCNO 1
Date_ 20120430
Time 8.58
INSTRUM spect
PROBHD 5 mm QNP 1H/13
PULPROG zg30
TD 32564
SOLVENT CDCl3
NS 16
DS 2
SWH 8223.685 Hz
FIDRES 0.252539 Hz
AQ 1.9799412 sec
RG 406
DW 60.800 usec
DE 6.50 usec
TE 300.0 K
D1 2.00000000 sec
TD0 1
======== CHANNEL f1 ========
NUC1 1H
P1 9.40 usec
PL1 -4.00 dB
PL1W 19.93825150 W
SFO1 400.1324710 MHz
SI 32768
SF 400.1300000 MHz
WDW EM
SSB 0
LB 0.30 Hz
GB 0
PC 1.00
7.284
5.907
5.891
5.882
5.875
5.866
5.849
5.840
5.833
5.824
5.808
5.330
5.327
5.288
5.285
5.260
5.257
3.876
3.860
3.215
3.196
2.865
1.619
1.600
1.583
1.577
1.571
1.559
1.550
1.309
1.283
1.241
0.923
0.906
0.889
N
Ms
N-allyl-N-dodecylmethanesulfonamide
9
8
7
6
5
4
3
2
1
ppm
5.07
1.00
2.09
2.09
2.09
3.14
9.60
20.94
3.75

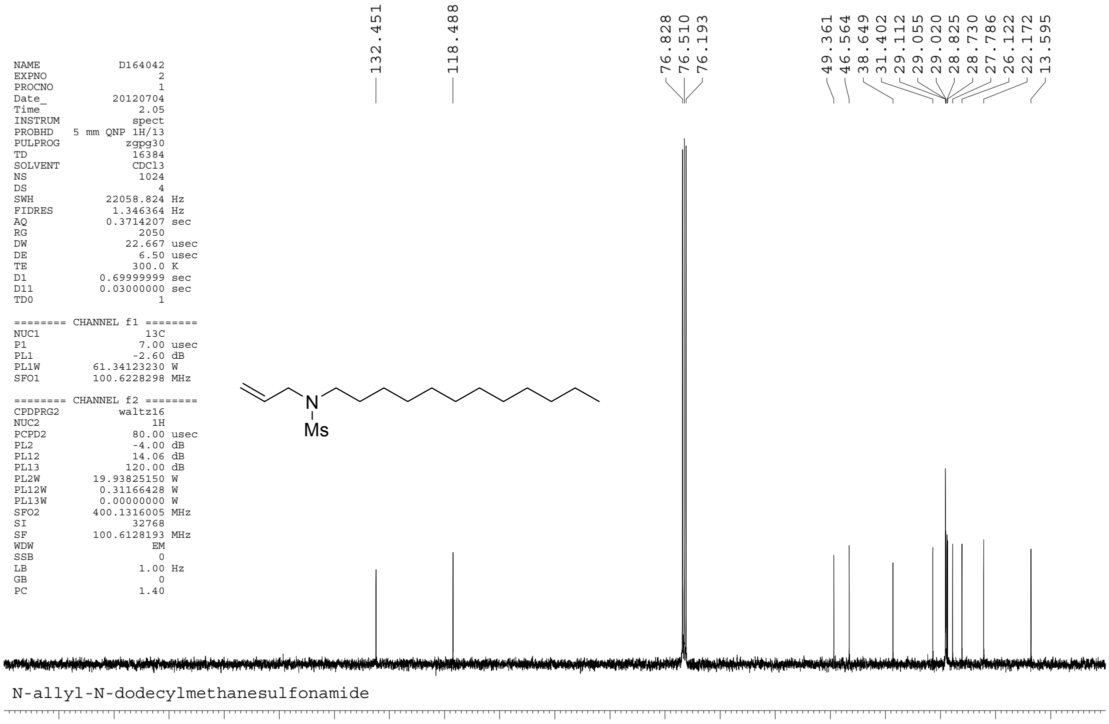
NAME D164042
EXPNO 2
PROCNO 1
Date_ 20120704
Time 2.05
INSTRUM spect
PROBHD 5 mm QNP 1H/13
PULPROG zgpg30
TD 16384
SOLVENT CDCl3
NS 1024
DS 4
SWH 22058.824 Hz
FIDRES 1.346364 Hz
AQ 0.3714207 sec
RG 2050
DW 22.667 usec
DE 6.50 usec
TE 300.0 K
D1 0.69999999 sec
D11 0.03000000 sec
TD0 1
======== CHANNEL f1 ========
NUC1 13C
P1 7.00 usec
PL1 -2.60 dB
PL1W 61.34123230 W
SFO1 100.6228298 MHz
======== CHANNEL f2 ========
CPDPRG2 waltz16
NUC2 1H
PCPD2 80.00 usec
PL2 -4.00 dB
PL12 14.06 dB
PL13 120.00 dB
PL2W 19.93825150 W
PL12W 0.31166428 W
PL13W 0.00000000 W
SFO2 400.1316005 MHz
SI 32768
SF 100.6128193 MHz
WDW EM
SSB 0
LB 1.00 Hz
GB 0
PC 1.40
132.451
118.488
76.828
76.510
76.193
49.361
46.564
38.649
31.402
29.112
29.055
29.020
28.825
28.730
27.786
26.122
22.172
13.595
N
Ms
N-allyl-N-dodecylmethanesulfonamide
190
180
170
160
150
140
130
120
110
100
90
80
70
60
50
40
30
20
10
ppm

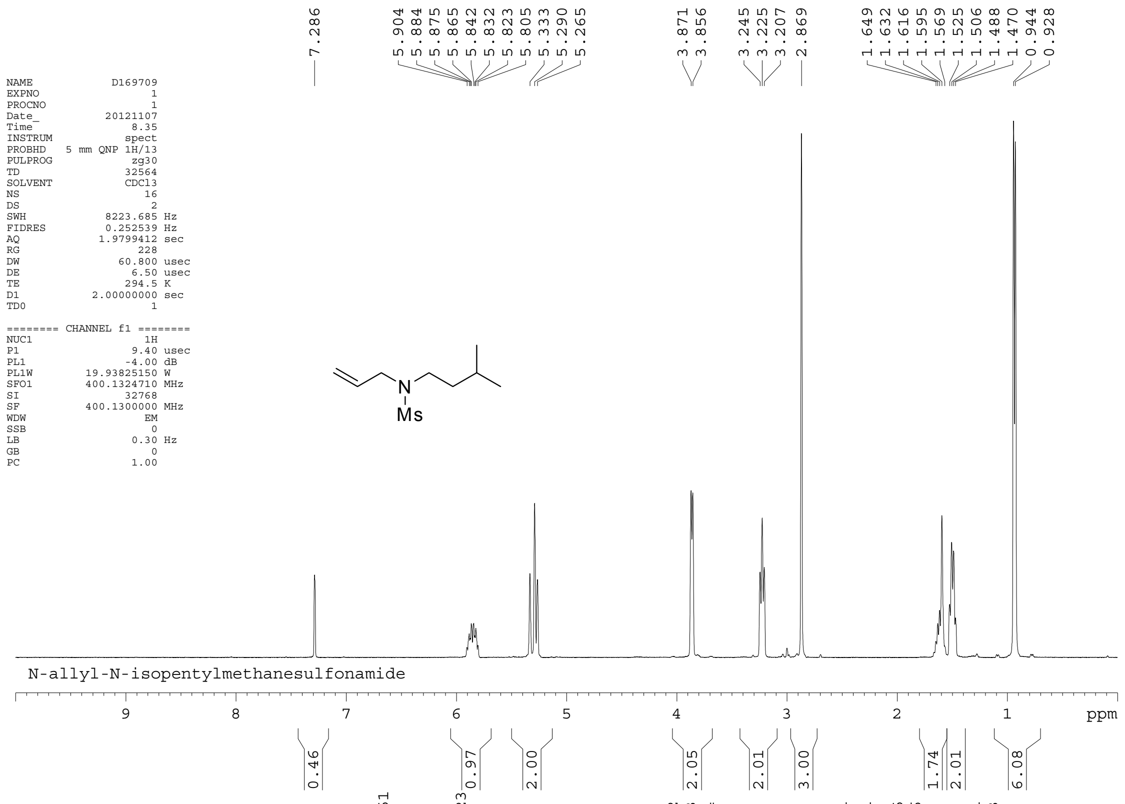
7.286
5.904
5.884
5.875
5.865
5.842
5.832
5.823
5.805
5.333
5.290
5.265
3.871
3.856
3.245
3.225
3.207
2.869
1.649
1.632
1.616
1.595
1.569
1.525
1.506
1.488
1.470
0.944
0.928
NAME D169709
EXPNO 1
PROCNO 1
Date_ 20121107
Time 8.35
INSTRUM spect
PROBHD 5 mm QNP 1H/13
PULPROG zg30
TD 32564
SOLVENT CDCl3
NS 16
DS 2
SWH 8223.685 Hz
FIDRES 0.252539 Hz
AQ 1.9799412 sec
RG 228
DW 60.800 usec
DE 6.50 usec
TE 294.5 K
D1 2.00000000 sec
TD0 1
======== CHANNEL f1 ========
NUC1 1H
P1 9.40 usec
PL1 -4.00 dB
PL1W 19.93825150 W
SFO1 400.1324710 MHz
SI 32768
SF 400.1300000 MHz
WDW EM
SSB 0
LB 0.30 Hz
GB 0
PC 1.00
N
Ms
N-allyl-N-isopentylmethanesulfonamide
9
8
7
6
5
4
3
2
1
ppm
0.46
0.97
2.00
2.05
2.01
3.00
1.74
2.01
6.08

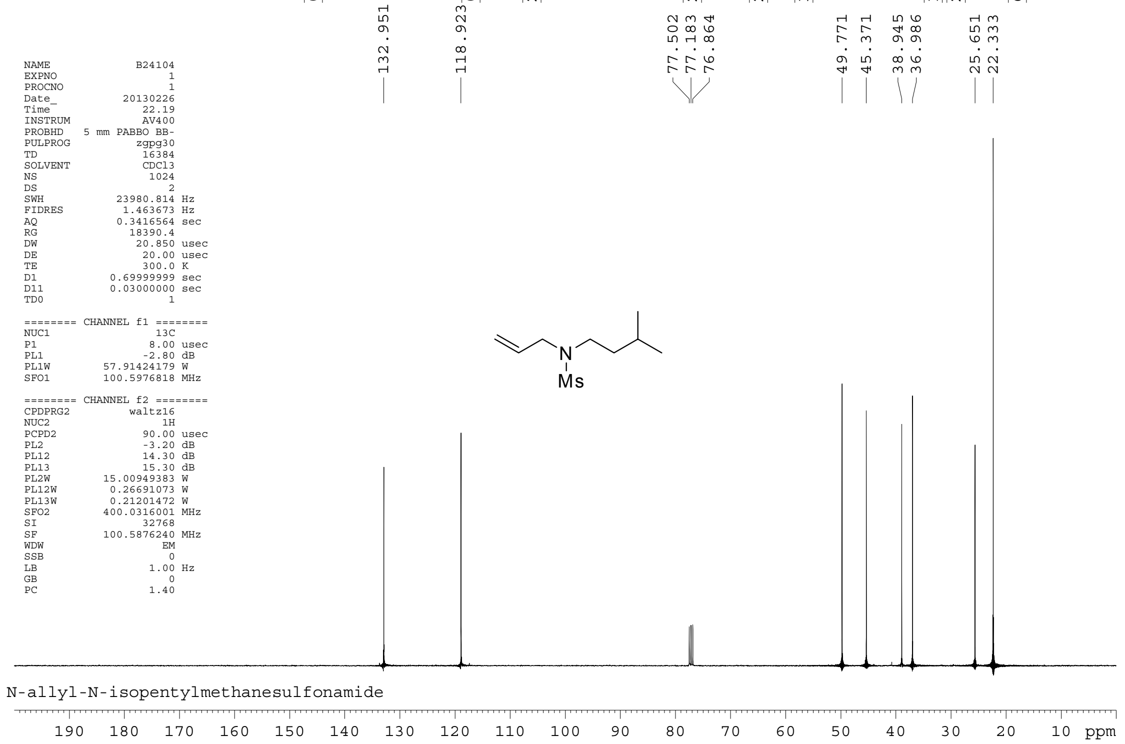
132.951
118.923
77.502
77.183
76.864
49.771
45.371
38.945
36.986
25.651
22.333
NAME B24104
EXPNO 1
PROCNO 1
Date_ 20130226
Time 22.19
INSTRUM AV400
PROBHD 5 mm PABBO BB-
PULPROG zgpg30
TD 16384
SOLVENT CDCl3
NS 1024
DS 2
SWH 23980.814 Hz
FIDRES 1.463673 Hz
AQ 0.3416564 sec
RG 18390.4
DW 20.850 usec
DE 20.00 usec
TE 300.0 K
D1 0.69999999 sec
D11 0.03000000 sec
TD0 1
======== CHANNEL f1 ========
NUC1 13C
P1 8.00 usec
PL1 -2.80 dB
PL1W 57.91424179 W
SFO1 100.5976818 MHz
======== CHANNEL f2 ========
CPDPRG2 waltz16
NUC2 1H
PCPD2 90.00 usec
PL2 -3.20 dB
PL12 14.30 dB
PL13 15.30 dB
PL2W 15.00949383 W
PL12W 0.26691073 W
PL13W 0.21201472 W
SFO2 400.0316001 MHz
SI 32768
SF 100.5876240 MHz
WDW EM
SSB 0
LB 1.00 Hz
GB 0
PC 1.40
N
Ms
N-allyl-N-isopentylmethanesulfonamide
190
180
170
160
150
140
130
120
110
100
90
80
70
60
50
40
30
20
10
ppm

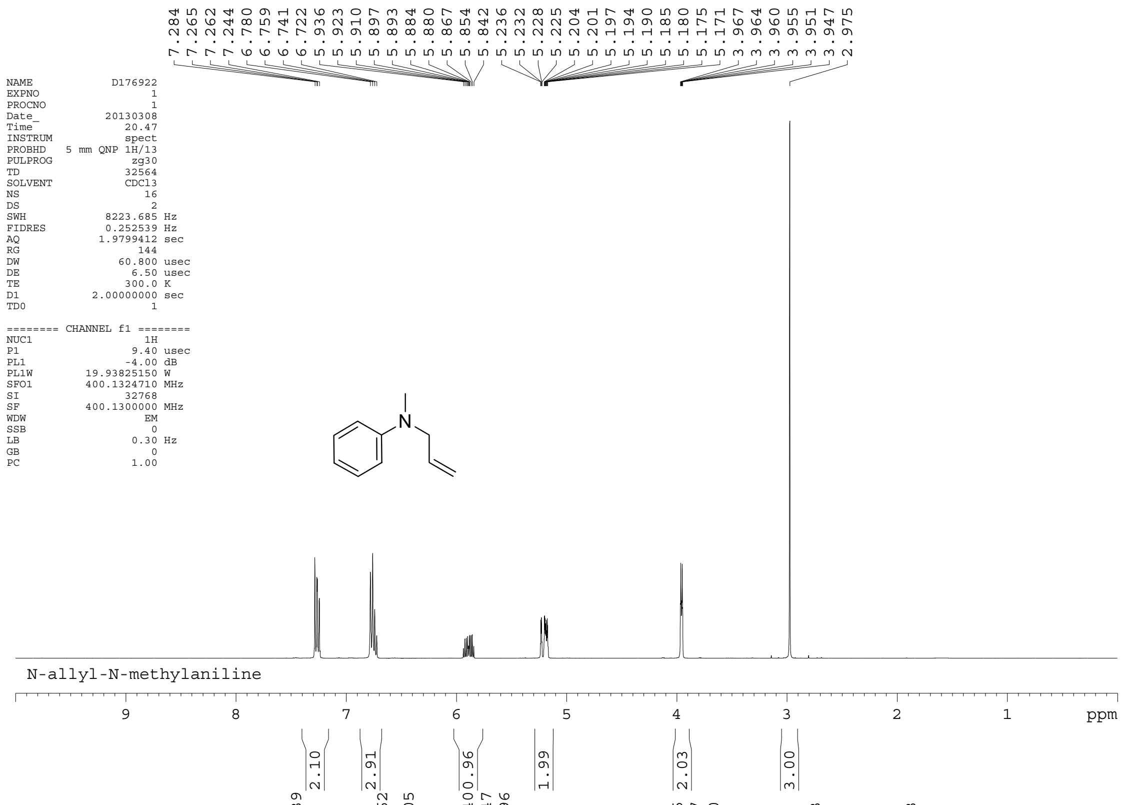
7.284 7.265 7.262 7.244 6.780 6.759 6.741 6.722 5.936 5.923 5.910 5.897 5.893 5.884 5.880 5.867 5.854 5.842 5.236 5.232 5.228 5.225 5.204 5.201 5.197 5.194 5.190 5.185 5.180 5.175 5.171 3.967 3.964 3.960 3.955 3.951 3.947 2.975
NAME D176922
EXPNO 1
PROCNO 1
Date_ 20130308
Time 20.47
INSTRUM spect
PROBHD 5 mm QNP 1H/13
PULPROG zg30
TD 32564
SOLVENT CDCl3
NS 16
DS 2
SWH 8223.685 Hz
FIDRES 0.252539 Hz
AQ 1.9799412 sec
RG 144
DW 60.800 usec
DE 6.50 usec
TE 300.0 K
D1 2.00000000 sec
TD0 1
======== CHANNEL f1 ========
NUC1 1H
P1 9.40 usec
PL1 -4.00 dB
PL1W 19.93825150 W
SFO1 400.1324710 MHz
SI 32768
SF 400.1300000 MHz
WDW EM
SSB 0
LB 0.30 Hz
GB 0
PC 1.00
N
N-allyl-N-methylaniline
9 8 7 6 5 4 3 2 1 ppm
2.10 2.91 0.96 1.99 2.03 3.00

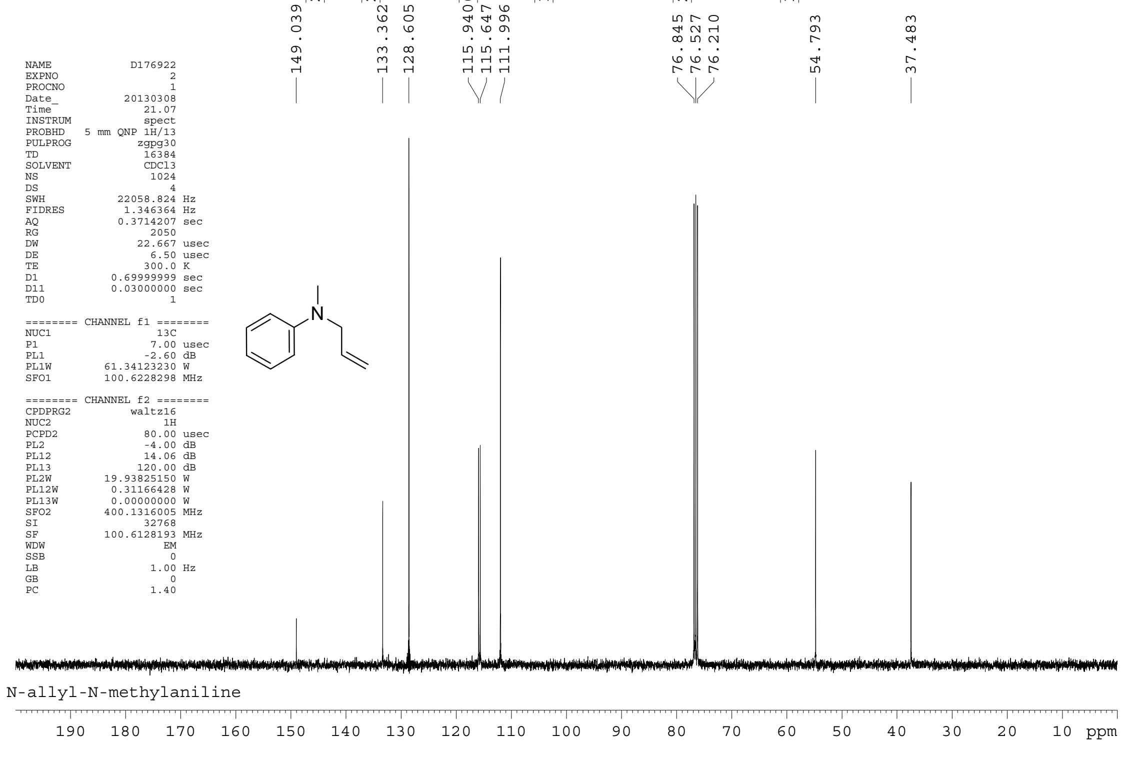
149.039 133.362 128.605 115.940 115.647 111.996 76.845 76.527 76.210 54.793 37.483
NAME D176922
EXPNO 2
PROCNO 1
Date_ 20130308
Time 21.07
INSTRUM spect
PROBHD 5 mm QNP 1H/13
PULPROG zgpg30
TD 16384
SOLVENT CDCl3
NS 1024
DS 4
SWH 22058.824 Hz
FIDRES 1.346364 Hz
AQ 0.3714207 sec
RG 2050
DW 22.667 usec
DE 6.50 usec
TE 300.0 K
D1 0.69999999 sec
D11 0.03000000 sec
TD0 1
======== CHANNEL f1 ========
NUC1 13C
P1 7.00 usec
PL1 -2.60 dB
PL1W 61.3412230 W
SFO1 100.6228298 MHz
======== CHANNEL f2 ========
CPDPRG2 waltz16
NUC2 1H
PCPD2 80.00 usec
PL2 -4.00 dB
PL12 14.06 dB
PL13 120.00 dB
PL2W 19.93825150 W
PL12W 0.31166428 W
PL13W 0.00000000 W
SFO2 400.1316005 MHz
SI 32768
SF 100.6128193 MHz
WDW EM
SSB 0
LB 1.00 Hz
GB 0
PC 1.40
N
N-allyl-N-methylaniline
190 180 170 160 150 140 130 120 110 100 90 80 70 60 50 40 30 20 10 ppm

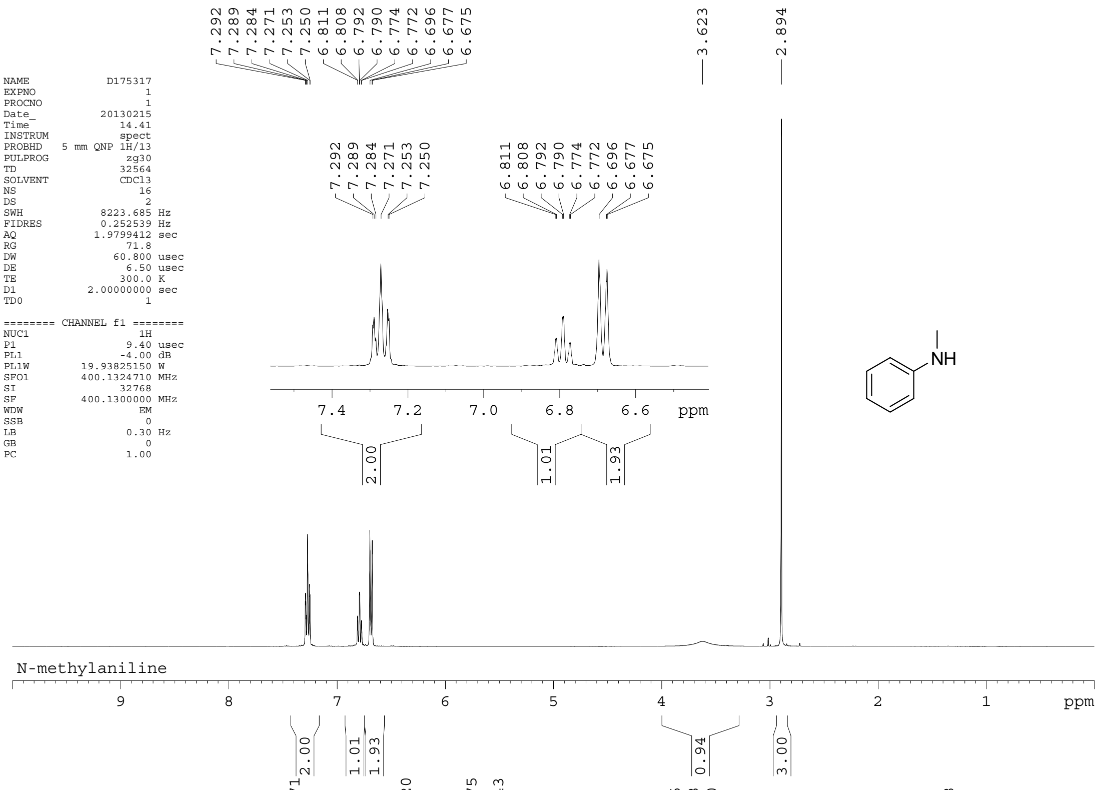
7.292 7.289 7.284 7.271 7.253 7.250 6.811 6.808 6.792 6.790 6.774 6.772 6.696 6.677 6.675
3.623
2.894
NAME D175317
EXPNO 1
PROCNO 1
Date_ 20130215
Time 14.41
INSTRUM spect
PROBHD 5 mm QNP 1H/13
PULPROG zg30
TD 32564
SOLVENT CDCl3
NS 16
DS 2
SWH 8223.685 Hz
FIDRES 0.252539 Hz
AQ 1.9799412 sec
RG 71.8
DW 60.800 usec
DE 6.50 usec
TE 300.0 K
D1 2.00000000 sec
TD0 1
======== CHANNEL f1 ========
NUC1 1H
P1 9.40 usec
PL1 -4.00 dB
PL1W 19.93825150 W
SFO1 400.1324710 MHz
SI 32768
SF 400.1300000 MHz
WDW EM
SSB 0
LB 0.30 Hz
GB 0
PC 1.00
7.4 7.2 7.0 6.8 6.6 ppm
2.00 1.01 1.93
NH
N-methylaniline
9 8 7 6 5 4 3 2 1 ppm
2.00 1.01 1.93 0.94 3.00

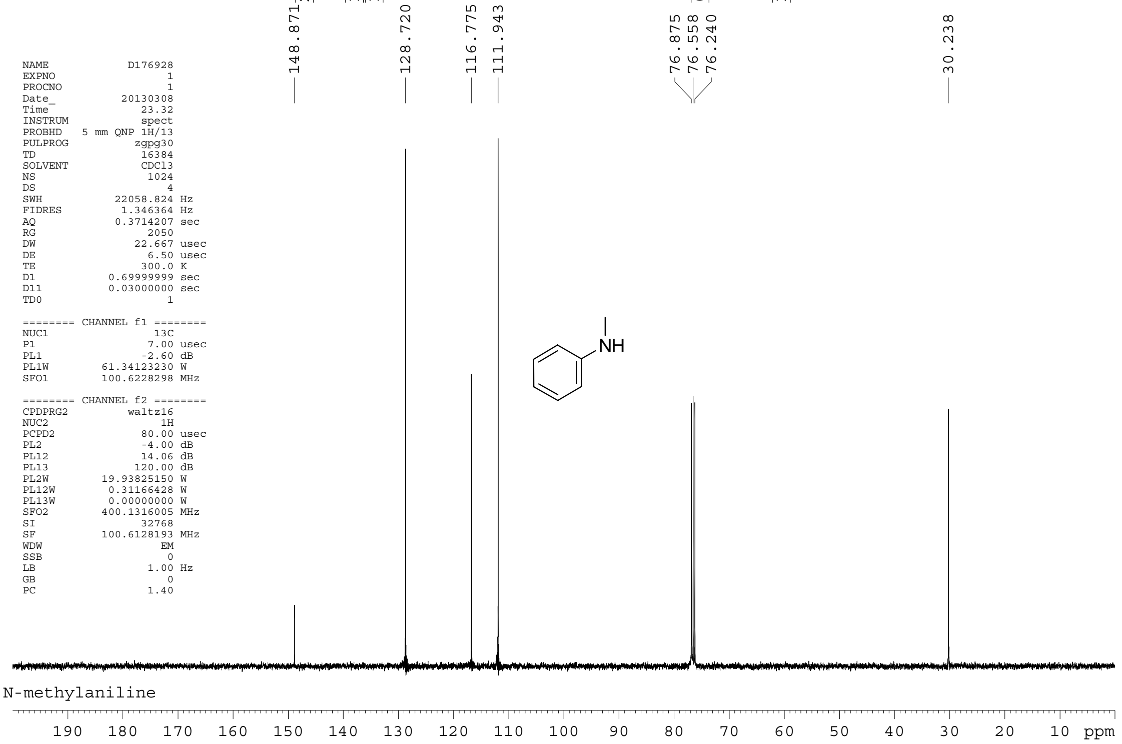
148.871
128.720
116.775
111.943
76.875 76.558 76.240
30.238
NAME D176928
EXPNO 1
PROCNO 1
Date_ 20130308
Time 23.32
INSTRUM spect
PROBHD 5 mm QNP 1H/13
PULPROG zgpg30
TD 16384
SOLVENT CDCl3
NS 1024
DS 4
SWH 22058.824 Hz
FIDRES 1.346364 Hz
AQ 0.3714207 sec
RG 2050
DW 22.667 usec
DE 6.50 usec
TE 300.0 K
D1 0.69999999 sec
D11 0.03000000 sec
TD0 1
======== CHANNEL f1 ========
NUC1 13C
P1 7.00 usec
PL1 -2.60 dB
PL1W 61.34123230 W
SFO1 100.6228298 MHz
======== CHANNEL f2 ========
CPDPRG2 waltz16
NUC2 1H
PCPD2 80.00 usec
PL2 -4.00 dB
PL12 14.06 dB
PL13 120.00 dB
PL2W 19.93825150 W
PL12W 0.31166428 W
PL13W 0.00000000 W
SFO2 400.1316005 MHz
SI 32768
SF 100.6128193 MHz
WDW EM
SSB 0
LB 1.00 Hz
GB 0
PC 1.40
NH
N-methylaniline
190 180 170 160 150 140 130 120 110 100 90 80 70 60 50 40 30 20 10 ppm

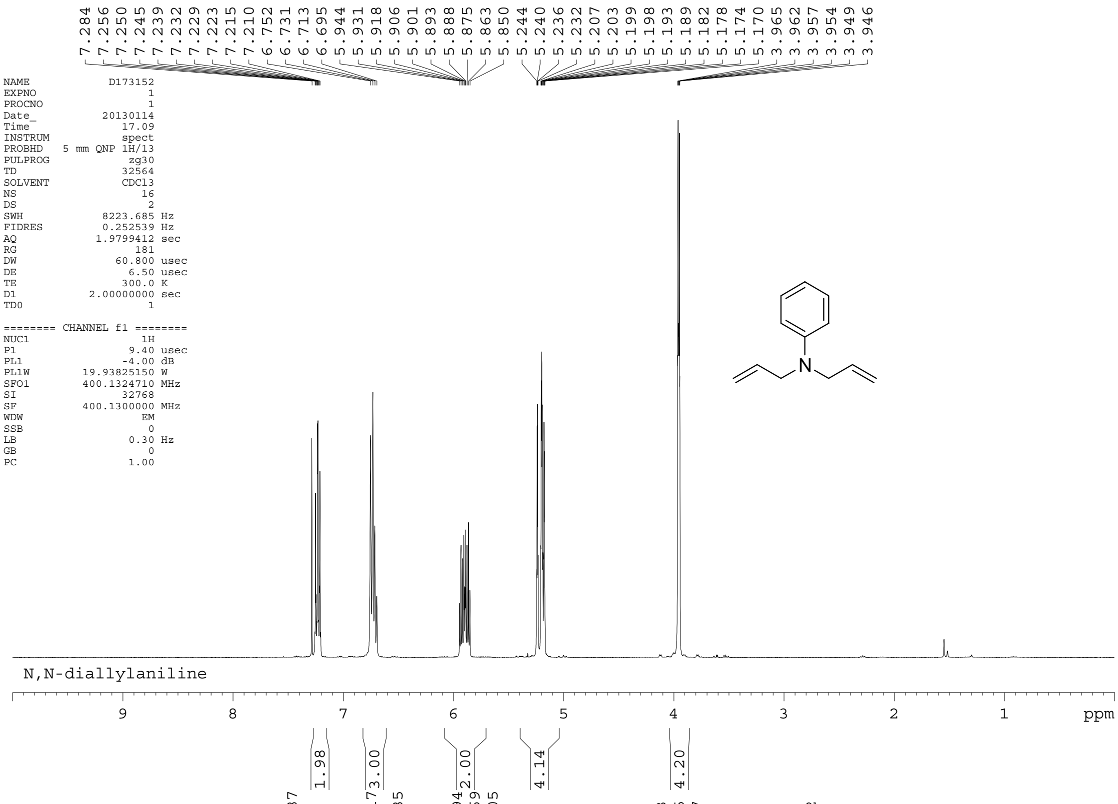
7.284
7.256
7.250
7.245
7.239
7.232
7.229
7.223
7.215
7.210
6.752
6.731
6.713
6.695
5.944
5.931
5.918
5.906
5.901
5.893
5.888
5.875
5.863
5.850
5.244
5.240
5.236
5.232
5.207
5.203
5.199
5.198
5.193
5.189
5.182
5.178
5.174
5.170
3.965
3.962
3.957
3.954
3.949
3.946
NAME D173152
EXPNO 1
PROCNO 1
Date_ 20130114
Time 17.09
INSTRUM spect
PROBHD 5 mm QNP 1H/13
PULPROG zg30
TD 32564
SOLVENT CDCl3
NS 16
DS 2
SWH 8223.685 Hz
FIDRES 0.252539 Hz
AQ 1.9799412 sec
RG 181
DW 60.800 usec
DE 6.50 usec
TE 300.0 K
D1 2.00000000 sec
TD0 1
======== CHANNEL f1 ========
NUC1 1H
P1 9.40 usec
PL1 -4.00 dB
PL1W 19.93825150 W
SFO1 400.1324710 MHz
SI 32768
SF 400.1300000 MHz
WDW EM
SSB 0
LB 0.30 Hz
GB 0
PC 1.00
N
N,N-diallylaniline
9
8
7
6
5
4
3
2
1
ppm
1.98
3.00
2.00
4.14
4.20

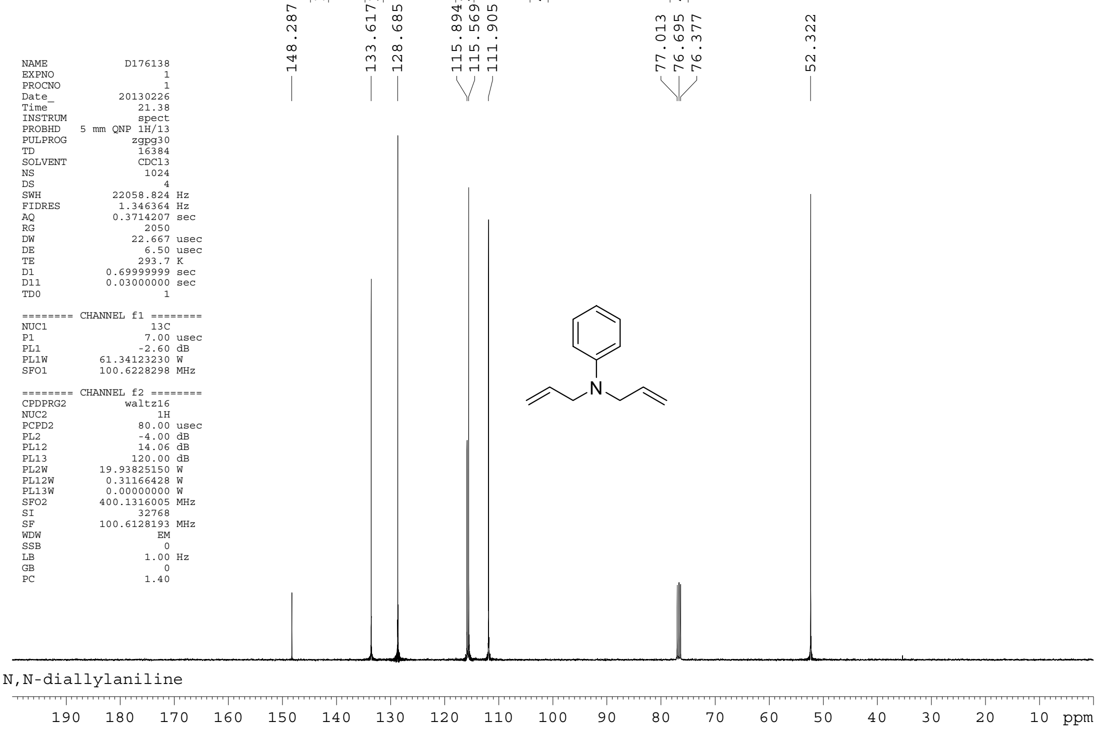
148.287
133.617
128.685
115.894
115.569
111.905
77.013
76.695
76.377
52.322
NAME D176138
EXPNO 1
PROCNO 1
Date_ 20130226
Time 21.38
INSTRUM spect
PROBHD 5 mm QNP 1H/13
PULPROG zgpg30
TD 16384
SOLVENT CDCl3
NS 1024
DS 4
SWH 22058.824 Hz
FIDRES 1.346364 Hz
AQ 0.3714207 sec
RG 2050
DW 22.667 usec
DE 6.50 usec
TE 293.7 K
D1 0.69999999 sec
D11 0.03000000 sec
TD0 1
======== CHANNEL f1 ========
NUC1 13C
P1 7.00 usec
PL1 -2.60 dB
PL1W 61.34123230 W
SFO1 100.6228298 MHz
======== CHANNEL f2 ========
CPDPRG2 waltz16
NUC2 1H
PCPD2 80.00 usec
PL2 -4.00 dB
PL12 14.06 dB
PL13 120.00 dB
PL2W 19.93825150 W
PL12W 0.31166428 W
PL13W 0.00000000 W
SFO2 400.1316005 MHz
SI 32768
SF 100.6128193 MHz
WDW EM
SSB 0
LB 1.00 Hz
GB 0
PC 1.40
N
N,N-diallylaniline
190
180
170
160
150
140
130
120
110
100
90
80
70
60
50
40
30
20
10
ppm

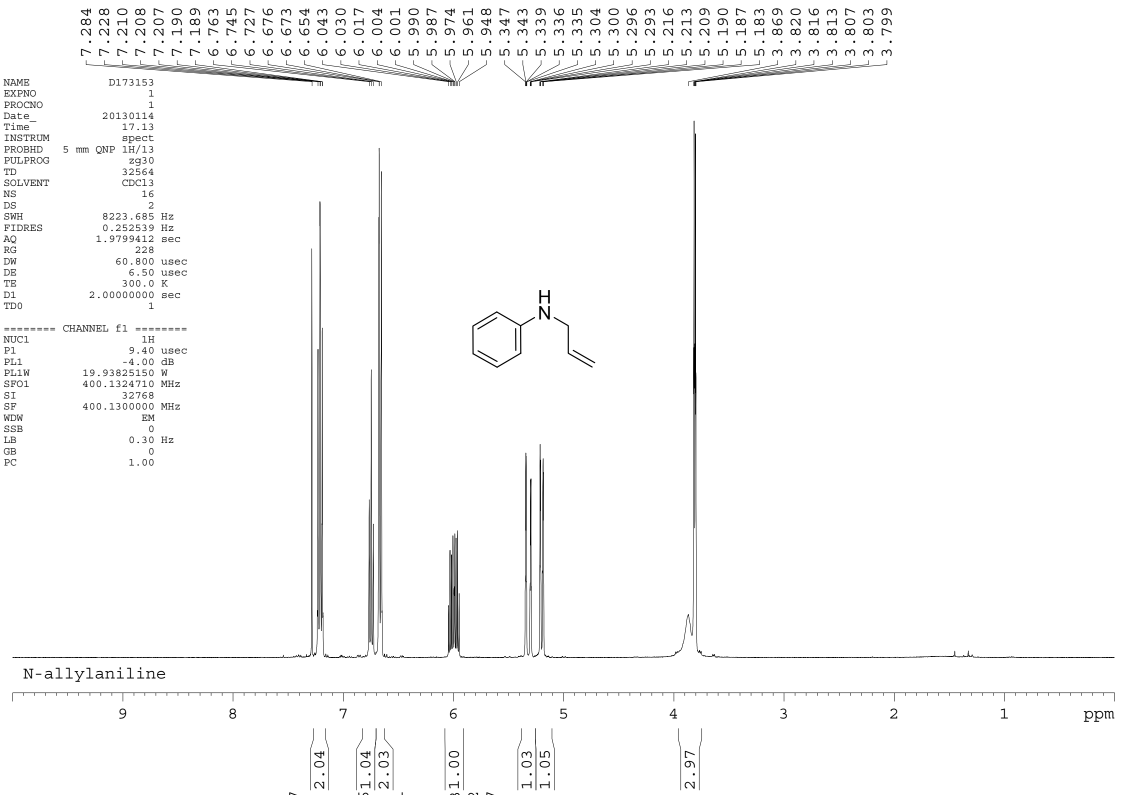
7.284 7.228 7.210 7.208 7.207 7.190 7.189 6.763 6.745 6.727 6.676 6.673 6.654 6.043 6.030 6.017 6.004 6.001 5.990 5.987 5.974 5.961 5.948 5.347 5.343 5.339 5.336 5.335 5.304 5.300 5.296 5.293 5.216 5.213 5.209 5.190 5.187 5.183 3.869 3.820 3.816 3.813 3.807 3.803 3.799
NAME D173153
EXPNO 1
PROCNO 1
Date_ 20130114
Time 17.13
INSTRUM spect
PROBHD 5 mm QNP 1H/13
PULPROG zg30
TD 32564
SOLVENT CDCl3
NS 16
DS 2
SWH 8223.685 Hz
FIDRES 0.252539 Hz
AQ 1.9799412 sec
RG 228
DW 60.800 usec
DE 6.50 usec
TE 300.0 K
D1 2.00000000 sec
TD0 1
======== CHANNEL f1 ========
NUC1 1H
P1 9.40 usec
PL1 -4.00 dB
PL1W 19.93825150 W
SFO1 400.1324710 MHz
SI 32768
SF 400.1300000 MHz
WDW EM
SSB 0
LB 0.30 Hz
GB 0
PC 1.00
H
N
N-allylaniline
9 8 7 6 5 4 3 2 1 ppm
2.04 1.04 2.03 1.00 1.03 1.05 2.97

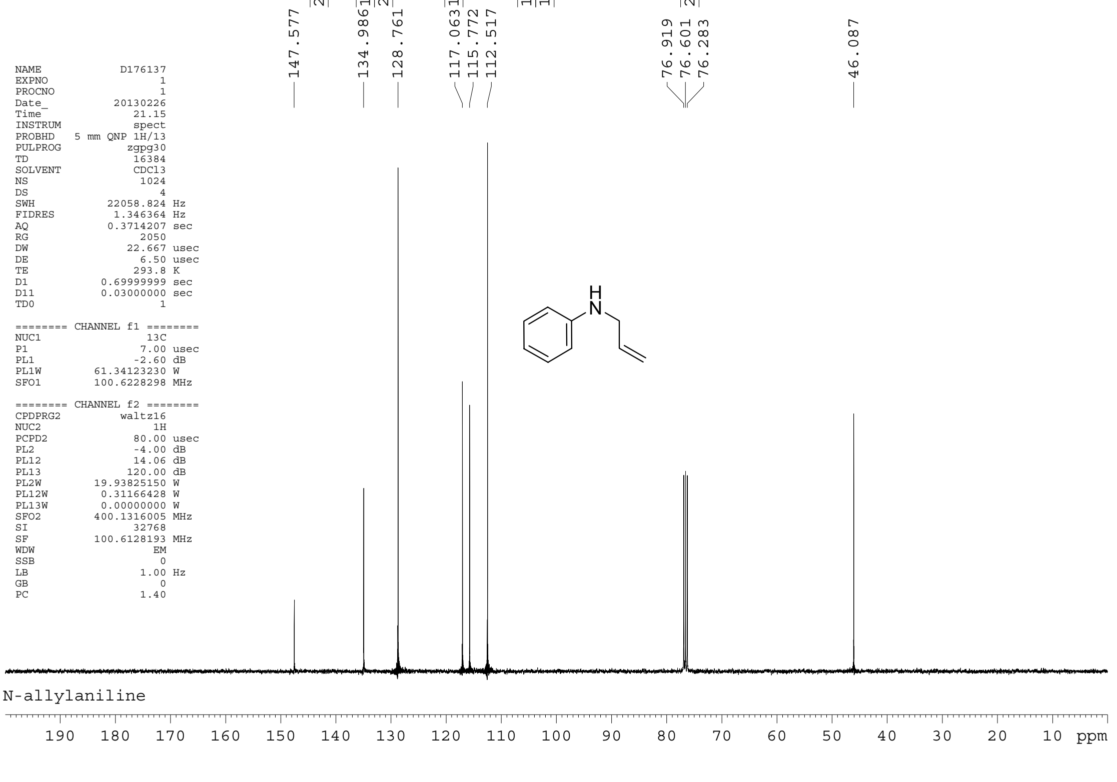
147.577 134.986 128.761 117.063 115.772 112.517 76.919 76.601 76.283 46.087
NAME D176137
EXPNO 1
PROCNO 1
Date_ 20130226
Time 21.15
INSTRUM spect
PROBHD 5 mm QNP 1H/13
PULPROG zgpg30
TD 16384
SOLVENT CDCl3
NS 1024
DS 4
SWH 22058.824 Hz
FIDRES 1.346364 Hz
AQ 0.3714207 sec
RG 2050
DW 22.667 usec
DE 6.50 usec
TE 293.8 K
D1 0.69999999 sec
D11 0.03000000 sec
TD0 1
======== CHANNEL f1 ========
NUC1 13C
P1 7.00 usec
PL1 -2.60 dB
PL1W 61.3412323 W
SFO1 100.6228298 MHz
======== CHANNEL f2 ========
CPDPRG2 waltz16
NUC2 1H
PCPD2 80.00 usec
PL2 -4.00 dB
PL12 14.06 dB
PL13 120.00 dB
PL2W 19.93825150 W
PL12W 0.31166428 W
PL13W 0.00000000 W
SFO2 400.1316005 MHz
SI 32768
SF 100.6128193 MHz
WDW EM
SSB 0
LB 1.00 Hz
GB 0
PC 1.40
H
N
N-allylaniline
190 180 170 160 150 140 130 120 110 100 90 80 70 60 50 40 30 20 10 ppm

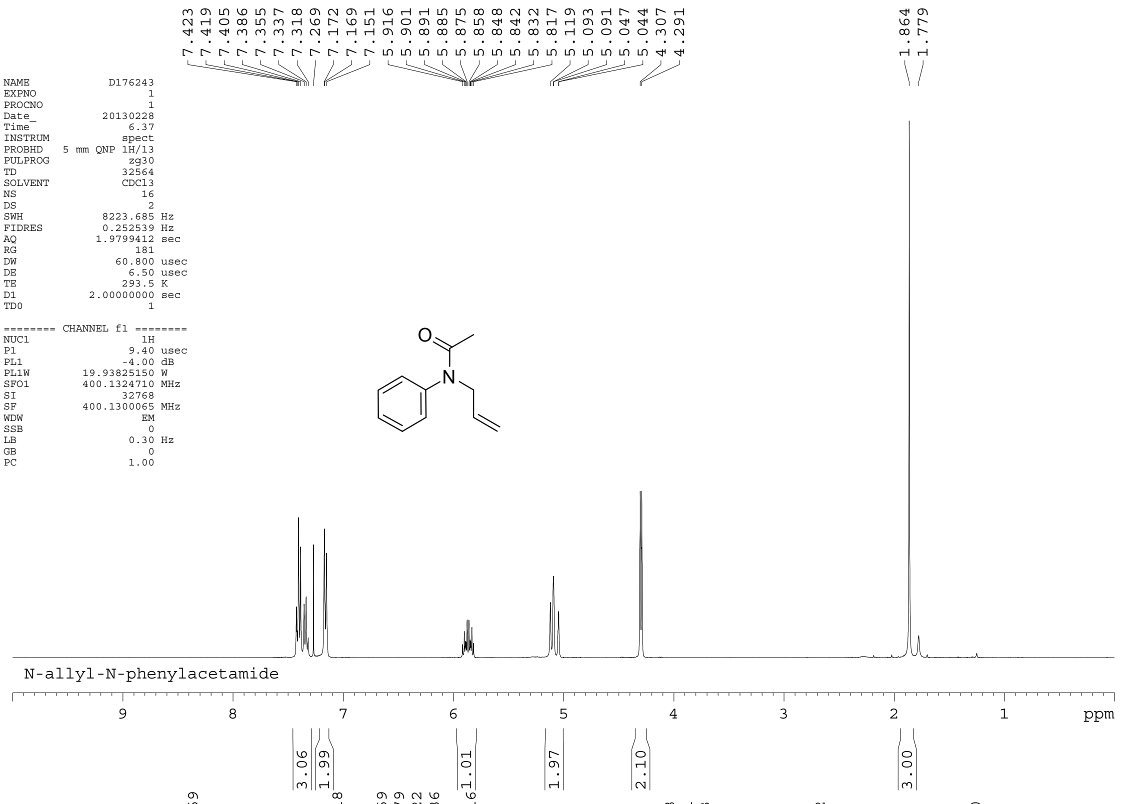
NAME D176243
EXPNO 1
PROCNO 1
Date_ 20130228
Time 6.37
INSTRUM spect
PROBHD 5 mm QNP 1H/13
PULPROG zg30
TD 32564
SOLVENT CDCl3
NS 16
DS 2
SWH 8223.685 Hz
FIDRES 0.252539 Hz
AQ 1.9799412 sec
RG 181
DW 60.800 usec
DE 6.50 usec
TE 293.5 K
D1 2.00000000 sec
TD0 1
======== CHANNEL f1 ========
NUC1 1H
P1 9.40 usec
PL1 -4.00 dB
PL1W 19.93825150 W
SFO1 400.1324710 MHz
SI 32768
SF 400.1300065 MHz
WDW EM
SSB 0
LB 0.30 Hz
GB 0
PC 1.00
7.423 7.419 7.405 7.386 7.355 7.337 7.318 7.269 7.172 7.169 7.151 5.916 5.901 5.891 5.885 5.875 5.858 5.848 5.842 5.832 5.817 5.119 5.093 5.091 5.047 5.044 4.307 4.291 1.864 1.779
O
N
N-allyl-N-phenylacetamide
9 8 7 6 5 4 3 2 1 ppm
3.06 1.99 1.01 1.97 2.10 3.00

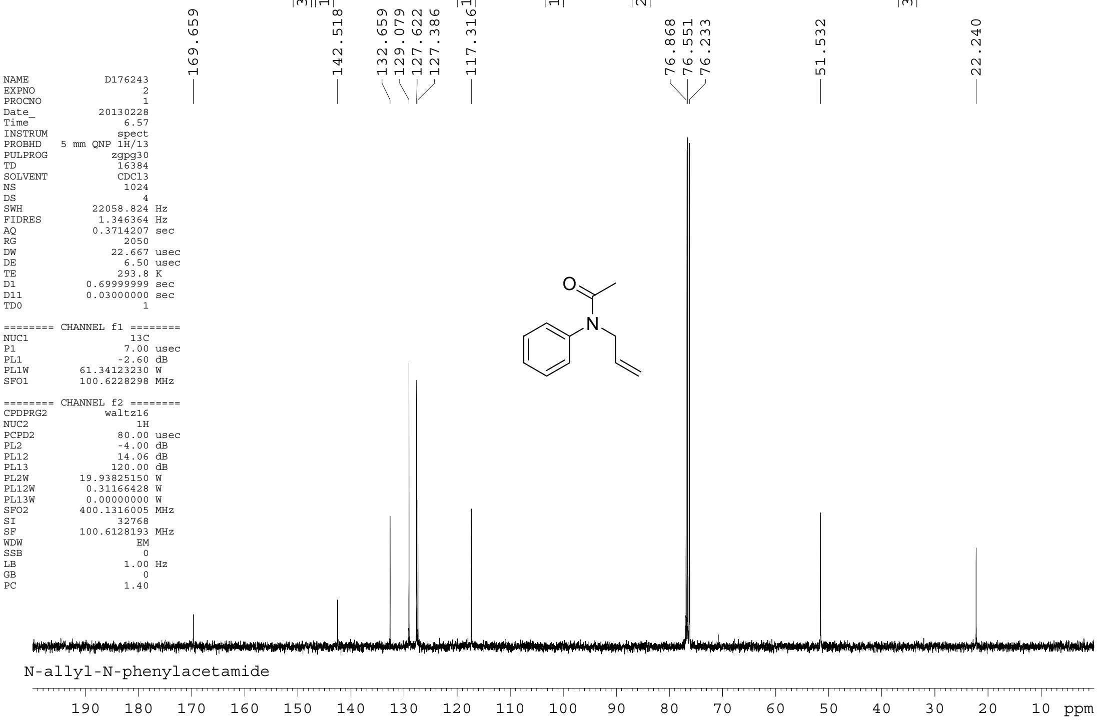
NAME D176243
EXPNO 2
PROCNO 1
Date_ 20130228
Time 6.57
INSTRUM spect
PROBHD 5 mm QNP 1H/13
PULPROG zgpg30
TD 16384
SOLVENT CDCl3
NS 1024
DS 4
SWH 22058.824 Hz
FIDRES 1.346364 Hz
AQ 0.3714207 sec
RG 2050
DW 22.667 usec
DE 6.50 usec
TE 293.8 K
D1 0.69999999 sec
D11 0.03000000 sec
TD0 1
======== CHANNEL f1 ========
NUC1 13C
P1 7.00 usec
PL1 -2.60 dB
PL1W 61.34123230 W
SFO1 100.6228298 MHz
======== CHANNEL f2 ========
CPDPRG2 waltz16
NUC2 1H
PCPD2 80.00 usec
PL2 -4.00 dB
PL12 14.06 dB
PL13 120.00 dB
PL2W 19.93825150 W
PL12W 0.31166428 W
PL13W 0.00000000 W
SFO2 400.1316005 MHz
SI 32768
SF 100.6128193 MHz
WDW EM
SSB 0
LB 1.00 Hz
GB 0
PC 1.40
169.659 142.518 132.659 129.079 127.622 127.386 117.316 76.868 76.551 76.233 51.532 22.240
O
N
N-allyl-N-phenylacetamide
190 180 170 160 150 140 130 120 110 100 90 80 70 60 50 40 30 20 10 ppm

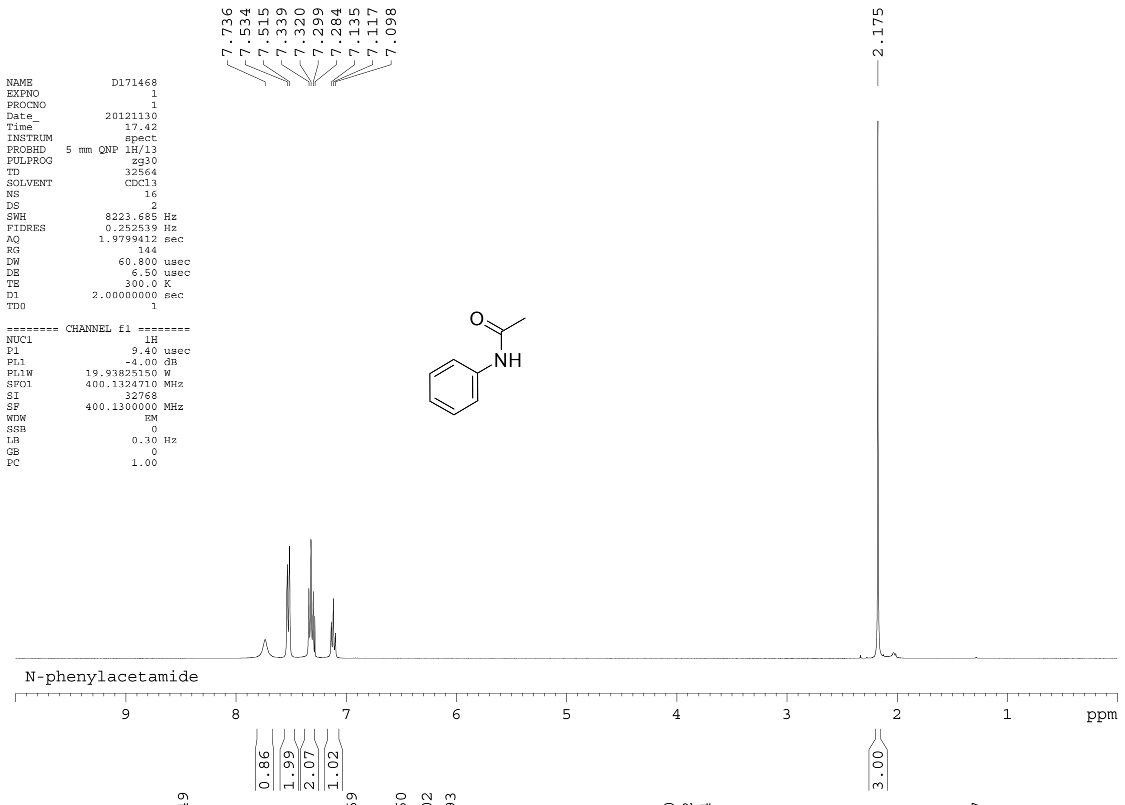

7.736
7.534
7.515
7.339
7.320
7.299
7.284
7.135
7.117
7.098
2.175
NAME D171468
EXPNO 1
PROCNO 1
Date_ 20121130
Time 17.42
INSTRUM spect
PROBHD 5 mm QNP 1H/13
PULPROG zg30
TD 32564
SOLVENT CDCl3
NS 16
DS 2
SWH 8223.685 Hz
FIDRES 0.252539 Hz
AQ 1.9799412 sec
RG 144
DW 60.800 usec
DE 6.50 usec
TE 300.0 K
D1 2.00000000 sec
TD0 1
======== CHANNEL f1 ========
NUC1 1H
P1 9.40 usec
PL1 -4.00 dB
PL1W 19.93825150 W
SFO1 400.1324710 MHz
SI 32768
SF 400.1300000 MHz
WDW EM
SSB 0
LB 0.30 Hz
GB 0
PC 1.00
O
NH
N-phenylacetamide
9 8 7 6 5 4 3 2 1 ppm
0.86
1.99
2.07
1.02
3.00

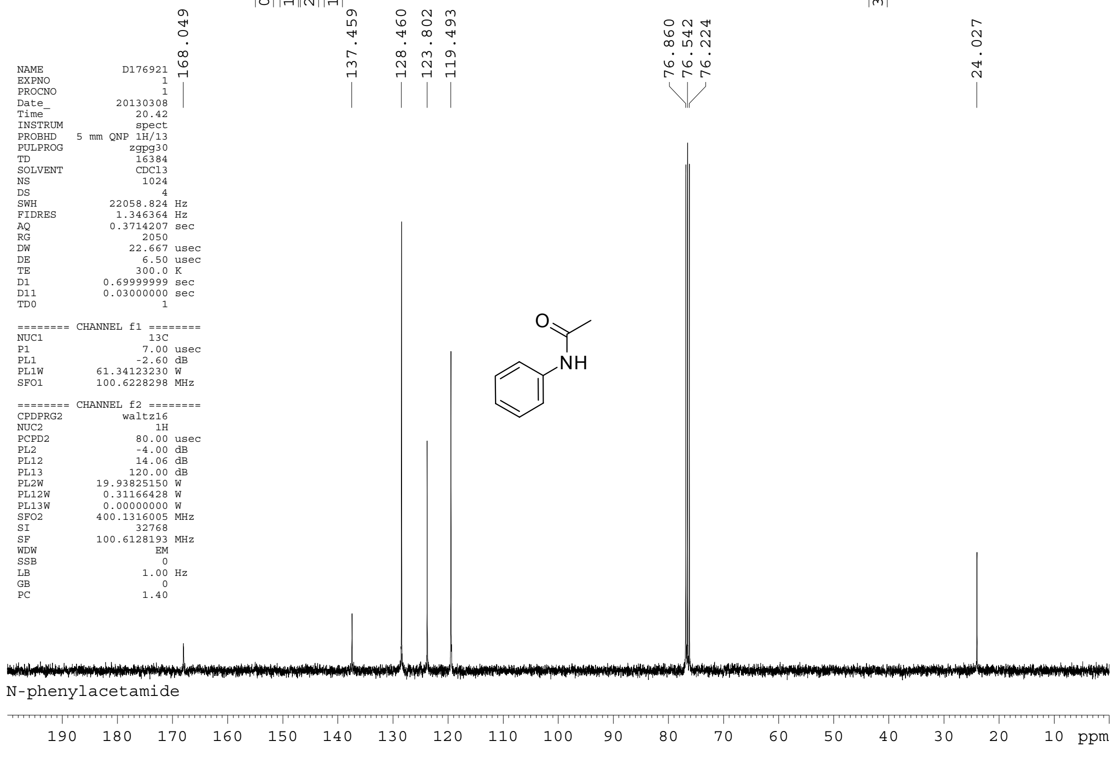

168.049
137.459
128.460
123.802
119.493
76.860
76.542
76.224
24.027
NAME D176921
EXPNO 1
PROCNO 1
Date_ 20130308
Time 20.42
INSTRUM spect
PROBHD 5 mm QNP 1H/13
PULPROG zgpg30
TD 16384
SOLVENT CDCl3
NS 1024
DS 4
SWH 22058.824 Hz
FIDRES 1.346364 Hz
AQ 0.3714207 sec
RG 2050
DW 22.667 usec
DE 6.50 usec
TE 300.0 K
D1 0.69999999 sec
D11 0.03000000 sec
TD0 1
======== CHANNEL f1 ========
NUC1 13C
P1 7.00 usec
PL1 -2.60 dB
PL1W 61.34123230 W
SFO1 100.6228298 MHz
======== CHANNEL f2 ========
CPDPRG2 waltz16
NUC2 1H
PCPD2 80.00 usec
PL2 -4.00 dB
PL12 14.06 dB
PL13 120.00 dB
PL2W 19.93825150 W
PL12W 0.31166428 W
PL13W 0.00000000 W
SFO2 400.1316005 MHz
SI 32768
SF 100.6128193 MHz
WDW EM
SSB 0
LB 1.00 Hz
GB 0
PC 1.40
O
NH
N-phenylacetamide
190 180 170 160 150 140 130 120 110 100 90 80 70 60 50 40 30 20 10 ppm

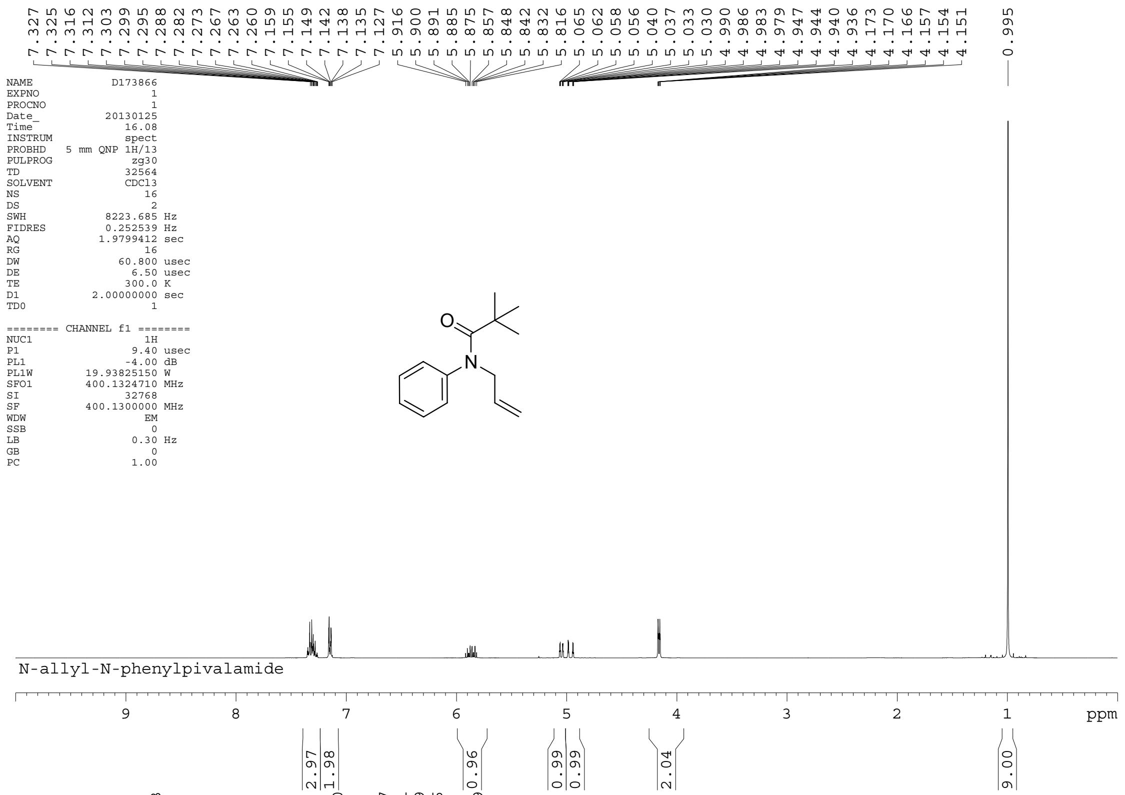
7.327 7.325 7.316 7.312 7.303 7.299 7.295 7.288 7.282 7.273 7.267 7.263 7.260 7.159 7.155 7.149 7.142 7.138 7.135 7.127 5.916 5.900 5.891 5.885 5.875 5.857 5.848 5.842 5.832 5.816 5.065 5.062 5.058 5.056 5.040 5.037 5.033 5.030 4.990 4.986 4.983 4.979 4.947 4.944 4.940 4.936 4.173 4.170 4.166 4.157 4.154 4.151 0.995
NAME D173866
EXPNO 1
PROCNO 1
Date_ 20130125
Time 16.08
INSTRUM spect
PROBHD 5 mm QNP 1H/13
PULPROG zg30
TD 32564
SOLVENT CDCl3
NS 16
DS 2
SWH 8223.685 Hz
FIDRES 0.252539 Hz
AQ 1.9799412 sec
RG 16
DW 60.800 usec
DE 6.50 usec
TE 300.0 K
D1 2.00000000 sec
TD0 1
======== CHANNEL f1 ========
NUC1 1H
P1 9.40 usec
PL1 -4.00 dB
PL1W 19.93825150 W
SFO1 400.1324710 MHz
SI 32768
SF 400.1300000 MHz
WDW EM
SSB 0
LB 0.30 Hz
GB 0
PC 1.00
O
N
N-allyl-N-phenylpivalamide
9 8 7 6 5 4 3 2 1 ppm
2.97 1.98 0.96 0.99 0.99 2.04 9.00

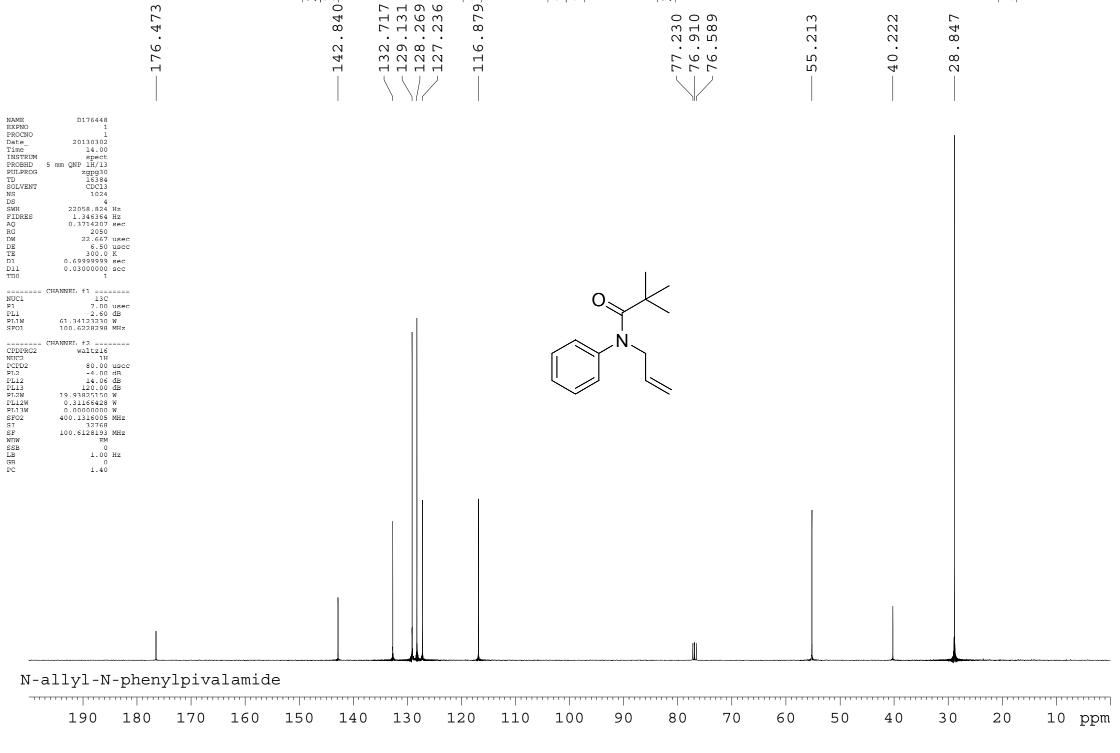
176.473 142.840 132.717 129.131 128.269 127.236 116.879 77.230 76.910 76.589 55.213 40.222 28.847
O
N
N-allyl-N-phenylpivalamide
190 180 170 160 150 140 130 120 110 100 90 80 70 60 50 40 30 20 10 ppm

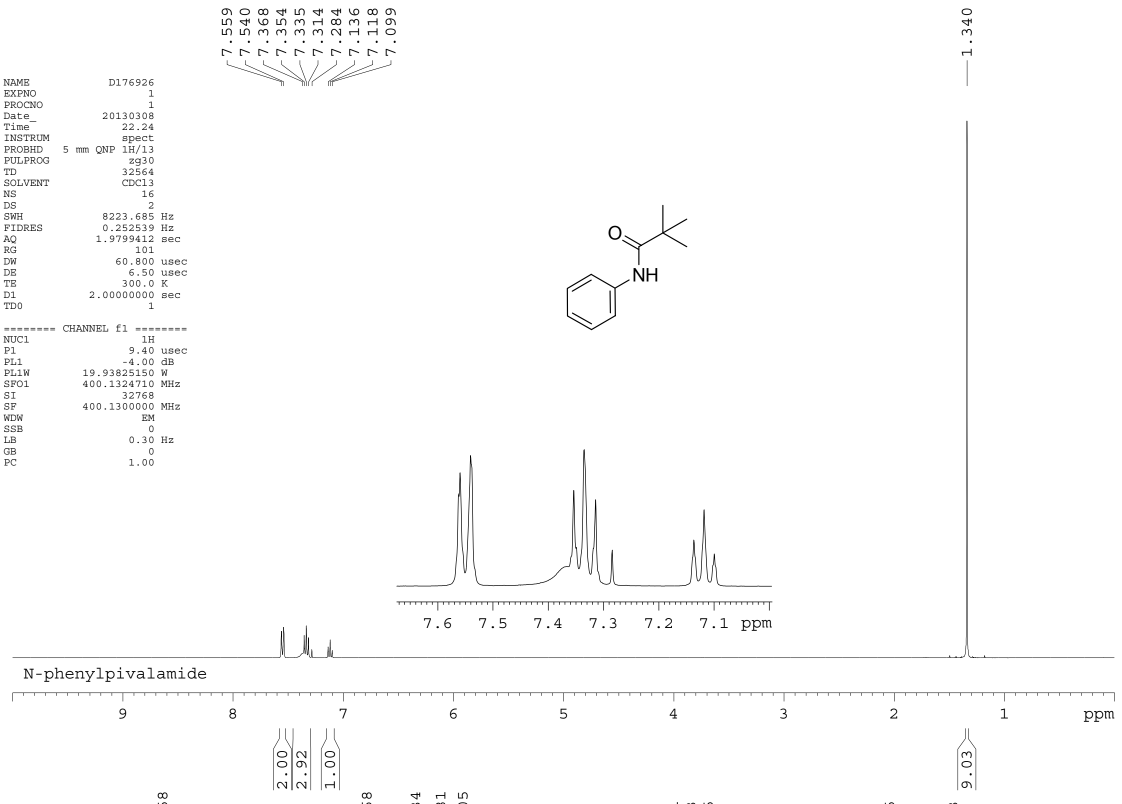
NAME D176926
EXPNO 1
PROCNO 1
Date_ 20130308
Time 22.24
INSTRUM spect
PROBHD 5 mm QNP 1H/13
PULPROG zg30
TD 32564
SOLVENT CDCl3
NS 16
DS 2
SWH 8223.685 Hz
FIDRES 0.252539 Hz
AQ 1.9799412 sec
RG 101
DW 60.800 usec
DE 6.50 usec
TE 300.0 K
D1 2.00000000 sec
TD0 1
======== CHANNEL f1 ========
NUC1 1H
P1 9.40 usec
PL1 -4.00 dB
PL1W 19.93825150 W
SFO1 400.1324710 MHz
SI 32768
SF 400.1300000 MHz
WDW EM
SSB 0
LB 0.30 Hz
GB 0
PC 1.00
7.559
7.540
7.368
7.354
7.335
7.314
7.284
7.136
7.118
7.099
1.340
O
NH
7.6 7.5 7.4 7.3 7.2 7.1 ppm
N-phenylpivalamide
9 8 7 6 5 4 3 2 1 ppm
2.00
2.92
1.00
9.03

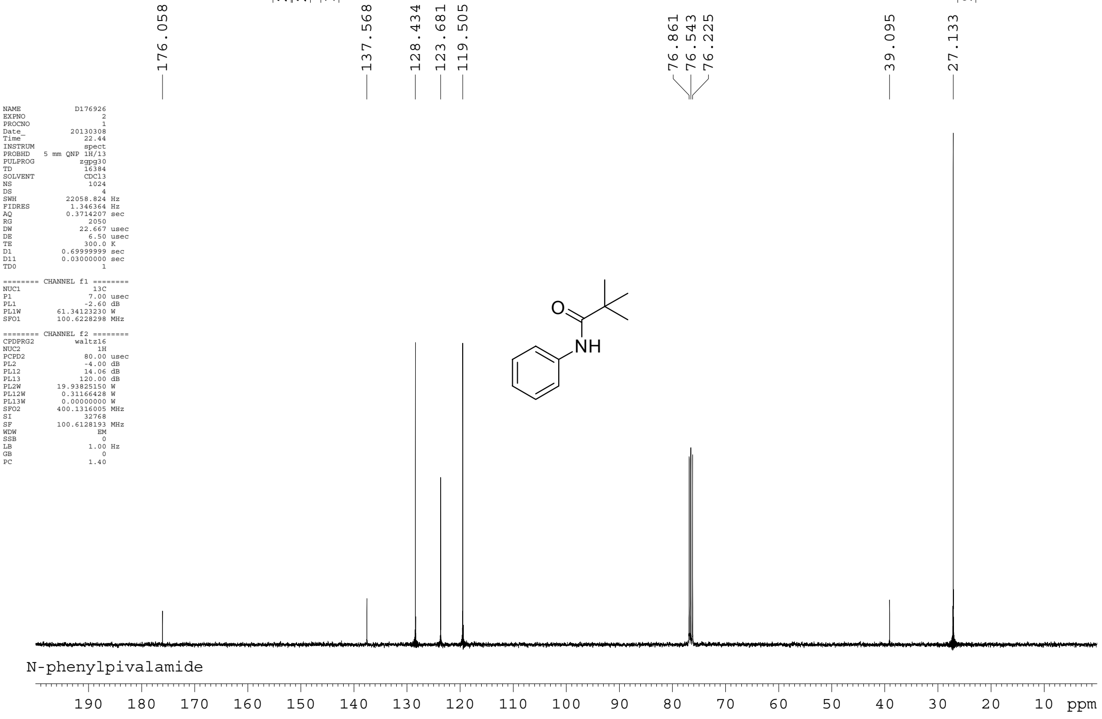
176.058
137.568
128.434
123.681
119.505
76.861
76.543
76.225
39.095
27.133
O
NH
N-phenylpivalamide
190 180 170 160 150 140 130 120 110 100 90 80 70 60 50 40 30 20 10 ppm

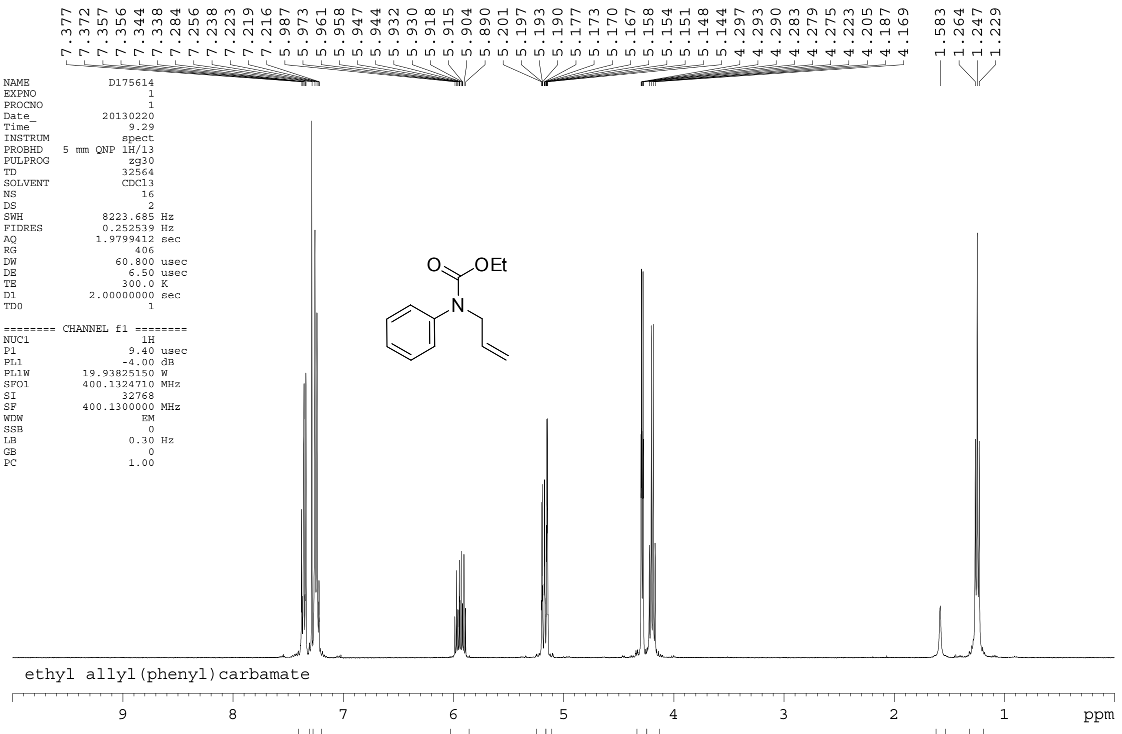
7.377 7.372 7.357 7.356 7.344 7.338 7.284 7.256 7.238 7.223 7.219 7.216 5.987 5.973 5.961 5.958 5.947 5.944 5.932 5.930 5.918 5.915 5.904 5.890 5.201 5.197 5.193 5.190 5.177 5.173 5.170 5.167 5.158 5.154 5.151 5.148 5.144 4.297 4.293 4.290 4.283 4.279 4.275 4.223 4.205 4.187 4.169 1.583 1.264 1.247 1.229
NAME D175614
EXPNO 1
PROCNO 1
Date_ 20130220
Time 9.29
INSTRUM spect
PROBHD 5 mm QNP 1H/13
PULPROG zg30
TD 32564
SOLVENT CDCl3
NS 16
DS 2
SWH 8223.685 Hz
FIDRES 0.252539 Hz
AQ 1.9799412 sec
RG 406
DW 60.800 usec
DE 6.50 usec
TE 300.0 K
D1 2.00000000 sec
TD0 1
======== CHANNEL f1 ========
NUC1 1H
P1 9.40 usec
PL1 -4.00 dB
PL1W 19.93825150 W
SFO1 400.1324710 MHz
SI 32768
SF 400.1300000 MHz
WDW EM
SSB 0
LB 0.30 Hz
GB 0
PC 1.00
O OEt
N
ethyl allyl(phenyl)carbamate
9 8 7 6 5 4 3 2 1 ppm
2.01 2.80 0.97 1.07 0.93 2.05 2.01 0.45 3.00

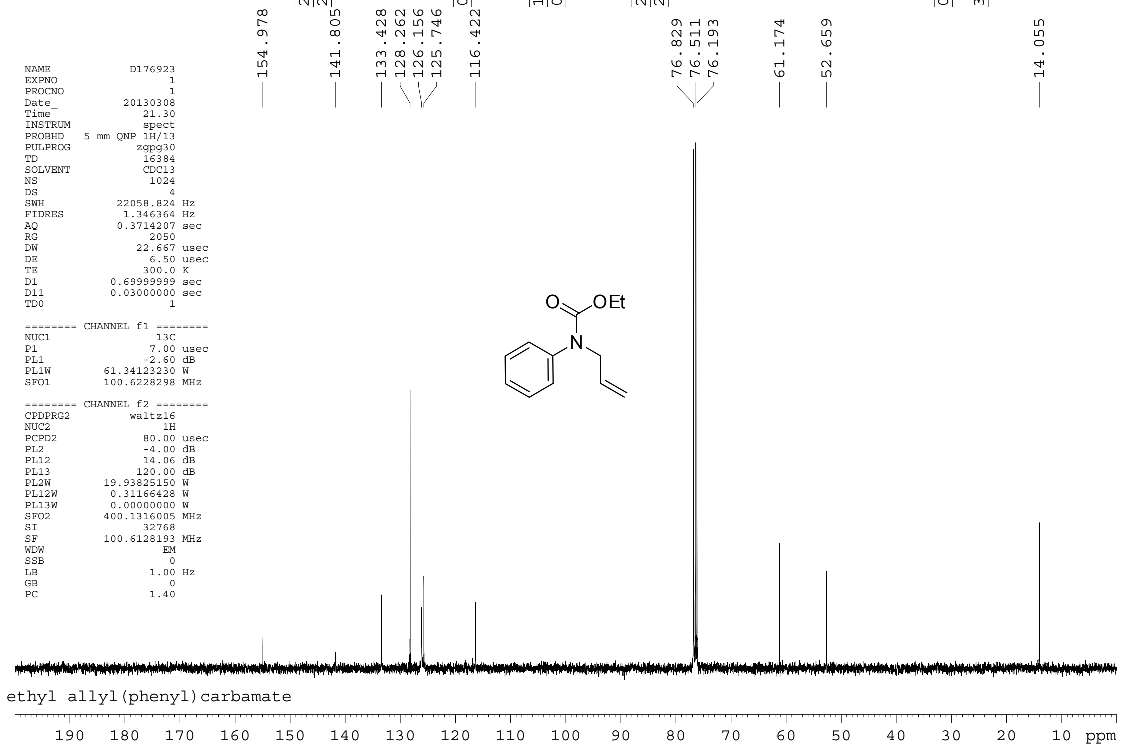
154.978 141.805 133.428 128.262 126.156 125.746 116.422 76.829 76.511 76.193 61.174 52.659 14.055
NAME D176923
EXPNO 1
PROCNO 1
Date_ 20130308
Time 21.30
INSTRUM spect
PROBHD 5 mm QNP 1H/13
PULPROG zgpg30
TD 16384
SOLVENT CDCl3
NS 1024
DS 4
SWH 22058.824 Hz
FIDRES 1.346364 Hz
AQ 0.3714207 sec
RG 2050
DW 22.667 usec
DE 6.50 usec
TE 300.0 K
D1 0.69999999 sec
D11 0.03000000 sec
TD0 1
======== CHANNEL f1 ========
NUC1 13C
P1 7.00 usec
PL1 -2.60 dB
PL1W 61.3412323 W
SFO1 100.6228298 MHz
======== CHANNEL f2 ========
CPDPRG2 waltz16
NUC2 1H
PCPD2 80.00 usec
PL2 -4.00 dB
PL12 14.06 dB
PL13 120.00 dB
PL2W 19.93825150 W
PL12W 0.31166428 W
PL13W 0.00000000 W
SFO2 400.1316005 MHz
SI 32768
SF 100.6128193 MHz
WDW EM
SSB 0
LB 1.00 Hz
GB 0
PC 1.40
O OEt
N
ethyl allyl(phenyl)carbamate
190 180 170 160 150 140 130 120 110 100 90 80 70 60 50 40 30 20 10 ppm

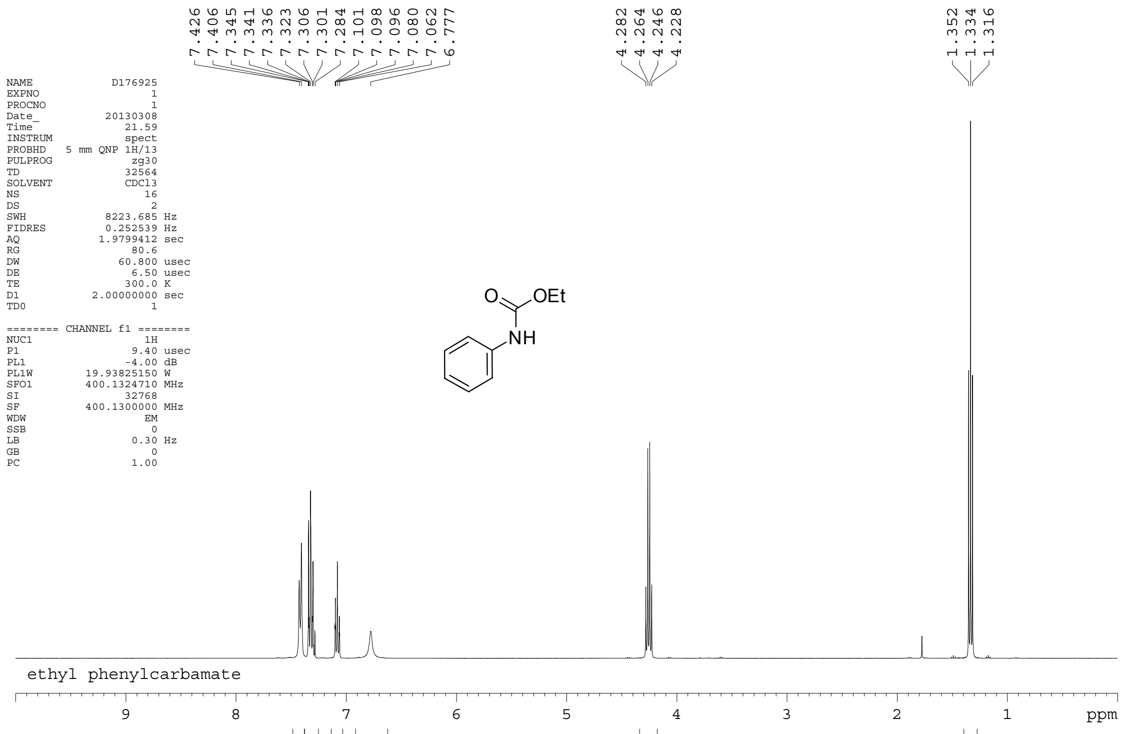
7.426
7.406
7.345
7.341
7.336
7.323
7.306
7.301
7.284
7.101
7.098
7.096
7.080
7.062
6.777
4.282
4.264
4.246
4.228
1.352
1.334
1.316
NAME D176925
EXPNO 1
PROCNO 1
Date_ 20130308
Time 21.59
INSTRUM spect
PROBHD 5 mm QNP 1H/13
PULPROG zg30
TD 32564
SOLVENT CDCl3
NS 16
DS 2
SWH 8223.685 Hz
FIDRES 0.252539 Hz
AQ 1.9799412 sec
RG 80.6
DW 60.800 usec
DE 6.50 usec
TE 300.0 K
D1 2.00000000 sec
TD0 1
======== CHANNEL f1 ========
NUC1 1H
P1 9.40 usec
PL1 -4.00 dB
PL1W 19.93825150 W
SFO1 400.1324710 MHz
SI 32768
SF 400.1300000 MHz
WDW EM
SSB 0
LB 0.30 Hz
GB 0
PC 1.00
O OEt
NH
ethyl phenylcarbamate
9
8
7
6
5
4
3
2
1
ppm
2.00
2.16
1.02
0.99
2.07
3.14

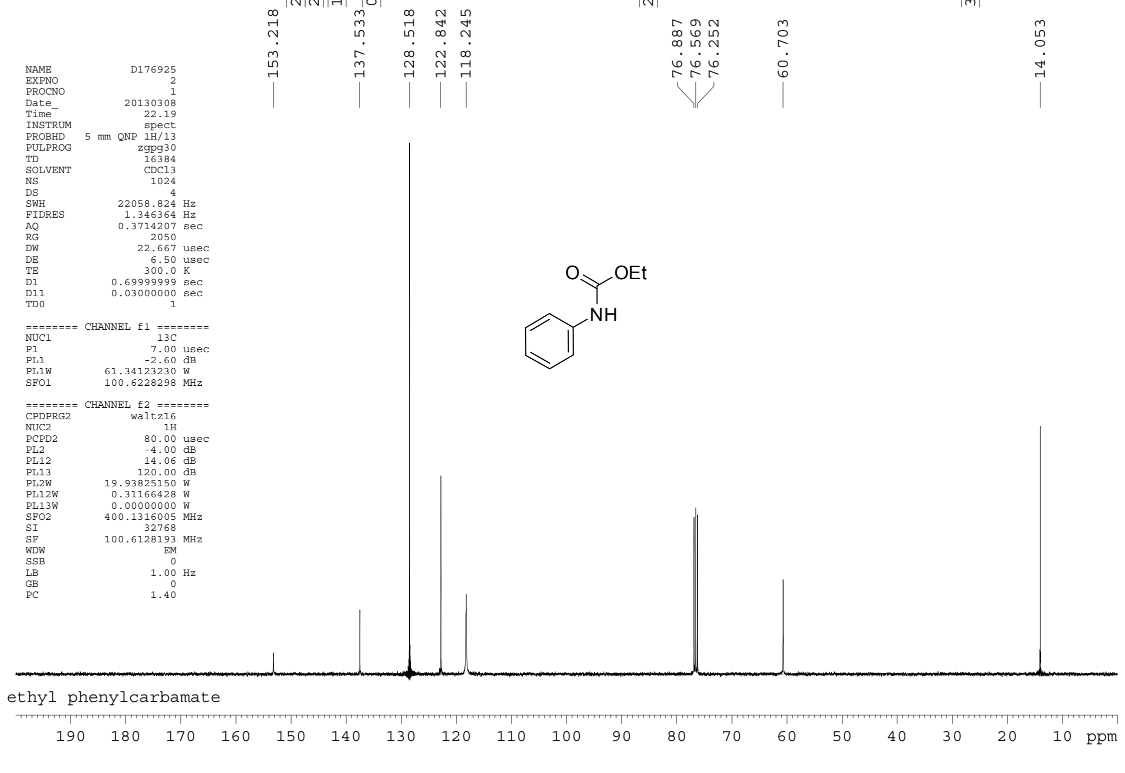
153.218
137.533
128.518
122.842
118.245
76.887
76.569
76.252
60.703
14.053
NAME D176925
EXPNO 2
PROCNO 1
Date_ 20130308
Time 22.19
INSTRUM spect
PROBHD 5 mm QNP 1H/13
PULPROG zgpg30
TD 16384
SOLVENT CDCl3
NS 1024
DS 4
SWH 22058.824 Hz
FIDRES 1.346364 Hz
AQ 0.3714207 sec
RG 2050
DW 22.667 usec
DE 6.50 usec
TE 300.0 K
D1 0.69999999 sec
D11 0.03000000 sec
TD0 1
======== CHANNEL f1 ========
NUC1 13C
P1 7.00 usec
PL1 -2.60 dB
PL1W 61.34123230 W
SFO1 100.6228298 MHz
======== CHANNEL f2 ========
CPDPRG2 waltz16
NUC2 1H
PCPD2 80.00 usec
PL2 -4.00 dB
PL12 14.06 dB
PL13 120.00 dB
PL2W 19.93825150 W
PL12W 0.31166428 W
PL13W 0.00000000 W
SFO2 400.1316005 MHz
SI 32768
SF 100.6128193 MHz
WDW EM
SSB 0
LB 1.00 Hz
GB 0
PC 1.40
O OEt
NH
ethyl phenylcarbamate
190
180
170
160
150
140
130
120
110
100
90
80
70
60
50
40
30
20
10
ppm

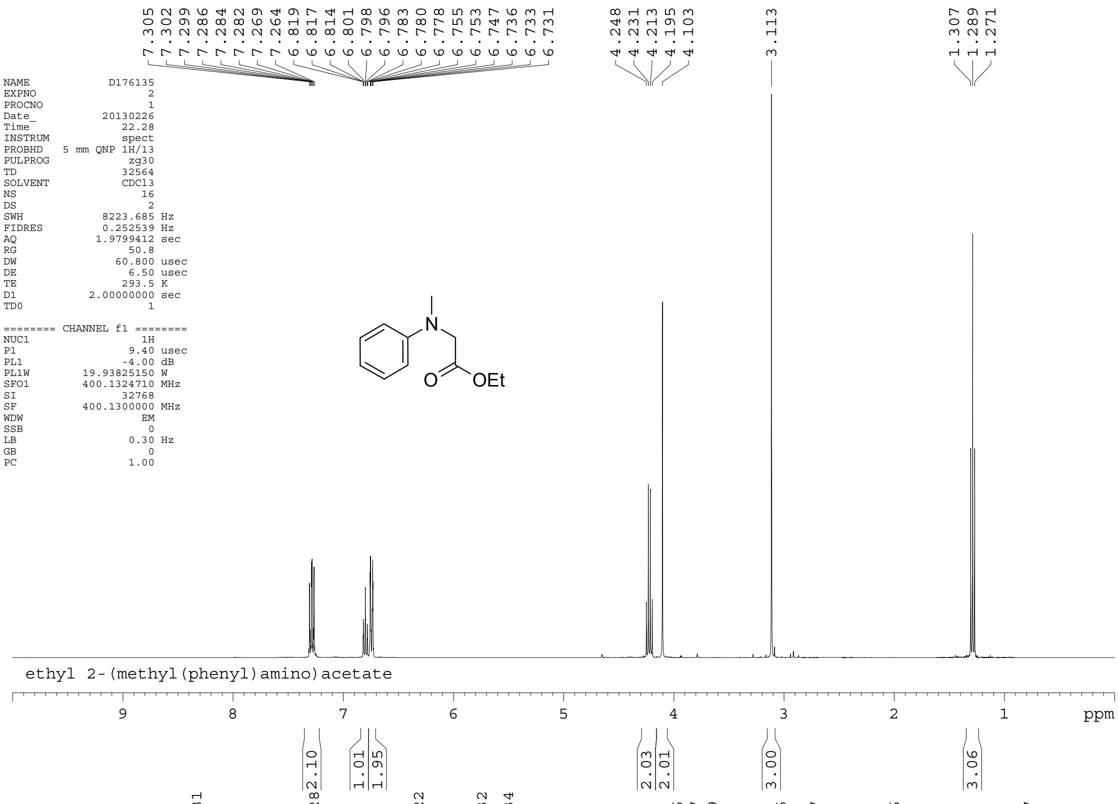
7.305 7.302 7.299 7.286 7.284 7.282 7.269 7.264 6.819 6.817 6.814 6.801 6.798 6.796 6.783 6.780 6.778 6.755 6.753 6.747 6.736 6.733 6.731
4.248 4.231 4.213 4.195 4.103
3.113
1.307 1.289 1.271
NAME D176135
EXPNO 2
PROCNO 1
Date_ 20130226
Time 22.28
INSTRUM spect
PROBHD 5 mm QNP 1H/13
PULPROG zg30
TD 32564
SOLVENT CDCl3
NS 16
DS 2
SWH 8223.685 Hz
FIDRES 0.252539 Hz
AQ 1.9799412 sec
RG 50.8
DW 60.800 usec
DE 6.50 usec
TE 293.5 K
D1 2.00000000 sec
TD0 1
======== CHANNEL f1 ========
NUC1 1H
P1 9.40 usec
PL1 -4.00 dB
PL1W 19.93825150 W
SFO1 400.1324710 MHz
SI 32768
SF 400.1300000 MHz
WDW EM
SSB 0
LB 0.30 Hz
GB 0
PC 1.00
N
O
OEt
ethyl 2-(methyl(phenyl)amino)acetate
9 8 7 6 5 4 3 2 1 ppm
2.10 1.01 1.95 2.03 2.01 3.00 3.06

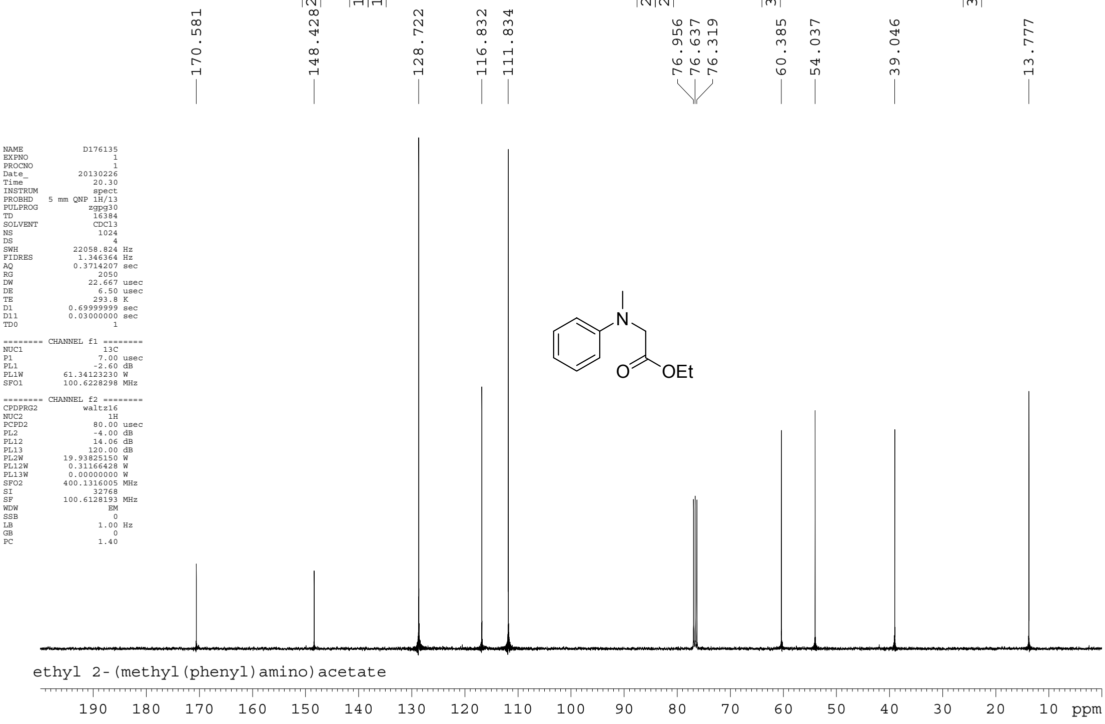
170.581
148.428
128.722
116.832
111.834
76.956 76.637 76.319
60.385
54.037
39.046
13.777
N
O
OEt
ethyl 2-(methyl(phenyl)amino)acetate
190 180 170 160 150 140 130 120 110 100 90 80 70 60 50 40 30 20 10 ppm

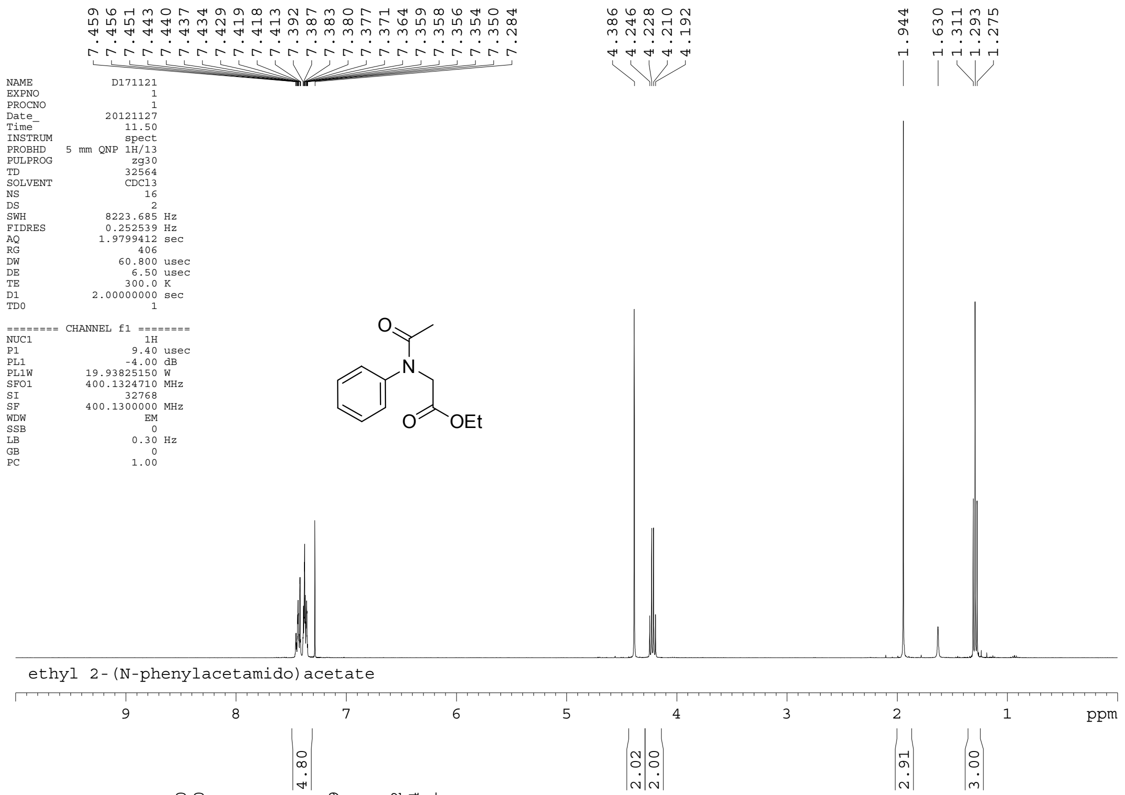
7.459 7.456 7.451 7.443 7.440 7.437 7.434 7.429 7.419 7.418 7.413 7.392 7.387 7.383 7.380 7.377 7.371 7.364 7.359 7.358 7.356 7.354 7.350 7.284
4.386 4.246 4.228 4.210 4.192
1.944 1.630 1.311 1.293 1.275
NAME D171121
EXPNO 1
PROCNO 1
Date_ 20121127
Time 11.50
INSTRUM spect
PROBHD 5 mm QNP 1H/13
PULPROG zg30
TD 32564
SOLVENT CDCl3
NS 16
DS 2
SWH 8223.685 Hz
FIDRES 0.252539 Hz
AQ 1.9799412 sec
RG 406
DW 60.800 usec
DE 6.50 usec
TE 300.0 K
D1 2.00000000 sec
TD0 1
======== CHANNEL f1 ========
NUC1 1H
P1 9.40 usec
PL1 -4.00 dB
PL1W 19.93825150 W
SFO1 400.1324710 MHz
SI 32768
SF 400.1300000 MHz
WDW EM
SSB 0
LB 0.30 Hz
GB 0
PC 1.00
O
N
O
OEt
ethyl 2-(N-phenylacetamido)acetate
9 8 7 6 5 4 3 2 1 ppm
4.80
2.02
2.00
2.91
3.00

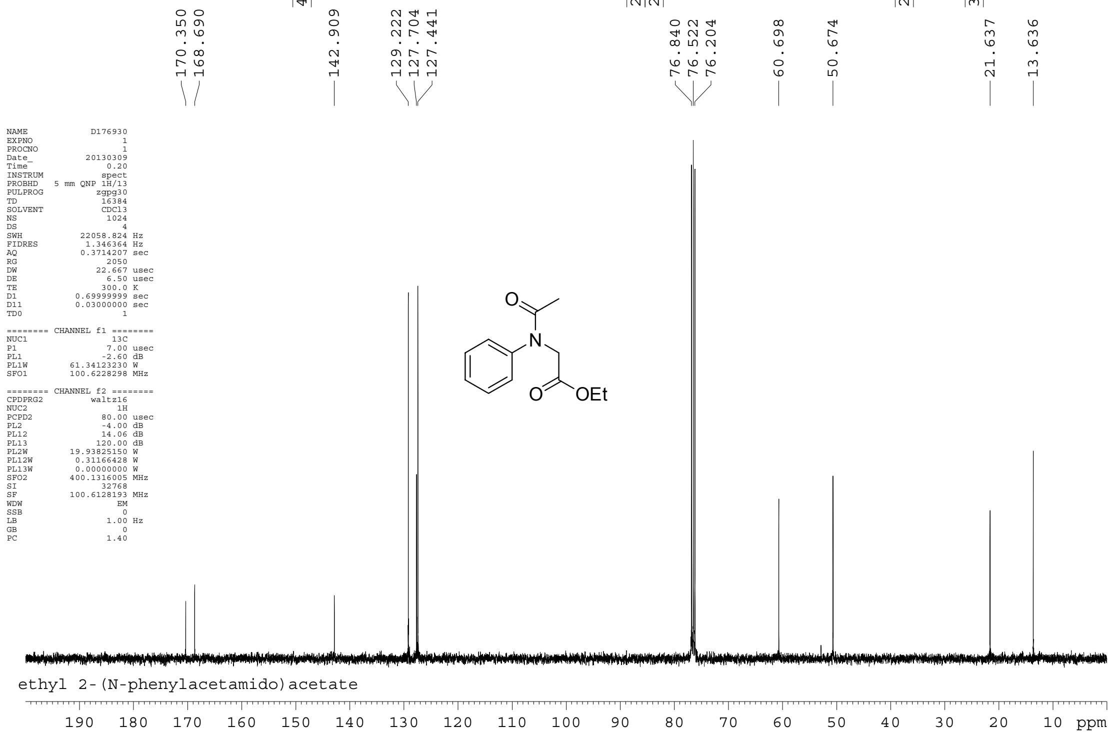
170.350 168.690
142.909
129.222 127.704 127.441
76.840 76.522 76.204
60.698
50.674
21.637
13.636
O
N
O
OEt
ethyl 2-(N-phenylacetamido)acetate
190 180 170 160 150 140 130 120 110 100 90 80 70 60 50 40 30 20 10 ppm

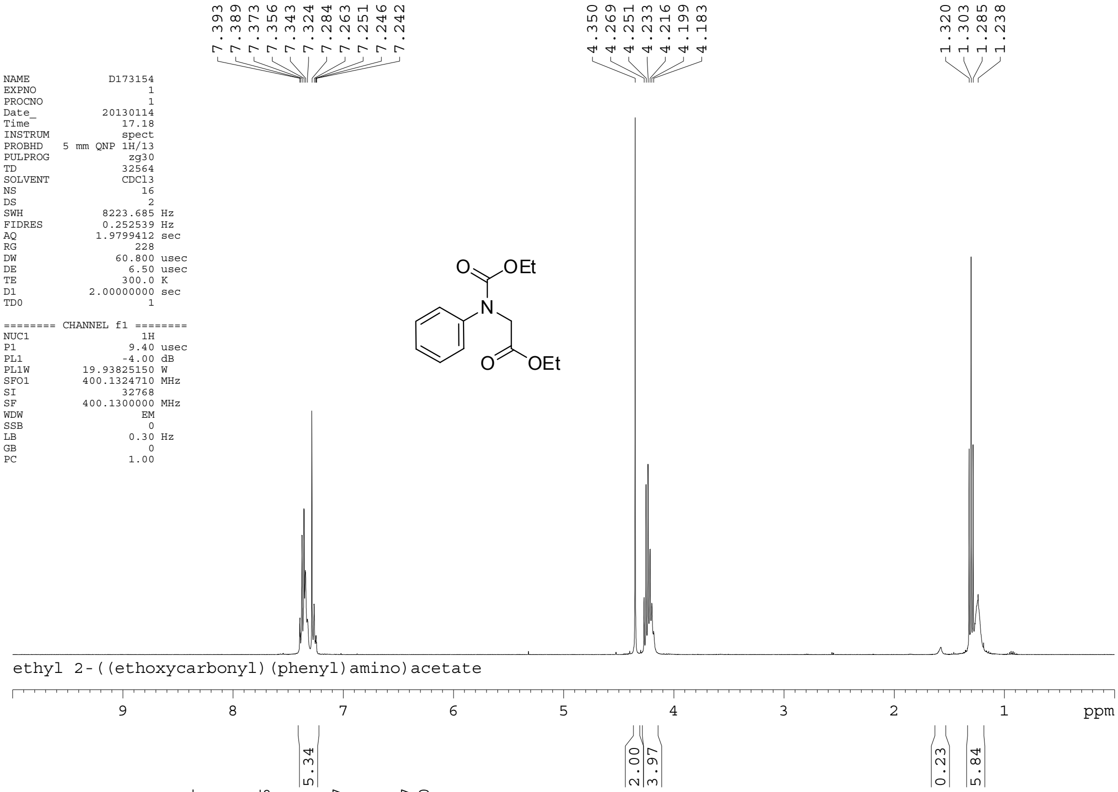
7.393 7.389 7.373 7.356 7.343 7.324 7.284 7.263 7.251 7.246 7.242
4.350 4.269 4.251 4.233 4.216 4.199 4.183
1.320 1.303 1.285 1.238
NAME D173154
EXPNO 1
PROCNO 1
Date_ 20130114
Time 17.18
INSTRUM spect
PROBHD 5 mm QNP 1H/13
PULPROG zg30
TD 32564
SOLVENT CDCl3
NS 16
DS 2
SWH 8223.685 Hz
FIDRES 0.252539 Hz
AQ 1.9799412 sec
RG 228
DW 60.800 usec
DE 6.50 usec
TE 300.0 K
D1 2.00000000 sec
TD0 1
======== CHANNEL f1 ========
NUC1 1H
P1 9.40 usec
PL1 -4.00 dB
PL1W 19.93825150 W
SFO1 400.1324710 MHz
SI 32768
SF 400.1300000 MHz
WDW EM
SSB 0
LB 0.30 Hz
GB 0
PC 1.00
O OEt
N
O OEt
ethyl 2-((ethoxycarbonyl)(phenyl)amino)acetate
9 8 7 6 5 4 3 2 1 ppm
5.34
2.00 3.97
0.23 5.84

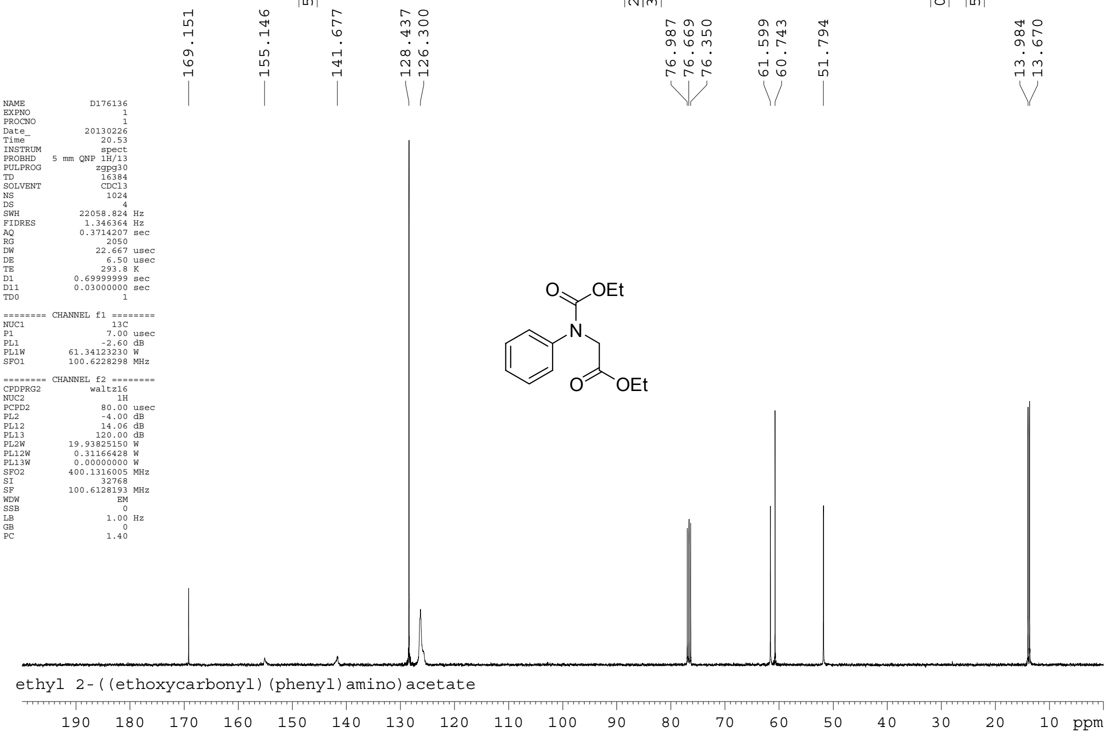
169.151
155.146
141.677
128.437 126.300
76.987 76.669 76.350
61.599 60.743
51.794
13.984 13.670
O OEt
N
O OEt
ethyl 2-((ethoxycarbonyl)(phenyl)amino)acetate
190 180 170 160 150 140 130 120 110 100 90 80 70 60 50 40 30 20 10 ppm

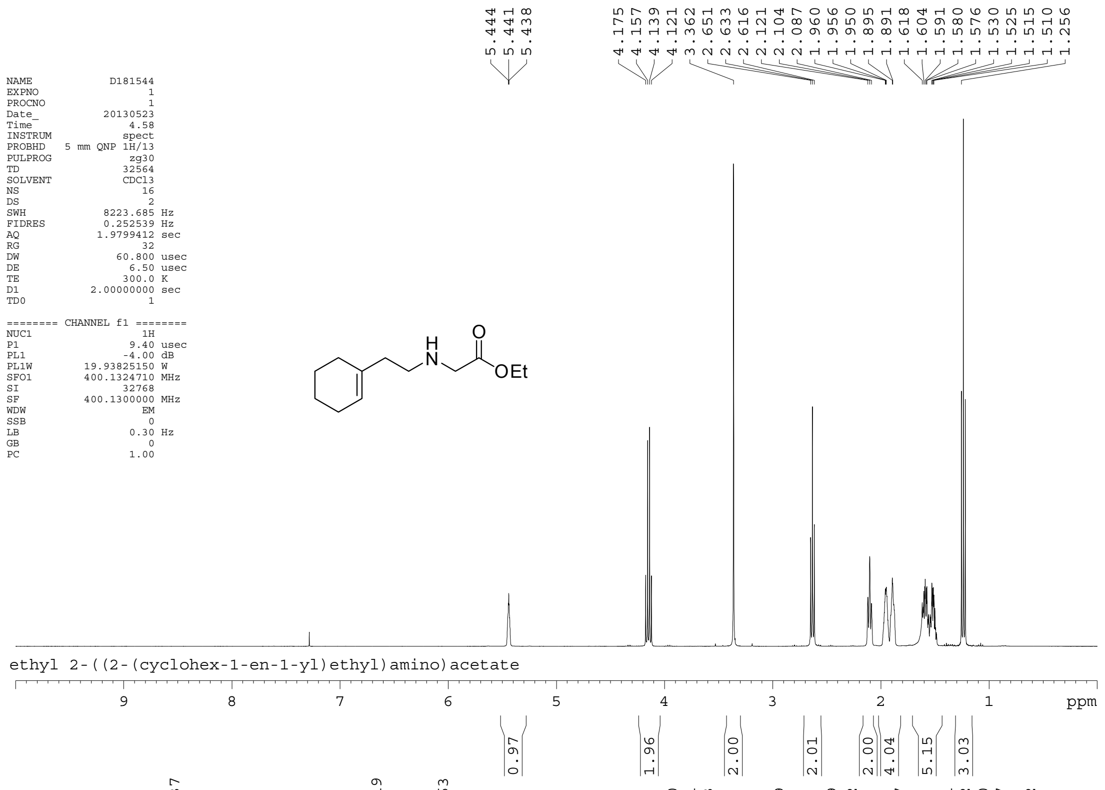
ethyl 2-((2-(cyclohex-1-en-1-yl)ethyl)amino)acetate

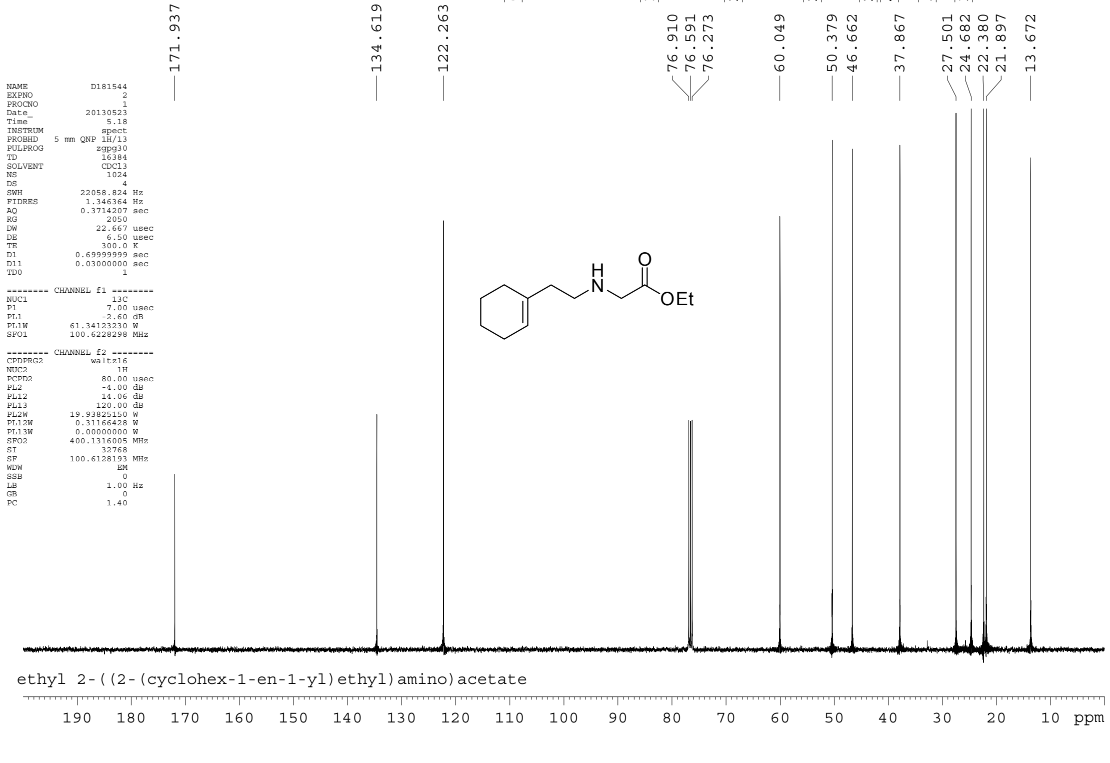
ethyl 2-((2-(cyclohex-1-en-1-yl)ethyl)amino)acetate

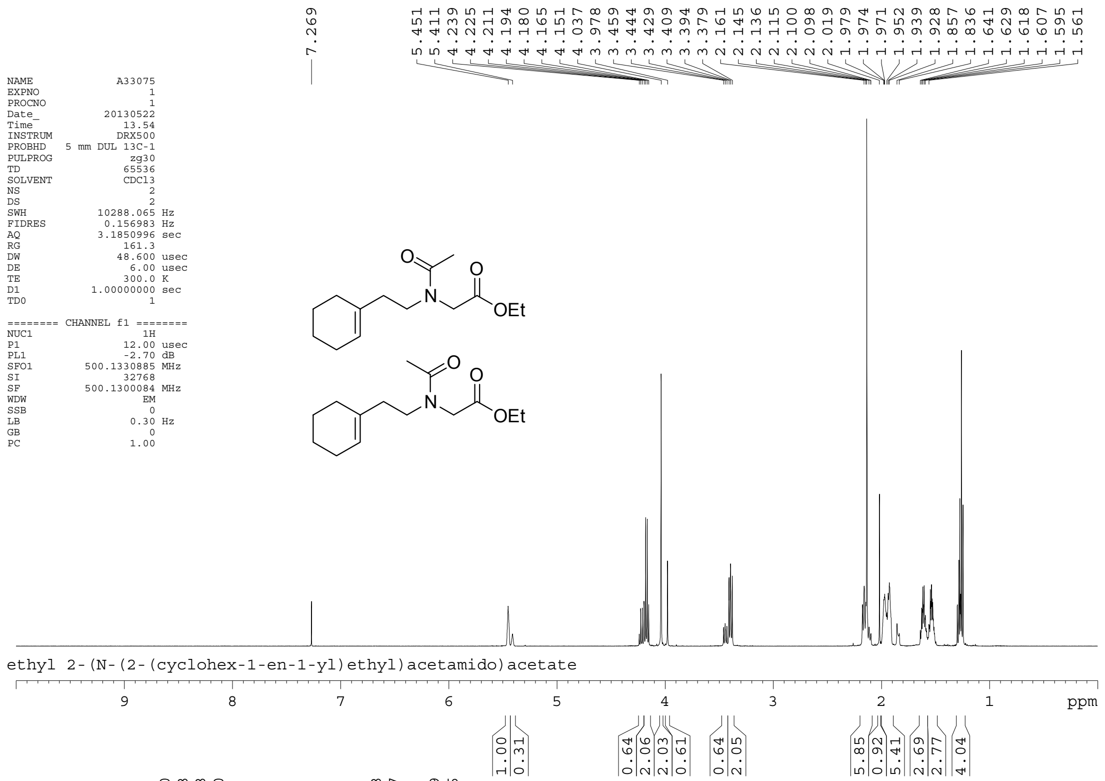
ethyl 2-(N-(2-(cyclohex-1-en-1-yl)ethyl)acetamido)acetate
7.269
5.451 5.411 4.239 4.225 4.211 4.194 4.180 4.165 4.151 4.037 3.978 3.459 3.444 3.429 3.409 3.394 3.379 2.161 2.145 2.136 2.115 2.100 2.098 2.019 1.979 1.974 1.971 1.952 1.939 1.928 1.857 1.836 1.641 1.629 1.618 1.607 1.595 1.561
1.00 0.31 0.64 2.06 2.03 0.61 0.64 2.05 5.85 0.92 5.41 2.69 2.77 4.04
OEt
ppm

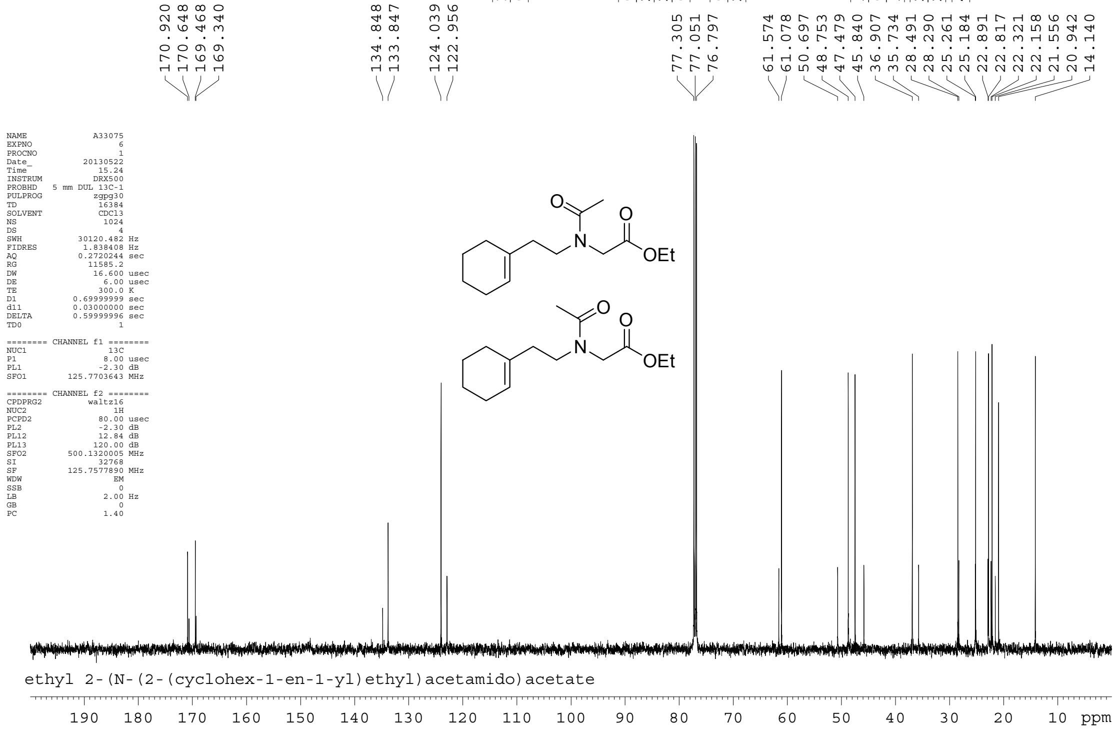
ethyl 2-(N-(2-(cyclohex-1-en-1-yl)ethyl)acetamido)acetate
170.920 170.648 169.468 169.340 134.848 133.847 124.039 122.956 77.305 77.051 76.797 61.574 61.078 50.697 48.753 47.479 45.840 36.907 35.734 28.491 28.290 25.261 25.184 22.891 22.817 22.321 22.158 21.556 20.942 14.140
OEt
ppm

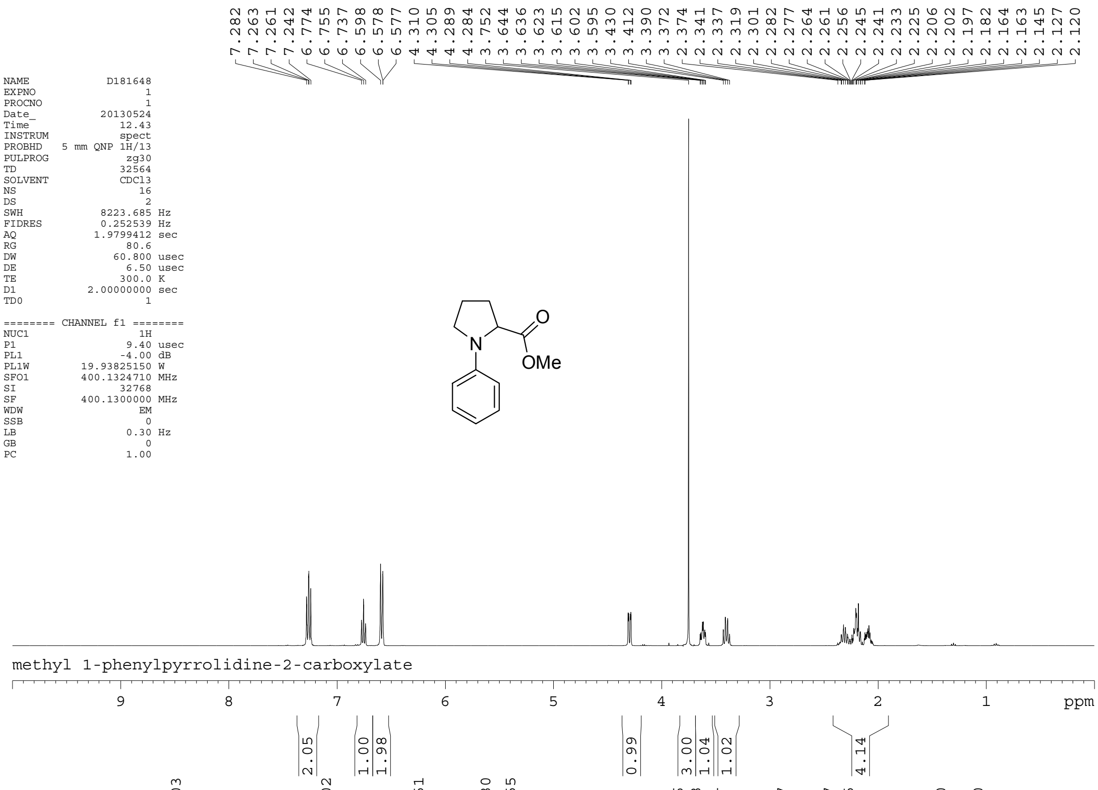
methyl 1-phenylpyrrolidine-2-carboxylate
OMe
ppm

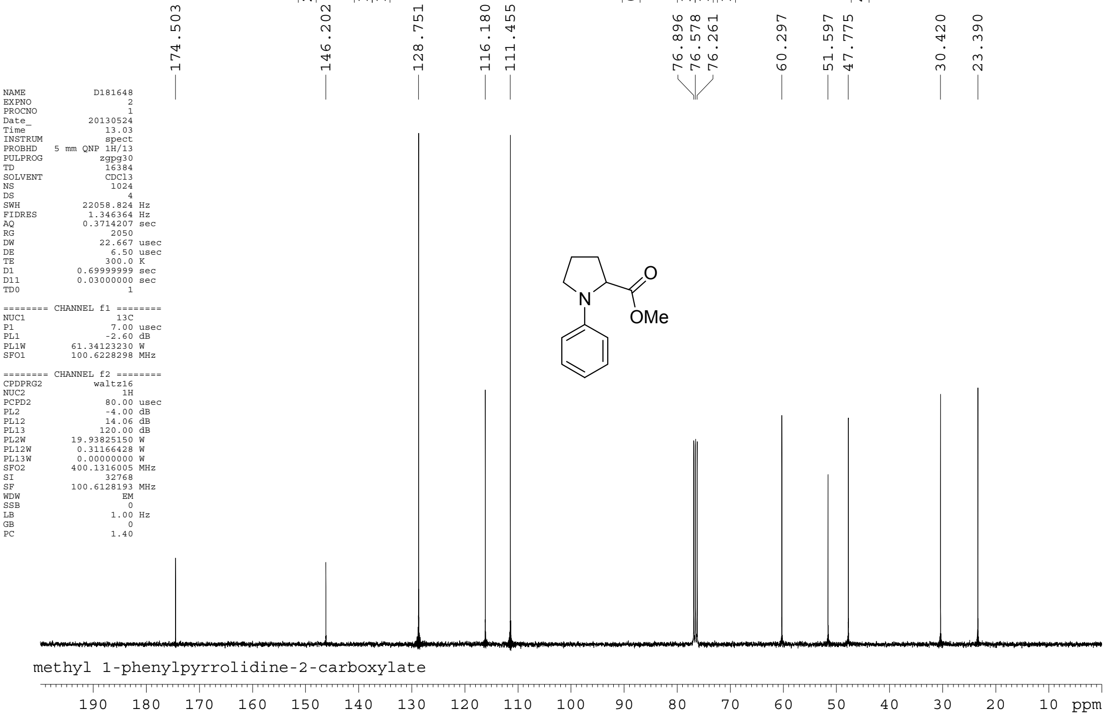
174.503
146.202
128.751
116.180
111.455
76.896
76.578
76.261
60.297
51.597
47.775
30.420
23.390
OMe
methyl 1-phenylpyrrolidine-2-carboxylate
ppm

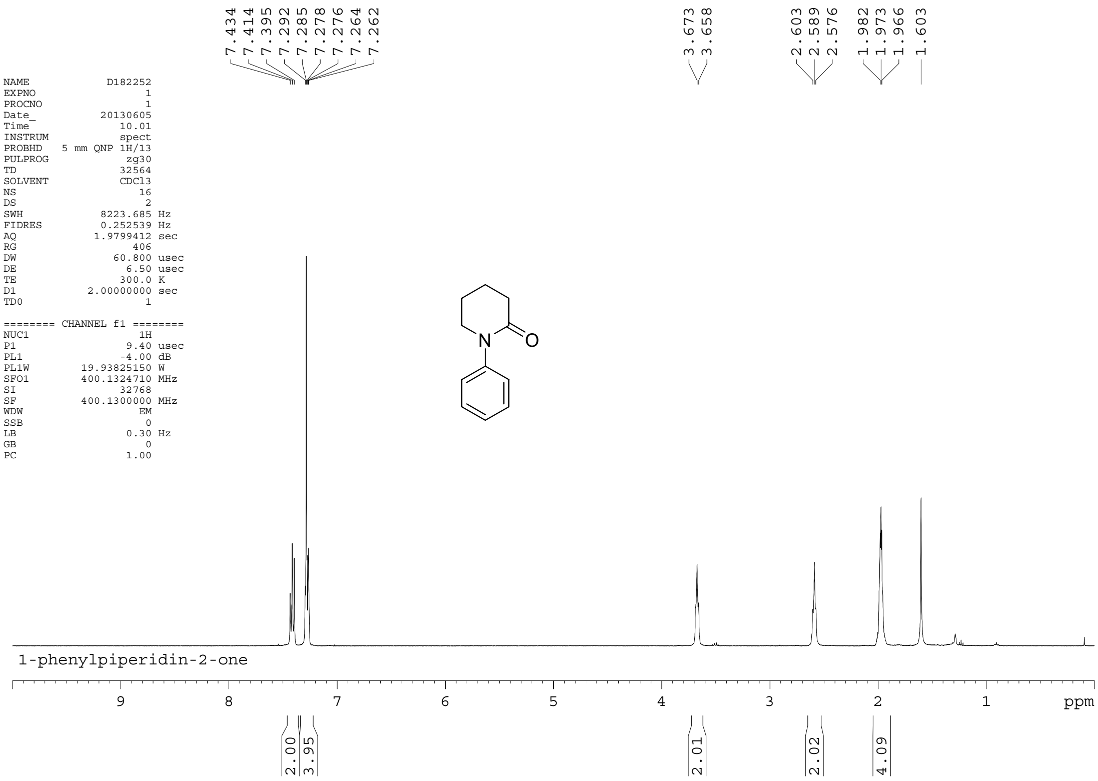
7.434 7.414 7.395 7.292 7.285 7.278 7.276 7.264 7.262
3.673 3.658
2.603 2.589 2.576
1.982 1.973 1.966
1.603
NAME D182252
EXPNO 1
PROCNO 1
Date_ 20130605
Time 10.01
INSTRUM spect
PROBHD 5 mm QNP 1H/13
PULPROG zg30
TD 32564
SOLVENT CDCl3
NS 16
DS 2
SWH 8223.685 Hz
FIDRES 0.252539 Hz
AQ 1.9799412 sec
RG 406
DW 60.800 usec
DE 6.50 usec
TE 300.0 K
D1 2.00000000 sec
TD0 1
======== CHANNEL f1 ========
NUC1 1H
P1 9.40 usec
PL1 -4.00 dB
PL1W 19.93825150 W
SFO1 400.1324710 MHz
SI 32768
SF 400.1300000 MHz
WDW EM
SSB 0
LB 0.30 Hz
GB 0
PC 1.00
N
O
1-phenylpiperidin-2-one
9 8 7 6 5 4 3 2 1 ppm
2.00 3.95 2.01 2.02 4.09

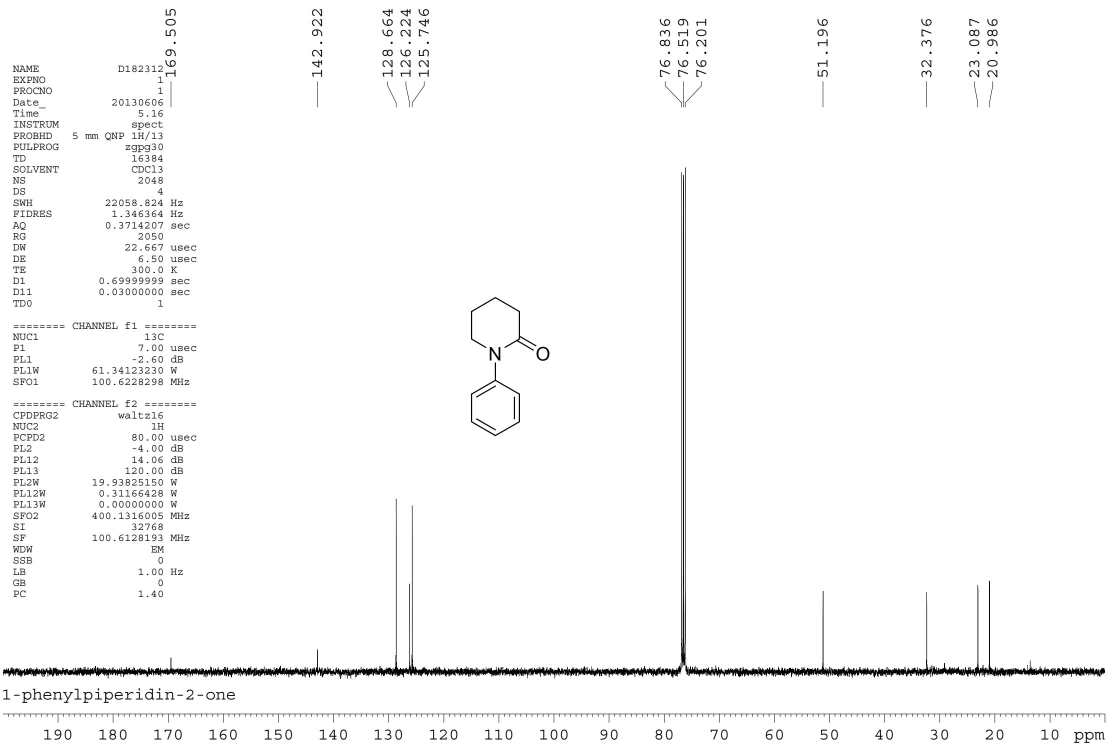
169.505
142.922
128.664 126.224 125.746
76.836 76.519 76.201
51.196
32.376
23.087 20.986
NAME D182312
EXPNO 1
PROCNO 1
Date_ 20130606
Time 5.16
INSTRUM spect
PROBHD 5 mm QNP 1H/13
PULPROG zgpg30
TD 16384
SOLVENT CDCl3
NS 2048
DS 4
SWH 22058.824 Hz
FIDRES 1.346364 Hz
AQ 0.3714207 sec
RG 2050
DW 22.667 usec
DE 6.50 usec
TE 300.0 K
D1 0.69999999 sec
D11 0.03000000 sec
TD0 1
======== CHANNEL f1 ========
NUC1 13C
P1 7.00 usec
PL1 -2.60 dB
PL1W 61.34123230 W
SFO1 100.6228298 MHz
======== CHANNEL f2 ========
CPDPRG2 waltz16
NUC2 1H
PCPD2 80.00 usec
PL2 -4.00 dB
PL12 14.06 dB
PL13 120.00 dB
PL2W 19.93825150 W
PL12W 0.31166428 W
PL13W 0.00000000 W
SFO2 400.1316005 MHz
SI 32768
SF 100.6128193 MHz
WDW EM
SSB 0
LB 1.00 Hz
GB 0
PC 1.40
N
O
1-phenylpiperidin-2-one
190 180 170 160 150 140 130 120 110 100 90 80 70 60 50 40 30 20 10 ppm

References

(1) E. Doni, S. O'Sullivan, J. A. Murphy, *Angew. Chem., Int. Ed.* **2013**, *52*, 2239.
(2) F. Schoenebeck; J.A. Murphy; S.Z. Zhou; Y. Uenoyama; Y. Miclo; Tuttle, T. *J. Am. Chem. Soc.* **2007**, *129*, 13368.
(3) N. J. Findlay, S. R. Park, F. Schoenebeck, E. Cahard, S.Z. Zhou, L.E. A. Berlouis, M. D. Spicer, T. Tuttle, J. A. Murphy, *J. Am. Chem. Soc.* **2010**, *132*, 15462.
(4) S. Kim, T. A. Lee, *Synlett* **1997**, 950.
(5) H. Naito, T. Hata, H. Urabe,. *Org. Lett.* **2010**, *12*, 1228.
(6) J. C. Shain, U. K. Khamrai, A. Basak, *Tetrahedron Lett.* **1997**, *38*, 6067.
(7) K. Ito, H. Tanaka, H. *Chem. Pharm. Bull.* **1977**, *25*, 1732.
(8) S. Cicchi, M. Bonanni, F. Cardona, J. Revuelta, A. Goti, *Org. Lett.* **2003**, *5*, 1773.
(9) H. F. Hodson, D. J. Madge, A. N. Slawin, D. A. Widowson, D. J. Williams, *Tetrahedron* **1994**, *50*, 1899.
(10) H. R. Snyder, E. P. Merica, C. R. Force, E. G. White,*J. Am. Chem. Soc.* **1958**, 4622.
(11) Z. Liu, R. C. Larock, *J. Org. Chem* **2006**, *71*, 3198.
(12) Z. Zhang, J. Mao, D. Zhu, F. Wu, H. Chen, B.Wan, *Tetrahedron* **2006**, *62*, 4435.
(13) A. J. Bridges, J. P. Sanchez,. *J. Het. Chem.* **1990**, *27*, 1527.
(14) H. Hoffmann, G. Platz, W. Ulbricht, *J. Phys. Chem.* **1981**, *85*, 1418.
(15) S. Sridhar, B. Srinivas, V. P. Kumar, M. Narender, K. R. Rao, *Adv. Synth. Catal.* **2007**, *349*, 1873.
(16) E. H. White, H. M. Lim, *J. Org. Chem.* **1987**, *52*, 2162.
(17) N. Torimoto, T. Shingaki, T.Nagai, *J. Org. Chem.* **1978**, *43*, 631.
(18) L. De Luca, G. Giacomelli, *J. Org. Chem.* **2008**, *73*, 3967.
(19) H. Naito, T. Hata, H. Urabe, *Org. Lett.* **2010**, *12*, 1228.
(20) S.F. Wang, C.-P. Chuang, J.-H. Lee, S.-T.; Liu, *Tetrahedron,* **1999**, *55*, 2273.
(21) B. A. Gellert, N. Kahlcke, M. Feurer, S. Roth, S. *Chem. Eur. J.* **2011**, *17*, 12203.
(22) P. Fleming, D. F. O'Shea, *J. Am. Chem. Soc.* **2011**, *133*, 1698.
(23) T. Shingaki, M. Inagaki, N. Torimoto, M. Takebayashi, M. *Chem. Lett.* **1972**, *1*, 1181.
(24) Gary, L., Pub. No. US5081261 (A), January 14th, 1992
(25) O. Chantarasriwong, D. O. Jang, W. Chavasiri, *Tetrahedron Lett.* **2006**, *47*, 7489.
(26) T. Ankner, G. R. Hilmersson, *Org. Lett.* **2009**, *11*, 503.
(27) X. Zhao, D. Liu, H. Guo, Y. Liu, W. Zhang, W. *J. Am. Chem. Soc.* **2011**, *133*, 19354.
(28) M. T. Barros, S. S. Dey, C. D. Maycock, *Eur. J. Org. Chem.* **2013**, *2013*, 742.
(29) B. Schmidt, S. Krehl, E. Jablowski, E. *Org. Biomol. Chem.* **2012**, *10*, 5119.
(30) H. Kim, C. Lee, C. *Angew. Chem.* **2012**, *124*, 12469.
(31) M. J. Zacuto, F. Xu, *J. Org. Chem.* **2007**, *72*, 6298.
(32) J. E. Herweh, C.E. Hoyle, *J. Org. Chem.* **1980**, *45*, 2195.
(33) Y. Wakita, S.-Y. Noma, M. Maeda, M. Kojima, *J. Organomet. Chem.* **1985**, *297*, 379.
(34) E. Tayama, T. Yanaki, H. Iwamoto, E. Hasegawa, *Eur. J. Org. Chem.* **2010**, *2010*, 6719.
(35) V. Pace, W. Holzer, G. Verniest, A. R. Alcántara, N. De Kimpe, N. *Adv. Synth. Catal.* **2013**, *355*, 919.
(36) B.T. Gillis, J. G. Dain, *J. Het. Chem.* **1971**, *8*, 339.
(37) M. Yamagishi, K. Nishigai, A. Ishii, T. Hata, H. Urabe, *Angew. Chem., Int. Ed.* **2012**, *51*, 6471.
(38) B. Jiang, Z.-G. Huang, K.-J. Cheng, *Tetrahedron: Asymmetry* **2006**, *17*, 942.
(39) Y. Zhao, S. W. Foo, S. Saito, *Angew. Chem., Int. Ed.* **2011**, *50*, 3006.

(40) M. Zhang, S. Imm, S. Bähn, L. Neubert, H. Neumann, M. Beller, *Angew. Chem., Int. Ed.* **2012**, *51*, 3905.
(41) Z. J. Kamiński, *Tetrahedron Lett.* **1985**, *26*, 2901.
(42) T. W. M. Spence, G. Tennant, *J. Chem. Soc., Perkin Trans. 1,* **1972**, 97.
(43) A. A. Fesenko, L. A. Trafimova, A. D. Shutalev, *Org. Biomol. Chem.* **2012**, *10*, 447.
(44) J. Yin, S. L. Buchwald, *Org. Lett.* **2000**, *2*, 1101.
(45) W. Kohn, L. J. Sham, *Phys. Rev.* **1965**, *140*, A1133.
(46) R. G. Parr; W. T. Yang *Density Functional Theory of Atoms and Molecules*; Oxford University Press: New York, 1989.
(47) S. Grimme, *J. Comput. Chem.* **2006**, *27*, 1787.
(48) R. Krishnan, J. S. Binkley, R. Seeger, J. A. Pople, *J. Phys. Chem.* **1980**, *72*, 650.
(49) A. D. McLean, G. S. Chandler, *J. Phys. Chem.* **1980**, *72*, 5639.
(50) J. Tomasi, B. Mennucci, R. Cammi, R. *Chem. Rev.* **2005**, *105*, 2999.
(51) M. J. Frisch, G. W. Trucks, H. B. Schlegel, G. E. Scuseria, M. A. Robb, J. R. Cheeseman, G. Scalman`, V. Barone, B. Mennucci, G. A. Petersson, H. Nakatsuji, M. Caricato, X. Li, H. P. Hratchian, A. F. Izmaylov, J. Bloino, G. Zheng, J. L. Sonnenberg, M. Hada, M. Ehara, K. Toyota, R. Fukuda, J. Hasegawa, M. Ishid, T. Nakajima, Y. HondA, O. Kitao, H. Nakai, T. Vreven, J. A. Montgomery, J. E. Peralta, F. Ogliaro, M. Bearpark, J. J. Heyd, E. Brothers, K. N. Kudin, V. N. Staroverov, R. Kobayashi, J. Normand, K. Raghavachari, A. Rendell, J. C. Burant, S. S. Iyengar, J. Tomasi, M. Cossi, N. Rega, J. M. Millam, M. Klene, J. E. Knox, J. B. Cross, V. Bakken, C Adamo, J. Jaramillo, R. Gomperts, R. E. Stratmann, O. Yazyev, A. J. Austin, R. Cammi, C. Pomelli, J. W. Ochterski, R. L. Martin, K. Morokuma, V. G. Zakrzewski, G. A. Voth, P. Salvador, J. J. Dannenberg, S. Dapprich, A. D. Daniels, O. Farkas, J. B. Foresman, J. V. Ortiz, J. Cioslowski` D. J. Fox, Gaussian 09, A.02 Gaussian, Inc.: Wallingford, CT, 2009.
(52) P. Maslak, R. D. Guthrie, *J. Am. Chem. Soc.* **1986**, *108*, 2628.
(53) P. Maslak, R. D. Guthrie, *J. Am. Chem. Soc.* **1986**, *108*, 2637.
